# Ginsenoside Rh2 repressed the progression of prostate cancer through the mitochondrial damage induced by mitophagy and ferroptosis

**DOI:** 10.3389/fonc.2025.1633891

**Published:** 2025-08-21

**Authors:** Zhen He, Jianxi Shi, Bing Zhu, Zhentao Tian, Zhihong Zhang, Changwen Zhang

**Affiliations:** ^1^ Department of Urology, First Teaching Hospital of Tianjin University of Traditional Chinese Medicine, National Clinical Research Center for Chinese Medicine Acupuncture and Moxibustion, Tianjin, China; ^2^ Department of Urology, Tianjin Institute of Urology, The Second Hospital of Tianjin Medical University, Tianjin, China

**Keywords:** ginsenoside Rh2, PC, mitochondrial damage, mitophagy, ferroptosis, Chinese medicine

## Abstract

**Introduction:**

Prostate cancer (PC), the most common male genitourinary malignancy and second leading cause of global cancer deaths in men, frequently progresses to lethal castration-resistant PC (CRPC). Ginsenoside Rh2 (GRh2), a ginseng-derived bioactive compound, exhibits antitumor potential, but its efficacy and mechanisms in PC remain unclear.

**Methods:**

PC3 cells were treated with GRh2 to assess proliferation (IC50 calculation), migration, and invasion. Mitochondrial function (membrane potential, ROS, ATP/ADP), mitophagy markers (PINK1/Parkin, VDAC1/TOM20, autophagosomes), and ferroptosis indicators (lipid ROS, MDA, Fe^2+^, GSH, SLC7A11/GPX4) were evaluated. Specific inhibitors (Mdivi-1 for mitophagy, Fer-1 for ferroptosis) validated mechanistic causality. Subcutaneous xenograft models in nude mice assessed in vivo efficacy.

**Results:**

GRh2 potently inhibited PC3 cell proliferation (IC50 = 19.3 μg/mL), migration, and invasion. It induced mitochondrial dysfunction (depolarized membrane, elevated ROS, disrupted ATP/ADP) and activated mitophagy, evidenced by upregulated PINK1/Parkin, reduced VDAC1/TOM20, and autophagosome accumulation. Concurrently, GRh2 triggered ferroptosis via lipid ROS accumulation, increased MDA/Fe^2+^, GSH depletion, and SLC7A11/GPX4 downregulation. All effects were reversed by Mdivi-1 or Fer-1, confirming pathway-specific causality. *In vivo*, GRh2 significantly suppressed tumor growth.

**Discussion:**

This study provides the first evidence that GRh2 exerts synergistic antitumor effects in PC through dual induction of mitophagy-associated mitochondrial damage and ferroptosis. The reversibility of both pathways by specific inhibitors establishes a causal mechanistic framework. GRh2 thus represents a multifaceted therapeutic agent against PC by targeting mitochondrial integrity.

## 1 Introduction

Prostate cancer (PC) is the most common malignancy of the male genitourinary system and the second leading cause of cancer-related deaths in men globally (1). In the United States, PC is projected to account for 29% of new cancer cases and 11% of cancer deaths among men in 2024 (2). In China, its incidence has risen annually, ranking as the sixth most common cancer and seventh leading cause of cancer mortality in males in 2020 (3, 4).

Androgen deprivation therapy (ADT) serves as the first-line treatment for PC (5), yet most patients develop resistance, progressing to castration-resistant PC (CRPC)—an aggressive, therapy-insensitive subtype associated with high mortality (6). CRPC cells exhibit unique metabolic reprogramming with heightened dependence on mitochondrial function for survival (7). Targeting mitochondrial damage (e.g., inducing mitophagy or ferroptosis) has proven effective in overcoming CRPC resistance (7–9), providing a rationale for novel therapies.

Ginsenoside Rh2 (GRh2), a protopanaxadiol-type saponin distinguished by its deglycosylation at C-20, exhibits significantly higher bioavailability than other ginsenosides (e.g., Rg3, Rb1) (10, 11). Studies indicate that GRh2 suppresses tumor growth via cell cycle arrest, apoptosis, and immunomodulation (11–13). In PC, it inhibits angiogenesis and proliferation of androgen-dependent cells (14), but its potential to target mitochondrial pathways in CRPC remains unexplored.

Mitochondrial damage represents a critical therapeutic axis. In CRPC, mitochondrial dysfunction can activate autophagic degradation (mitophagy) or trigger iron-dependent death (ferroptosis) (7, 8). Emerging evidence reveals crosstalk between these processes: mitophagy may promote ferroptosis by releasing free iron (15). This dynamic interplay—poorly understood in the CRPC microenvironment—represents a promising target for combinatorial therapy.

This study demonstrates that GRh2 suppresses CRPC by orchestrating synergistic crosstalk between mitophagy and ferroptosis. Specifically, GRh2 induces mitochondrial membrane potential collapse, ROS accumulation, and ATP depletion, activating PINK1/Parkin-mediated mitophagy. Concurrently, it triggers ferroptosis via SLC7A11/GPX4 downregulation. The functional interplay of these death pathways underpins GRh2’s efficacy against CRPC resistance, highlighting a novel phytochemical strategy for mitochondrial-targeted therapy.

## 2 Materials and methods

### 2.1 Cell culture

Human prostate cancer (PC) cell lines PC3 and DU145 were obtained from the Shanghai Cell Bank, Type Culture Collection Center, Chinese Academy of Sciences. Cells were cultured in RPMI-1640 medium (HyClone, USA) supplemented with 10% fetal bovine serum (HyClone, USA) and 100 units/ml penicillin-streptomycin, and maintained in an incubator at 37°C with 5% CO2. Ginsenoside Rh2 (GRh2), mitochondrial division inhibitor 1 (Mdivi-1), and Ferrostatin-1 (Fer-1, 2 μM, purity >98%) were purchased from MedChemExpress (MCE, USA).

### 2.2 Cell viability assay

PC cells were cultured in 96-well plates, and cell viability was assessed using the CCK-8 kit (Solarbio, China). After incubation with CCK-8 reagent for 2 hours, absorbance at 450 nm was measured using a microplate reader (Infinite F50, Switzerland).

### 2.3 Plate colony formation assay

The colony formation assay reflects cell proliferation capacity and population dependency. PC3 and DU145 cells in the logarithmic growth phase were trypsinized, counted, and seeded at 1,000 cells per plate in normal medium, GRh2-containing medium, or GRh2 + Mdivi-1-containing medium. The cells were cultured in a 37°C incubator with 5% CO2 for 2–3 weeks until visible colonies formed. After incubation, cells were washed with PBS three times and fixed with 5 mL of 4% paraformaldehyde for 15 minutes. Following fixation, the paraformaldehyde was removed, and the cells were stained with Giemsa stain for 10–30 minutes. After gently washing with PBS and air drying, the plates were inverted, and images were taken to count the number of colonies. The colony formation rate was calculated as:


$$Colony\;Formation\;Rate=\left(\frac{Number\;of\;Colonies}{Number\;of\;Seeded\;Cells}\right)\times 100\%$$

### 2.4 Transwell assay

Matrigel (Thermo, USA) and RPMI-1640 (Gibco, USA) were mixed in a 1:4 ratio, and 60 μL of the mixture was added to the upper chamber of a Transwell chamber (8 μm pore size, Millipore, USA). The chamber was incubated at 37°C for 1 hour to allow Matrigel to solidify. Cells were then treated with GRh2 and GRh2 + Mdivi-1 for 48 hours. A total of 1.0 × 10^4^ cells were seeded in 100 μL of medium in the lower chamber, subjected to serum chemotaxis. After the treatment, 4% paraformaldehyde was removed and 600 μL of it was added to a new well of a 24-well plate. The Transwell chamber was then placed into this new well for 20 minutes, followed by 5 minutes in a fresh well. Cells were fixed and stained with 0.1% crystal violet. The stained membranes were examined under an inverted microscope.

### 2.5 Wound healing assay

A wound healing assay was conducted to assess the migratory ability of PC3 and DU145 cells. Cells were cultured in 6-well plates and allowed to adhere until they reached full confluence. Afterward, they were treated with normal, GRh2-containing, and GRh2 + Mdivi-1-containing complete media. A scratch was made in the cell monolayer using a 10 μL pipette tip. The cells were rinsed three times with PBS and cultured in RPMI-1640. Images were taken at pre-labeled locations immediately after the scratch and again at 24 and 48 hours. The wound area was quantified using the Image-Pro image analysis system.

### 2.6 Mitochondrial membrane potential assay

Mitochondrial membrane potential changes were assessed using JC-1 (Beyotime, China) according to the manufacturer’s instructions. PC3 and DU145 cells were seeded in a six-well plate. After aspirating the culture medium, the cells were washed once with PBS and then replenished with fresh culture medium. The JC-1 staining working solution was added and thoroughly mixed. The cells were incubated at 37°C for 20 minutes, after which the supernatant was aspirated, and the cells were washed twice with JC-1 staining buffer. Fresh culture medium was added, and the cells were observed under a fluorescence microscope (Nikon Eclipse 80i, Japan).

### 2.7 Measurement of MtROS

BBcellProbe (BestBio, China) was used to measure the mitochondrial reactive oxygen species (MtROS) content in PC3 and DU145 cells. Following the product instructions, the staining working solution was prepared by diluting the BBcellProbe Fluorescent Dye 10-fold with Dye Diluent, then further diluting it 10-fold with Buffer. The cells were cultured in appropriate dishes, and the preheated probe-containing working solution was added to the cells. After incubating for 10 minutes in the dark at 37°C, fluorescence intensity was measured using a fluorescence microscope (Nikon Eclipse 80i, Japan) with a maximum excitation/emission wavelength of 510/580 nm.

### 2.8 Measurement of ATP

The adenosine triphosphate (ATP) content in the experimental samples was measured using the ATP detection kit (Beyotime, China), following the manufacturer’s instructions. The ATP content was then normalized to the cell number.

### 2.9 Measurement of ADP/ATP Ratio

The content of adenosine diphosphate (ADP) and adenosine triphosphate (ATP) in the experimental samples was determined using the ADP/ATP Ratio Assay Kit (Abnova, Wuhan, China).

A. ATP measurement: Cells were lysed using the working solution provided in the kit. ATP reacts with the substrate D-luciferase to produce fluorescence, which is then measured to determine the ATP concentration.

B. ADP measurement: ADP is converted to ATP through an enzymatic reaction, after which the ATP concentration is measured as described in step A.

### 2.10 Transmission electron microscope

Cells from each group were collected and washed with PBS. The samples were fixed with 2.5% glutaraldehyde for 1.5 hours, followed by rinsing. Afterward, the samples were further fixed with 2% osmium tetroxide for 1.5 hours and rinsed again. The fixed cell samples were then dehydrated through a graded series of acetone. Next, the samples were infiltrated with pure acetone and embedding solution at room temperature for 1 to 1.5 hours, followed by overnight infiltration with pure embedding solution at room temperature. Ultrathin sections (70 nm) were prepared using an ultramicrotome. Double staining was performed with 2% uranyl acetate and lead citrate. Finally, the samples were observed under a transmission electron microscope.

### 2.11 Transcriptome sequencing analysis

Firstly, total RNA was extracted from the samples according to the Trizol extraction protocol, and the RNA concentration and purity were assessed using a spectrophotometer. Next, a sequencing library was constructed. DNA was then amplified into clusters, and high-throughput sequencing was performed to obtain fastq data. All data were analyzed using the Dr. Tom Multi-Organomics Data Mining System, with a cutoff of Log2FC absolute value > 1 and Q value < 0.05 to identify differentially expressed genes. Subsequently, KEGG pathway enrichment and gene ontology (GO) enrichment analyses were performed.

### 2.12 Western blot analysis

Firstly, proteins were extracted from PC3 cells, and protein concentration was determined using the BCA method. Equal amounts of protein were loaded onto a 10% polyacrylamide gel for SDS-PAGE, followed by electrophoresis, membrane transfer, blocking, and incubation with primary and secondary antibodies. Finally, protein bands were detected using ECL chemiluminescence, and images were captured. The antibodies used in this study, including PINK1, Parkin, VDAC1, TOM20, SLC7A11, GPX4, and GAPDH, were purchased from Proteintech Wuhan.

### 2.13 Animal experiment

Six-week-old immunodeficient BALB/C nude mice which were purchased from Beijing Laboratory Animal Research Centre were housed under sterile, aseptic conditions. To assess the effect of GRh2 on tumor growth, the mice were randomly assigned to three groups: the blank group, GRh2 group, and GRh2 + Mdivi-1 group. PC3 cells (1 × 10^7 cells/mL) were subcutaneously inoculated bilaterally on the medial aspect of the right upper limb-trunk junction. The cells were mixed with Matrigel (BD Biosciences, San Jose, CA, USA) and diluted 1:3 in RPMI-1640. Mice were treated according to the designated group for 21 days. Tumor size was monitored regularly with a Vernier caliper and calculated using the formula (1/2 × W × H). When the tumor volume reached 1.5 cm³, mice were euthanized by cervical dislocation. Tumor specimens were then fixed with formaldehyde and stored at -80°C for future analysis. All animal experiments were approved by the Ethics Committee of Tianjin Medical University. At the same time, we confirmed that all methods were performed in accordance with the relevant guidelines and regulations from the Ethics Committee of Tianjin Medical University.

### 2.14 Measurement of intracellular free iron levels

Fe^2+^ content in the cell samples was measured using a Ferrous Iron Colorimetric Assay Kit (Elabscience), following the manufacturer’s instructions. Absorbance was read at 593 nm using a microplate reader.

### 2.15 Lipid ROS assay

Relative lipid ROS levels in cells were assessed using C11-BODIPY dye (Thermo Fisher Scientific, D3861). Cells were incubated with 5 μM C11-BODIPY for 30 minutes, then washed three times with PBS and resuspended in 500 μL PBS. Fluorescence intensity was measured using a fluorescence microscope (Nikon Eclipse 80i, Japan) with excitation/emission wavelengths of 581/591 nm (Texas Red filter) for vat dyes and 488/510 nm (FITC filter) for oxidation dyes.

### 2.16 Measurement of MDA and GSH level

PC cells were seeded in cell culture dishes and exposed to the designated treatment groups. After incubation, cells were counted, and an equal number of cells were collected for analysis. Malondialdehyde (MDA) levels were measured using a lipid peroxide malondialdehyde assay kit (Beyotime). Glutathione (GSH) levels were determined according to the manufacturer’s instructions (kktb1600, Abbkine).

### 2.17 Statistical analyses

Statistical analysis was conducted using GraphPad Prism 8 software (GraphPad, USA). The specific statistical tests used were chosen based on data type and experimental design. Comparisons between two groups were analyzed using a two-tailed Student’s t-test. Comparisons across multiple groups were analyzed using one-way analysis of variance (ANOVA) followed by Tukey’s *post hoc* test for pairwise comparisons. Cell proliferation curves were analyzed using two-way ANOVA followed by Sidak’s *post hoc* test for comparisons at specific time points. All data are presented as the mean ± standard error of the mean (SEM). A p-value of < 0.05 was considered statistically significant.

## 3 Results

### 3.1 GRh2 inhibited viability, proliferation, migration and invasion of prostate cancer cells

**Figure 1:**
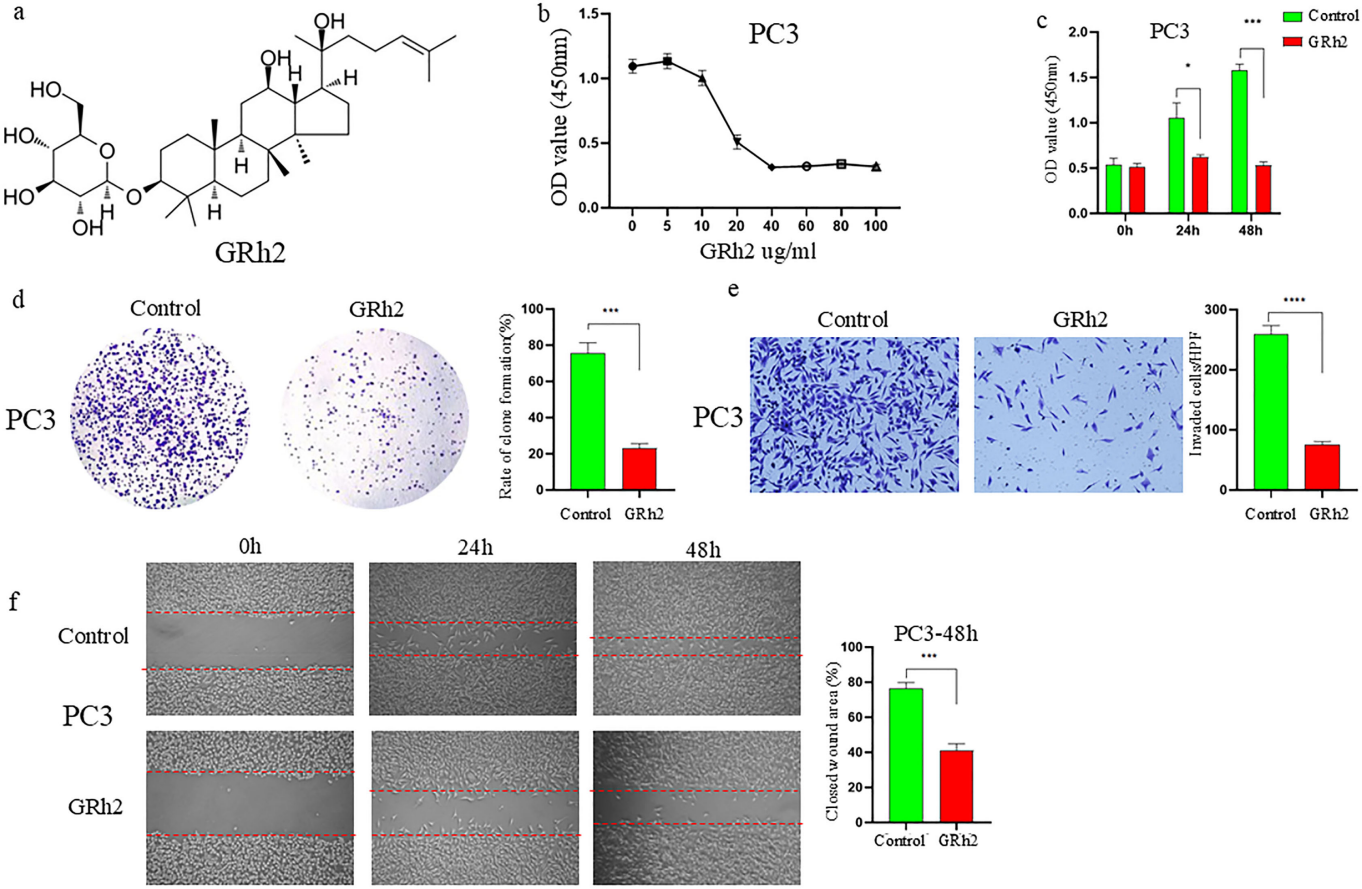
GRh2 inhibited viability, proliferation, migration and invasion of PC3 cells were treated with GRh2. **(a)** The molecular structure of GRh2. **(b)** The cell viability of PC3 treated by GRh2 were measured using CCK8 assays. **(c)** The ability of cell proliferation was significantly reduced after exposing to GRh2 by CCK8 assays. **(d)** PC3 cells were treated with GRh2, and colony formation was assessed by staining with crystal violet. **(e)** PC3 cells were exposed to GRh2, the invasive capability was evaluated by transwell assay. **(f)** PC3 cells were exposed to GRh2, migration capacity was evaluated by wound healing assay. (Data are shown as mean ± SEM; n = 3 independent experiments (biological replicates: independent cell culture batches). Statistical significance: n.s, not significant; *P < 0.05; **P < 0.01; ***P < 0.001; ****P < 0.0001; determined by one-way ANOVA with Tukey’s *post hoc* test **(b, d, e, f)** and two-way ANOVA with Sidak’s *post hoc* test **(c)**.

To investigate the inhibitory effect of GRh2 (**Figure 1a**) on the growth of prostate cancer cells, PC3 cells were exposed to eight different concentrations of GRh2 (0 μg/ml, 5 μg/ml, 10 μg/ml, 20 μg/ml, 40 μg/ml, 60 μg/ml, 80 μg/ml, and 100 μg/ml) for 48 hours, and cellular viability was assessed by CCK-8 assay. As shown in **Figure 1b**, the viability of PC3 cells gradually decreased with increasing GRh2 concentrations, with the half-maximal inhibitory concentration (IC50) calculated to be 19.3 μg/ml. To further verify the effect of GRh2 at the IC50 concentration, we evaluated its impact on cell proliferation at three time points: 0h, 24h, and 48h. The results revealed a significant reduction in cell proliferation following GRh2 exposure (**Figure 1c**). Plate colony formation assays also indicated a diminished cloning capability of PC3 cells upon GRh2 treatment (**Figure 1d**).

Subsequently, we assessed the effects of GRh2 on the invasion and migration capacities of PC3 cells. Transwell assays showed a marked reduction in the number of invaded PC3 cells after GRh2 treatment (**Figure 1e**). Additionally, wound healing assays demonstrated a significant decrease in migration in the GRh2-treated group compared to the control group (**Figure 1f**). Similar results were observed in DU145 cells (**Supplementary Figure S1**).

These findings collectively suggest that GRh2 effectively inhibits the viability, proliferation, migration, and invasion of prostate cancer cells.

### 3.2 GRh2 driven mitochondrial damage in PC cells

**Figure 2:**
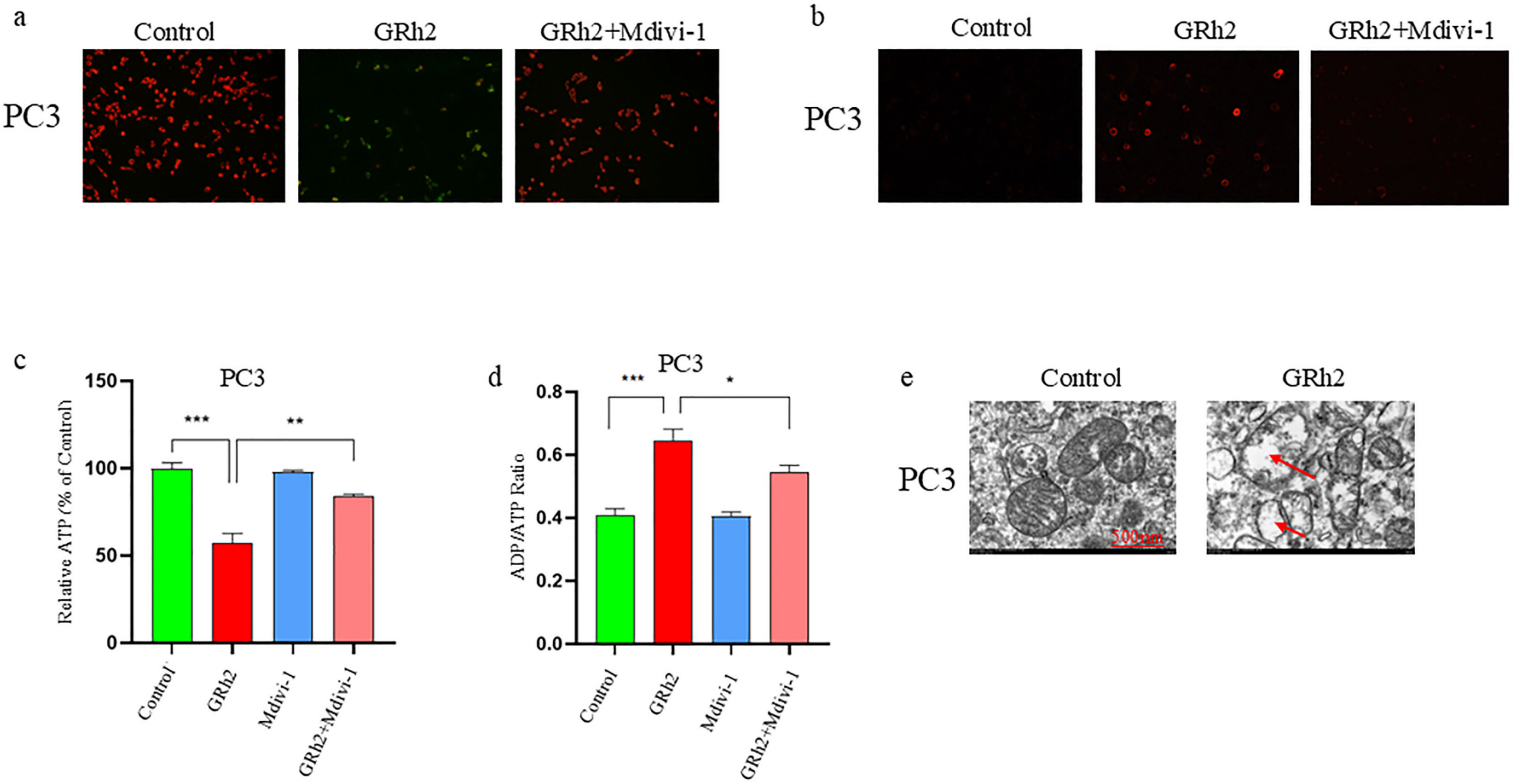
GRh2 potentiated mitochondria damage in PC3 cells. **(a)** PC3 cells were cultured in different group (Control, GRh2, and GRh2+Mdivi-1) for 48 h, and mitochondrial membrane potential was detected using the JC-1 probe. **(b)** PC3 cells were treated in different group for 48 h (Control, GRh2, and GRh2+Mdivi-1), then the levels of mitochondrial ROS were observed by fluorescence microscope. **(c)** PC3 cells were treated for 48 h in different group (Control, GRh2, Mdivi-1, and GRh2+Mdivi-1), the intracellular ATP level was determined using an ATP detection assay kit. **(d)** PC3 cells were treated for 48 h in different group (Control, GRh2, Mdivi-1, and GRh2+Mdivi-1), the ADP/ATP ratio was determined using the ADP/ATP ratio assay kit. **(e)** Representative transmission electron microscopy (TEM) images of PC3 cells exposed to GRh2 for 48 **(h)** Arrows highlight mitochondria exhibiting damage, characterized by swelling and loss of cristae. Scale bar, 500nm. Magnification: 500,000 times. (Data in a-d are shown as mean ± SEM; n = 3 independent experiments (biological replicates: independent cell culture batches). Statistical significance: n.s, not significant; *P < 0.05; **P < 0.01; ***P < 0.001; determined by one-way ANOVA with Tukey’s *post hoc* test).

Mitochondria are the powerhouses of cells, and mitochondrial damage plays a critical role in regulating cell death. To explore whether GRh2 induces mitochondrial damage leading to cell death, we first examined the mitochondrial membrane potential in PC3 cells. As shown in **Figure 2a**, JC-1 staining revealed a significant reduction in mitochondrial membrane potential following GRh2 treatment. However, the mitochondrial membrane potential was restored by the addition of Mdivi-1, a mitochondrial division inhibitor.

Mitochondrial ROS is a key indicator of mitochondrial dysfunction, so we assessed the production of mitochondrial ROS using the BBcell probe. Fluorescence microscopy results showed that GRh2 markedly increased mitochondrial ROS levels in PC3 cells. This increase was reversed by the addition of Mdivi-1, confirming the role of mitochondrial damage in GRh2-induced effects (**Figure 2b**).

We also measured ATP levels and the ADP/ATP ratio, both of which were significantly altered in GRh2-treated cells compared to controls (**Figures 2c**, **2d**). To further validate these findings, we performed transmission electron microscopy (TEM). As depicted in **Figure 2e**, GRh2 treatment induced mitochondrial swelling, an increased density of bilayer membranes, and a reduction or complete disappearance of mitochondrial cristae in PC3 cells. These observations collectively indicate that GRh2 induces mitochondrial damage, leading to cellular dysfunction and death.

Similar results were obtained in DU145 cells (**Supplementary Figure S2**), supporting the consistency of GRh2’s effect on mitochondrial impairment across different prostate cancer cell lines.

### 3.3 GRh2 resulted in mitophagy in PC cells

**Figure 3:**
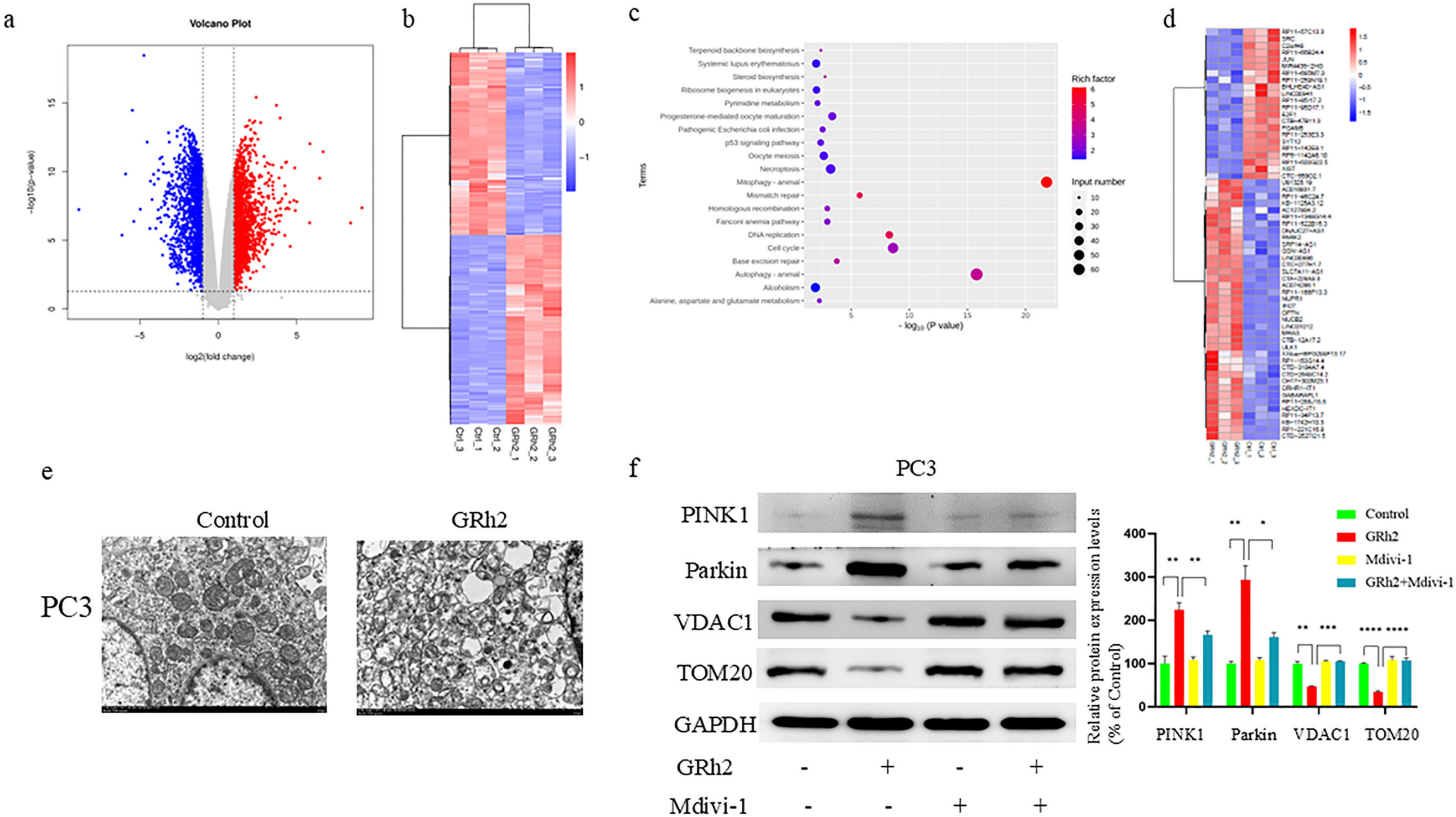
GRh2 driven mitophagy in PC cells. **(a–d)** RNA-seq analysis reveals the DEGs in PC3 cells from two groups: control (n = 3 per group) and GRh2 (n = 3 per group). **(a)** Volcano map showed genes with decreased and increased expression levels. **(b)** Heat map representing the significantly regulated genes detected in RNA-seq analysis. **(c)** KEGG pathway enrichment analysis in GRh2-treated PC3 cells compared with control PC3 cells. **(d)** Heat map representing the mitophagy- related genes detected in RNA-seq analysis of PC3 cells. **(e)** Electron microscopy of mitochondria in PC3 cells. **(f)** Protein expression of PINK1, Parkin, VDAC1, and TOM20 were measured by WB, blots were used by clear delineation with dividing lines. (Data in f are shown as mean ± SEM; n = 3 independent experiments; Statistical significance determined by one-way ANOVA with Tukey’s *post hoc* test; *P < 0.05; **P < 0.01; ***P < 0.001; ****P < 0.0001).

To investigate the molecular mechanisms underlying GRh2’s repressive effects on prostate cancer (PC), RNA sequencing was conducted on PC3 cells from two groups: control and GRh2-treated. The differentially expressed genes (DEGs) between the two groups are shown in **Figures 3a**, **3b**. Kyoto Encyclopedia of Genes and Genomes (KEGG) pathway analysis revealed that the mitophagy pathway was the top enriched pathway among the DEGs in the GRh2-treated group compared to the control group (**Figure 3c**).

Further analysis of the DEGs associated with mitophagy (**Figure 3d**) confirmed the involvement of this pathway in GRh2’s effects on PC3 cells. Based on our previous observations of mitochondrial damage following GRh2 treatment, we sought to validate these findings by assessing mitophagy in PC cells. Transmission electron microscopy (TEM) revealed that GRh2 treatment induced mitochondrial damage, with damaged mitochondria surrounded by membranous structures forming autophagosomes (**Figure 3e**).

Additionally, the expression levels of key mitophagy markers, including PINK1, Parkin, VDAC1, and TOM20, were analyzed by western blotting. Our results showed that PINK1 and Parkin were significantly upregulated, whereas VDAC1 and TOM20 were downregulated in the GRh2-treated group compared to the control group (**Figure 3f**). These changes were reversed by the addition of Mdivi-1, further supporting the role of mitochondrial damage in GRh2-induced mitophagy.

Taken together, these findings suggest that GRh2 induces mitophagy in PC cells, contributing to its anti-cancer effects.

### 3.4 GRh2 repressed the progression of prostate cancer cells through mitophagy

**Figure 4:**
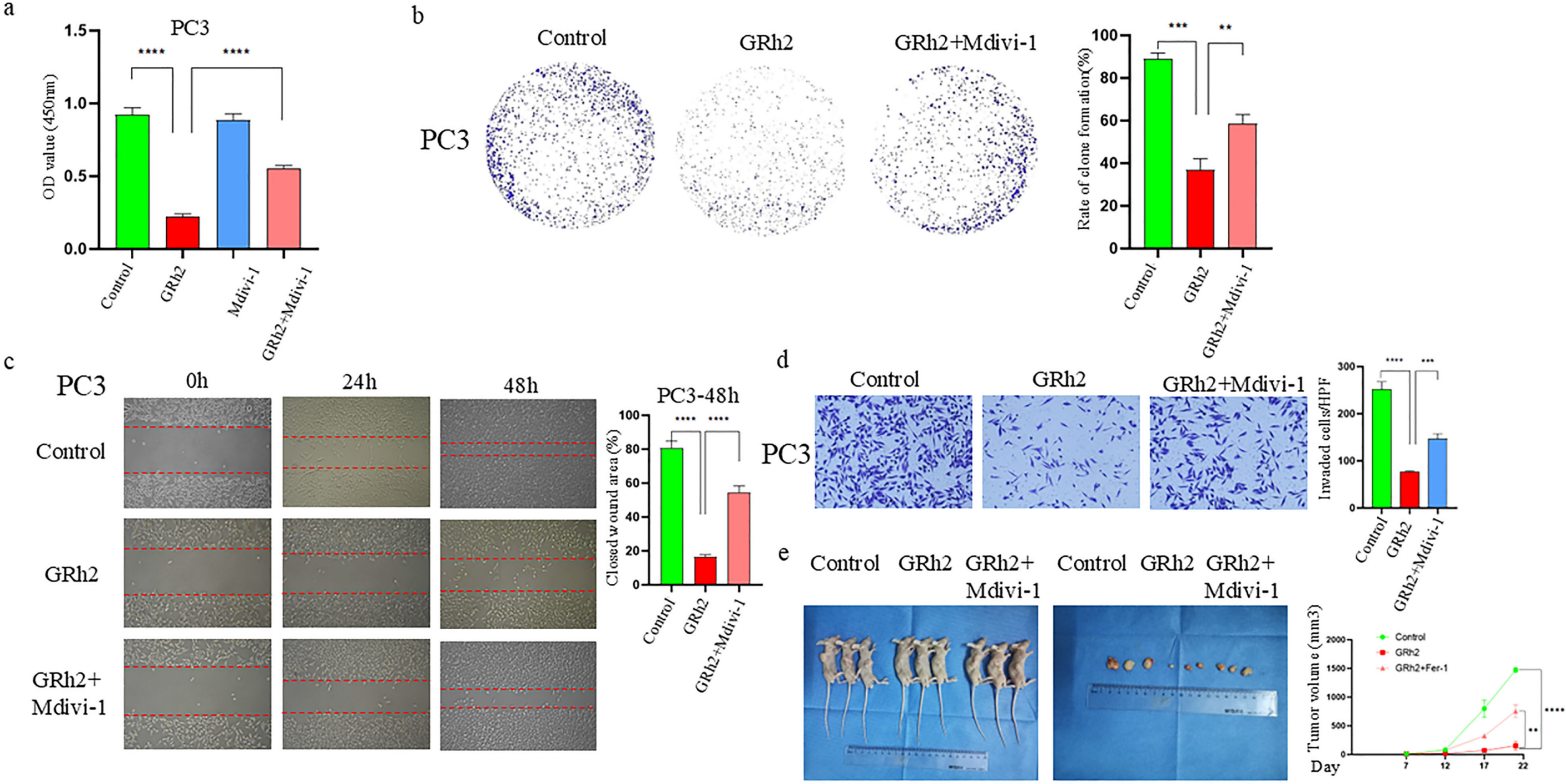
GRh2 repressed the progression of prostate cancer cells through mitophagy. **(a)** The cell viability of PC3 treated by GRh2 and Mdivi-1 were measured using CCK8 assays **(b)** PC3 cells were treated with GRh2 and Mdivi-1, and colony formation was assessed by staining with crystal violet. **(c)** PC3 cells were exposed to GRh2 and Mdivi-1, migration capacity was evaluated by wound healing assay. **(d)** PC3 cells were exposed to GRh2 and Mdivi-1, the invasive capability was evaluated by Transwell assay. **(e)** *In vivo* experiments verified the effect of GRh2 exposure on xenograft tumors in nude mice, and compared the changes in volume of xenograft tumors. (Data in a-d are shown as mean ± SEM; n = 3 independent experiments (biological replicates: independent cell culture batches). Data in e are shown as mean ± SEM; n = 3 mice per group. Statistical significance: n.s, not significant; *P < 0.05; **P < 0.01; ***P < 0.001; ****P < 0.0001; determined by one-way ANOVA with Tukey’s *post hoc* test).

To further investigate the effect of GRh2 on the progression of prostate cancer cells via mitophagy, we performed a series of experimental verifications. Firstly, we treated PC3 cells with four different groups (control, GRh2, Mdivi-1, and GRh2 + Mdivi-1) for 48 hours and assessed cell viability using the CCK8 assay. As shown in **Figure 4a**, GRh2 exposure led to a decrease in cell viability, which was restored by the addition of Mdivi-1. Additionally, colony formation assays revealed an increase in the clonogenic capacity of PC3 cells when treated with GRh2 + Mdivi-1 compared to the GRh2-only group (**Figure 4b**).

Next, we examined the effect of GRh2 with and without Mdivi-1 on the invasion and migration capacity of PC3 cells. Wound healing assays (**Figure 4c**) showed that cell migration was significantly increased in the GRh2 + Mdivi-1 group compared to the GRh2-only group. Similarly, Transwell assays indicated that the number of invaded PC3 cells was higher in the GRh2 + Mdivi-1 group than in the GRh2 group (**Figure 4d**).

To further explore whether GRh2 affects prostate cancer progression *in vivo*, we established a subcutaneous PC model in BALB/c nude mice using PC3 cells. The mice were randomly assigned to the control group, GRh2 group, and GRh2 + Mdivi-1 group. The results showed that tumor growth was significantly suppressed in the GRh2 group compared to the control group, with tumor volume in the GRh2 group being approximately five times smaller than that in the control group. However, after the addition of Mdivi-1, both tumor growth rate and volume increased compared to the GRh2-only group (**Figure 4e**).

In summary, our results indicate that GRh2 inhibits the progression of prostate cancer cells through the induction of mitophagy.

### 3.5 GRh2 exposure promoted ferroptosis in prostate cancer cells by inhibiting the SLC7A11/GPX4 axis

In our previous study, we observed that GRh2 exposure to prostate cancer cells resulted in mitochondrial damage, as well as changes in mitochondrial morphology and structure. Based on these findings, we hypothesized a potential link between GRh2 exposure and ferroptosis induction in PC cells, and conducted the following studies to explore this connection.

**Figure 5:**
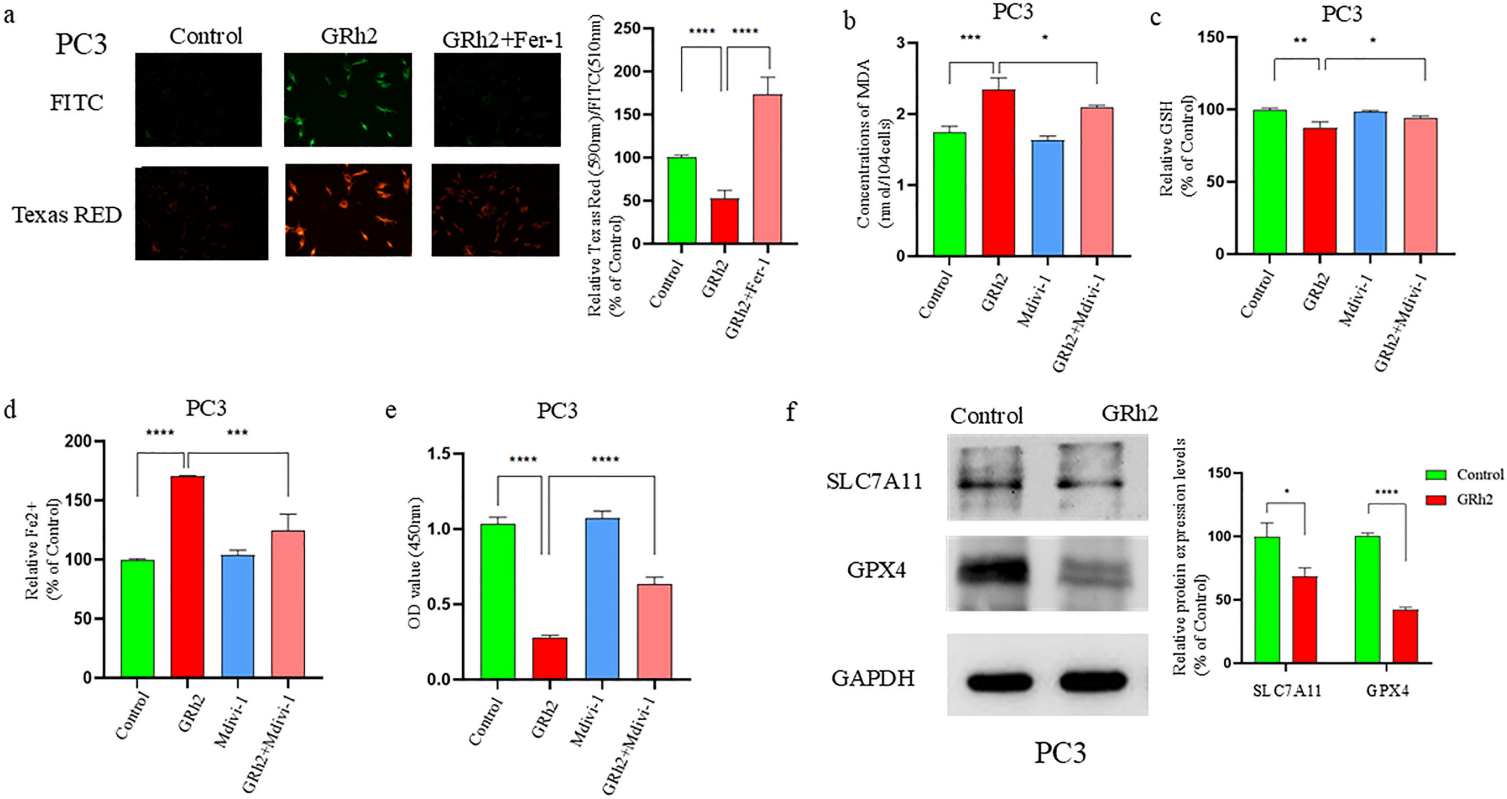
GRh2 can inhibit viability of prostate cancer cells by promoting SLC7A11/GPX4 pathway-mediated ferroptosis. **(a)** C11 BODIPY 488/561 probe detected and quantified the effect on lipid reactive oxygen species (ROS) in GRh2-exposed PC3 cells. **(b)** GRh2 exposure can promote the increase of Malondialdehyde (MDA) in PC3 cells. **(c)** Effect on Glutathione (GSH) in PC3 cells exposed to GRh2. **(d)** GRh2 exposure can promote the increase of ferrous iron (Fe^2+^) in PC3 cells. **(e)** The cell viability of PC3 treated by GRh2 and Ferrostatin-1 (Fer-1) were measured using Cell Counting Kit-8 (CCK-8) assays. **(f)** The expression of solute carrier family 7member 11 (SLC7A11) and glutathione peroxidase 4 (GPX4) protein between control group and GRh2 group was detected by Western blotting (WB), blots were used by clear delineation with dividing lines. (Data in a-e are shown as mean ± SEM; n = 3 independent experiments (biological replicates: independent cell culture batches). Statistical significance: n.s, not significant; *P < 0.05; **P < 0.01; ***P < 0.001; ****P < 0.0001; determined by one-way ANOVA with Tukey’s *post hoc* test).

To investigate whether GRh2 exposure promotes ferroptosis, we treated PC3 cells with GRh2, GRh2 + Fer-1 (ferroptosis inhibitors), and control groups. Lipid ROS, a key marker of ferroptosis, was measured, and our results showed that the green fluorescence intensity in the GRh2-exposed group was significantly stronger than in the other groups, indicating a marked increase in intracellular lipid ROS levels (**Figure 5a**).

We further assessed several ferroptosis-related markers, including Fe2+, GSH, and MDA levels. The GRh2-exposed group showed significantly higher Fe2+ and MDA levels, while GSH levels were significantly reduced compared to the Fer-1 and control groups (**Figures 5b–d**). To further validate these findings, we performed a CCK8 assay on PC3 cells treated with GRh2, Fer-1, or GRh2 + Fer-1 for 48 hours. The results (**Figure 5e**) indicated that GRh2 exposure decreased cell viability, but this effect was reversed by the addition of Fer-1.

GPX4, a key GSH peroxidase, plays an essential role in ferroptosis by inhibiting lipid peroxidation (16). We observed that GRh2 exposure decreased intracellular GSH levels, suggesting a potential link to GPX4 inhibition. Additionally, SLC7A11, an upstream regulator of GPX4, is known to promote ferroptosis when downregulated (17). We examined the expression levels of GPX4 and SLC7A11 by Western blotting and found that their expression was significantly reduced in the GRh2-exposed group compared to the control (**Figure 5f**).

In summary, our findings suggest that GRh2 exposure induces ferroptosis in prostate cancer cells by repressing the SLC7A11/GPX4 pathway, leading to decreased cell viability.

## 4 Discussion

Prostate cancer (PC) remains a global health challenge, driven by an aging population and the limitations of current therapies like endocrine therapy and chemotherapy (2, 18). The emergence of drug resistance underscores the urgent need for novel mechanisms-based strategies (19). Mitochondrial damage has gained traction as a promising therapeutic target in oncology due to its pivotal role in cell survival, death, and metabolism (20, 21). Our study demonstrates that Ginsenoside Rh2 (GRh2) exerts potent anti-tumor effects against prostate cancer *in vitro* and *in vivo* primarily through the induction of mitochondrial damage, subsequently activating two key processes: PINK1/Parkin-mediated mitophagy and ferroptosis.

Critically, our findings extend beyond simply documenting GRh2-induced mitochondrial damage (evidenced by MMP depolarization, mtROS surge, ATP depletion, and ultrastructural changes like cristae loss). The functional significance lies in how this damage mechanistically underpins the observed suppression of proliferation, migration, and invasion. Depletion of ATP directly cripples the bioenergetic demands essential for cell division and cytoskeletal remodeling required for motility. Furthermore, the surge in mtROS acts as a dual-edged sword: it disrupts redox-sensitive signaling pathways critical for cell cycle progression and metastatic behavior, while simultaneously serving as a trigger for mitophagy and ferroptosis – processes inherently antagonistic to tumor growth and spread (22, 23). This positions GRh2-induced mitochondrial dysfunction as a central driver of its multifaceted anti-cancer phenotype.

Our transcriptomic and biochemical analyses revealed that GRh2 robustly activates PINK1/Parkin-mediated mitophagy, a finding supported by TEM visualization of mitochondria engulfed within autophagosome structures. The functional dependency of GRh2’s effects on mitophagy was unequivocally demonstrated by the reversal of its anti-proliferative, anti-migratory, and anti-invasive actions upon co-treatment with specific mitophagy inhibitors. This dependency underscores a crucial mechanistic insight: GRh2 leverages the cell’s own quality control system (mitophagy) to eliminate damaged mitochondria (24), thereby amplifying the initial insult into a sustained anti-tumor response. Blocking mitophagy rescues the energy deficit and mitigates ROS-mediated signaling disruption, allowing cancer cells to partially recover their malignant potential. This aligns with the established role of mitophagy in maintaining cellular fitness but highlights its potential as a vulnerability when exploited therapeutically in cancer contexts.

Simultaneously, we provide compelling evidence that GRh2 triggers ferroptosis in PC cells. The observed mitochondrial pathology (swelling, cristae reduction) is a recognized hallmark of ferroptosis. Crucially, the ferroptosis inhibitor Ferrostatin-1 (Fer-1) significantly rescued GRh2-induced cell death. GRh2 treatment induced classical ferroptosis features (25): accumulation of lipid ROS and MDA, depletion of GSH, elevation of intracellular free iron, and downregulation of key anti-ferroptosis proteins GPX4 and SLC7A11. This induction of ferroptosis represents a significant complementary mechanism to mitophagy. While mitophagy removes damaged organelles, ferroptosis delivers a lethal blow through catastrophic lipid peroxidation, directly compromising membrane integrity and function – a death mechanism fundamentally incompatible with cell viability.

Our study places GRh2 within a growing class of natural compounds targeting mitochondrial integrity for cancer therapy, but its dual activation of mitophagy and ferroptosis is particularly noteworthy. While mitophagy is generally cytoprotective, its hyperactivation under conditions of severe mitochondrial stress, as induced by GRh2, can become detrimental. Furthermore, the link between mitochondrial damage and sensitization to ferroptosis is an area of intense research. Our data suggest GRh2 exploits this vulnerability, potentially offering an advantage over therapies targeting single death pathways, as cancer cells may have redundant escape mechanisms for apoptosis or necrosis. The observed downregulation of mitochondrial membrane proteins (TOM20, VDAC1) likely reflects both mitophagy degradation and broader mitochondrial disruption.

However, several important questions and limitations warrant consideration. Firstly, while we demonstrate the involvement of both mitophagy and ferroptosis, the precise molecular crosstalk or sequence of events linking GRh2-induced mitochondrial damage to the activation of these distinct pathways remains unclear. Does mitophagy flux precede ferroptosis, or do they occur concurrently? Secondly, our study primarily utilized the PC3 cell line (androgen-independent); confirming these mechanisms in androgen-dependent models (like DU145) and diverse PC subtypes would strengthen the generalizability of our findings. Finally, the *in vivo* evidence, while supportive, focused on tumor growth suppression. Future studies should directly assess markers of mitophagy and ferroptosis within the tumor microenvironment of the xenograft models.

**Figure 6:**
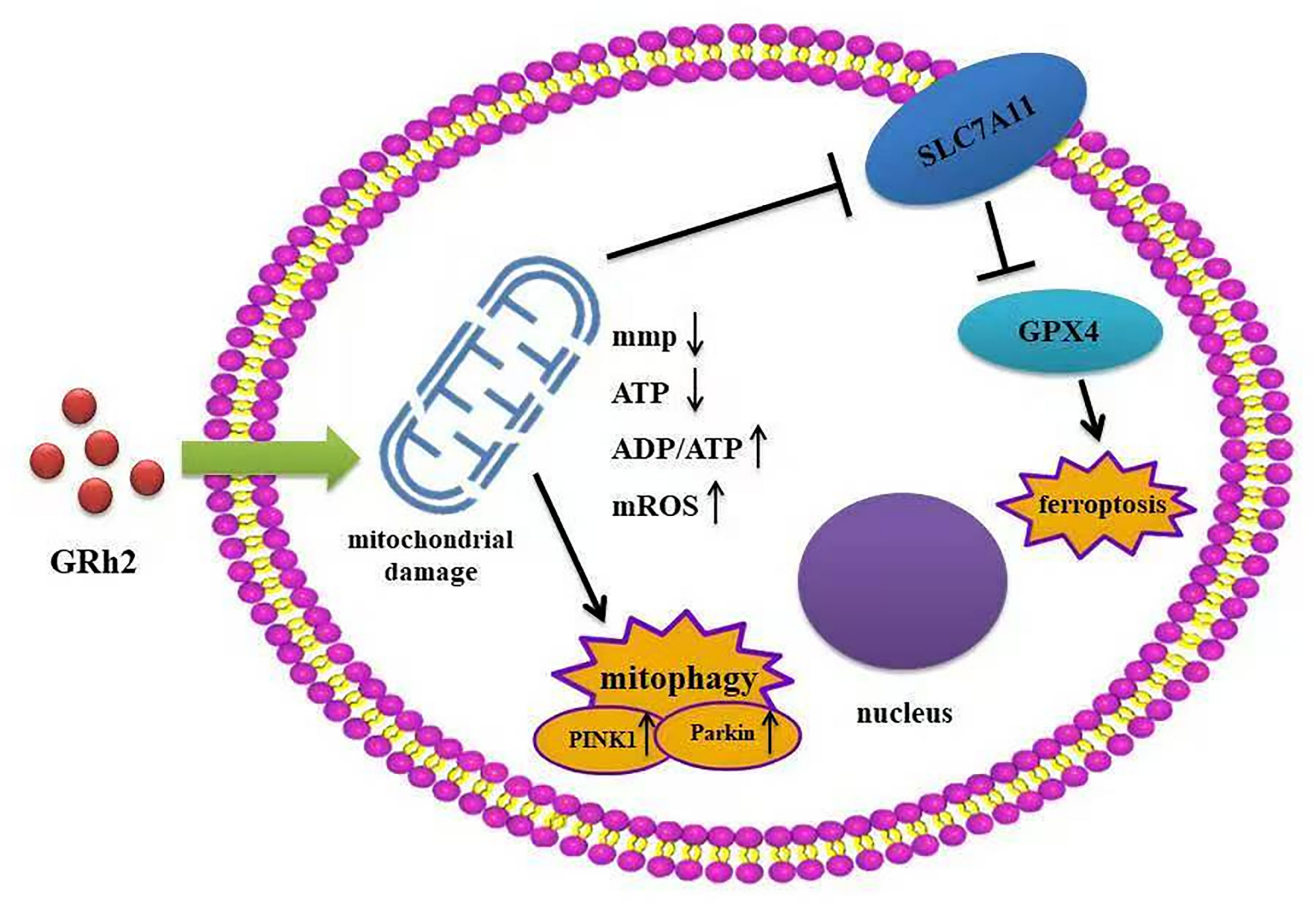
GRh2 induces mitochondrial damage via mitophagy and ferroptosis.

In conclusion, our study elucidates that GRh2 targets prostate cancer by inducing mitochondrial damage, which subsequently activates two critical pathways: PINK1/Parkin-mediated mitophagy and ferroptosis (**Figure 6**). This dual mechanism provides a robust foundation for its anti-tumor efficacy, disrupting energy metabolism, survival signals, and membrane integrity. While the interplay between mitophagy and ferroptosis requires further dissection, our findings position GRh2 as a promising multi-mechanistic agent and underscore the therapeutic potential of exploiting mitochondrial vulnerabilities in prostate cancer. This work contributes to the expanding field of mitochondrial medicine in oncology and highlights GRh2 as a candidate worthy of further preclinical development.

## Data Availability

All relevant data is contained within the article. The original contributions presented in the study are included in the article/**Supplementary Material**, further inquiries can be directed to the corresponding authors.

## Supplementary material

Supplementary Figure 1 GRh2 inhibited viability, proliferation, migration and invasion of DU145 cells were treated with GRh2. **(a)** The cell viability of DU145 treated by GRh2 were measured using CCK8 assays **(b)** The ability of cell proliferation was significantly reduced after exposing to GRh2 by CCK8 assays **(c)** DU145 cells were treated with GRh2, and colony formation was assessed by staining with crystal violet. **(d)** DU145 cells were exposed to GRh2, migration capacity was evaluated by wound healing assay. **(e)** DU145 cells were exposed to GRh2, the invasive capability was evaluated by transwell assay. (Data are shown as mean ± SEM; n = 3 independent experiments (biological replicates: independent cell culture batches). Statistical significance: n.s, not significant; *, P < 0.05; **, P < 0.01; ***, P < 0.001; ****, P < 0.0001; determined by one-way ANOVA with Tukey’s *post hoc* test **(a, c–e)** and two-way ANOVA with Sidak’s *post hoc* test **(b)**).

Supplementary Figure 2 GRh2 potentiated mitochondria damage in DU145 cells. **(a)** DU145 cells were cultured in different group (Control, GRh2, and GRh2+Mdivi-1) for 48 h, and mitochondrial membrane potential was detected using the JC-1 probe. **(b)** DU145 cells were treated in different group for 48 h (Control, GRh2, and GRh2+Mdivi-1), then the levels of mitochondrial ROS were observed by fluorescence microscope. **(c)** DU145 cells were treated for 48 h in different group (Control, GRh2, Mdivi-1, and GRh2+Mdivi-1), the intracellular ATP level was determined using an ATP detection assay kit. **(d)** DU145 cells were treated for 48 h in different group (Control, GRh2, Mdivi-1, and GRh2+Mdivi-1), the ADP/ATP ratio was determined using the ADP/ATP ratio assay kit. **(e)** Representative transmission electron microscopy (TEM) images of DU145 cells exposed to GRh2 for 48 h. Arrows highlight mitochondria exhibiting damage, characterized by swelling and loss of cristae. Scale bar, 500nm. Magnification: 500,000 times. (Data in a-d are shown as mean ± SEM; n = 3 independent experiments (biological replicates: independent cell culture batches). Statistical significance: n.s, not significant; *, P < 0.05; **, P < 0.01; ***, P < 0.001; determined by one-way ANOVA with Tukey’s *post hoc* test).
