# Supplementary material for: Ginsenoside Rh2 repressed the progression of prostate cancer through the mitochondrial damage induced by mitophagy and ferroptosis
Source: Front Oncol. 2025 Aug 21;15:1633891. doi: 10.3389/fonc.2025.1633891 (PMC12408308; doi:10.3389/fonc.2025.1633891)
Supplement: Supplementary Figure 1 — GRh2 inhibited viability, proliferation, migration and invasion of DU145 cells were treated with GRh2. (a) The cell viability of DU145 treated by GRh2 were measured using CCK8 assays (b) The ability of cell proliferation was significantly reduced after exposing to GRh2 by CCK8 assays (c) DU145 cells were treated with GRh2, and colony formation was assessed by staining with crystal violet. (d) DU145 cells were exposed to GRh2, migration capacity was evaluated by wound healing assay. (e) DU145 cells were exposed to GRh2, the invasive capability was evaluated by transwell assay. (Data are shown as mean ± SEM; n = 3 independent experiments (biological replicates: independent cell culture batches). Statistical significance: n.s, not significant; *, P < 0.05; **, P < 0.01; ***, P < 0.001; ****, P < 0.0001; determined by one-way ANOVA with Tukey’s post hoc test (a, c–e) and two-way ANOVA with Sidak’s post hoc test (b)). [file Image1.pdf]

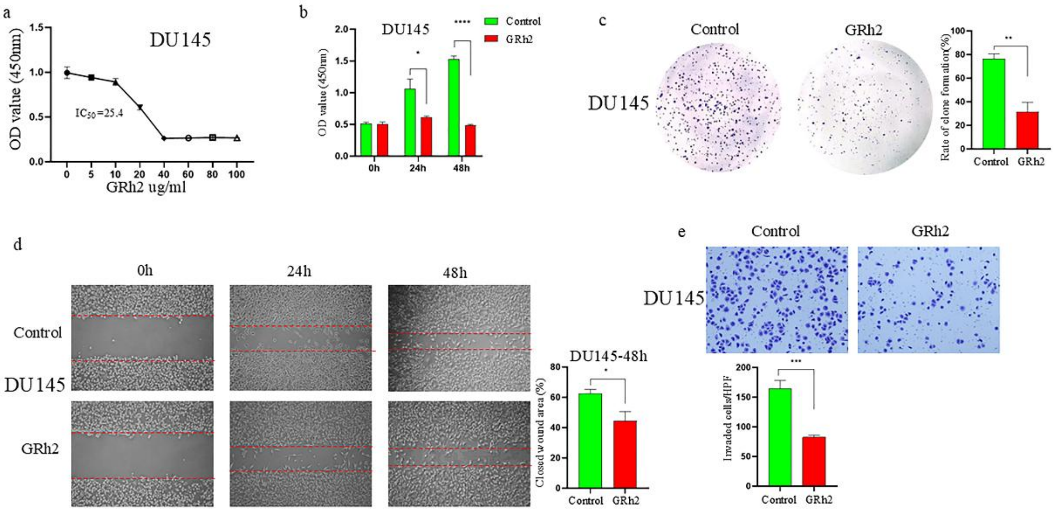

Fig. 1S. GRh2 inhibited viability, proliferation, migration and invasion of DU145 cells were treated with GRh2. (a) The cell viability of DU145 treated by GRh2 were measured using CCK8 assays (b) The ability of cell proliferation was significantly reduced after exposing to GRh2 by CCK8 assays (c) DU145 cells were treated with GRh2, and colony formation was assessed by staining with crystal violet. (d) DU145 cells were exposed to GRh2, migration capacity was evaluated by wound healing assay. (e) DU145 cells were exposed to GRh2, the invasive capability was evaluated by transwell assay.. (Data are shown as mean ± SEM; n = 3 independent experiments (biological replicates: independent cell culture batches). Statistical significance: n.s, not significant; *, $P < 0.05$; **, $P < 0.01$; ***, $P < 0.001$; ****, $P < 0.0001$; determined by one-way ANOVA with Tukey's post hoc test (a, c, d, e) and two-way ANOVA with Sidak's post hoc test (b)).
