# Supplementary material for: Ginsenoside Rh2 repressed the progression of prostate cancer through the mitochondrial damage induced by mitophagy and ferroptosis
Source: Front Oncol. 2025 Aug 21;15:1633891. doi: 10.3389/fonc.2025.1633891 (PMC12408308; doi:10.3389/fonc.2025.1633891)
Supplement: Supplementary Figure 2 — GRh2 potentiated mitochondria damage in DU145 cells. (a) DU145 cells were cultured in different group (Control, GRh2, and GRh2+Mdivi-1) for 48 h, and mitochondrial membrane potential was detected using the JC-1 probe. (b) DU145 cells were treated in different group for 48 h (Control, GRh2, and GRh2+Mdivi-1), then the levels of mitochondrial ROS were observed by fluorescence microscope. (c) DU145 cells were treated for 48 h in different group (Control, GRh2, Mdivi-1, and GRh2+Mdivi-1), the intracellular ATP level was determined using an ATP detection assay kit. (d) DU145 cells were treated for 48 h in different group (Control, GRh2, Mdivi-1, and GRh2+Mdivi-1), the ADP/ATP ratio was determined using the ADP/ATP ratio assay kit. (e) Representative transmission electron microscopy (TEM) images of DU145 cells exposed to GRh2 for 48 h. Arrows highlight mitochondria exhibiting damage, characterized by swelling and loss of cristae. Scale bar, 500nm. Magnification: 500,000 times. (Data in a-d are shown as mean ± SEM; n = 3 independent experiments (biological replicates: independent cell culture batches). Statistical significance: n.s, not significant; *, P < 0.05; **, P < 0.01; ***, P < 0.001; determined by one-way ANOVA with Tukey’s post hoc test). [file Image2.pdf]

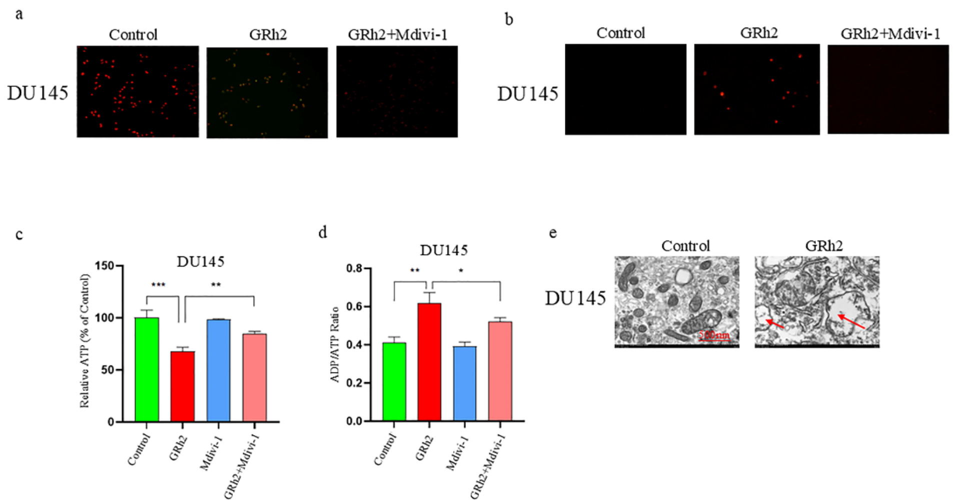

Fig. 2S. GRh2 potentiated mitochondria damage in DU145 cells. (a) DU145 cells were cultured in different group (Control, GRh2, and GRh2+Mdivi-1) for 48 h, and mitochondrial membrane potential was detected using the JC-1 probe. (b) DU145 cells were treated in different group for 48 h (Control, GRh2, and GRh2+Mdivi-1), then the levels of mitochondrial ROS were observed by fluorescence microscope. (c) DU145 cells were treated for 48 h in different group (Control, GRh2, Mdivi-1, and GRh2+Mdivi-1), the intracellular ATP level was determined using an ATP detection assay kit. (d) DU145 cells were treated for 48 h in different group (Control, GRh2, Mdivi-1, and GRh2+Mdivi-1), the ADP/ATP ratio was determined using the ADP/ATP ratio assay kit. (e) Representative transmission electron microscopy (TEM) images of DU145 cells exposed to GRh2 for 48 h. Arrows highlight mitochondria exhibiting damage, characterized by swelling and loss of cristae. Scale bar, 500nm. Magnification: 500,000 times. (Data in a-d are shown as mean ± SEM; n = 3 independent experiments (biological replicates: independent cell culture batches). Statistical significance: n.s, not significant; *, $P < 0.05$; **, $P < 0.01$; ***, $P < 0.001$; determined by one-way ANOVA with Tukey's post hoc test).
