# Supplementary figures and images for: Ginsenoside Rh2 repressed the progression of prostate cancer through the mitochondrial damage induced by mitophagy and ferroptosis (part 1 of 2)
Source: Front Oncol. 2025 Aug 21;15:1633891. doi: 10.3389/fonc.2025.1633891 (PMC12408308; doi:10.3389/fonc.2025.1633891)

Fig.5

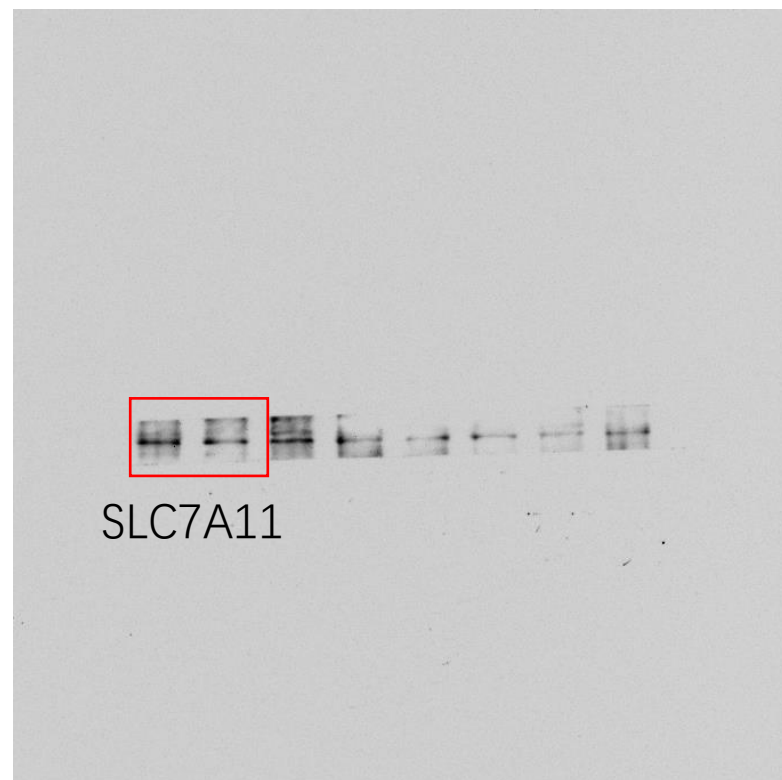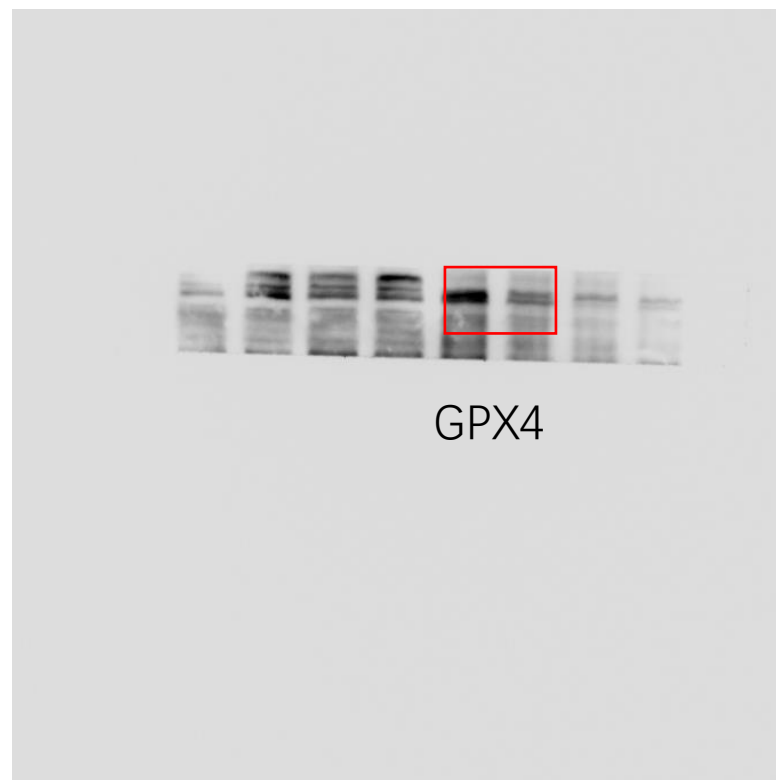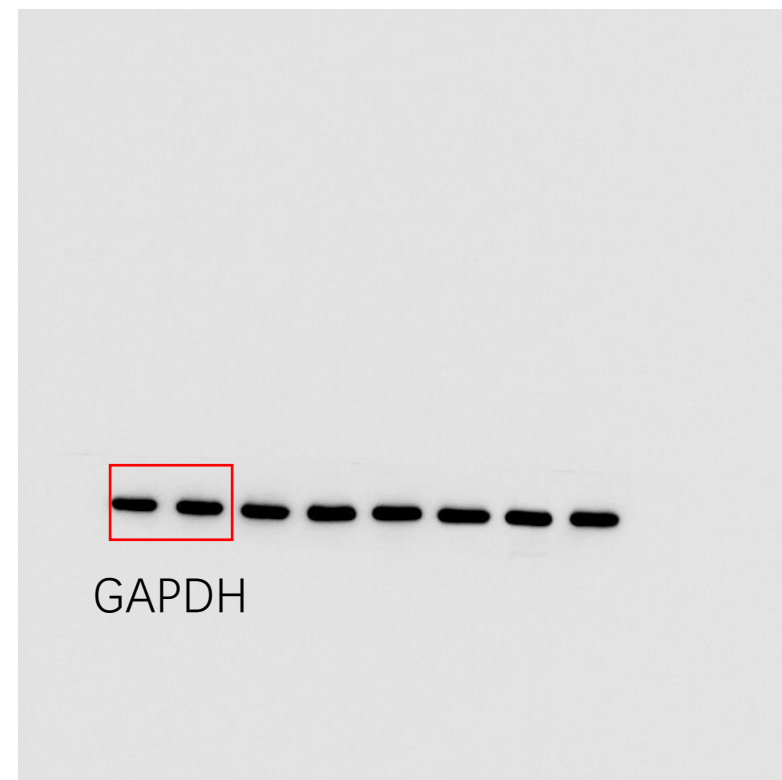

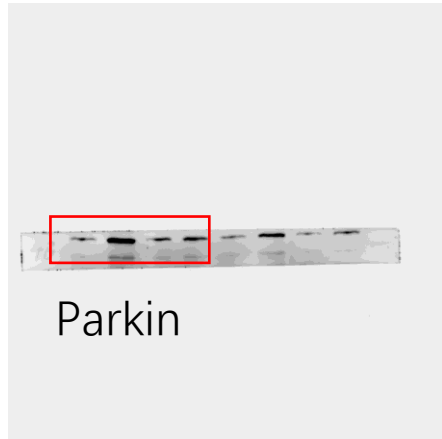

Parkin

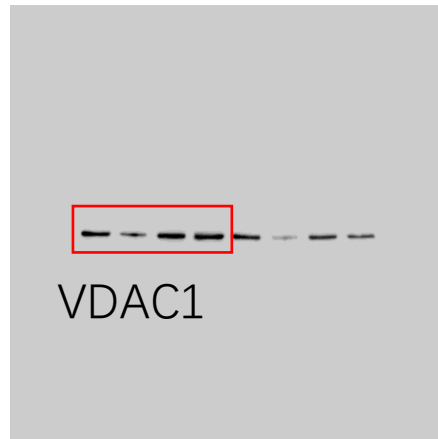

VDAC1

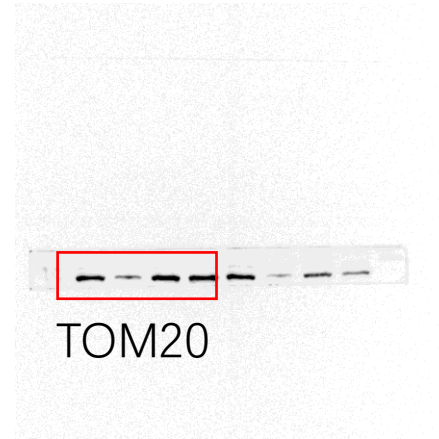

TOM20

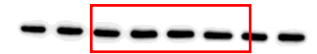

GAPDH

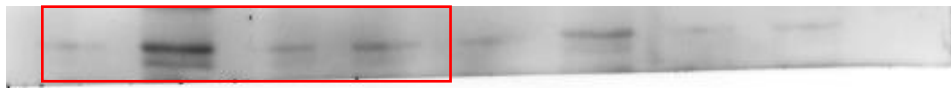

PINK1

Supplement: Supplementary file 3 [file DataSheet1.pdf]

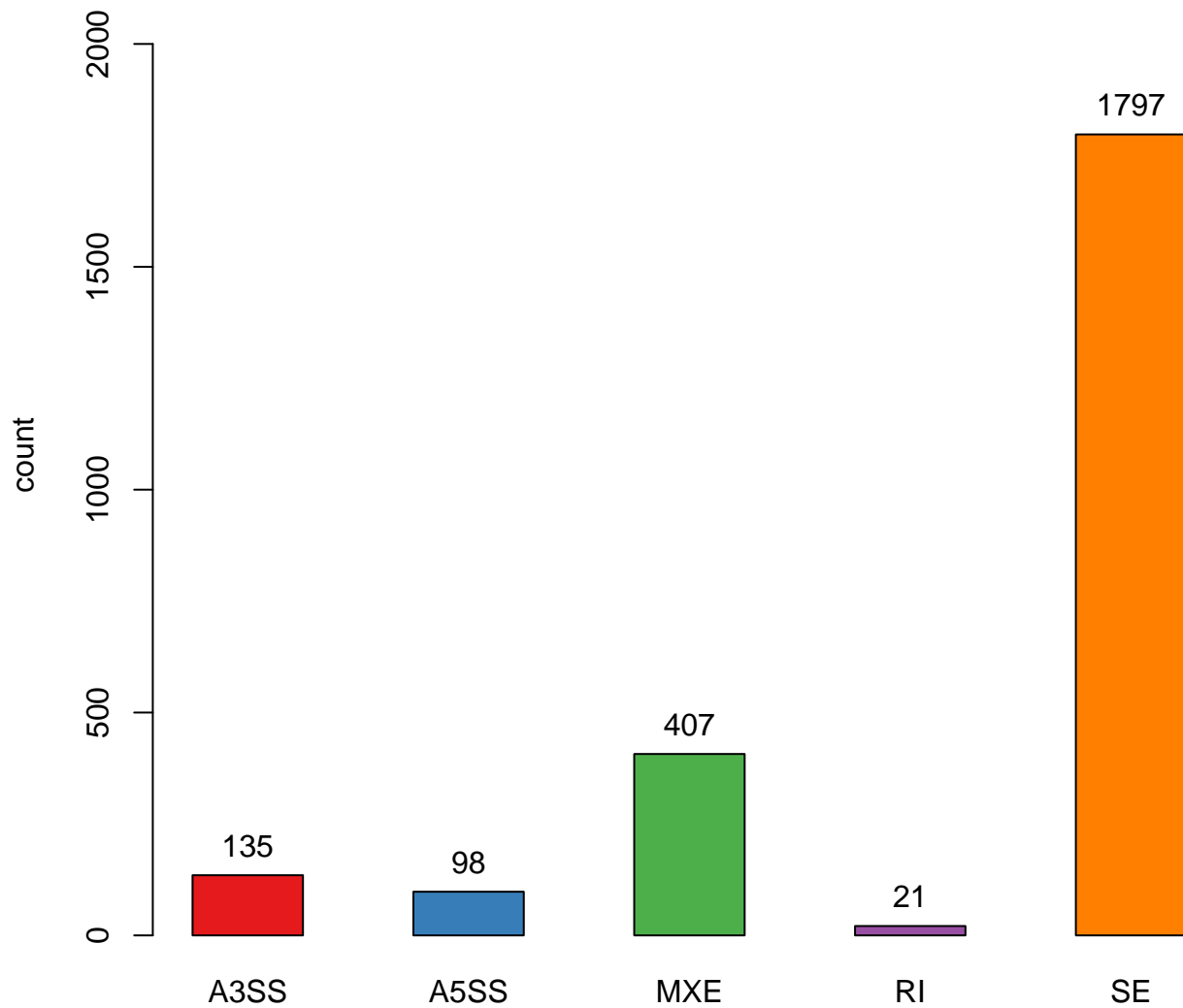

Supplement: Supplementary file 4 [file DataSheet2.zip › supp/AS/GRh2__Ctrl/Junction_event_records_diff_level_plot.pdf]

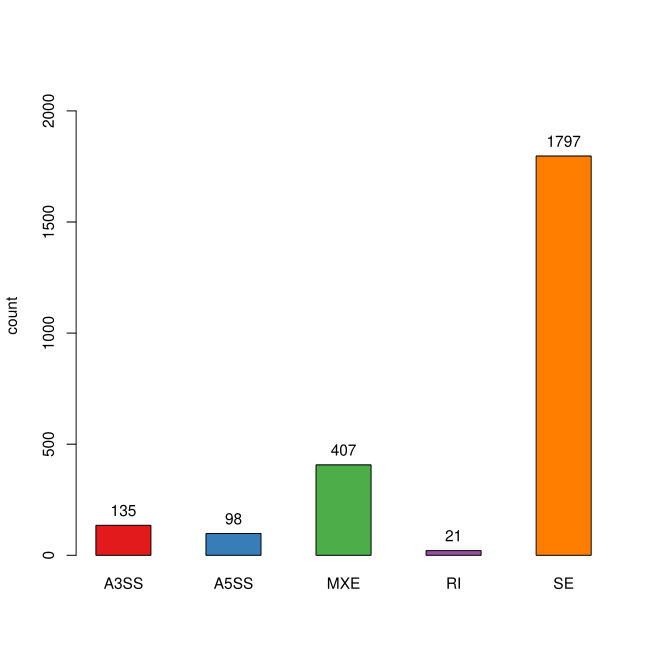

Supplement: Supplementary file 4 [file DataSheet2.zip › supp/AS/GRh2__Ctrl/Junction_event_records_diff_level_plot.png]

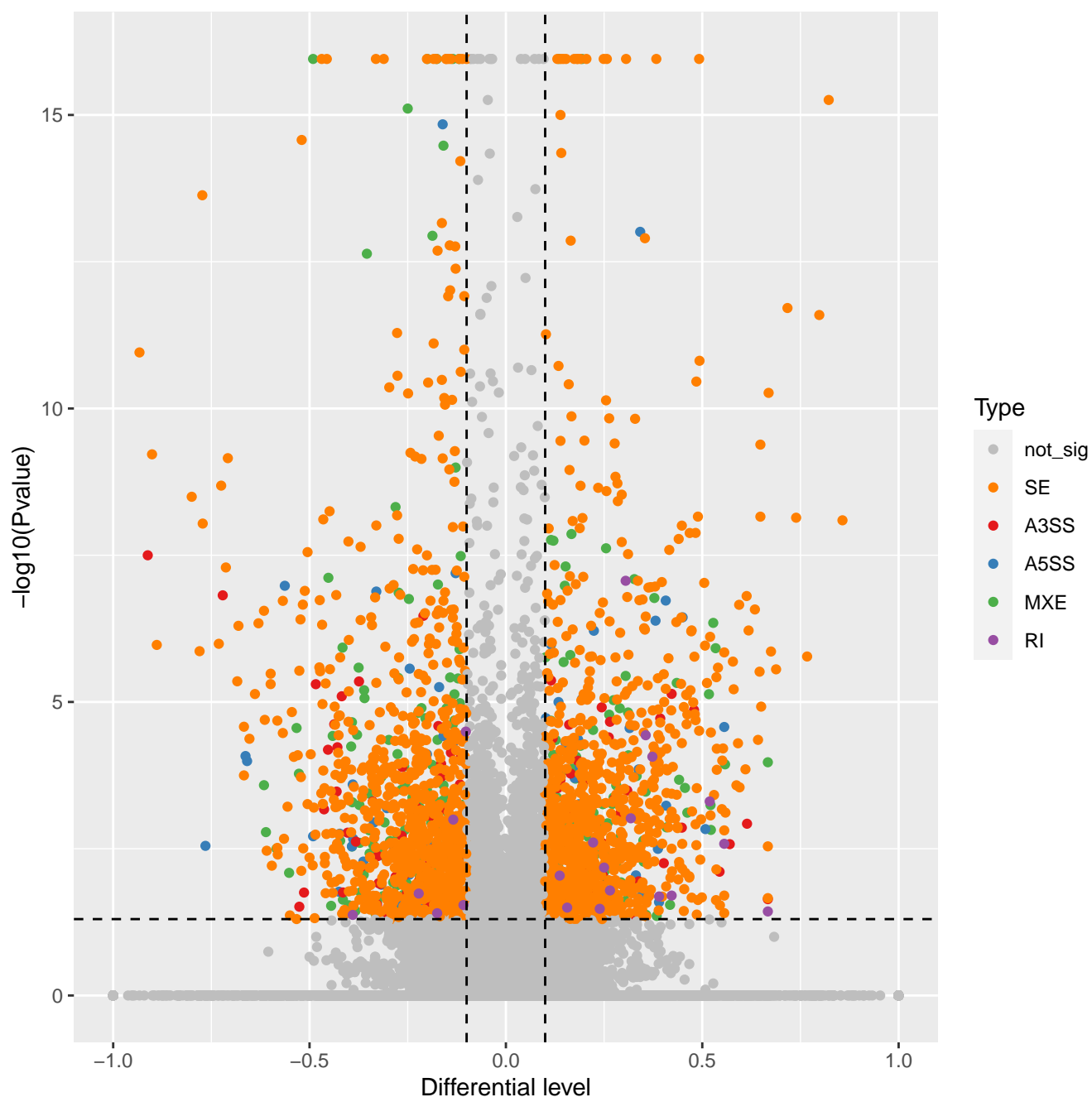

Supplement: Supplementary file 4 [file DataSheet2.zip › supp/AS/GRh2__Ctrl/Junction_event_records_volcan_plot.pdf]

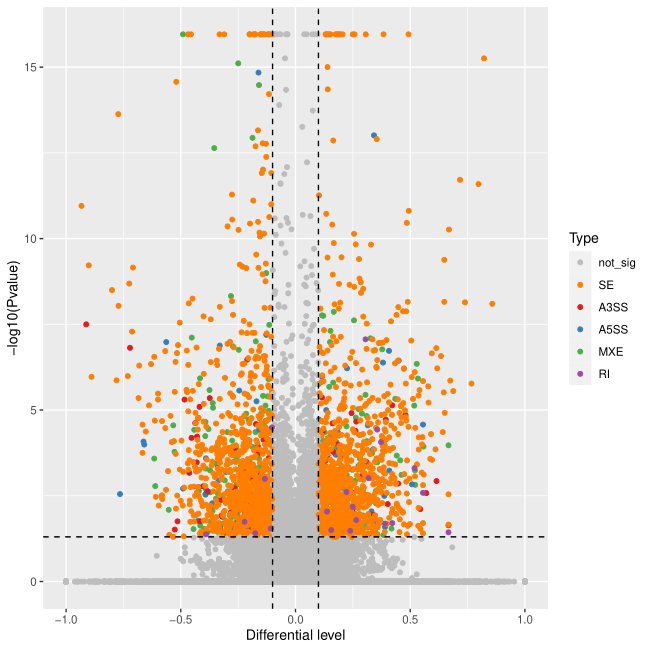

Supplement: Supplementary file 4 [file DataSheet2.zip › supp/AS/GRh2__Ctrl/Junction_event_records_volcan_plot.png]

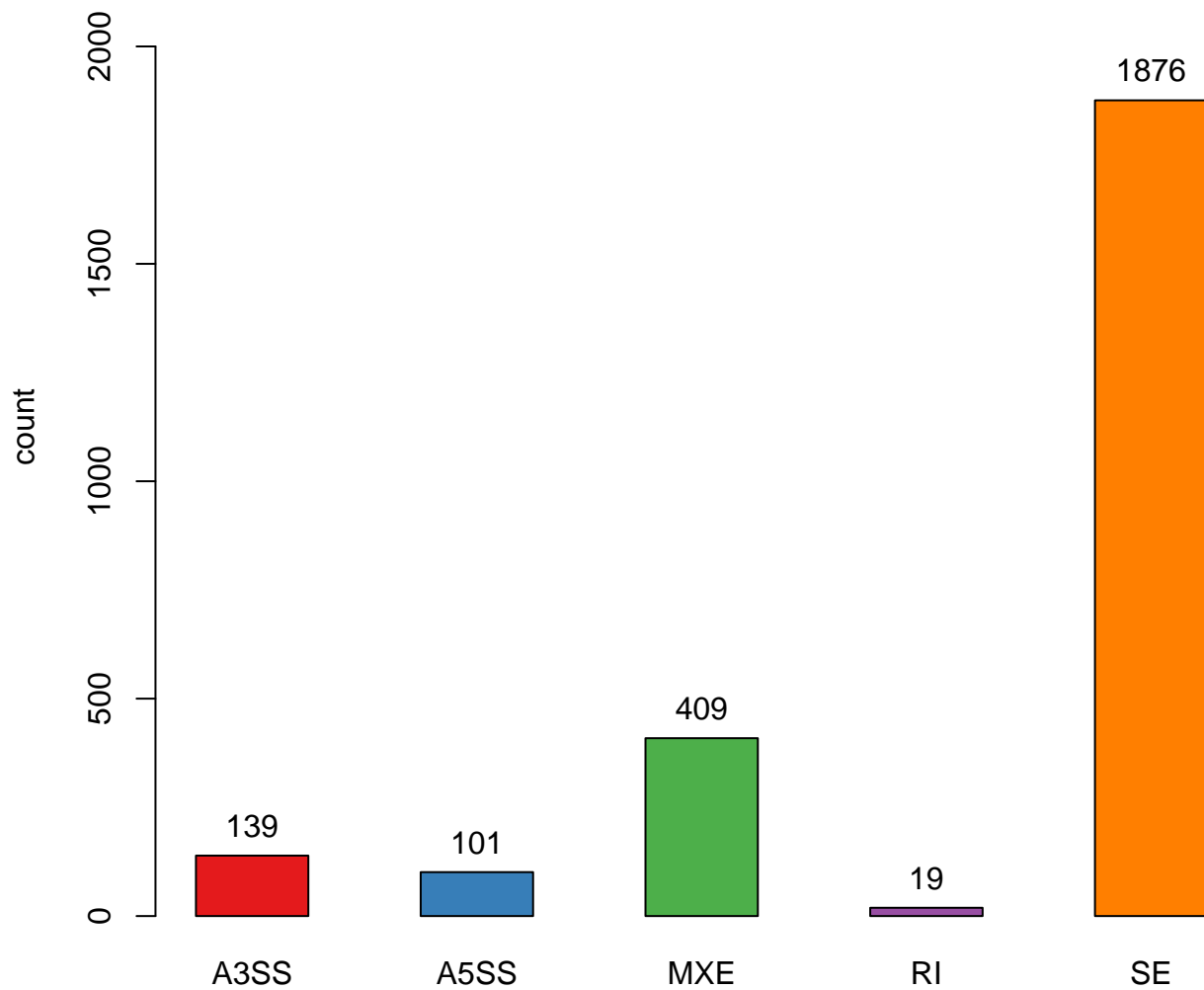

Supplement: Supplementary file 4 [file DataSheet2.zip › supp/AS/GRh2__Ctrl/TargetandJunc_event_records_diff_level_plot.pdf]

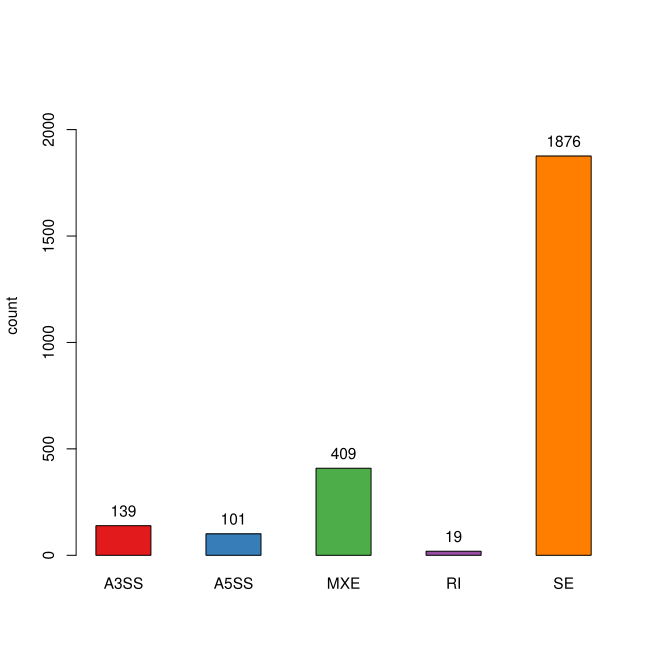

Supplement: Supplementary file 4 [file DataSheet2.zip › supp/AS/GRh2__Ctrl/TargetandJunc_event_records_diff_level_plot.png]

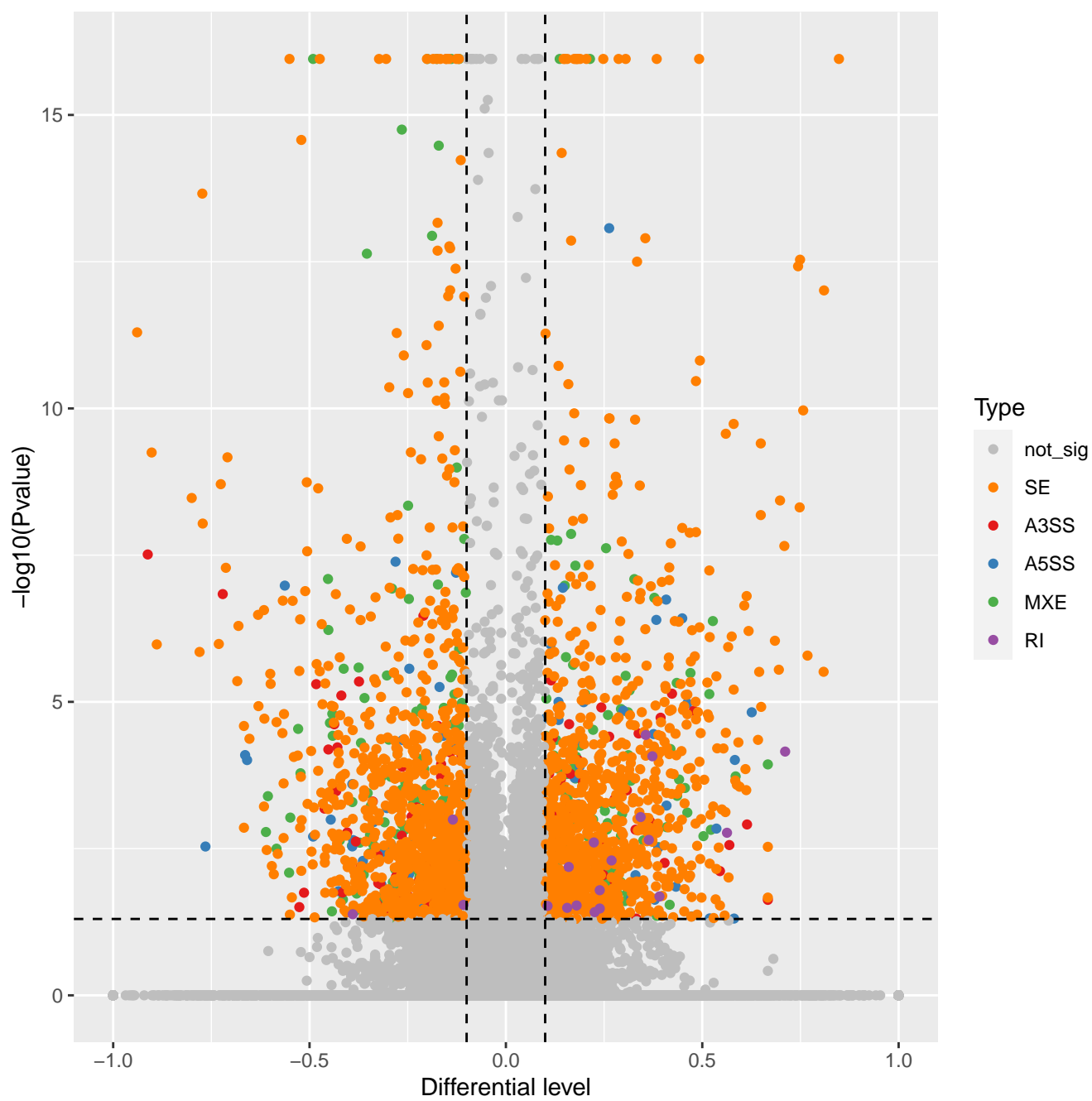

Supplement: Supplementary file 4 [file DataSheet2.zip › supp/AS/GRh2__Ctrl/TargetandJunc_event_records_volcan_plot.pdf]

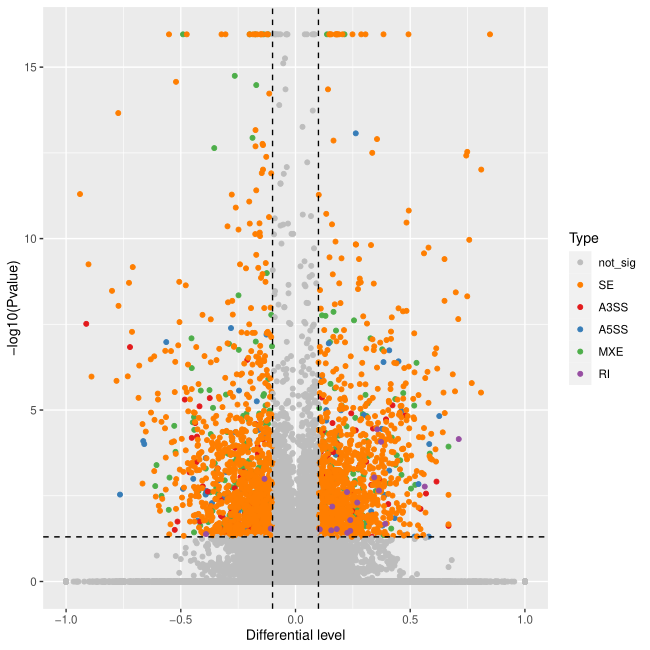

Supplement: Supplementary file 4 [file DataSheet2.zip › supp/AS/GRh2__Ctrl/TargetandJunc_event_records_volcan_plot.png]

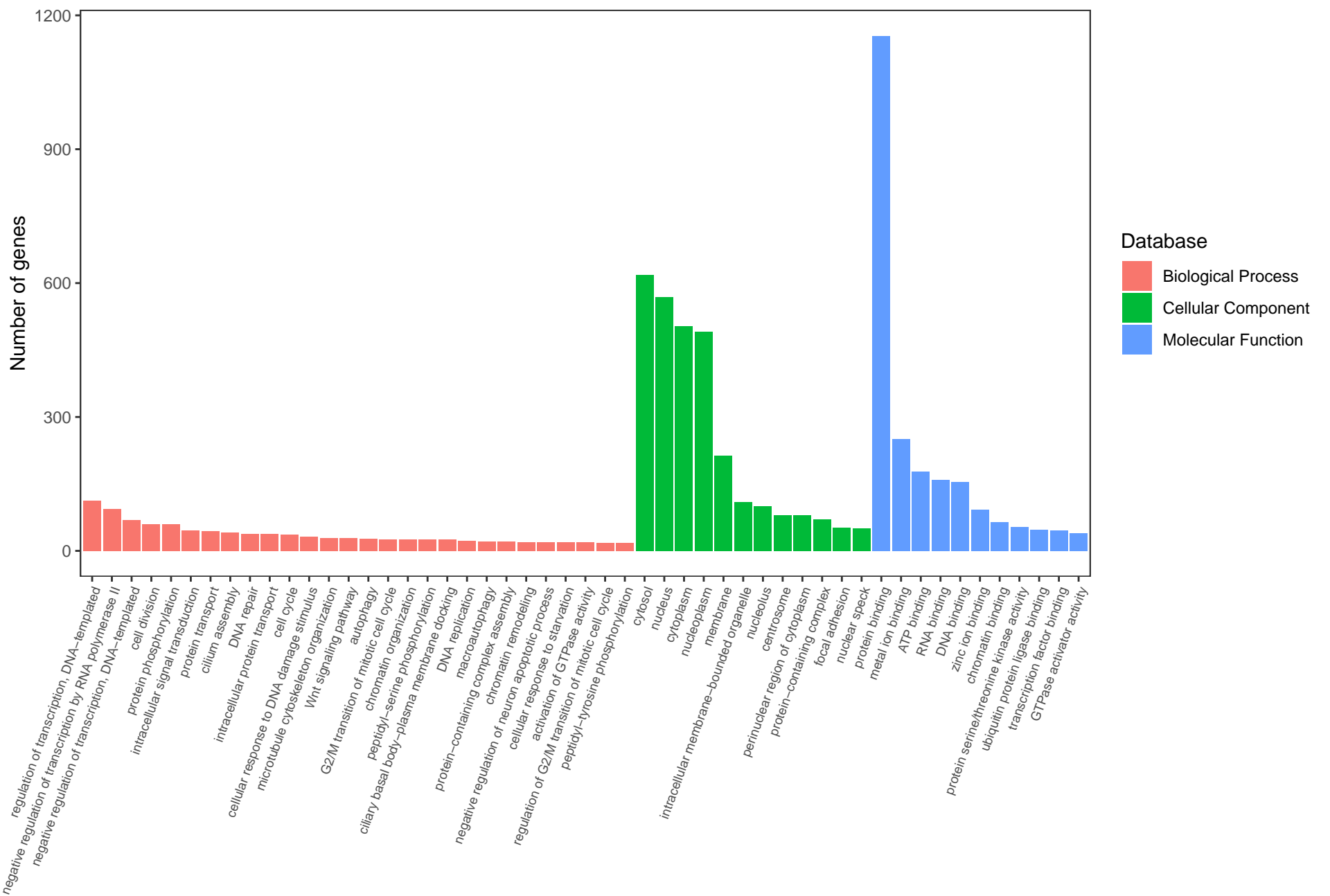

Supplement: Supplementary file 4 [file DataSheet2.zip › supp/AS_GO_KEGG/GRh2__Ctrl/Junction_diff_gene_go.enrich.pdf]

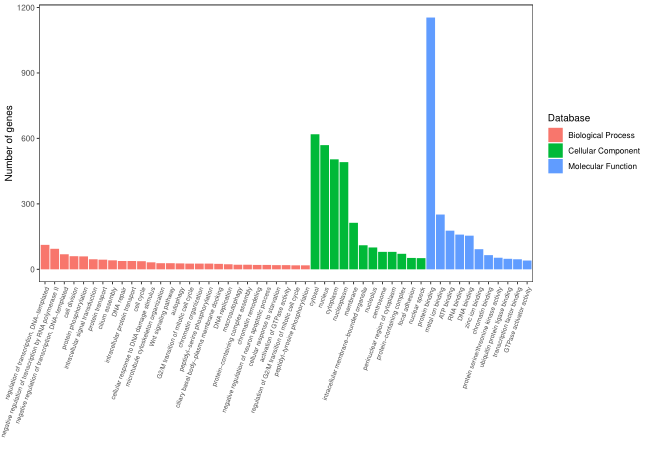

Supplement: Supplementary file 4 [file DataSheet2.zip › supp/AS_GO_KEGG/GRh2__Ctrl/Junction_diff_gene_go.enrich.png]

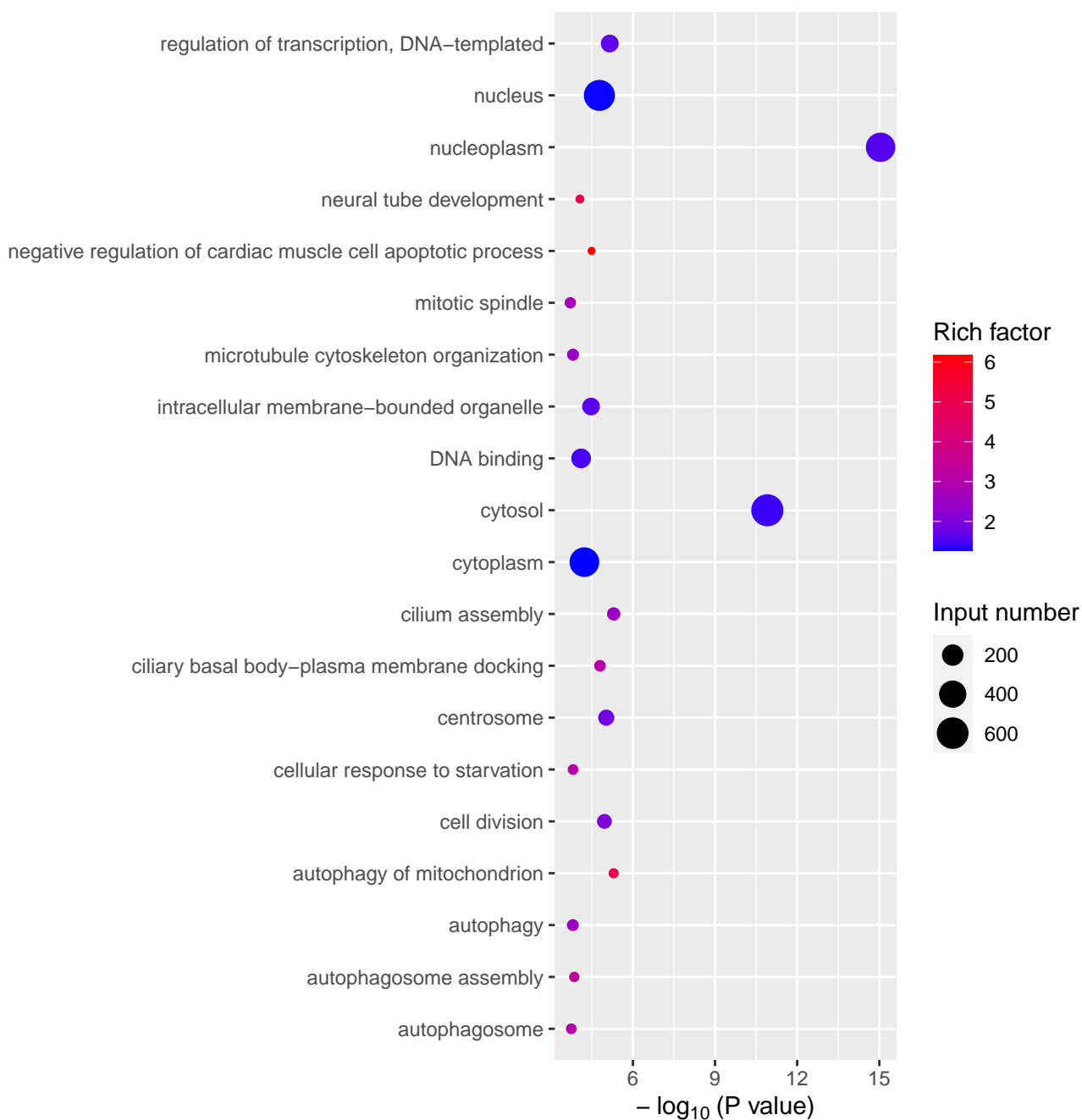

Supplement: Supplementary file 4 [file DataSheet2.zip › supp/AS_GO_KEGG/GRh2__Ctrl/Junction_diff_gene_go.pdf]

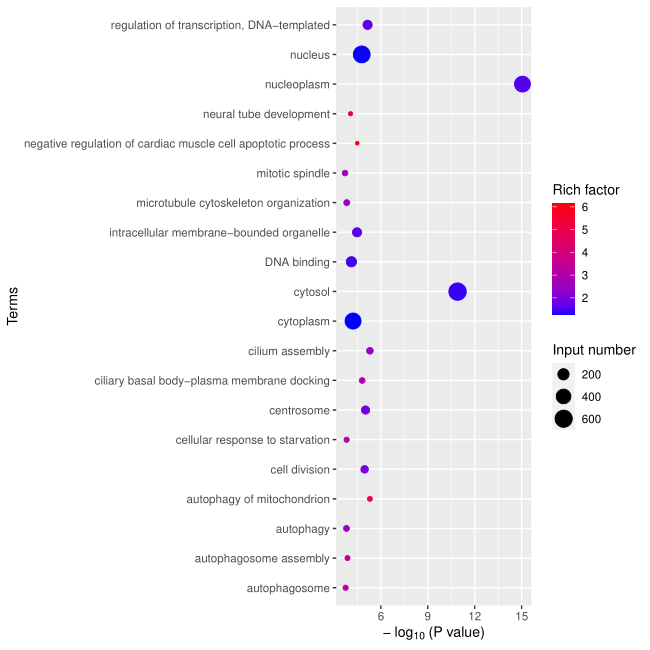

Supplement: Supplementary file 4 [file DataSheet2.zip › supp/AS_GO_KEGG/GRh2__Ctrl/Junction_diff_gene_go.png]

Terms

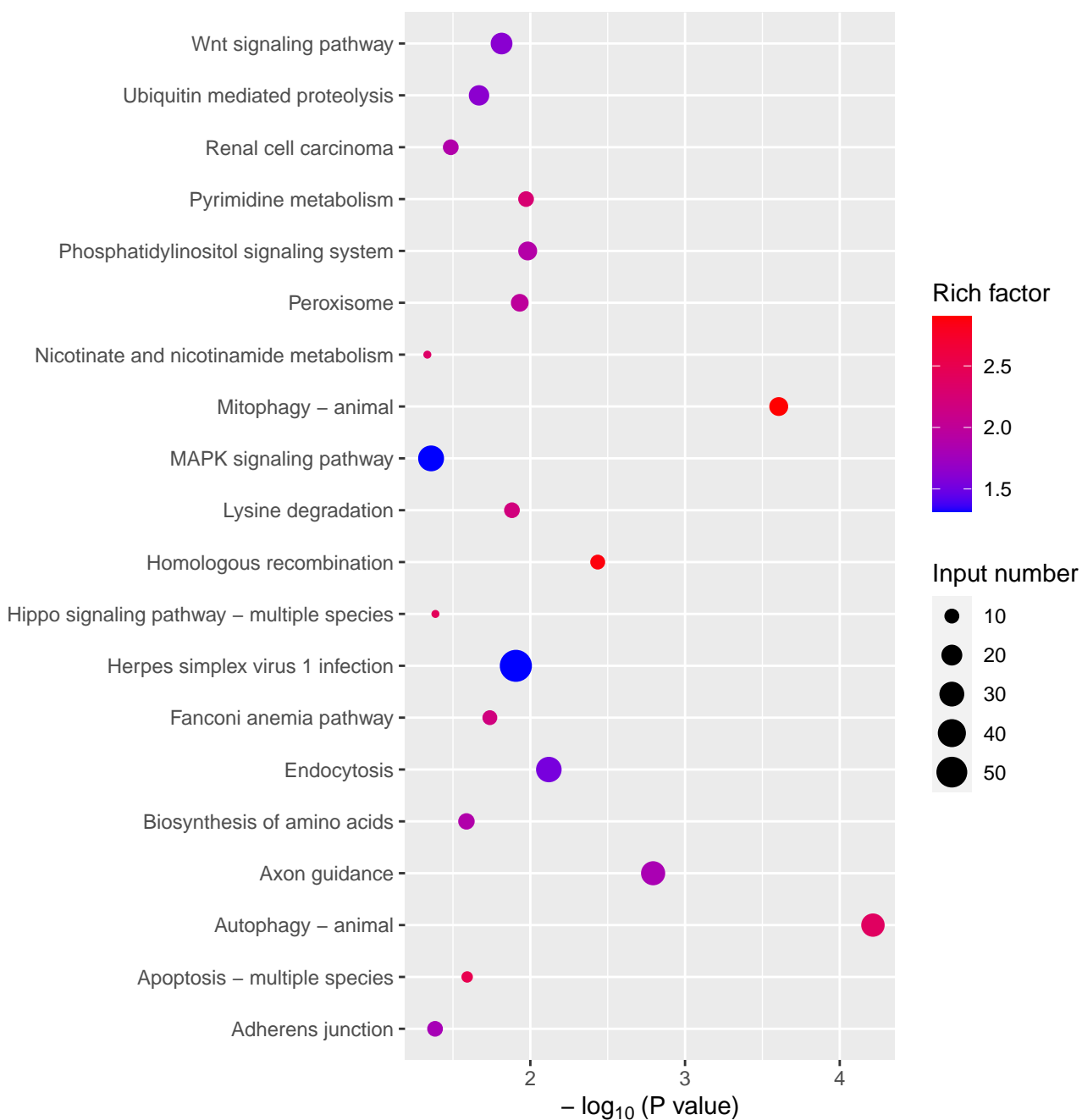

Supplement: Supplementary file 4 [file DataSheet2.zip › supp/AS_GO_KEGG/GRh2__Ctrl/Junction_diff_gene_kegg.pdf]

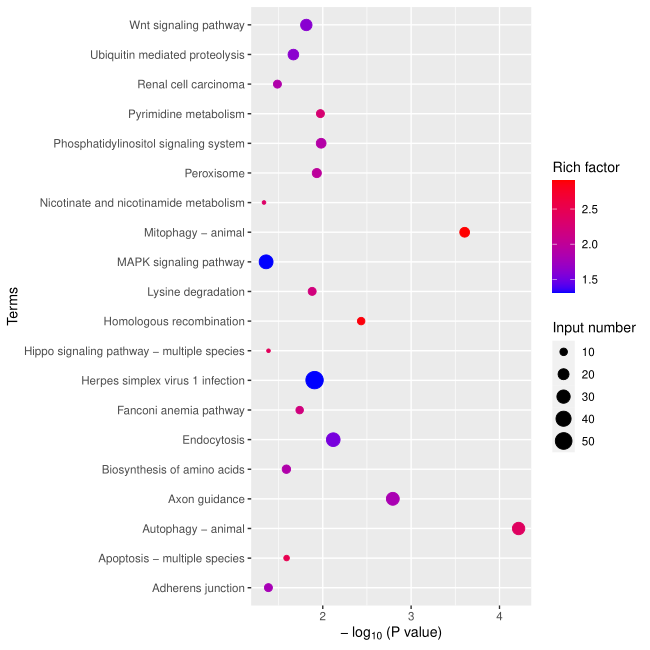

Supplement: Supplementary file 4 [file DataSheet2.zip › supp/AS_GO_KEGG/GRh2__Ctrl/Junction_diff_gene_kegg.png]

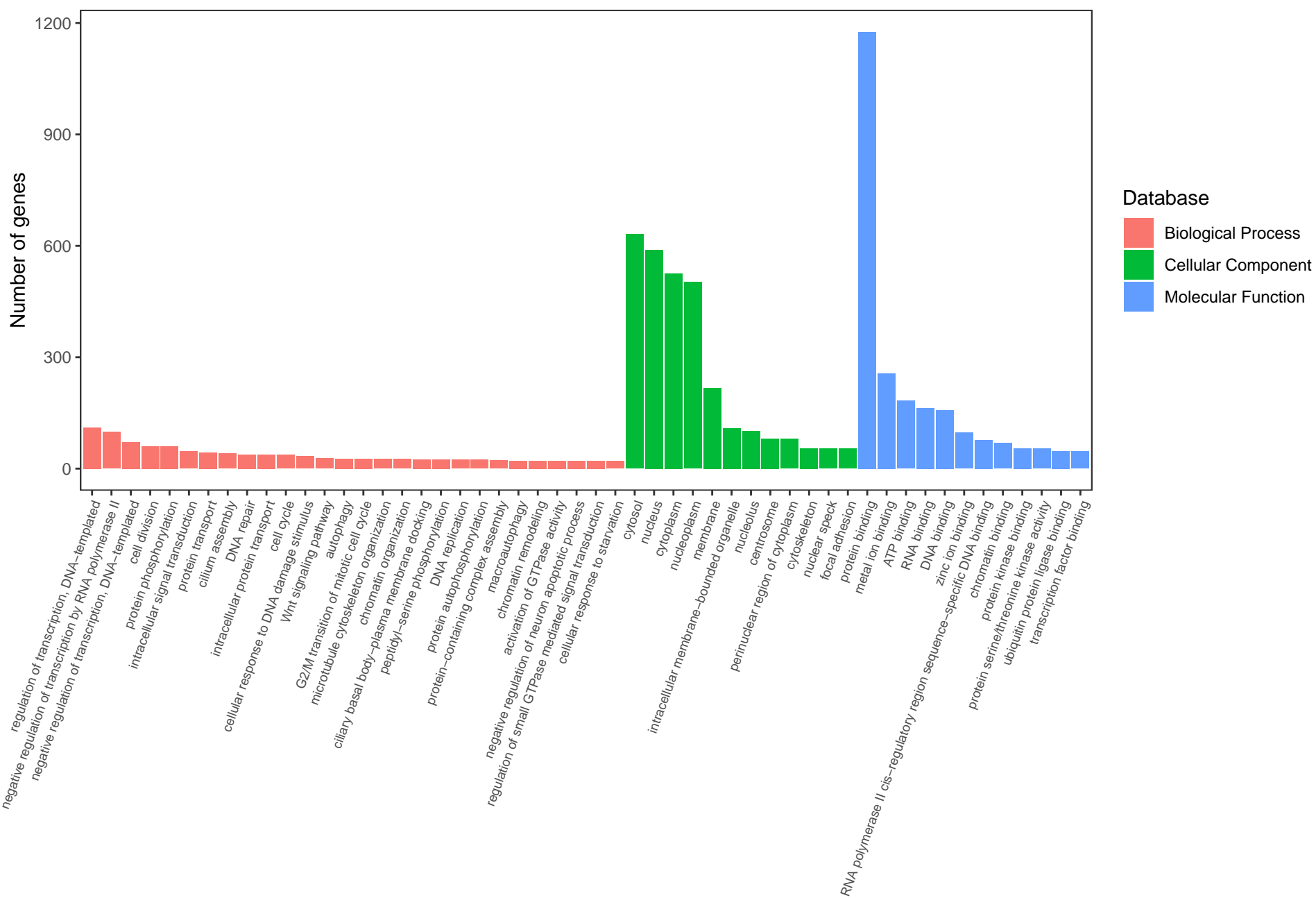

Supplement: Supplementary file 4 [file DataSheet2.zip › supp/AS_GO_KEGG/GRh2__Ctrl/TargetAndJunction_diff_gene_go.enrich.pdf]

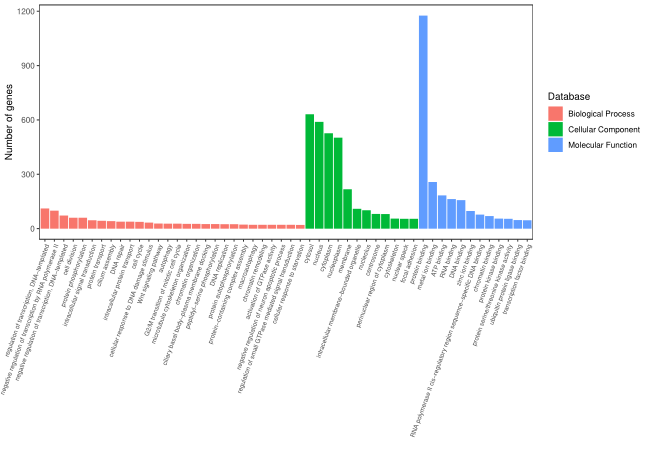

Supplement: Supplementary file 4 [file DataSheet2.zip › supp/AS_GO_KEGG/GRh2__Ctrl/TargetAndJunction_diff_gene_go.enrich.png]

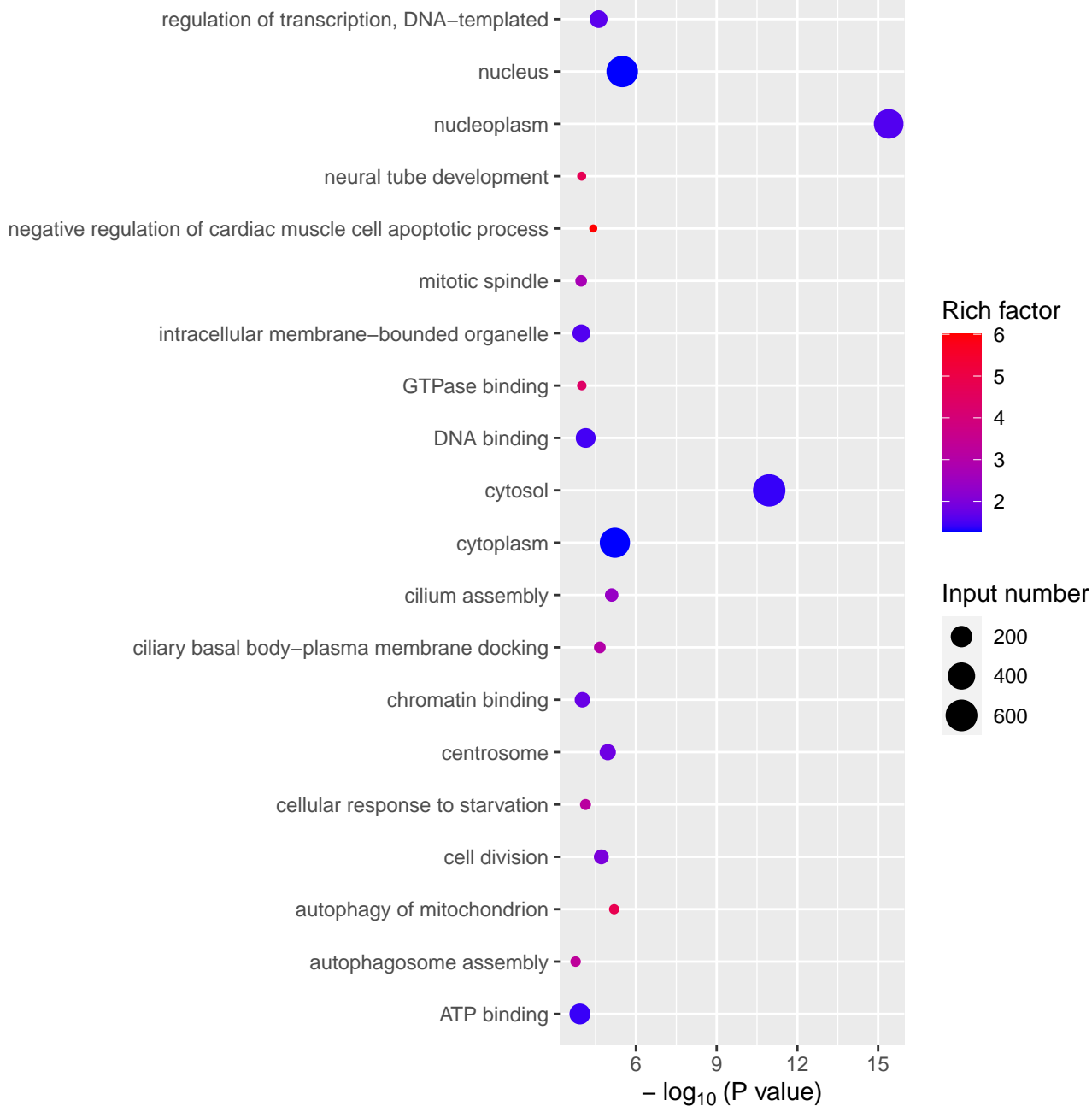

Supplement: Supplementary file 4 [file DataSheet2.zip › supp/AS_GO_KEGG/GRh2__Ctrl/TargetAndJunction_diff_gene_go.pdf]

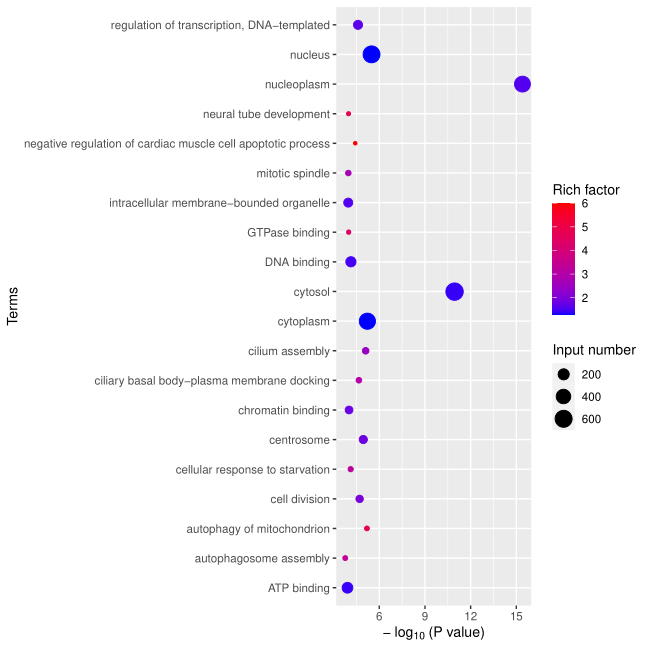

Supplement: Supplementary file 4 [file DataSheet2.zip › supp/AS_GO_KEGG/GRh2__Ctrl/TargetAndJunction_diff_gene_go.png]

Terms

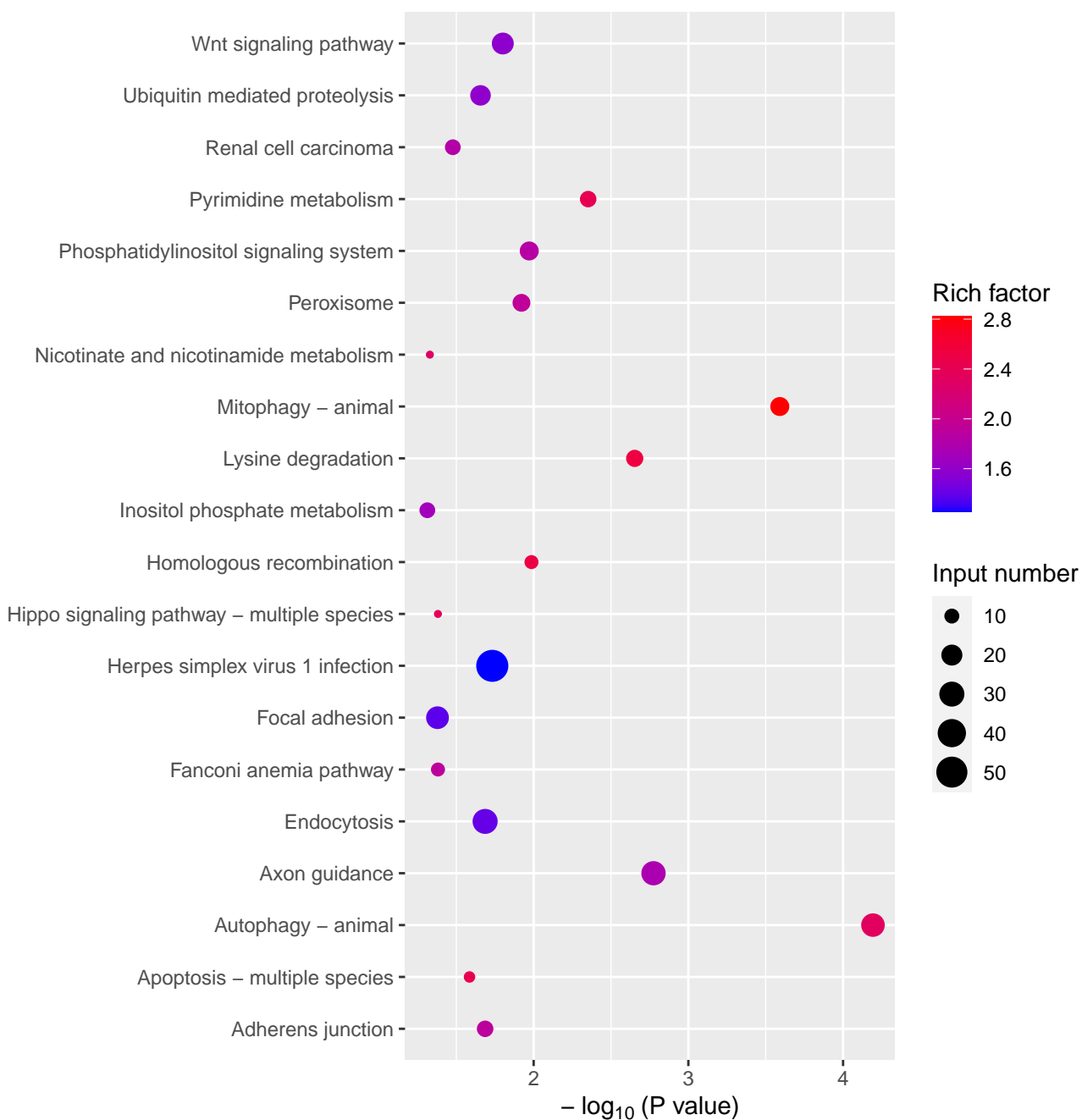

Supplement: Supplementary file 4 [file DataSheet2.zip › supp/AS_GO_KEGG/GRh2__Ctrl/TargetAndJunction_diff_gene_kegg.pdf]

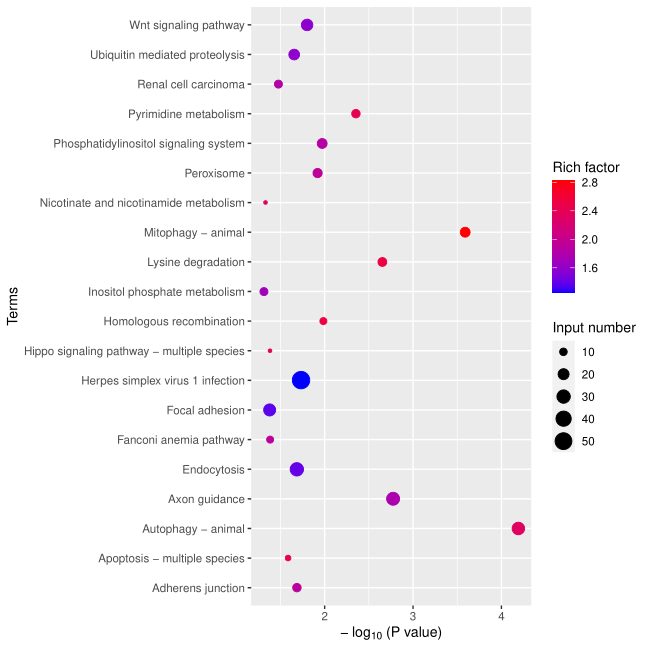

Supplement: Supplementary file 4 [file DataSheet2.zip › supp/AS_GO_KEGG/GRh2__Ctrl/TargetAndJunction_diff_gene_kegg.png]

Up-regulated genes: 1541  
Down-regulated genes: 1484

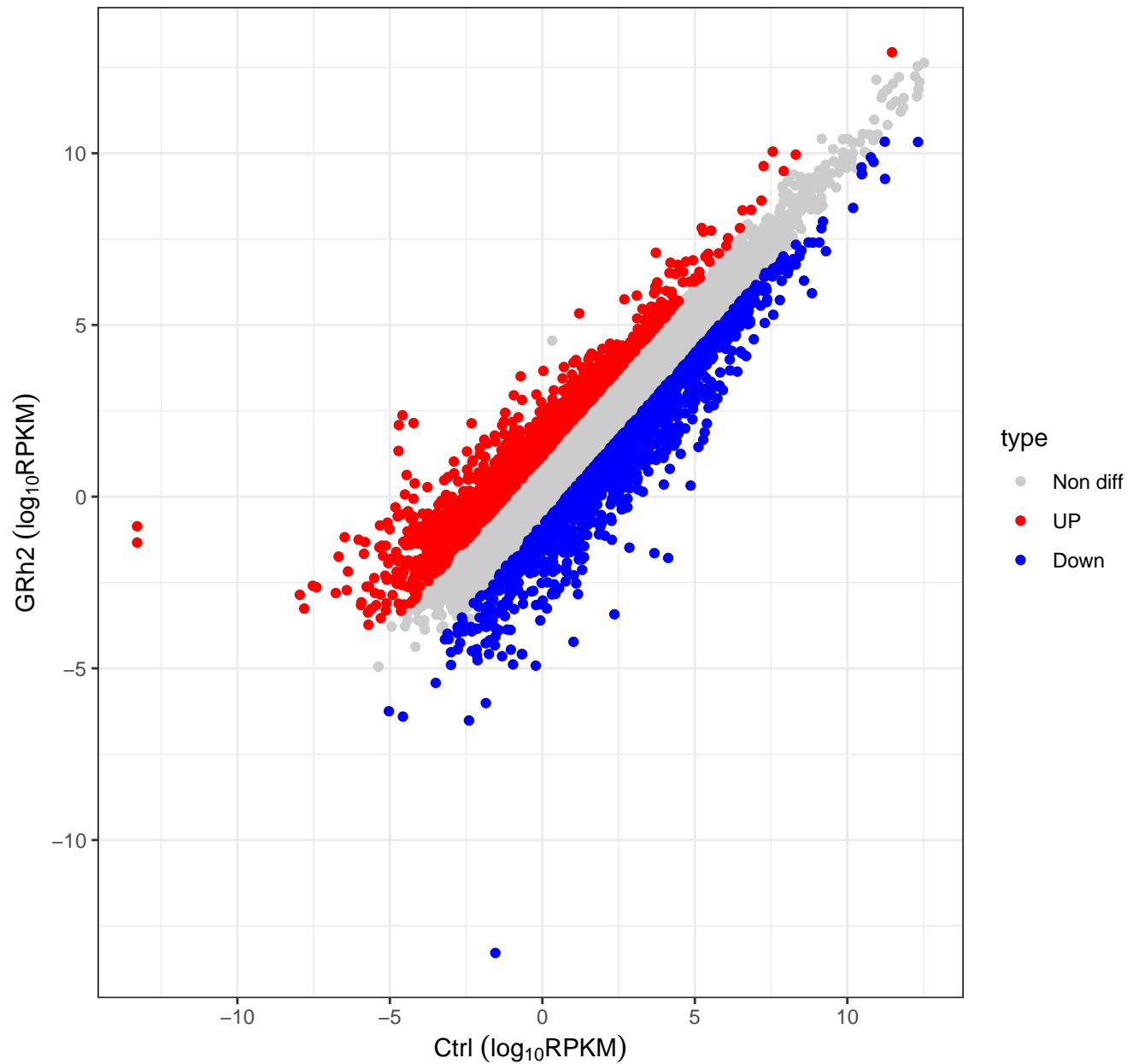

Supplement: Supplementary file 4 [file DataSheet2.zip › supp/DEG/GRh2___Ctrl/diff_expr_plot.pdf]

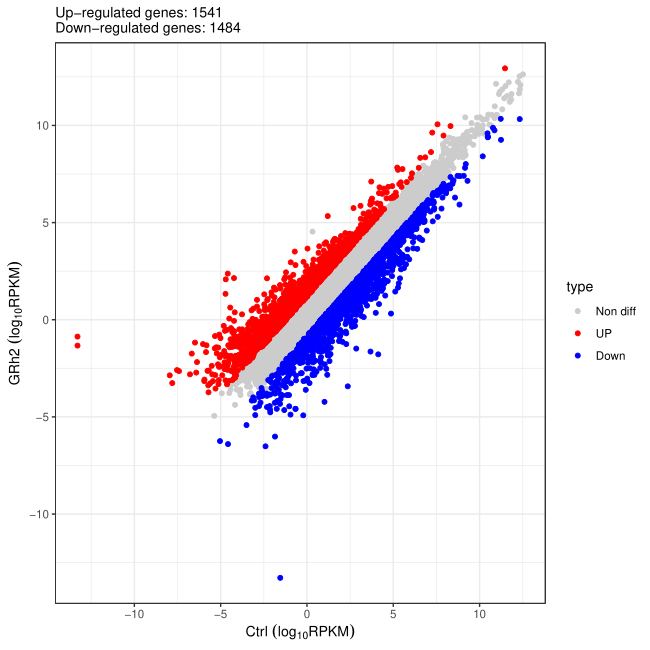

Supplement: Supplementary file 4 [file DataSheet2.zip › supp/DEG/GRh2___Ctrl/diff_expr_plot.png]

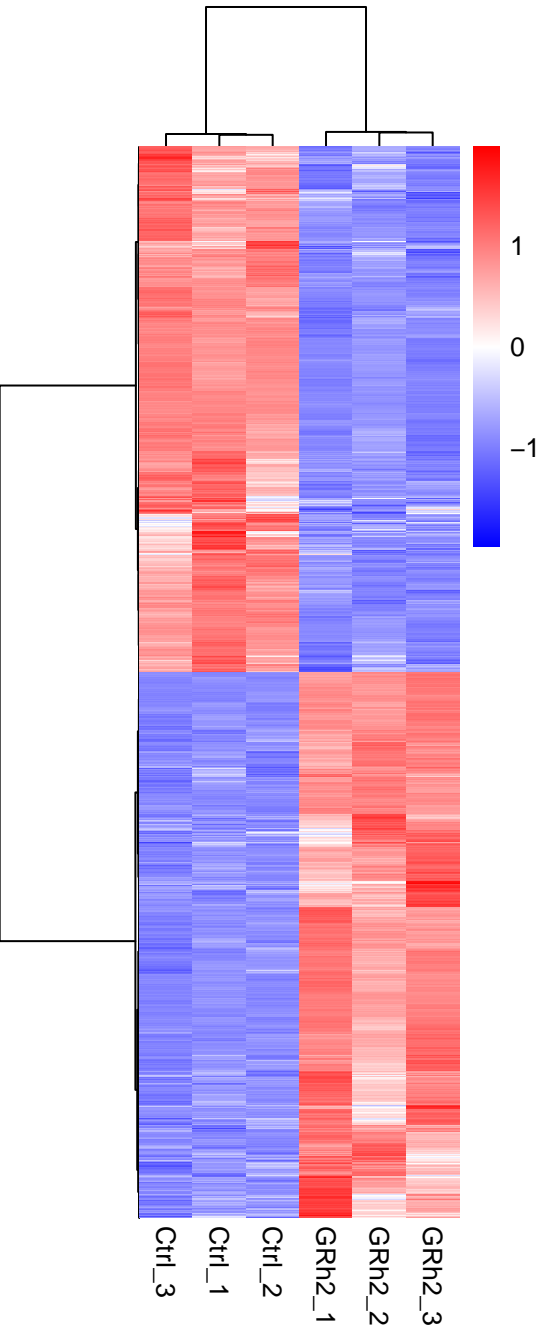

Supplement: Supplementary file 4 [file DataSheet2.zip › supp/DEG/GRh2___Ctrl/heatmap_of_any_diff_genes_1.pdf]

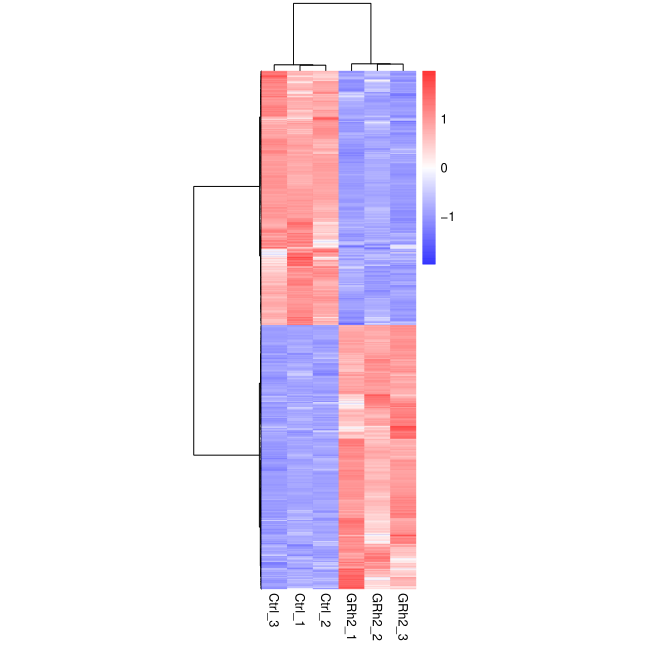

Supplement: Supplementary file 4 [file DataSheet2.zip › supp/DEG/GRh2___Ctrl/heatmap_of_any_diff_genes_1.png]

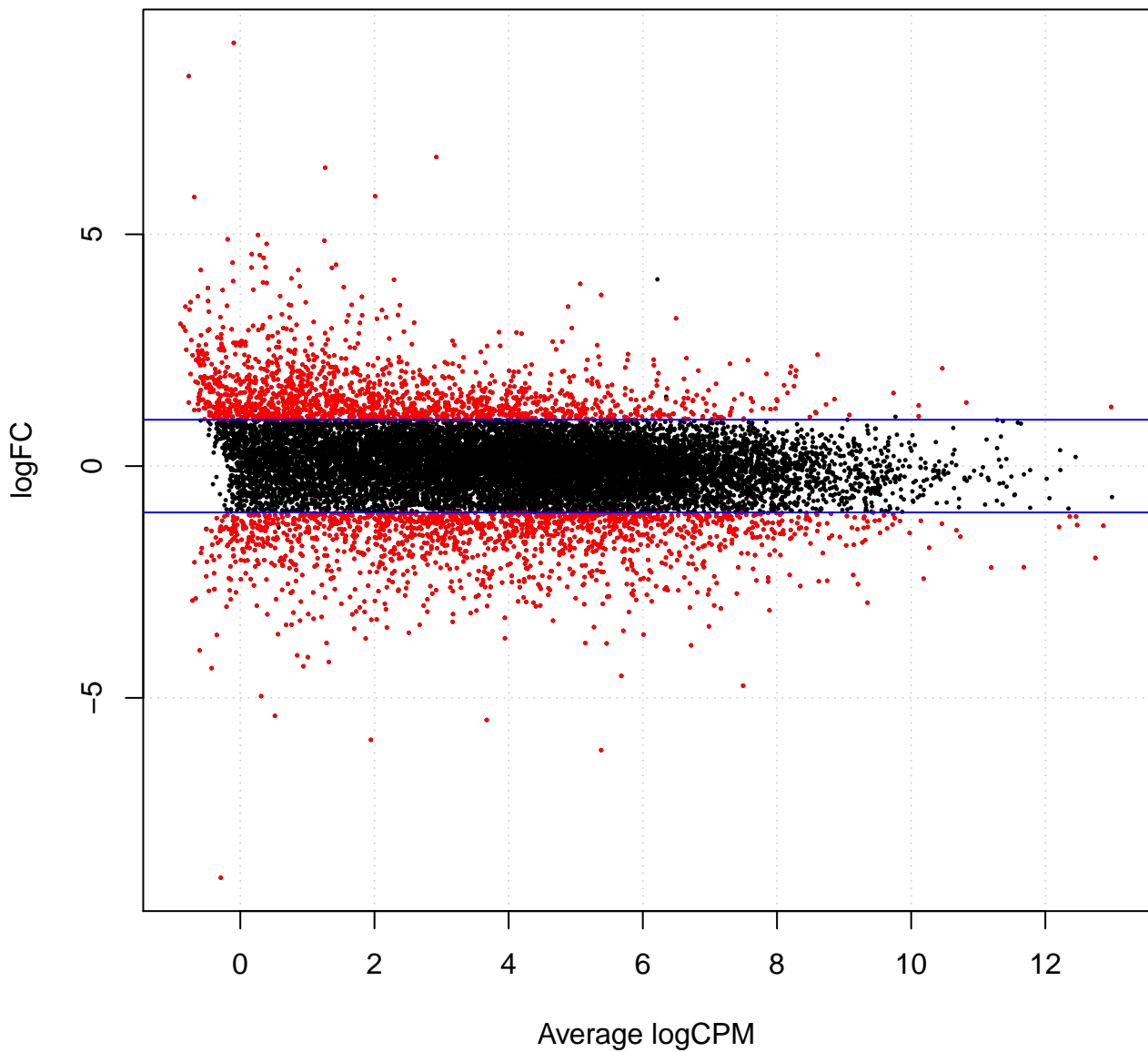

Supplement: Supplementary file 4 [file DataSheet2.zip › supp/DEG/GRh2___Ctrl/MA_plot.pdf]

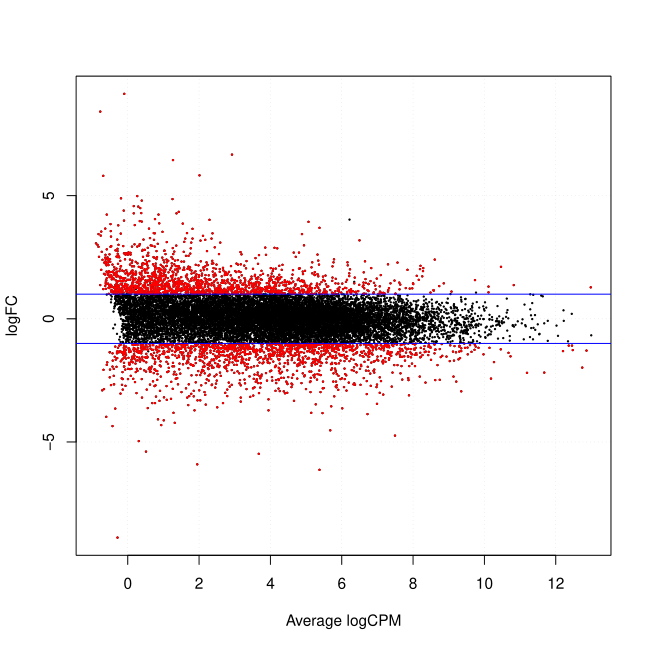

Supplement: Supplementary file 4 [file DataSheet2.zip › supp/DEG/GRh2___Ctrl/MA_plot.png]

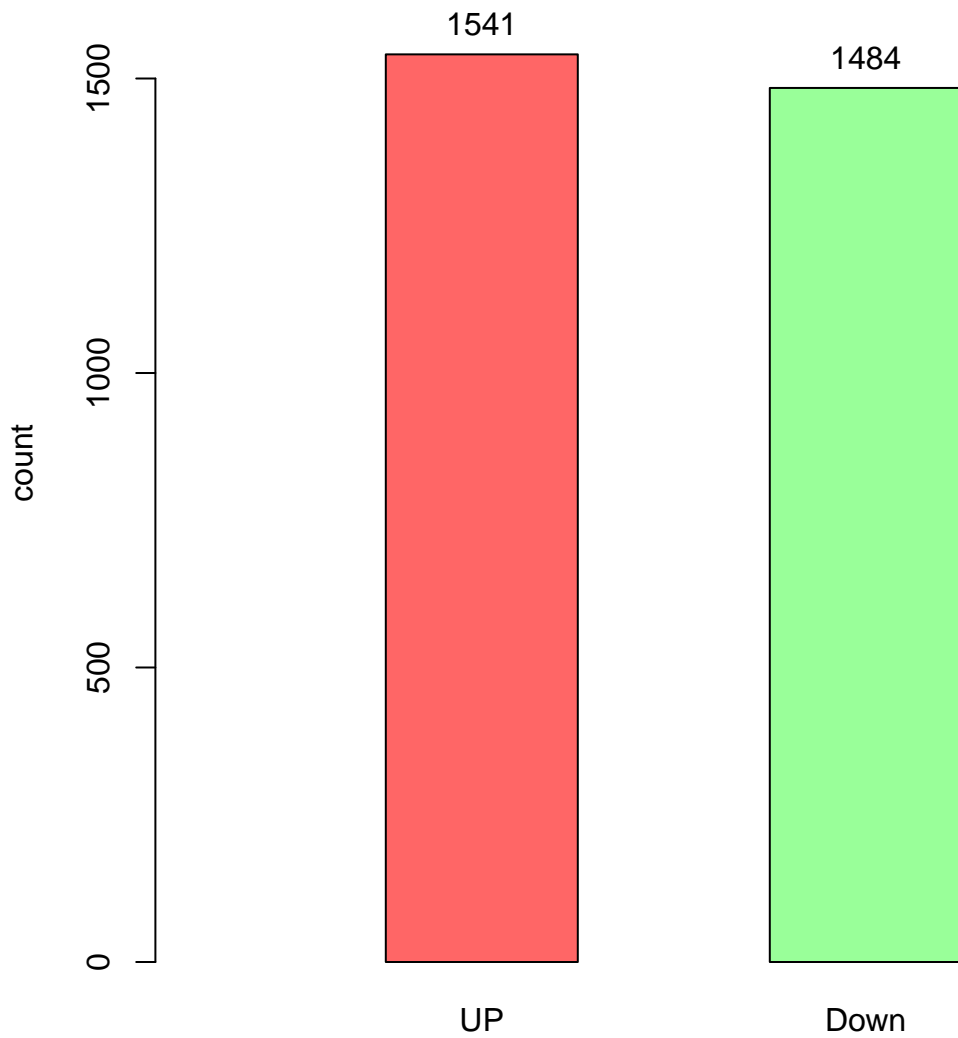

Supplement: Supplementary file 4 [file DataSheet2.zip › supp/DEG/GRh2___Ctrl/numbers_of_diff_genes.pdf]

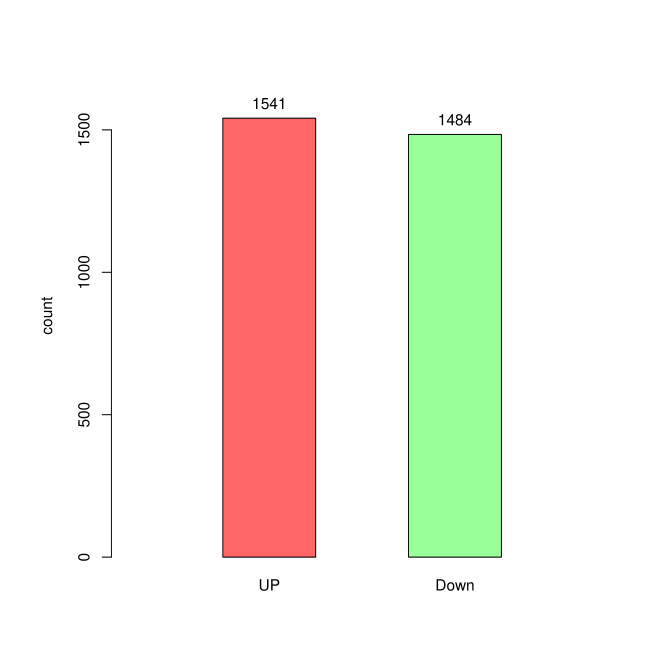

Supplement: Supplementary file 4 [file DataSheet2.zip › supp/DEG/GRh2___Ctrl/numbers_of_diff_genes.png]

Volcano Plot

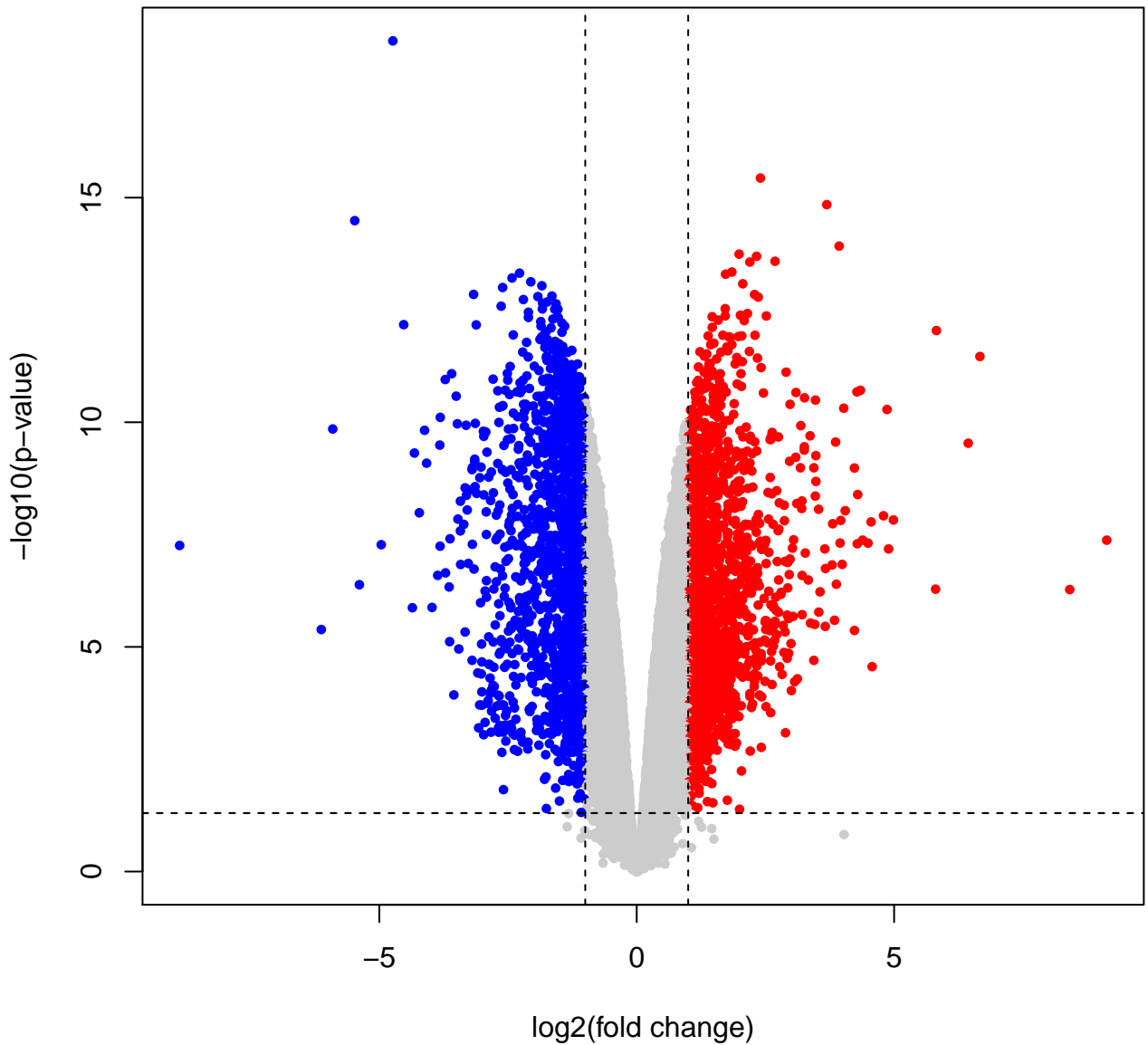

Supplement: Supplementary file 4 [file DataSheet2.zip › supp/DEG/GRh2___Ctrl/volcano_plot.pdf]

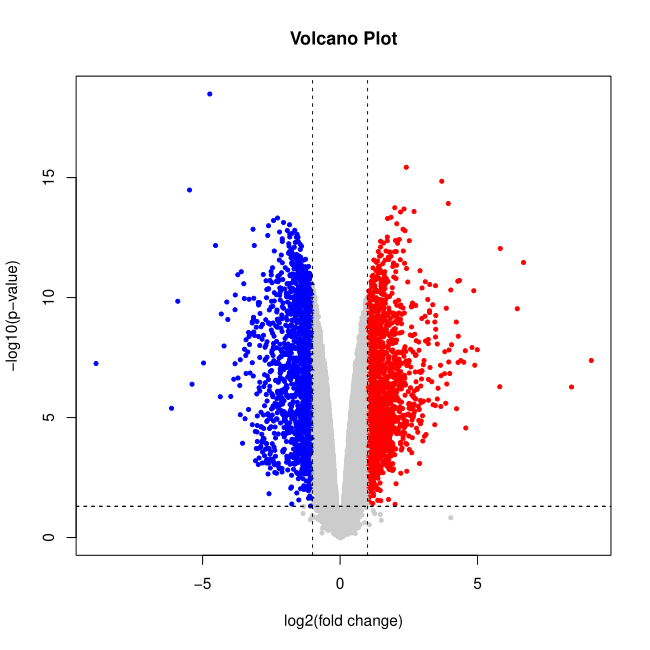

Supplement: Supplementary file 4 [file DataSheet2.zip › supp/DEG/GRh2___Ctrl/volcano_plot.png]

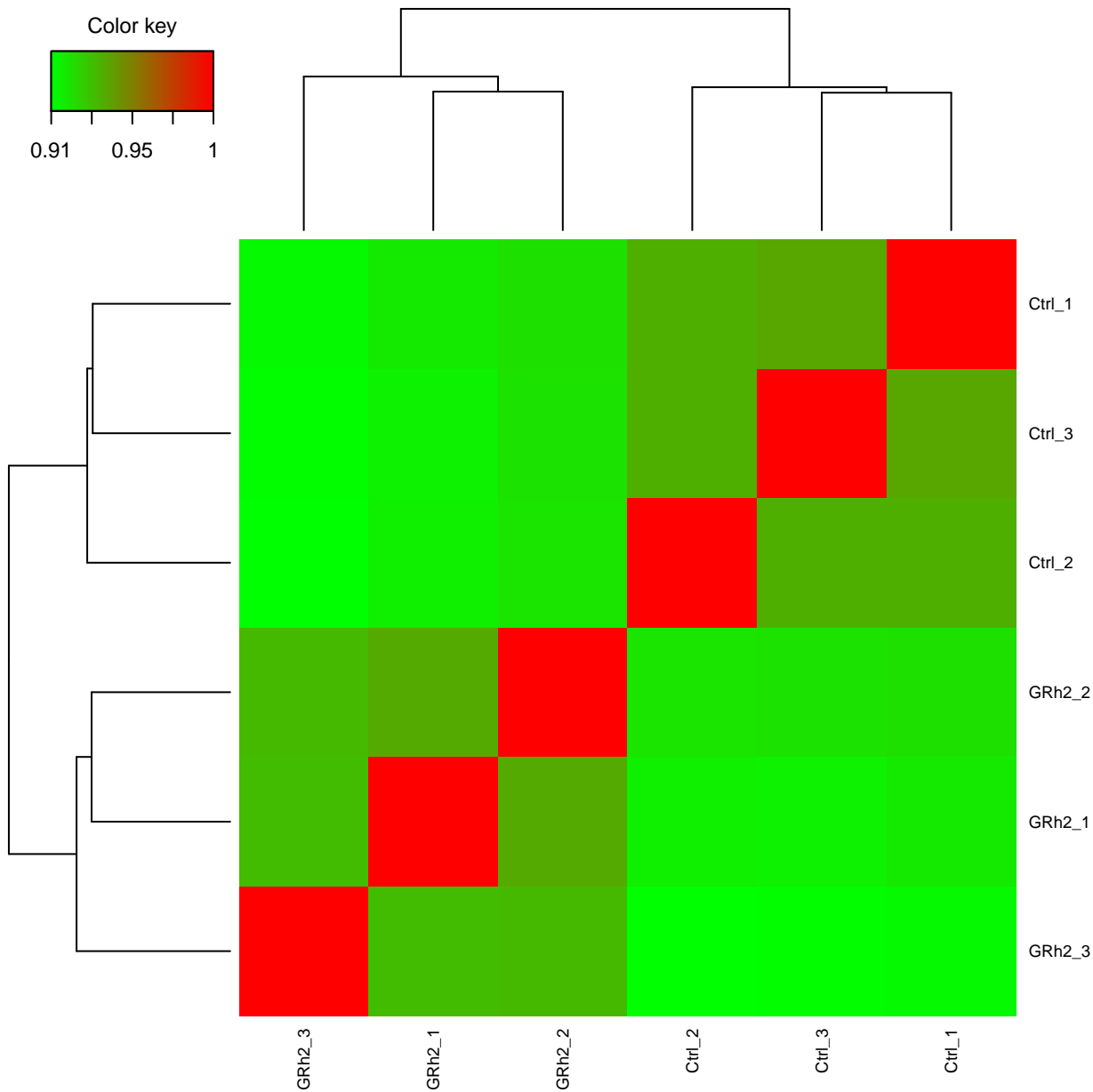

Supplement: Supplementary file 4 [file DataSheet2.zip › supp/DEG/Sample_corr_heatmap.pdf]

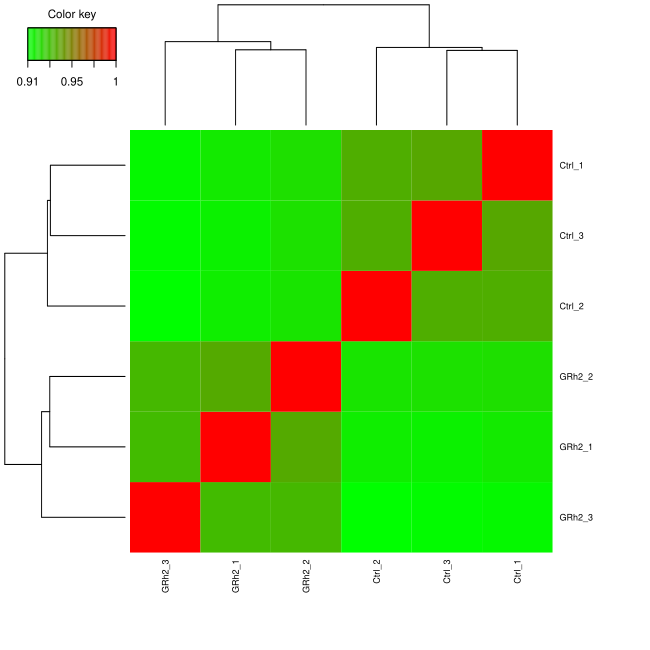

Supplement: Supplementary file 4 [file DataSheet2.zip › supp/DEG/Sample_corr_heatmap.png]

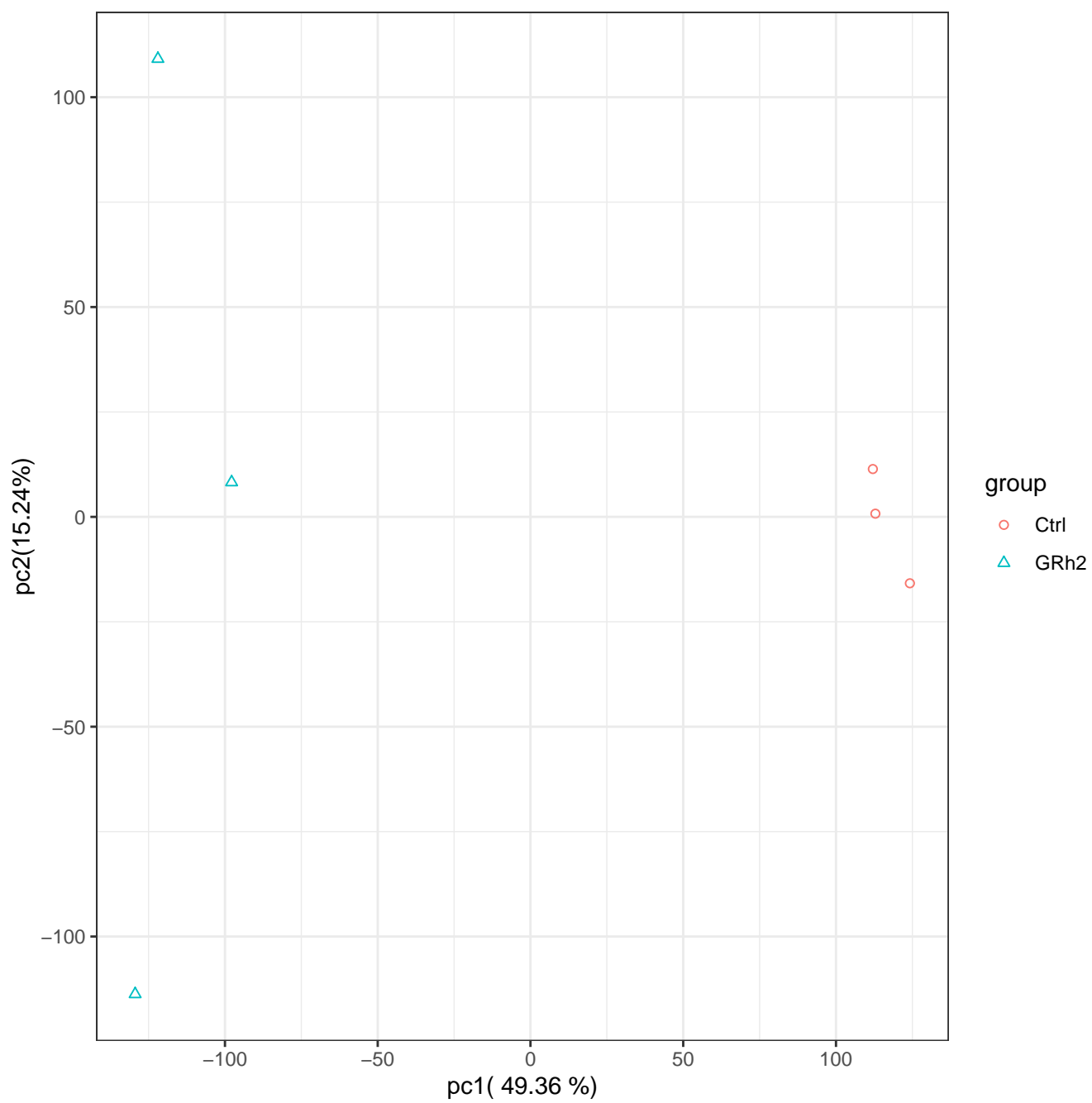

Supplement: Supplementary file 4 [file DataSheet2.zip › supp/DEG/Sample_PCA_correlation.pdf]

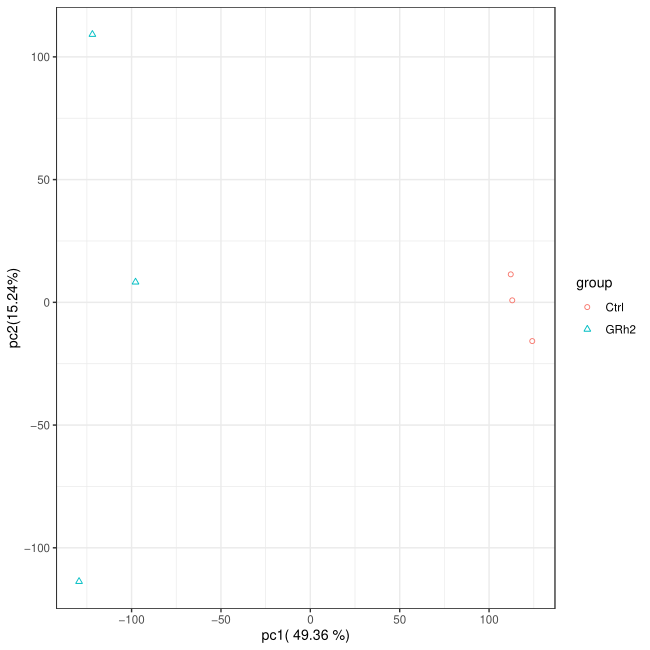

Supplement: Supplementary file 4 [file DataSheet2.zip › supp/DEG/Sample_PCA_correlation.png]

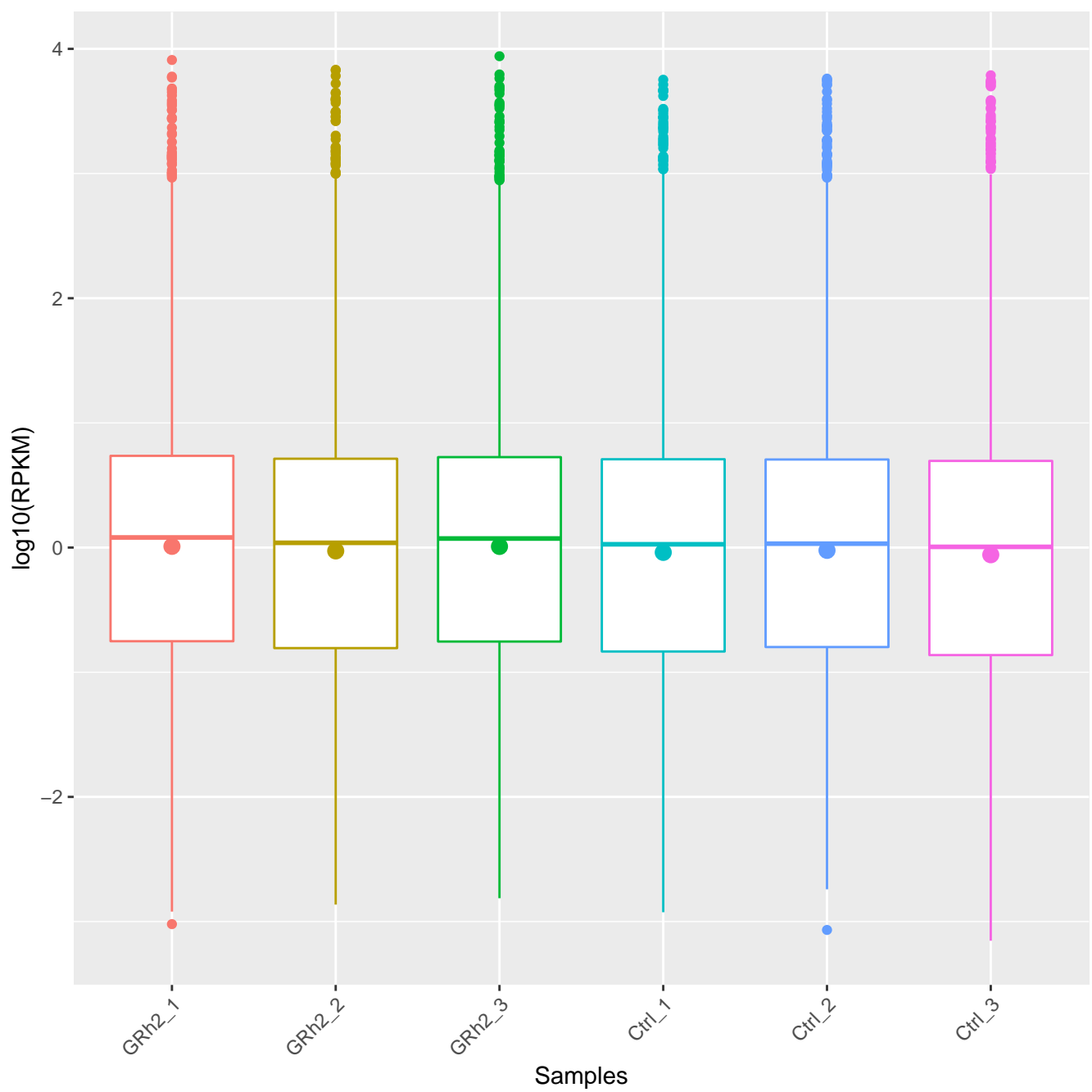

Supplement: Supplementary file 4 [file DataSheet2.zip › supp/DEG/Sample_RPKM_boxplot.pdf]

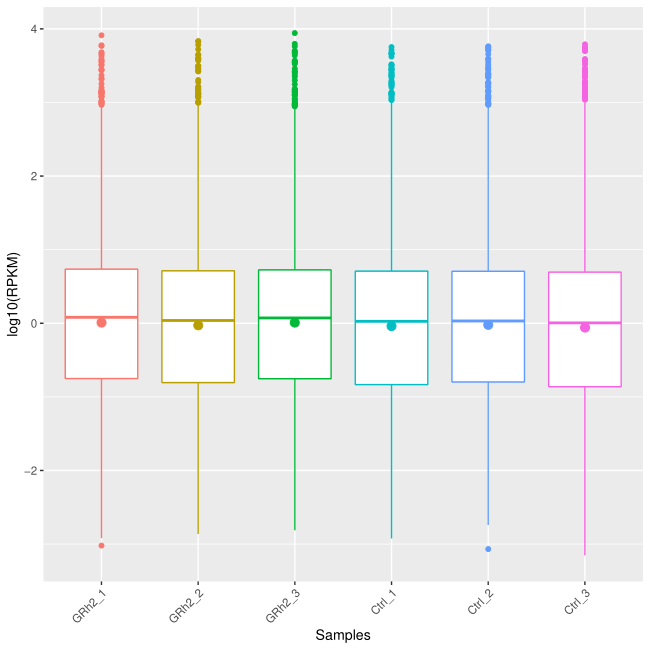

Supplement: Supplementary file 4 [file DataSheet2.zip › supp/DEG/Sample_RPKM_boxplot.png]

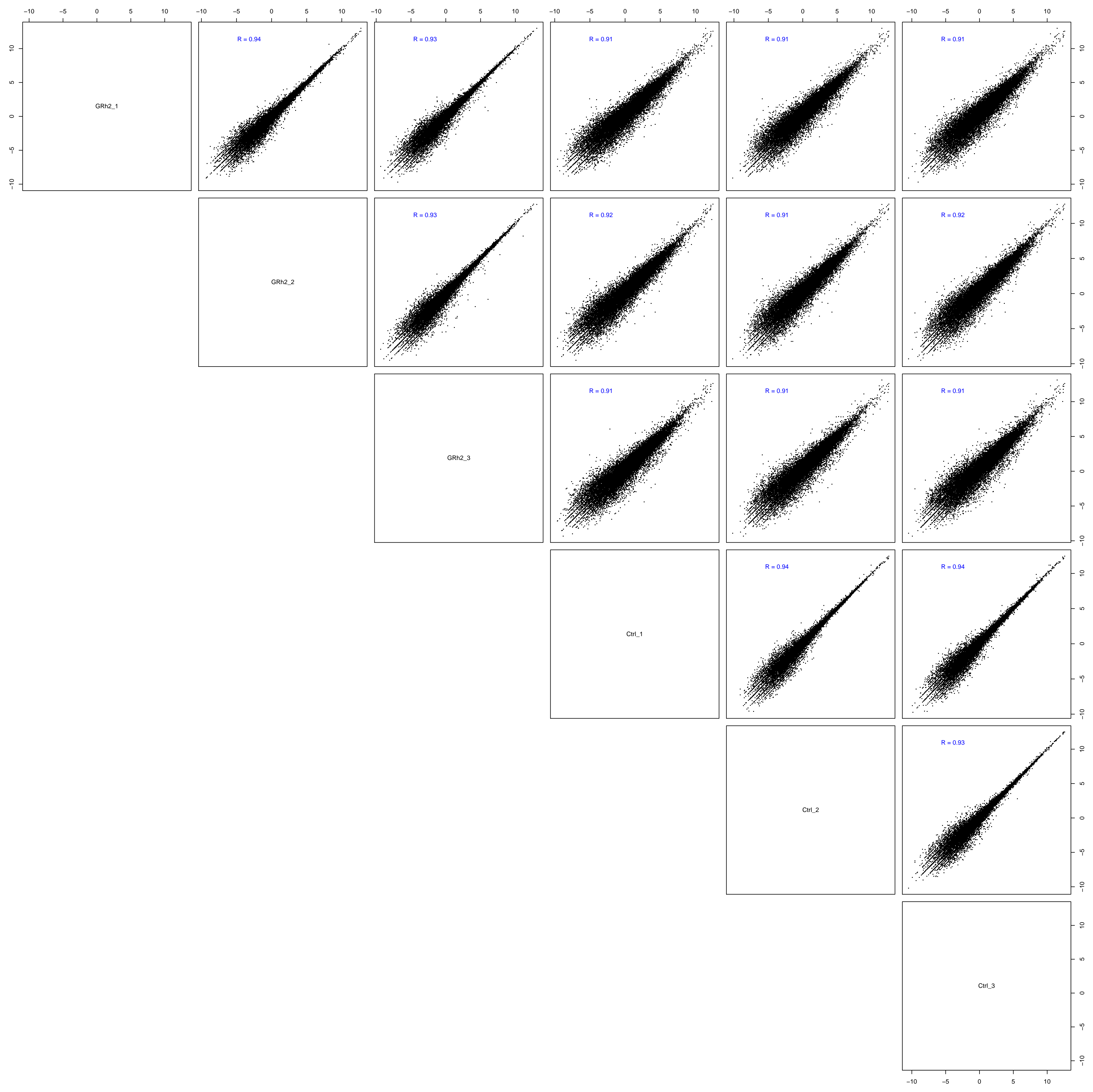

Supplement: Supplementary file 4 [file DataSheet2.zip › supp/DEG/Sample_RPKM_correlation.pdf]

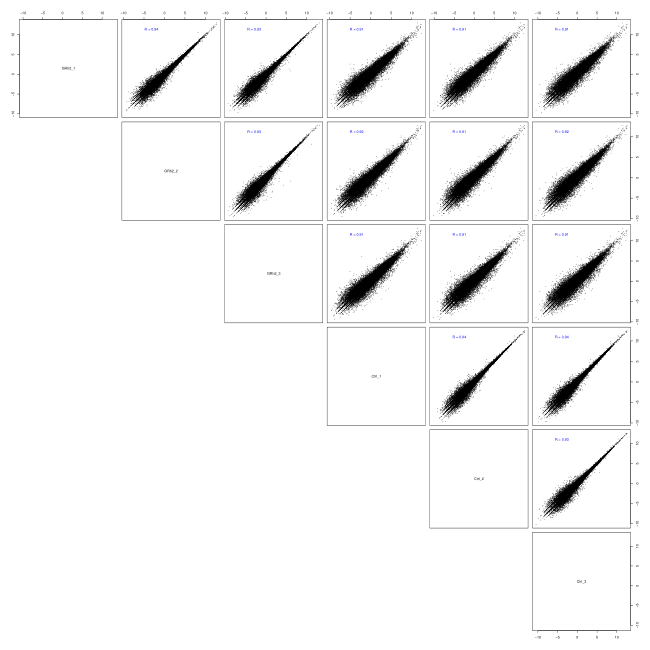

Supplement: Supplementary file 4 [file DataSheet2.zip › supp/DEG/Sample_RPKM_correlation.png]

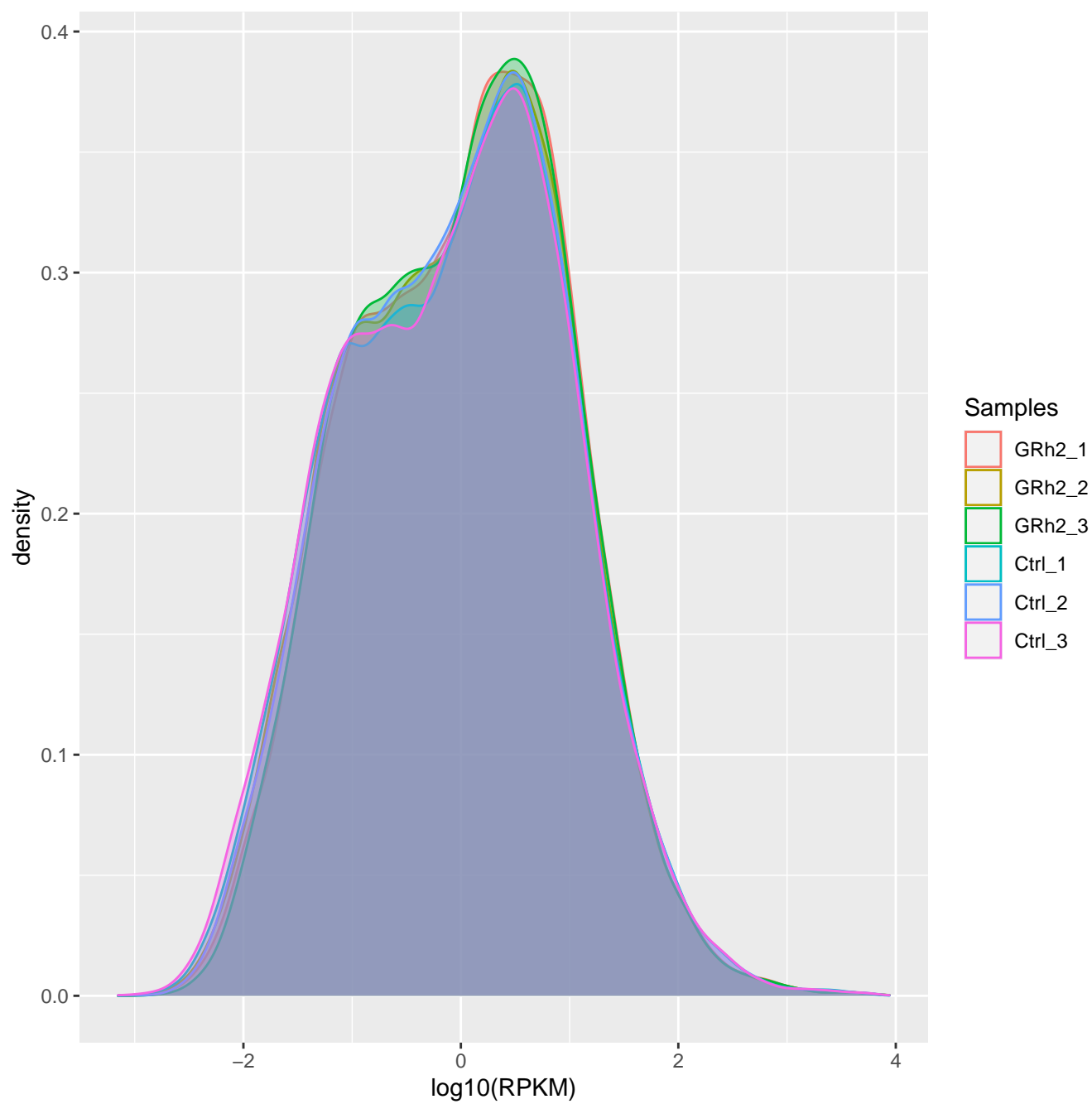

Supplement: Supplementary file 4 [file DataSheet2.zip › supp/DEG/Sample_RPKM_density.pdf]

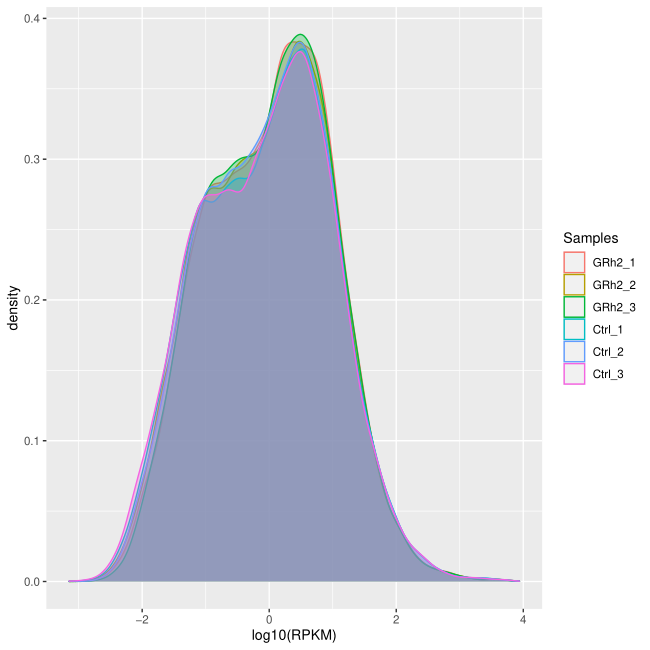

Supplement: Supplementary file 4 [file DataSheet2.zip › supp/DEG/Sample_RPKM_density.png]

Number of genes

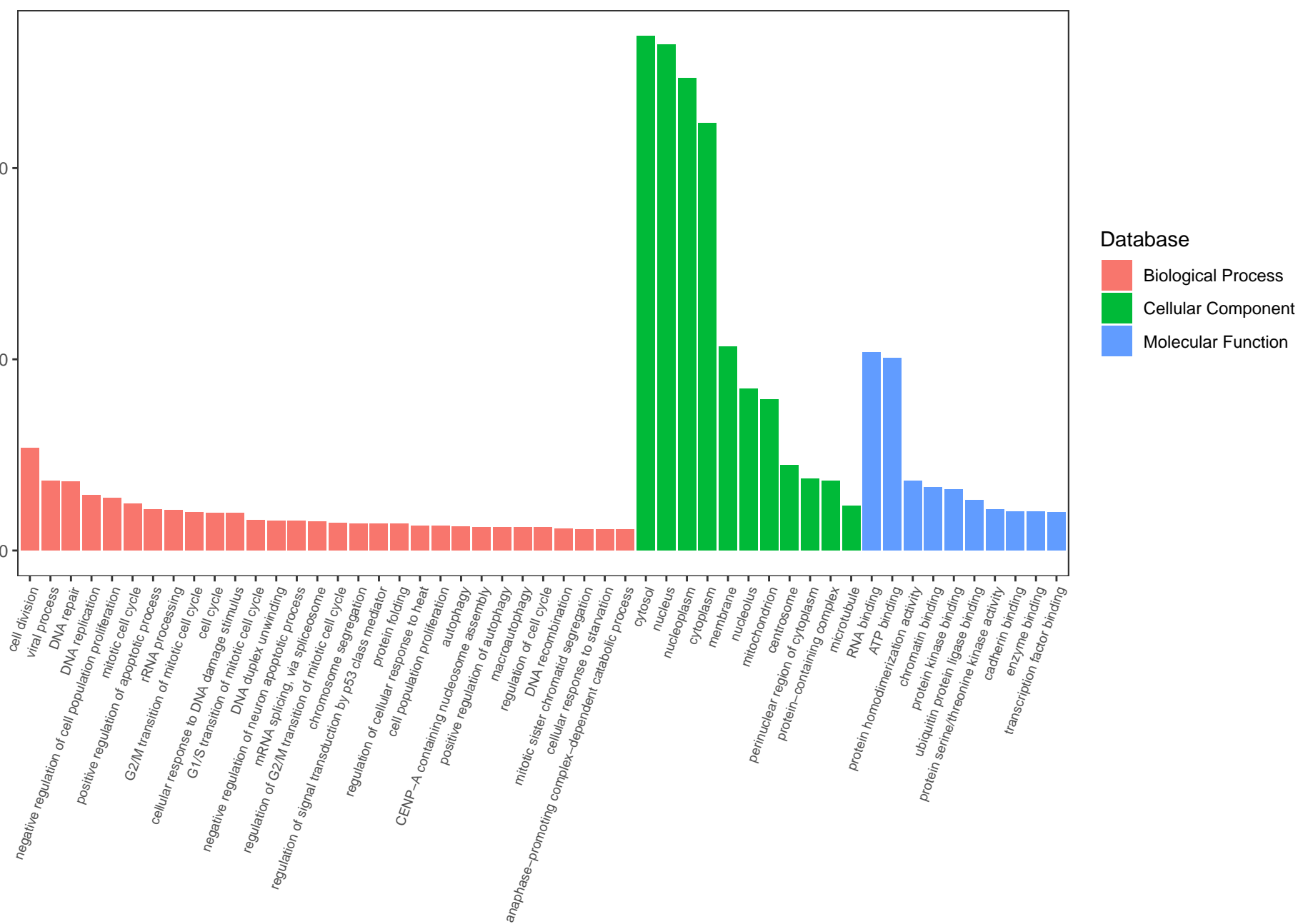

Supplement: Supplementary file 4 [file DataSheet2.zip › supp/GO_KEGG/GRh2___Ctrl/sig_Down_genes_go.enrich.pdf]

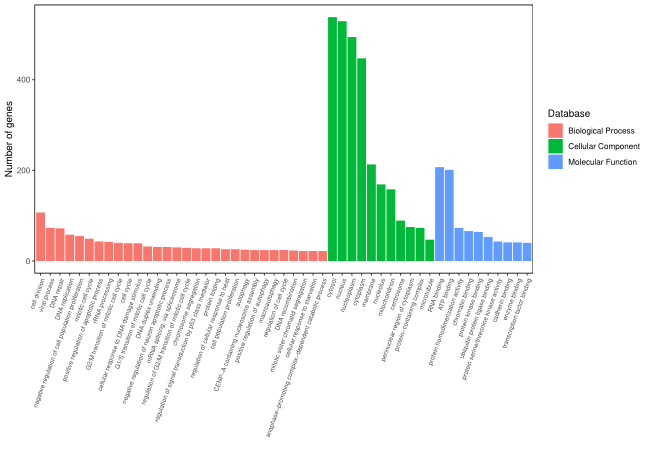

Supplement: Supplementary file 4 [file DataSheet2.zip › supp/GO_KEGG/GRh2___Ctrl/sig_Down_genes_go.enrich.png]

Terms

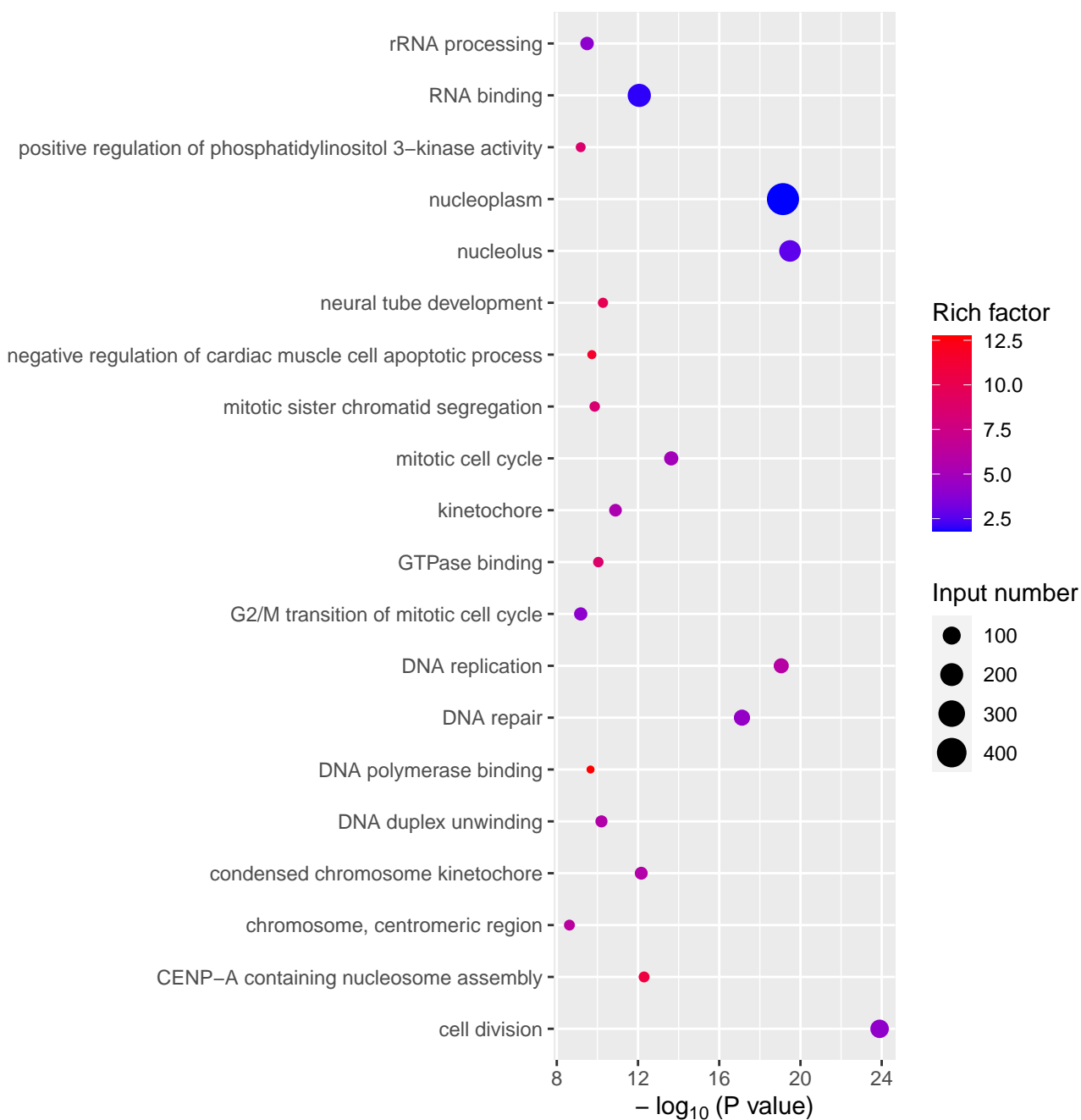

Supplement: Supplementary file 4 [file DataSheet2.zip › supp/GO_KEGG/GRh2___Ctrl/sig_Down_genes_go.pdf]

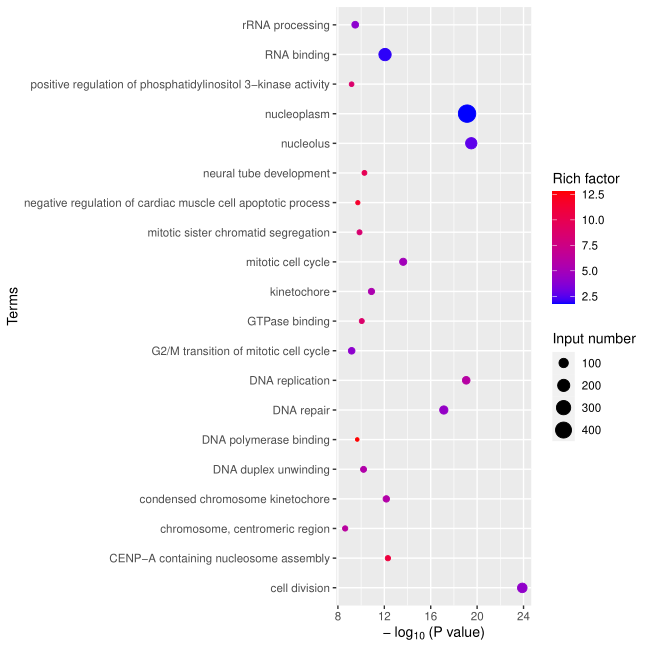

Supplement: Supplementary file 4 [file DataSheet2.zip › supp/GO_KEGG/GRh2___Ctrl/sig_Down_genes_go.png]

Terms

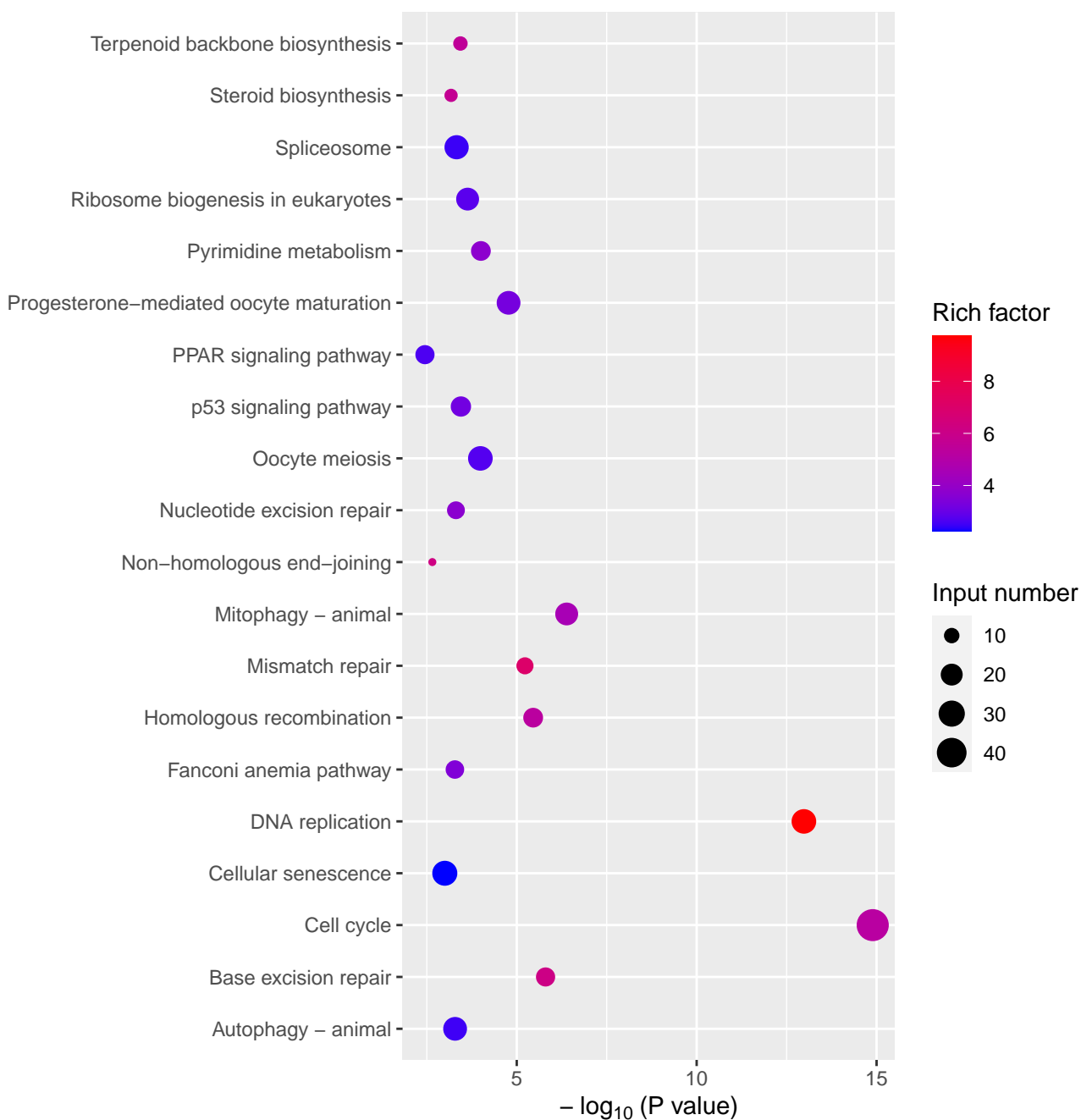

Supplement: Supplementary file 4 [file DataSheet2.zip › supp/GO_KEGG/GRh2___Ctrl/sig_Down_genes_kegg.pdf]

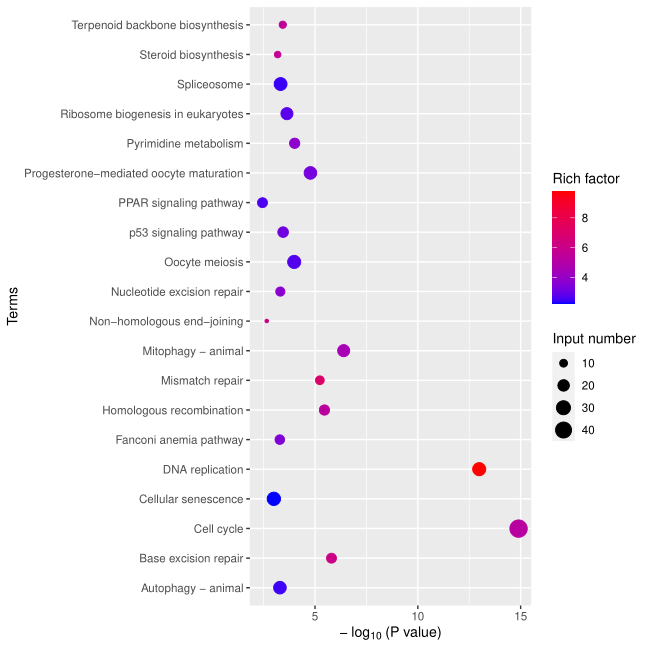

Supplement: Supplementary file 4 [file DataSheet2.zip › supp/GO_KEGG/GRh2___Ctrl/sig_Down_genes_kegg.png]

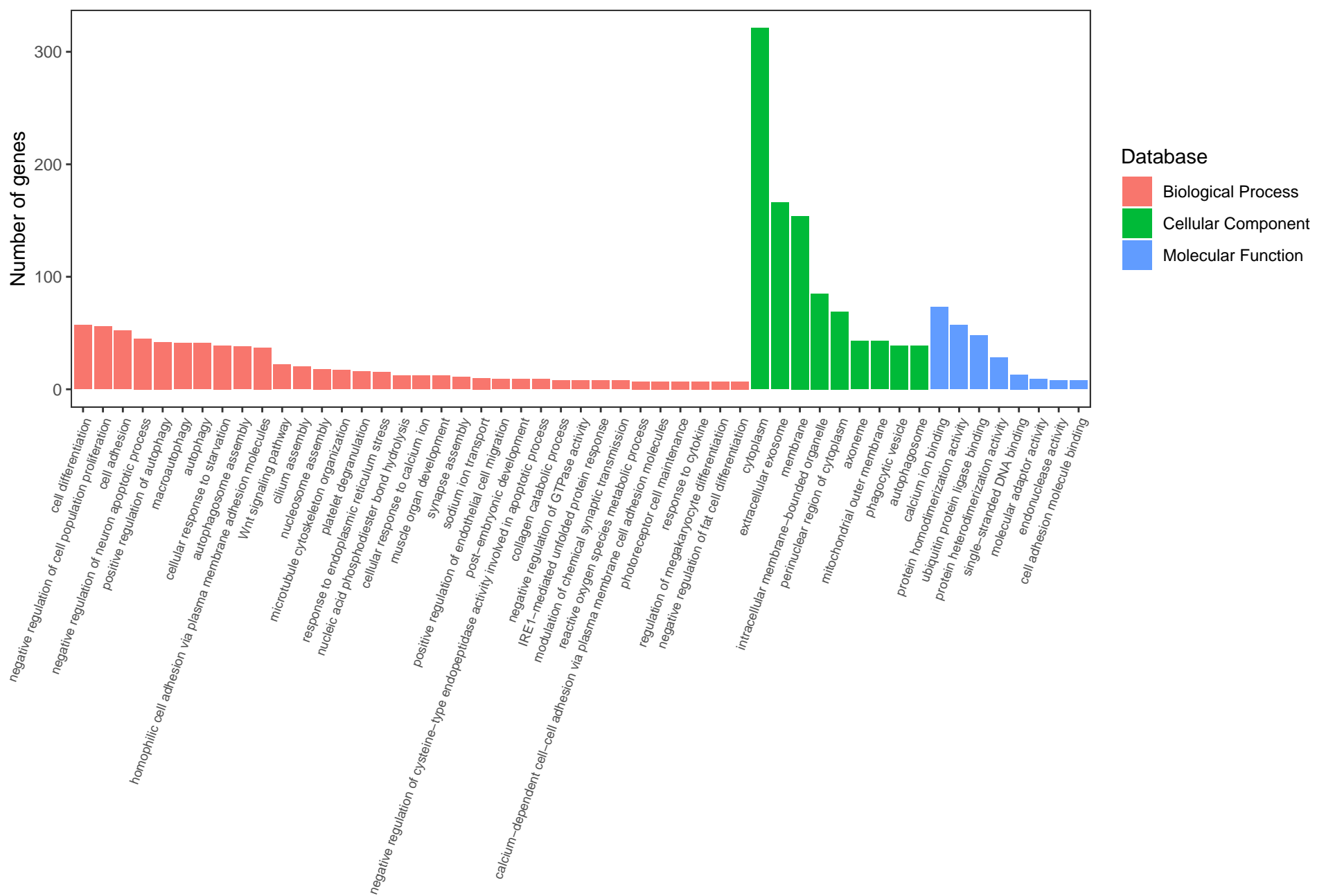

Supplement: Supplementary file 4 [file DataSheet2.zip › supp/GO_KEGG/GRh2___Ctrl/sig_UP_genes_go.enrich.pdf]

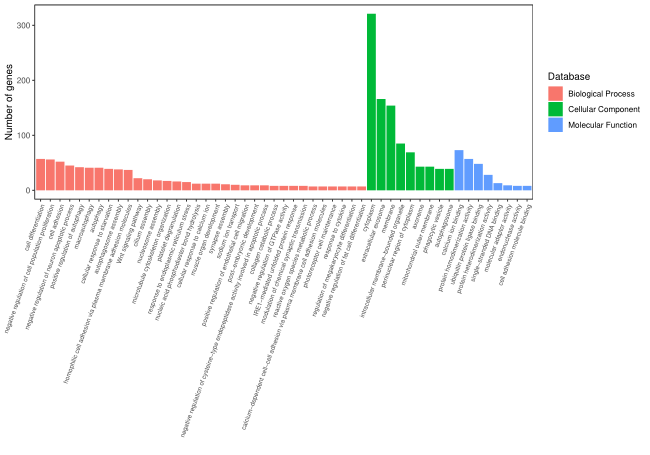

Supplement: Supplementary file 4 [file DataSheet2.zip › supp/GO_KEGG/GRh2___Ctrl/sig_UP_genes_go.enrich.png]

Terms

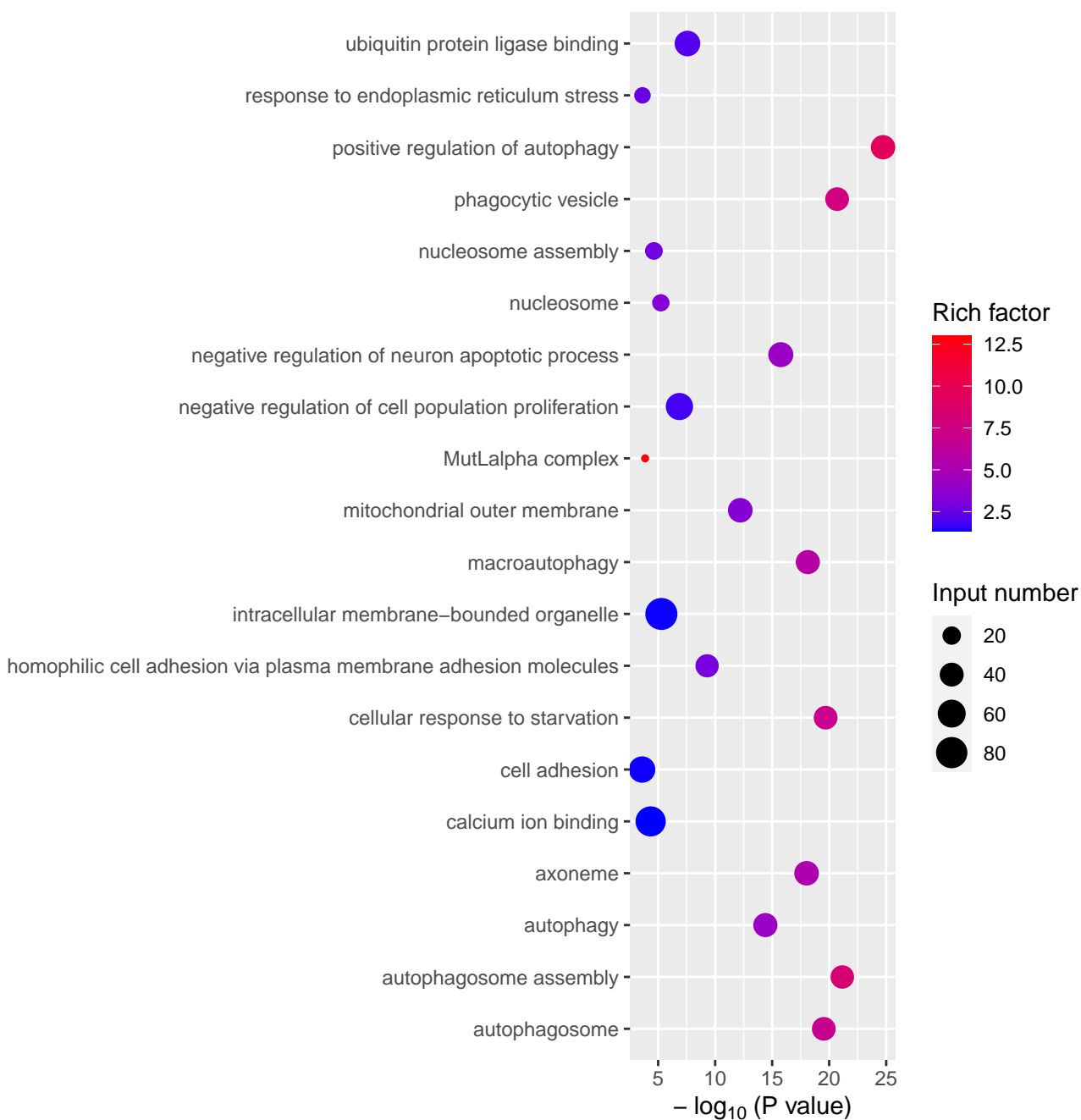

Supplement: Supplementary file 4 [file DataSheet2.zip › supp/GO_KEGG/GRh2___Ctrl/sig_UP_genes_go.pdf]

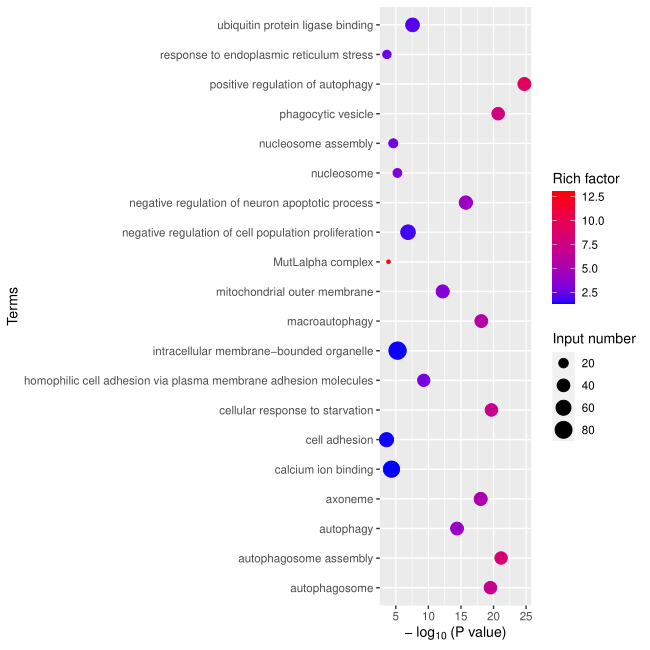

Supplement: Supplementary file 4 [file DataSheet2.zip › supp/GO_KEGG/GRh2___Ctrl/sig_UP_genes_go.png]

Terms

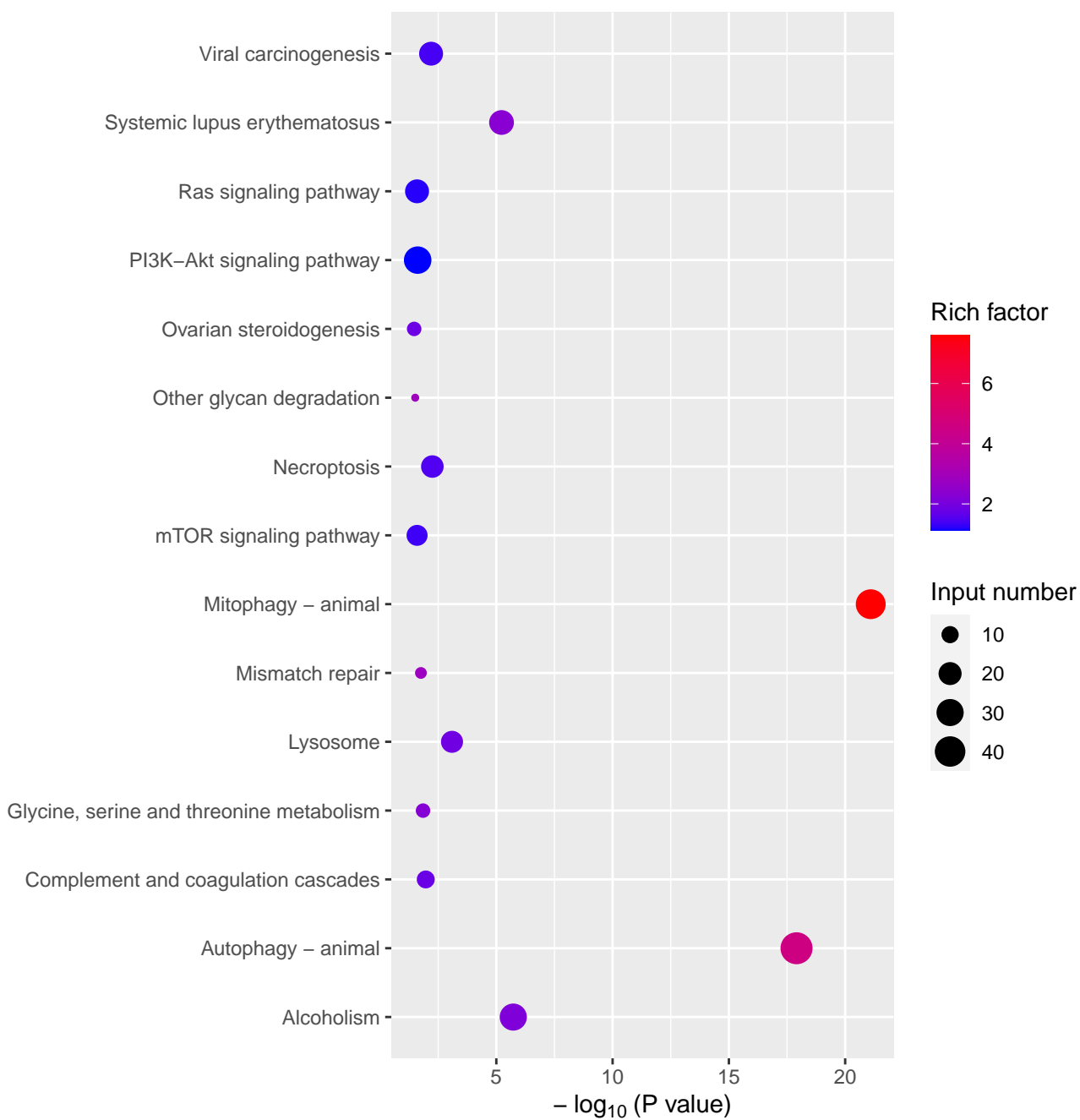

Supplement: Supplementary file 4 [file DataSheet2.zip › supp/GO_KEGG/GRh2___Ctrl/sig_UP_genes_kegg.pdf]

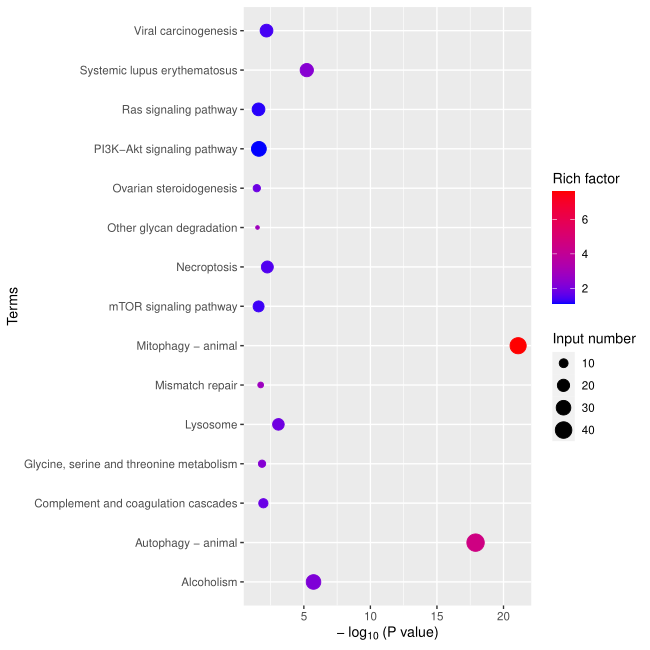

Supplement: Supplementary file 4 [file DataSheet2.zip › supp/GO_KEGG/GRh2___Ctrl/sig_UP_genes_kegg.png]

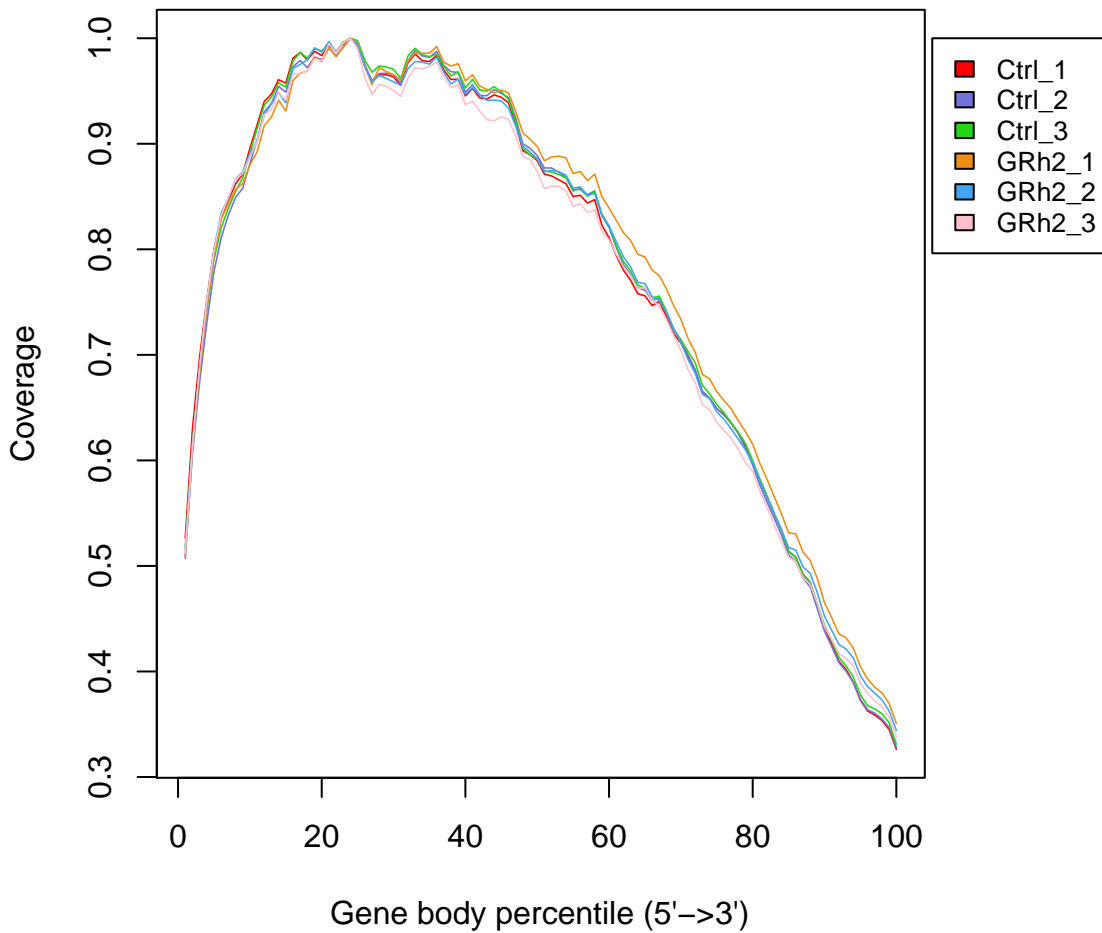

Supplement: Supplementary file 4 [file DataSheet2.zip › supp/mapping/geneBodyCoverage.pdf]

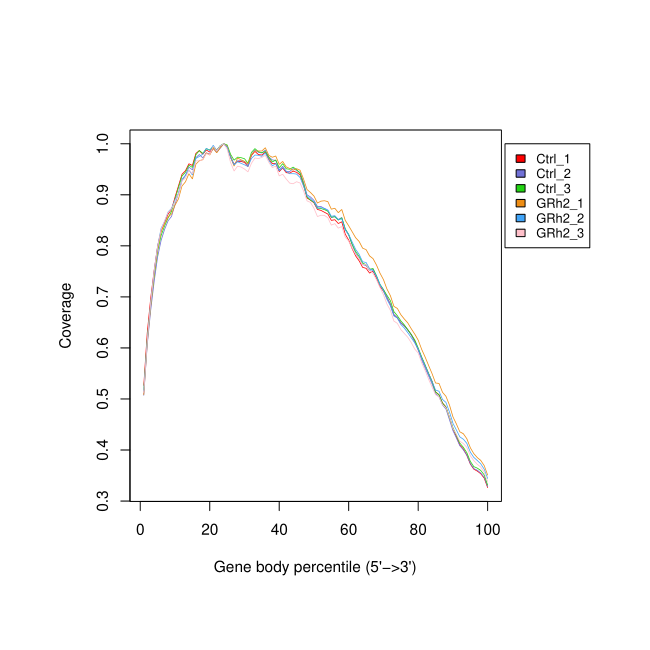

Supplement: Supplementary file 4 [file DataSheet2.zip › supp/mapping/geneBodyCoverage.png]

Reads distribution across genomic regions

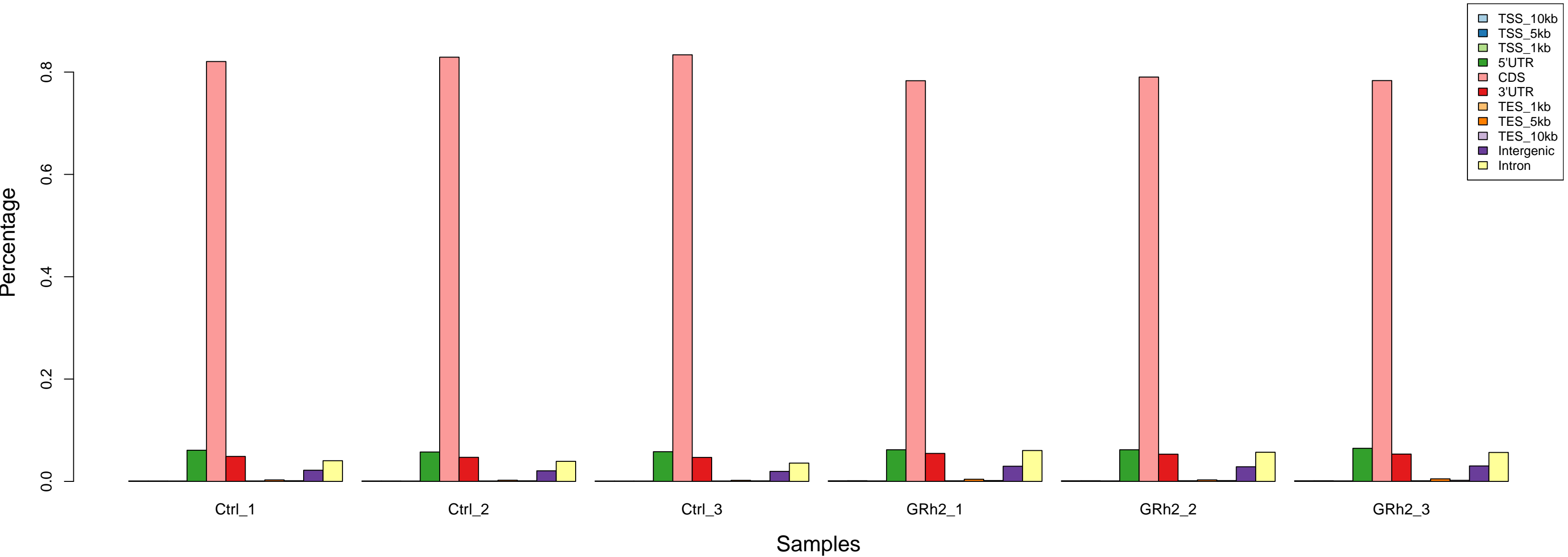

Supplement: Supplementary file 4 [file DataSheet2.zip › supp/mapping/read_distribution.pdf]

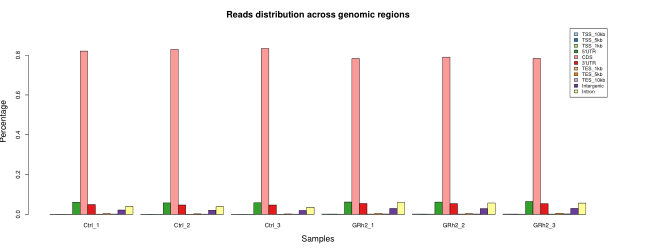

Supplement: Supplementary file 4 [file DataSheet2.zip › supp/mapping/read_distribution.png]

Percentage of Bases across All Bases (Ctrl\_1)

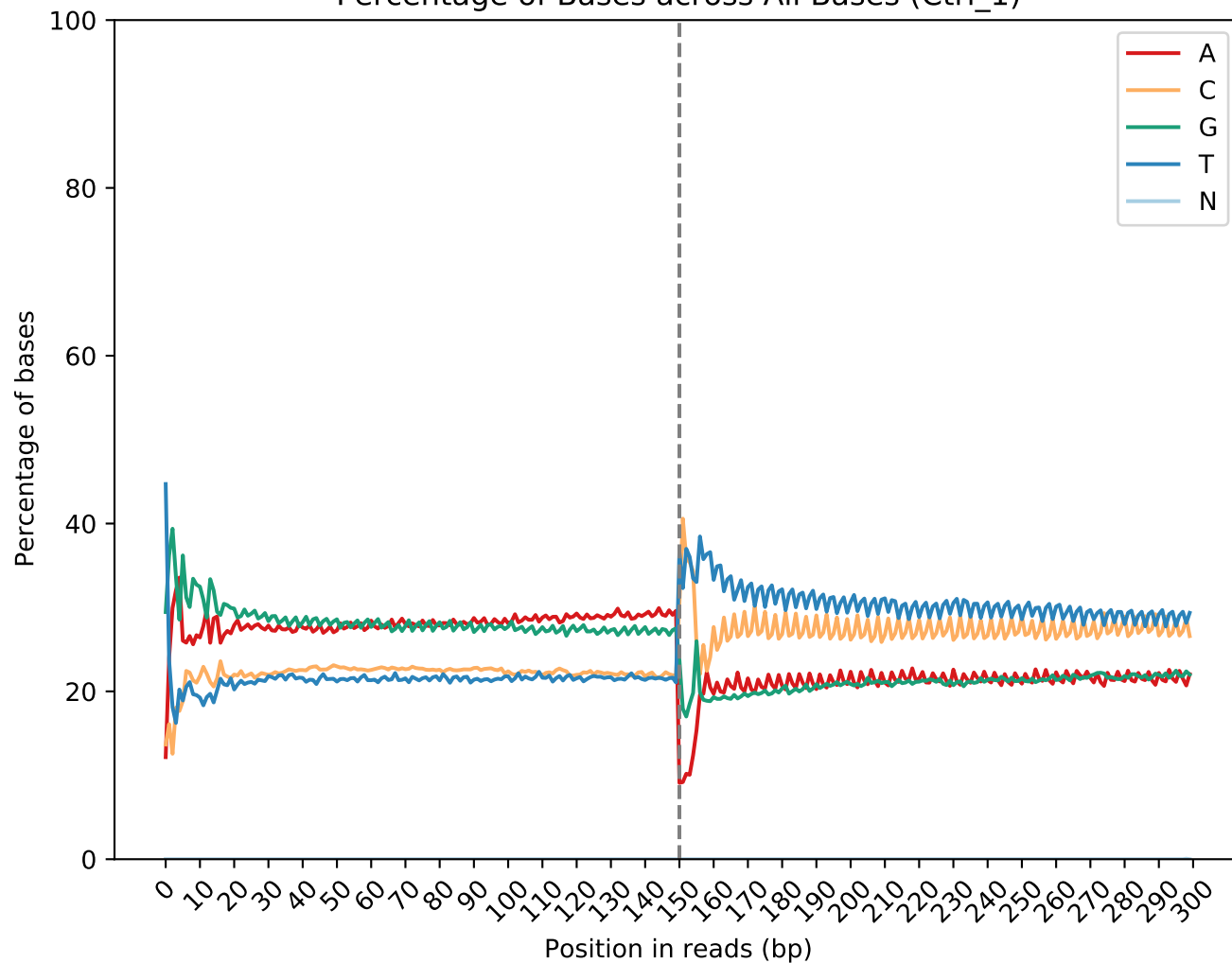

Supplement: Supplementary file 4 [file DataSheet2.zip › supp/QC/clean/Ctrl_1/Ctrl_1.bases.content.pdf]

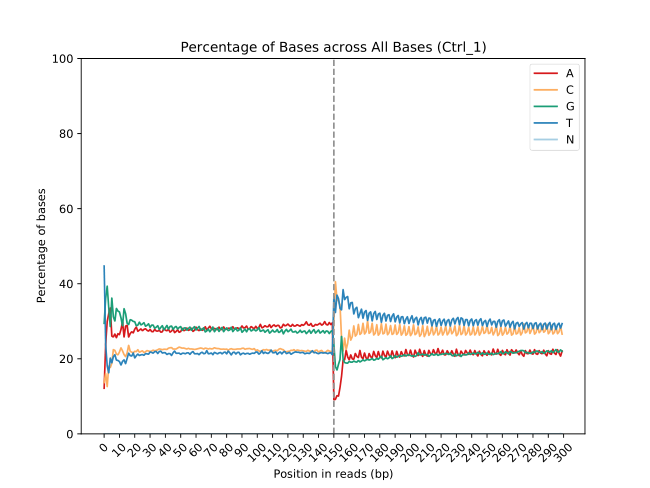

Supplement: Supplementary file 4 [file DataSheet2.zip › supp/QC/clean/Ctrl_1/Ctrl_1.bases.content.png]

Base Quality across All Bases (Ctrl\_1)

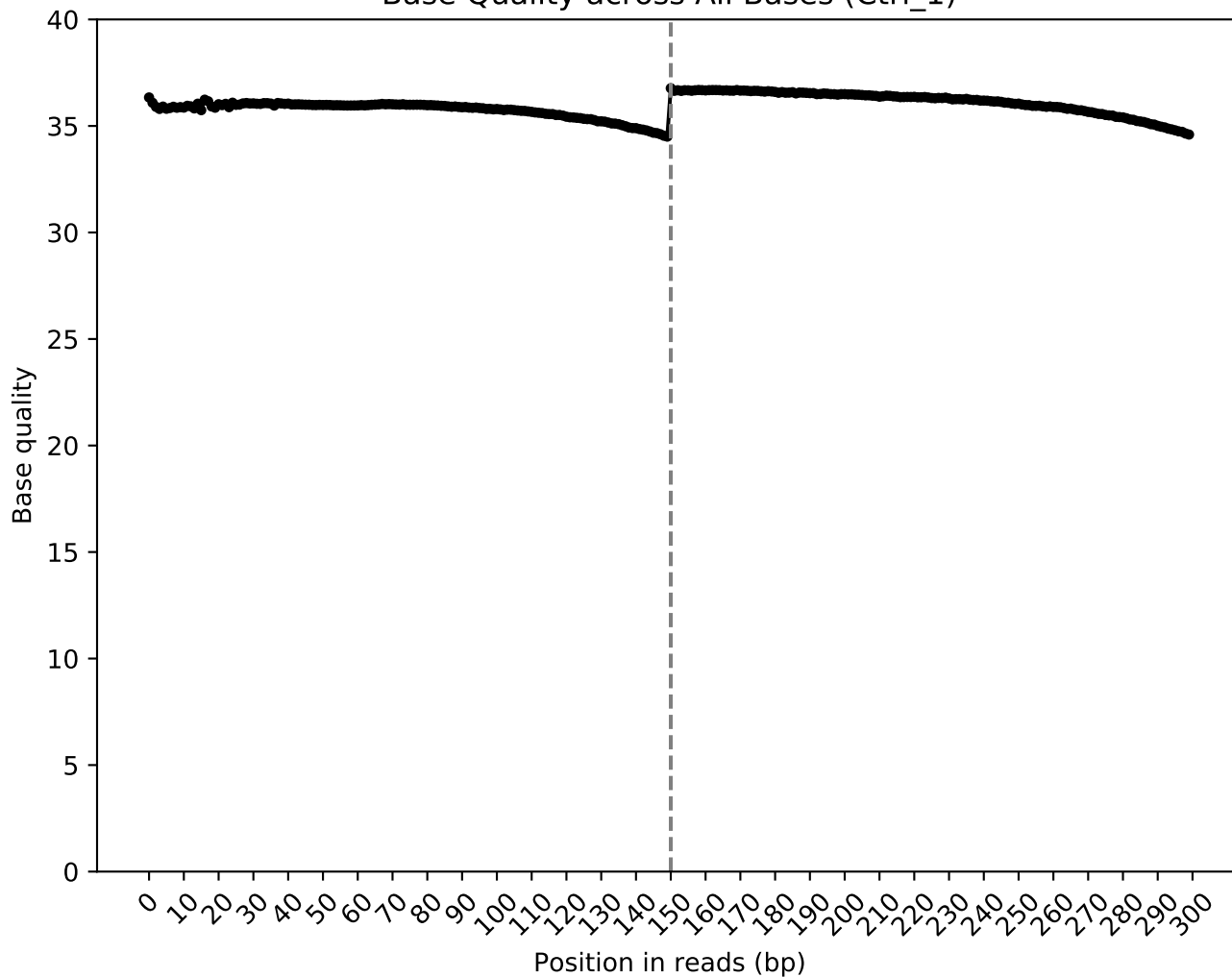

Supplement: Supplementary file 4 [file DataSheet2.zip › supp/QC/clean/Ctrl_1/Ctrl_1.bases.quality.pdf]

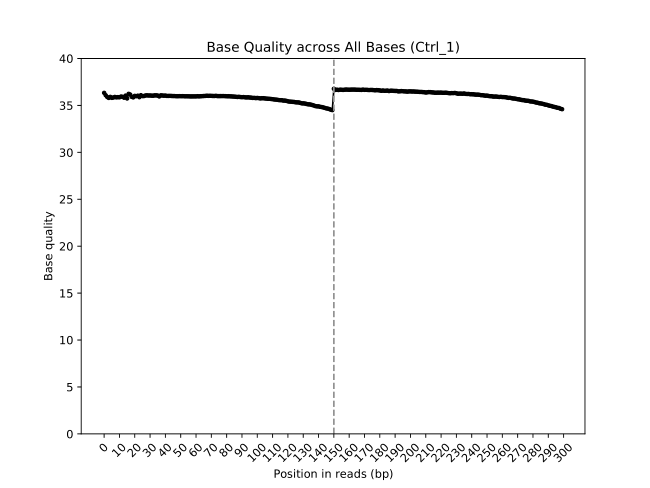

Supplement: Supplementary file 4 [file DataSheet2.zip › supp/QC/clean/Ctrl_1/Ctrl_1.bases.quality.png]

GC Distribution over all reads (Ctrl\_1)

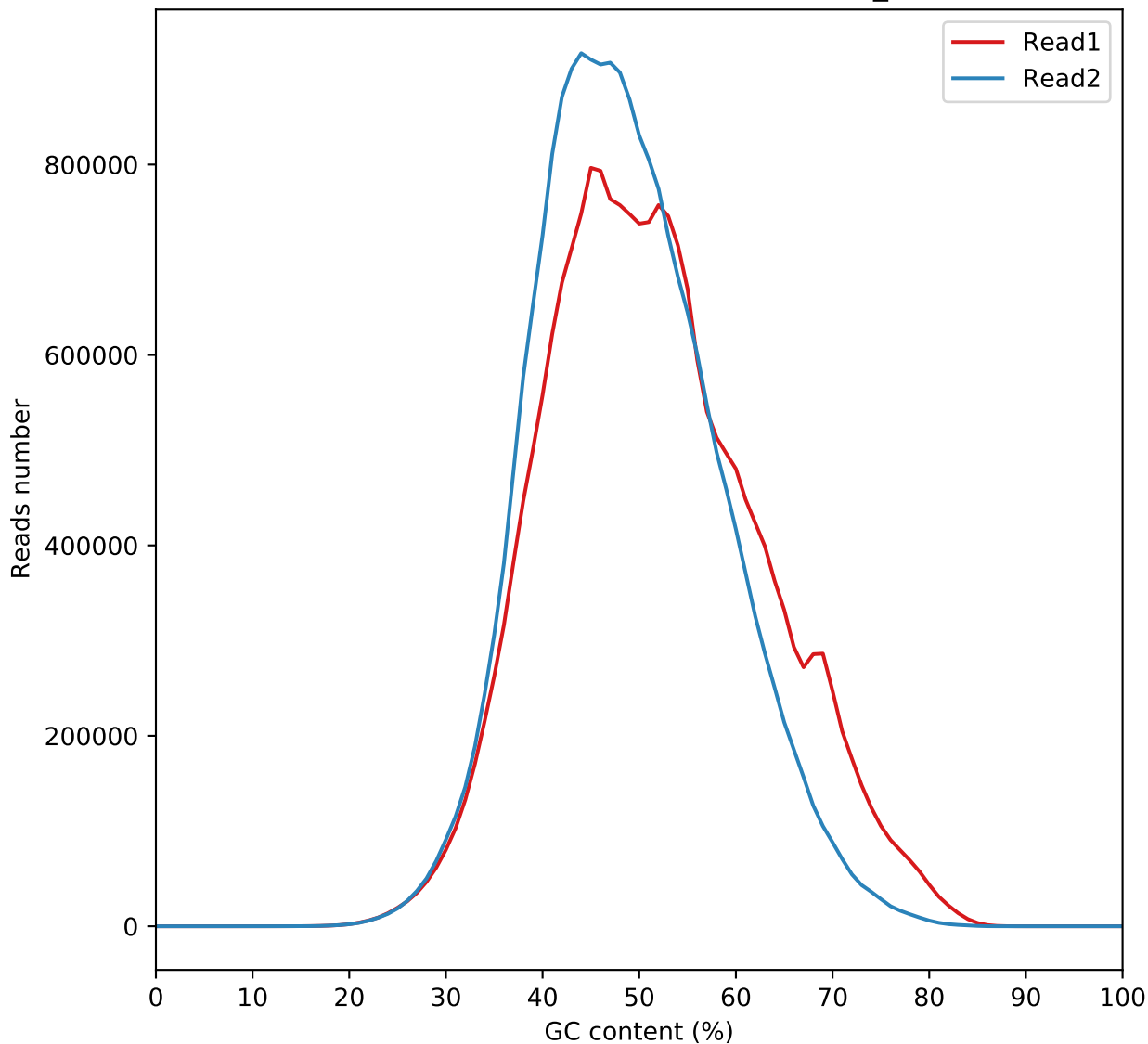

Supplement: Supplementary file 4 [file DataSheet2.zip › supp/QC/clean/Ctrl_1/Ctrl_1.GC.distribution.pdf]

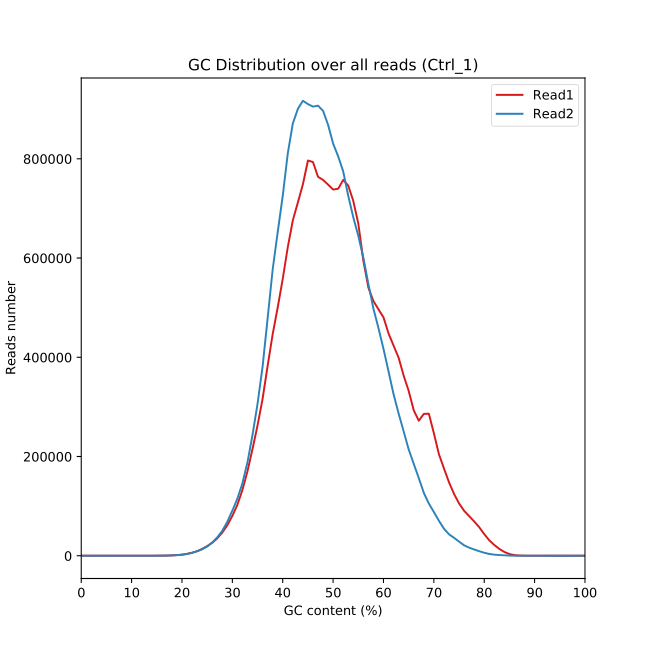

Supplement: Supplementary file 4 [file DataSheet2.zip › supp/QC/clean/Ctrl_1/Ctrl_1.GC.distribution.png]

Reads Filtering Result (Ctrl\_1)

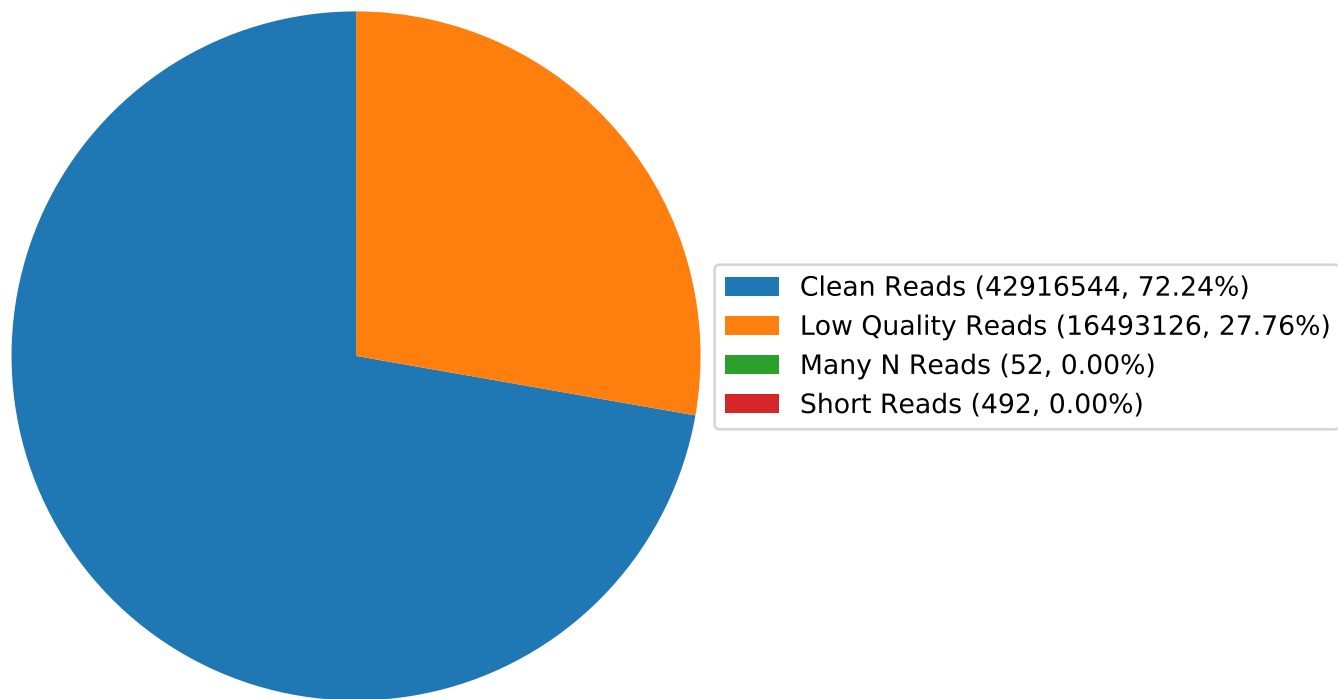

Supplement: Supplementary file 4 [file DataSheet2.zip › supp/QC/clean/Ctrl_1/Ctrl_1.reads.filter.pdf]

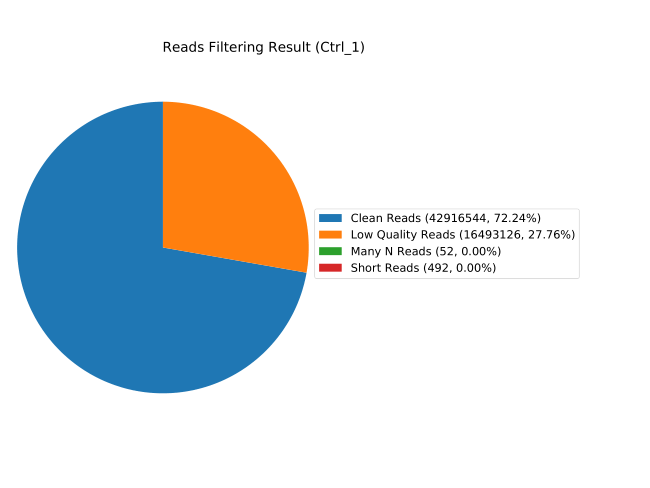

Supplement: Supplementary file 4 [file DataSheet2.zip › supp/QC/clean/Ctrl_1/Ctrl_1.reads.filter.png]

Percentage of Bases across All Bases (Ctrl\_2)

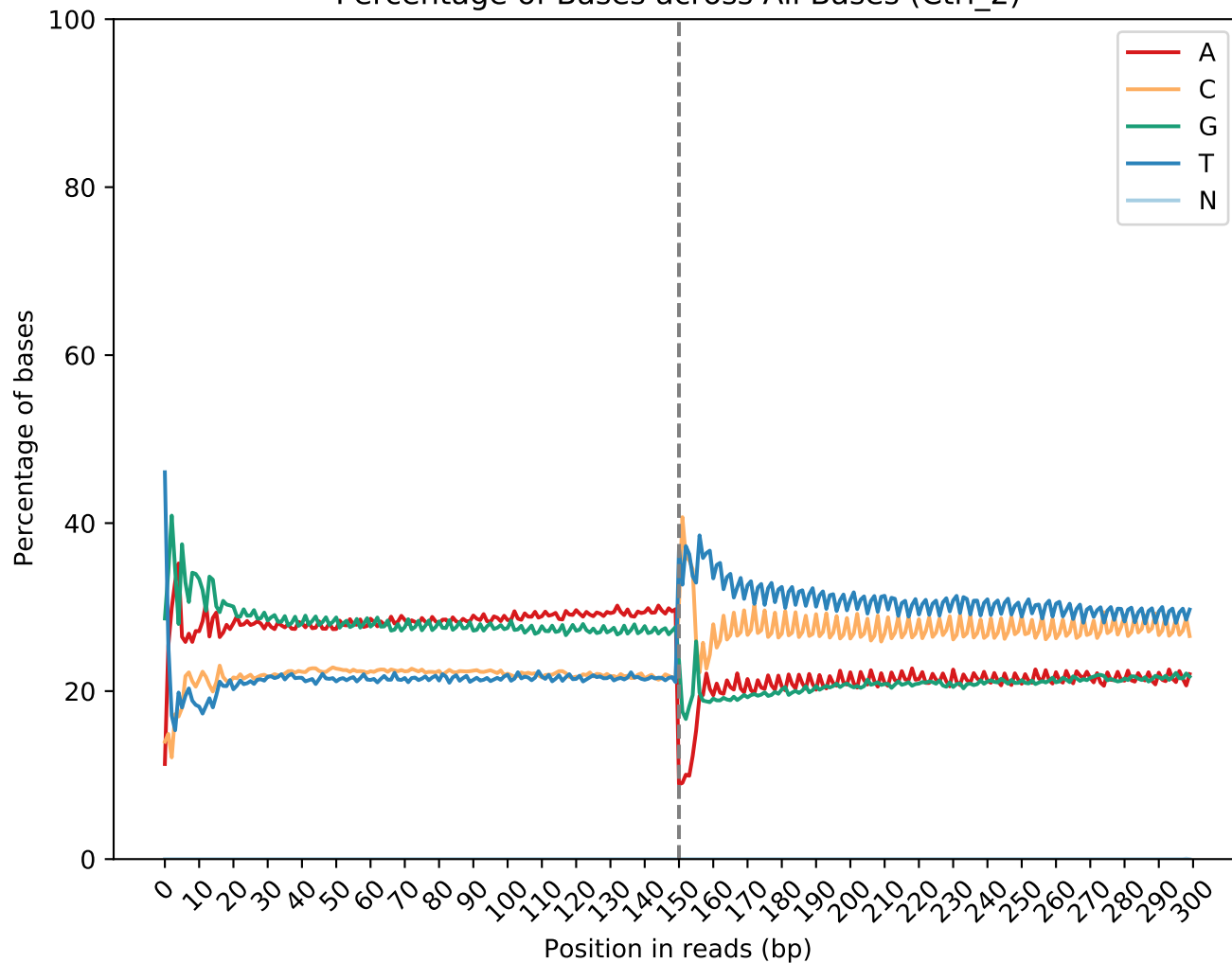

Supplement: Supplementary file 4 [file DataSheet2.zip › supp/QC/clean/Ctrl_2/Ctrl_2.bases.content.pdf]

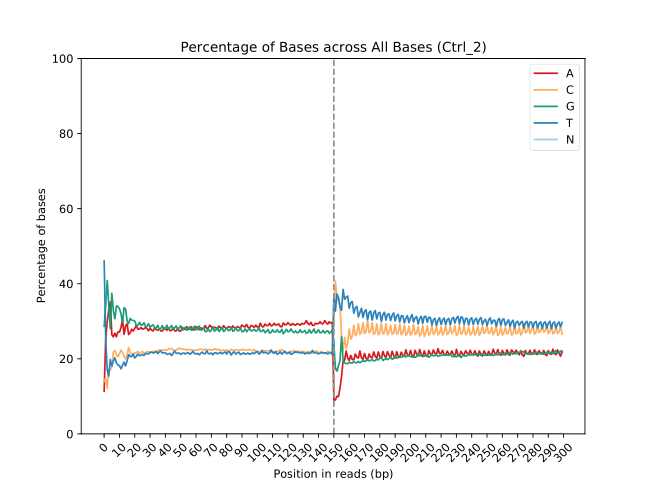

Supplement: Supplementary file 4 [file DataSheet2.zip › supp/QC/clean/Ctrl_2/Ctrl_2.bases.content.png]

Base Quality across All Bases (Ctrl\_2)

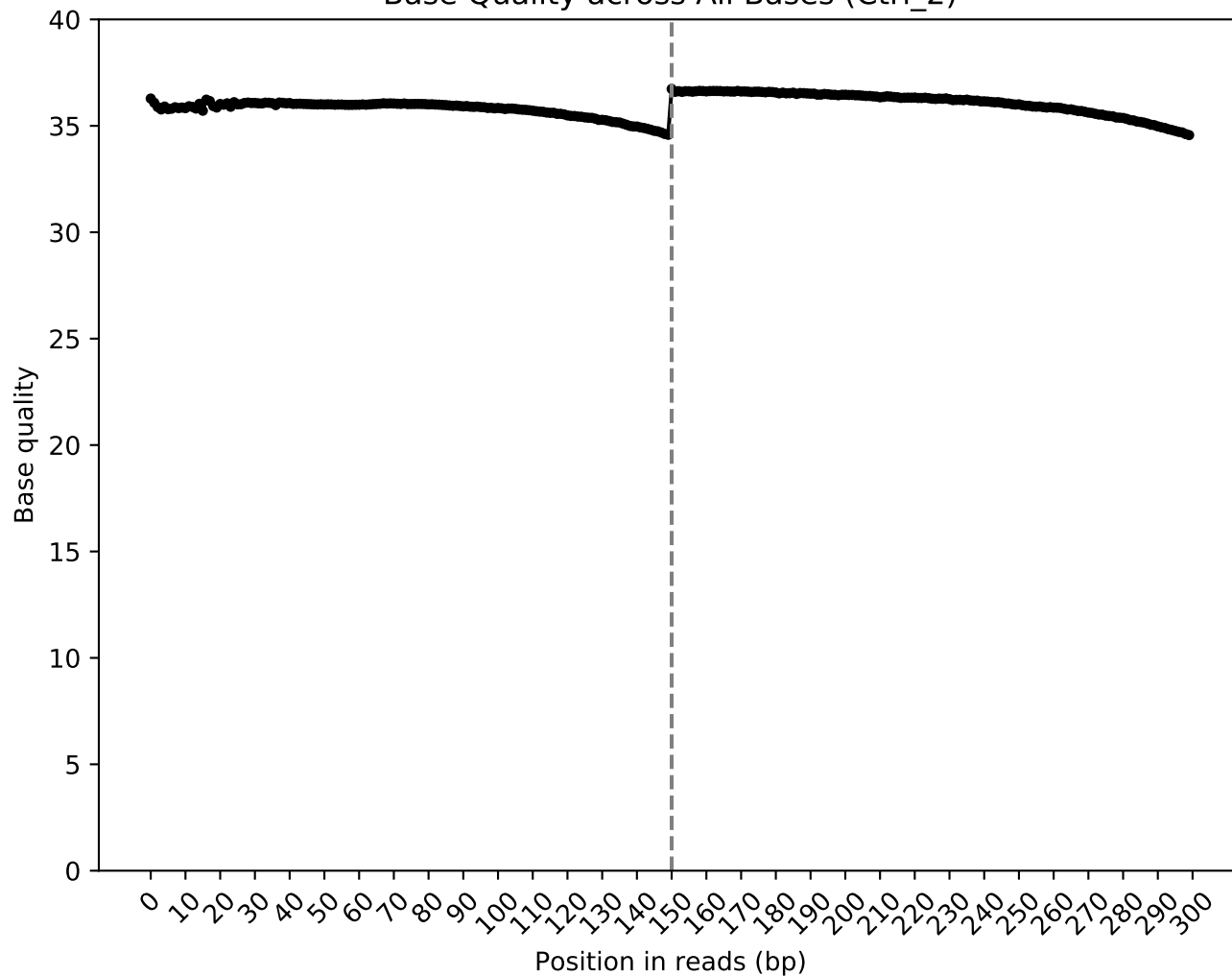

Supplement: Supplementary file 4 [file DataSheet2.zip › supp/QC/clean/Ctrl_2/Ctrl_2.bases.quality.pdf]

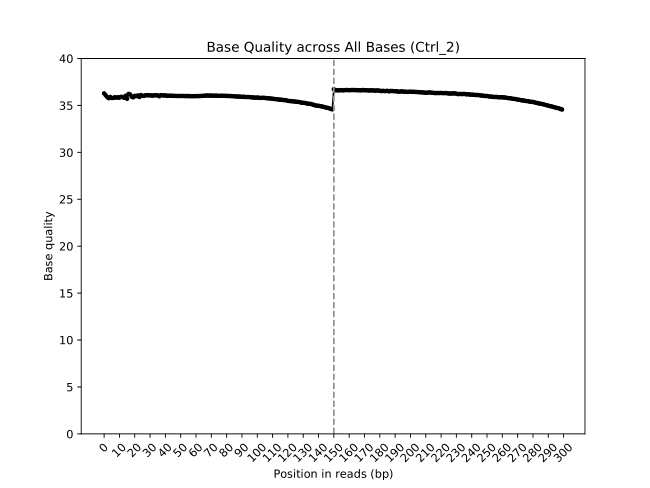

Supplement: Supplementary file 4 [file DataSheet2.zip › supp/QC/clean/Ctrl_2/Ctrl_2.bases.quality.png]

GC Distribution over all reads (Ctrl\_2)

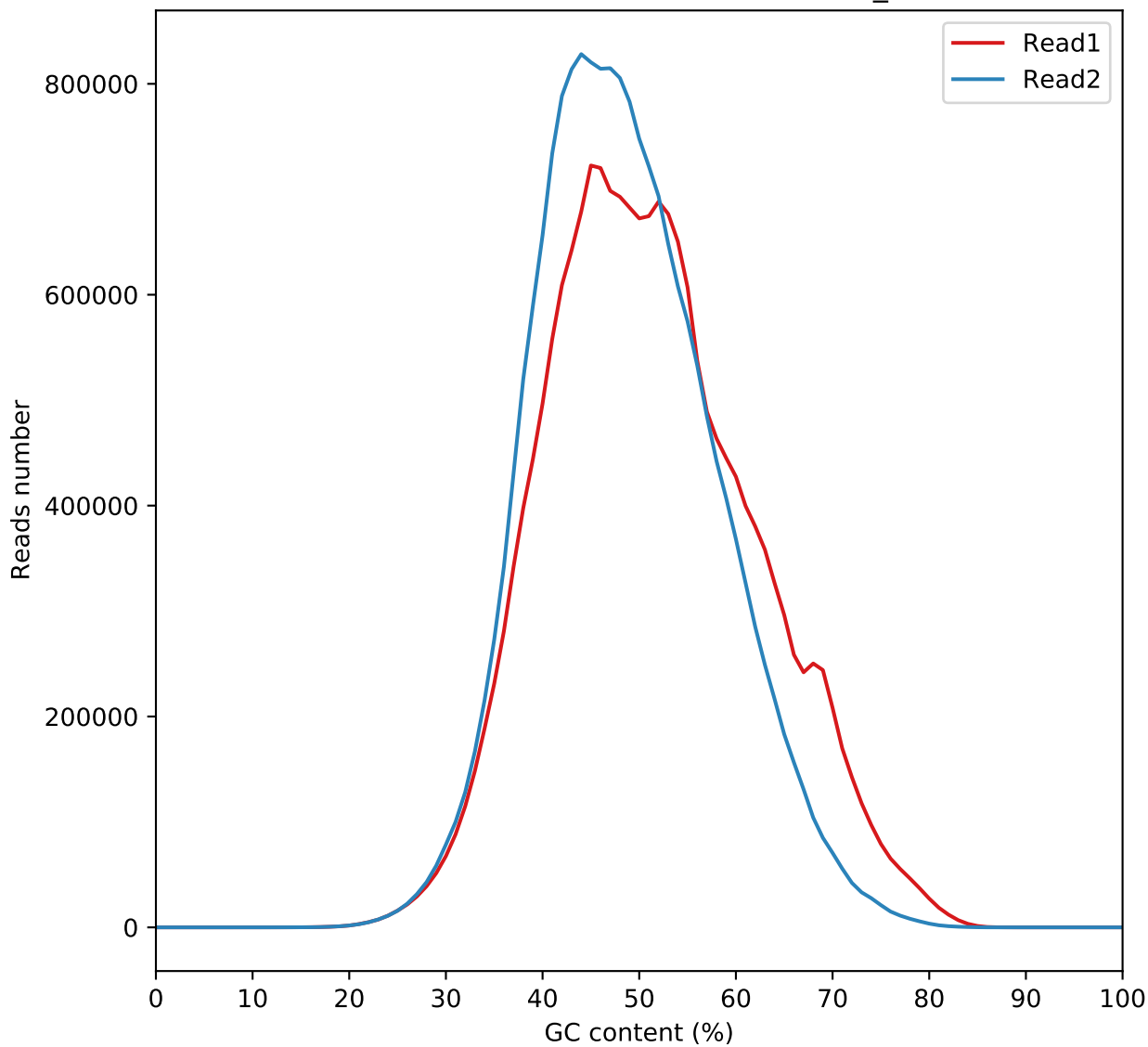

Supplement: Supplementary file 4 [file DataSheet2.zip › supp/QC/clean/Ctrl_2/Ctrl_2.GC.distribution.pdf]

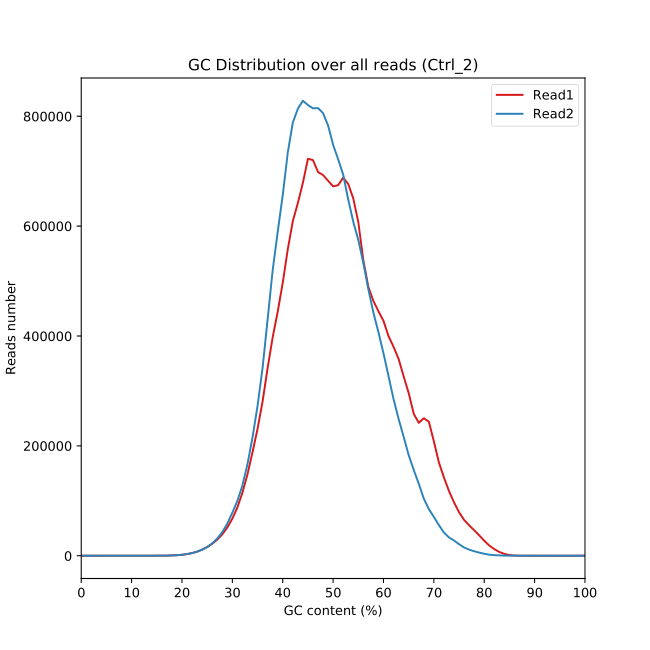

Supplement: Supplementary file 4 [file DataSheet2.zip › supp/QC/clean/Ctrl_2/Ctrl_2.GC.distribution.png]

Reads Filtering Result (Ctrl\_2)

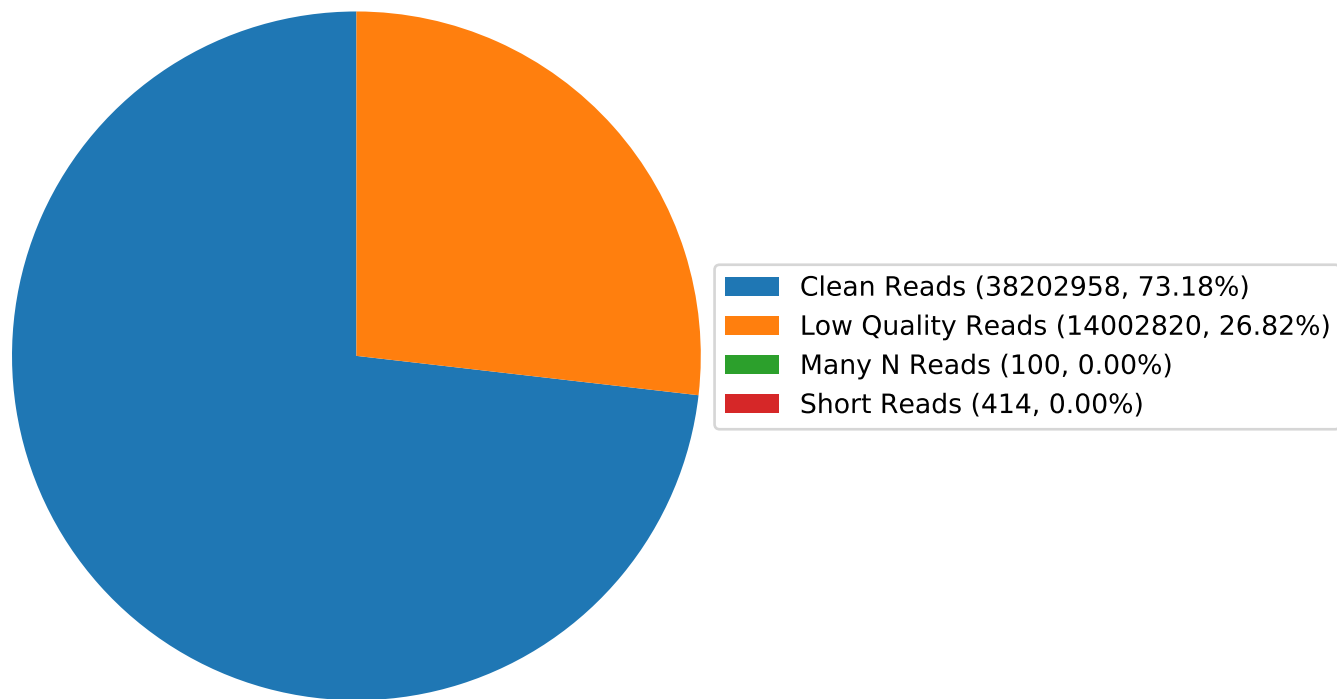

Supplement: Supplementary file 4 [file DataSheet2.zip › supp/QC/clean/Ctrl_2/Ctrl_2.reads.filter.pdf]

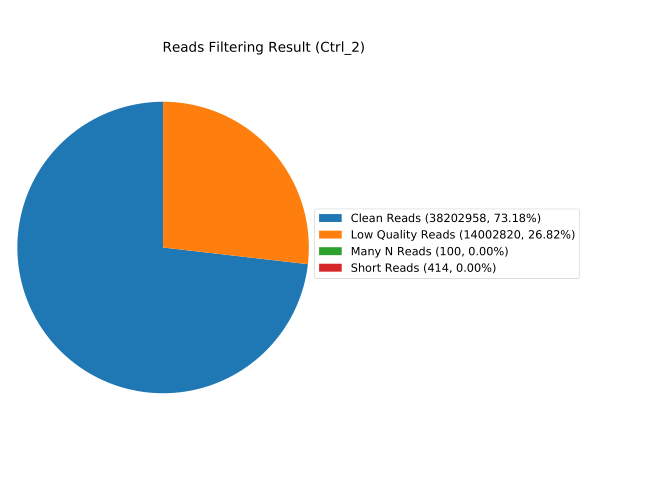

Supplement: Supplementary file 4 [file DataSheet2.zip › supp/QC/clean/Ctrl_2/Ctrl_2.reads.filter.png]

Percentage of Bases across All Bases (Ctrl\_3)

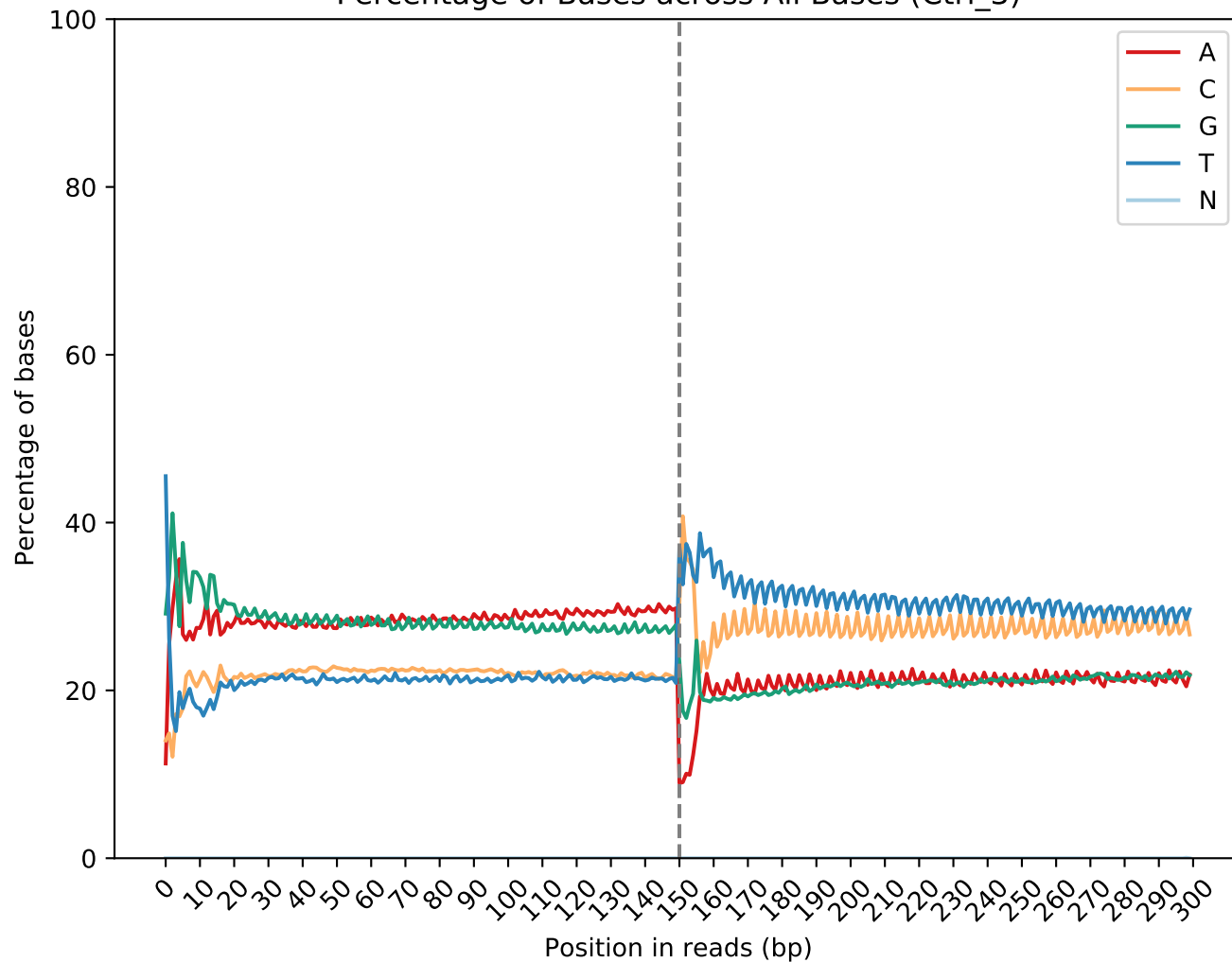

Supplement: Supplementary file 4 [file DataSheet2.zip › supp/QC/clean/Ctrl_3/Ctrl_3.bases.content.pdf]

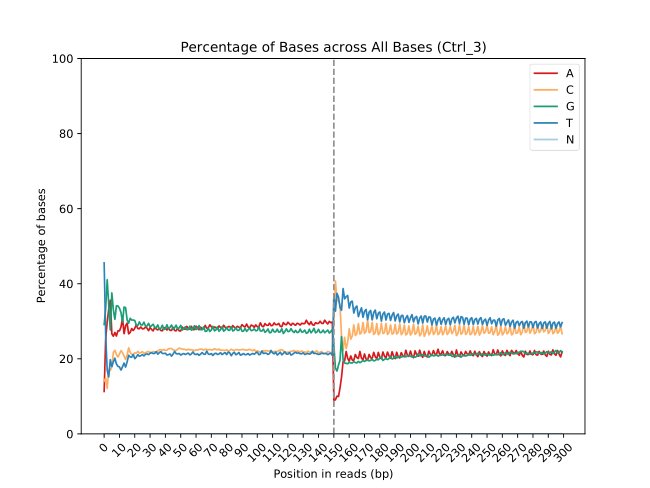

Supplement: Supplementary file 4 [file DataSheet2.zip › supp/QC/clean/Ctrl_3/Ctrl_3.bases.content.png]

Base Quality across All Bases (Ctrl\_3)

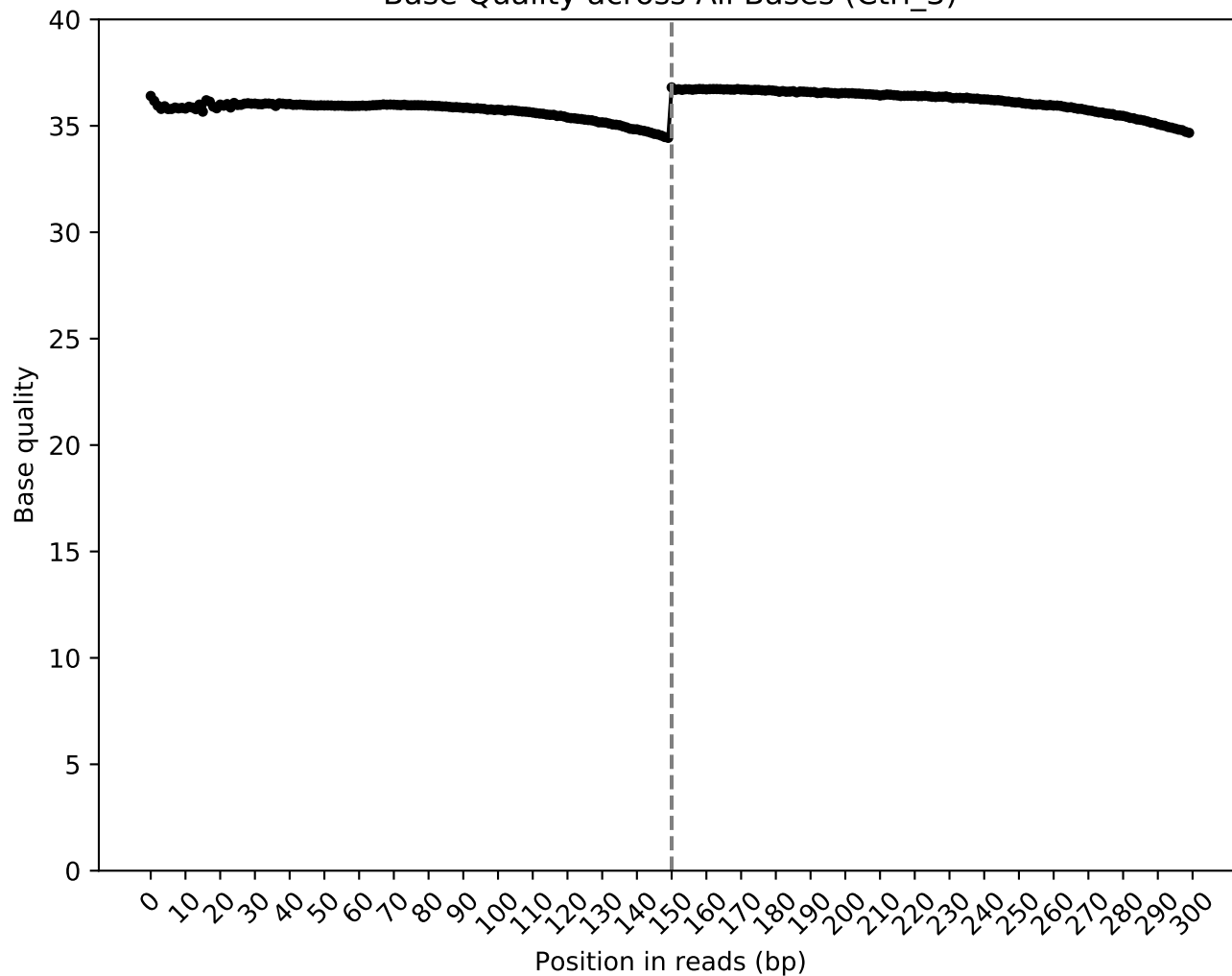

Supplement: Supplementary file 4 [file DataSheet2.zip › supp/QC/clean/Ctrl_3/Ctrl_3.bases.quality.pdf]

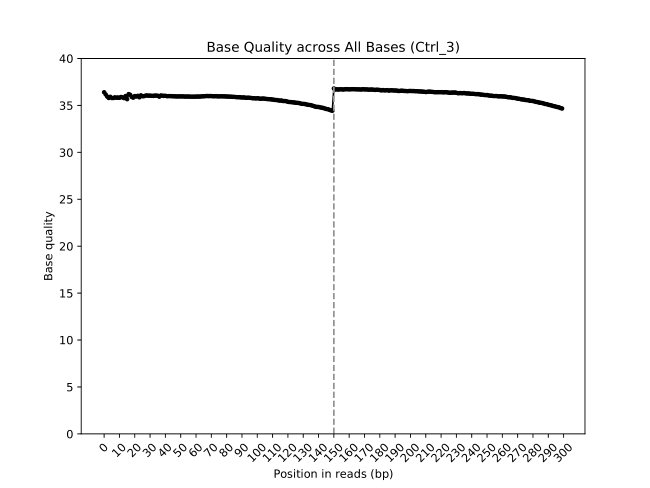

Supplement: Supplementary file 4 [file DataSheet2.zip › supp/QC/clean/Ctrl_3/Ctrl_3.bases.quality.png]

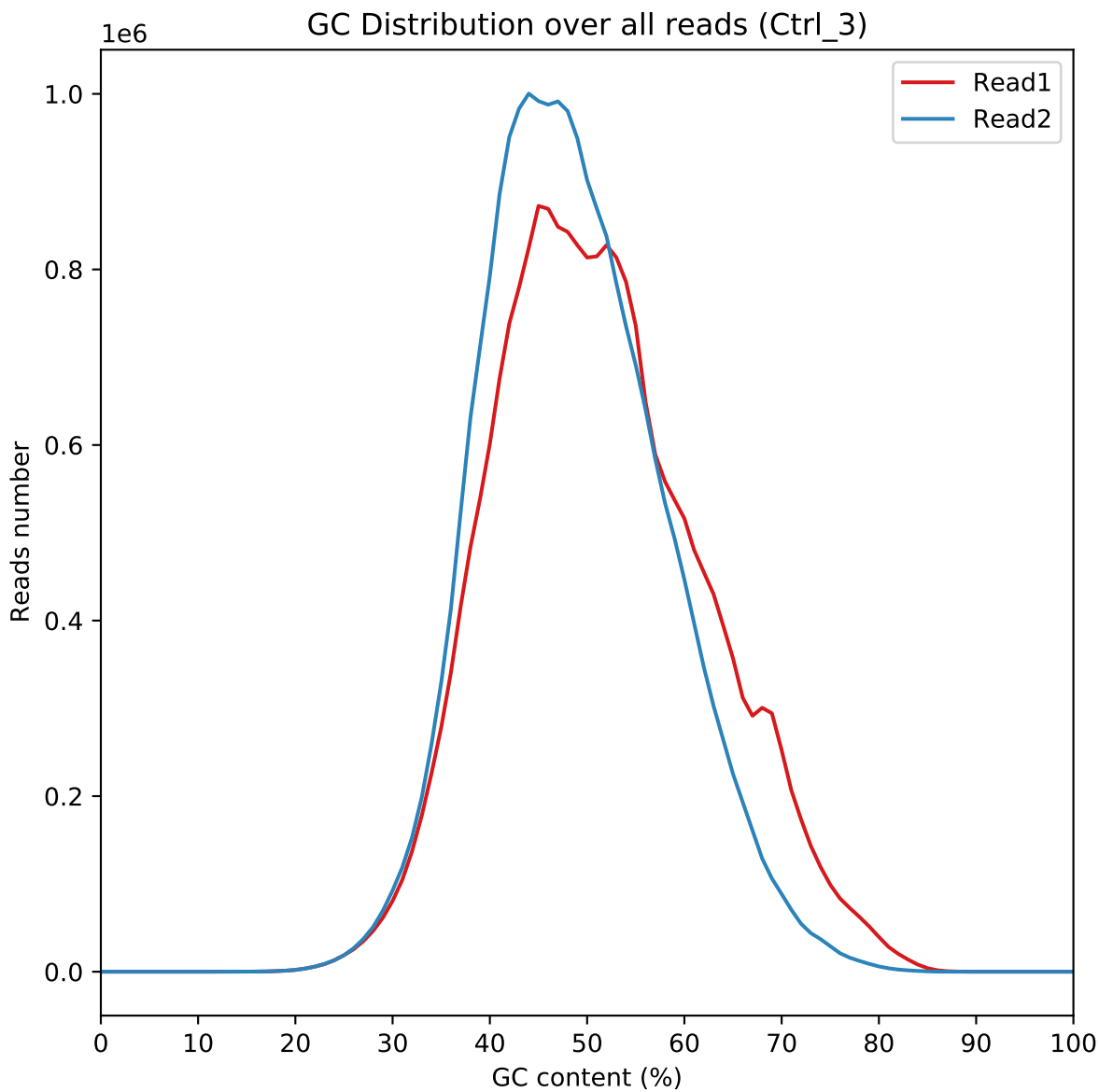

Supplement: Supplementary file 4 [file DataSheet2.zip › supp/QC/clean/Ctrl_3/Ctrl_3.GC.distribution.pdf]

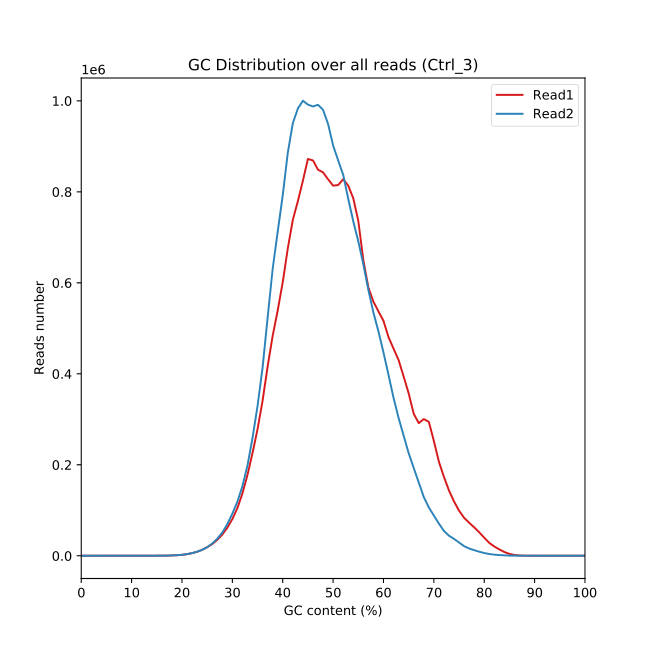

Supplement: Supplementary file 4 [file DataSheet2.zip › supp/QC/clean/Ctrl_3/Ctrl_3.GC.distribution.png]

## Reads Filtering Result (Ctrl\_3)

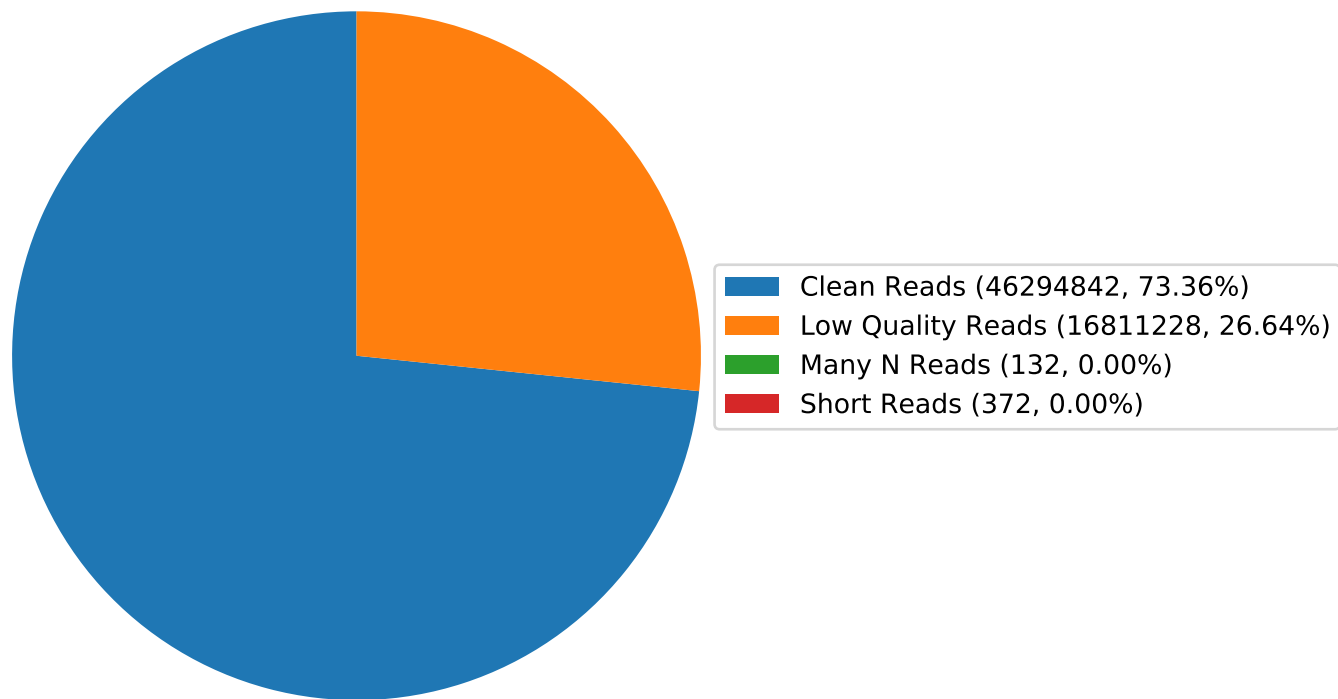

Supplement: Supplementary file 4 [file DataSheet2.zip › supp/QC/clean/Ctrl_3/Ctrl_3.reads.filter.pdf]

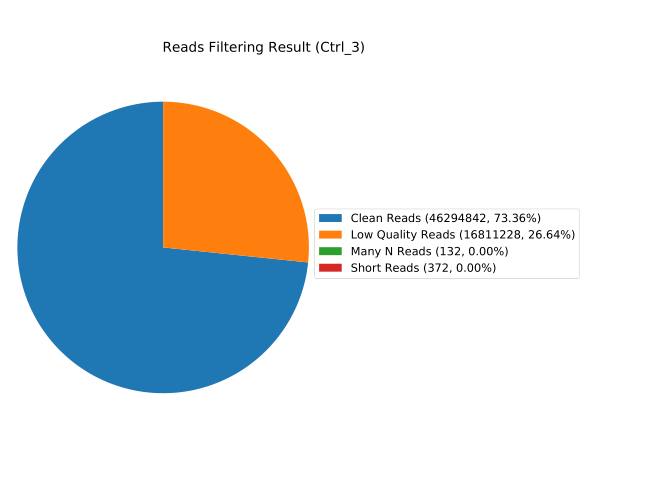

Supplement: Supplementary file 4 [file DataSheet2.zip › supp/QC/clean/Ctrl_3/Ctrl_3.reads.filter.png]

Percentage of Bases across All Bases (GRh2\_1)

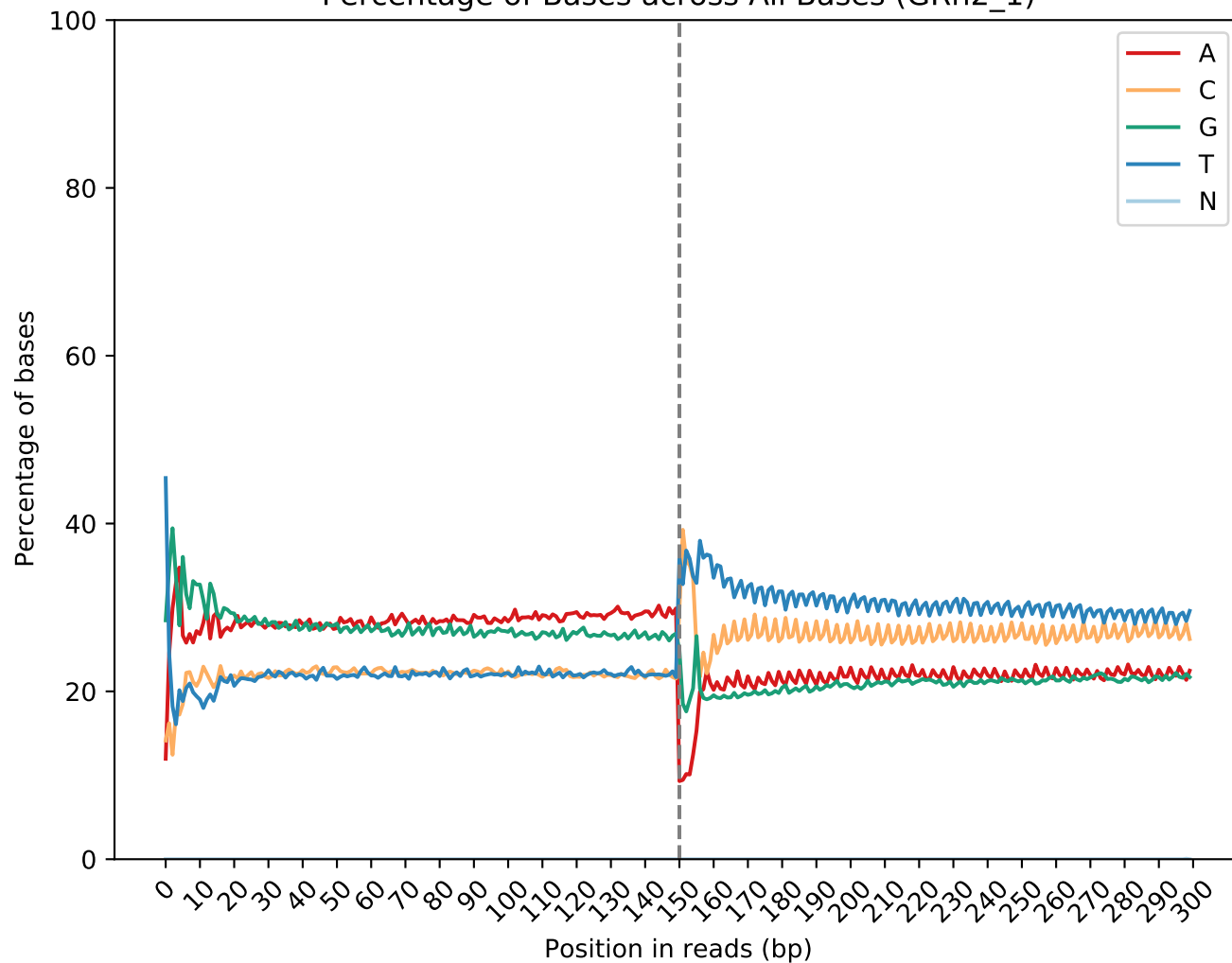

Supplement: Supplementary file 4 [file DataSheet2.zip › supp/QC/clean/GRh2_1/GRh2_1.bases.content.pdf]

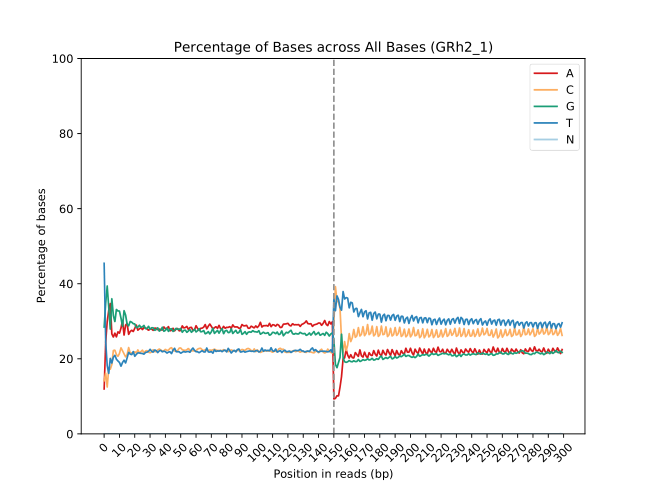

Supplement: Supplementary file 4 [file DataSheet2.zip › supp/QC/clean/GRh2_1/GRh2_1.bases.content.png]

Base Quality across All Bases (GRh2\_1)

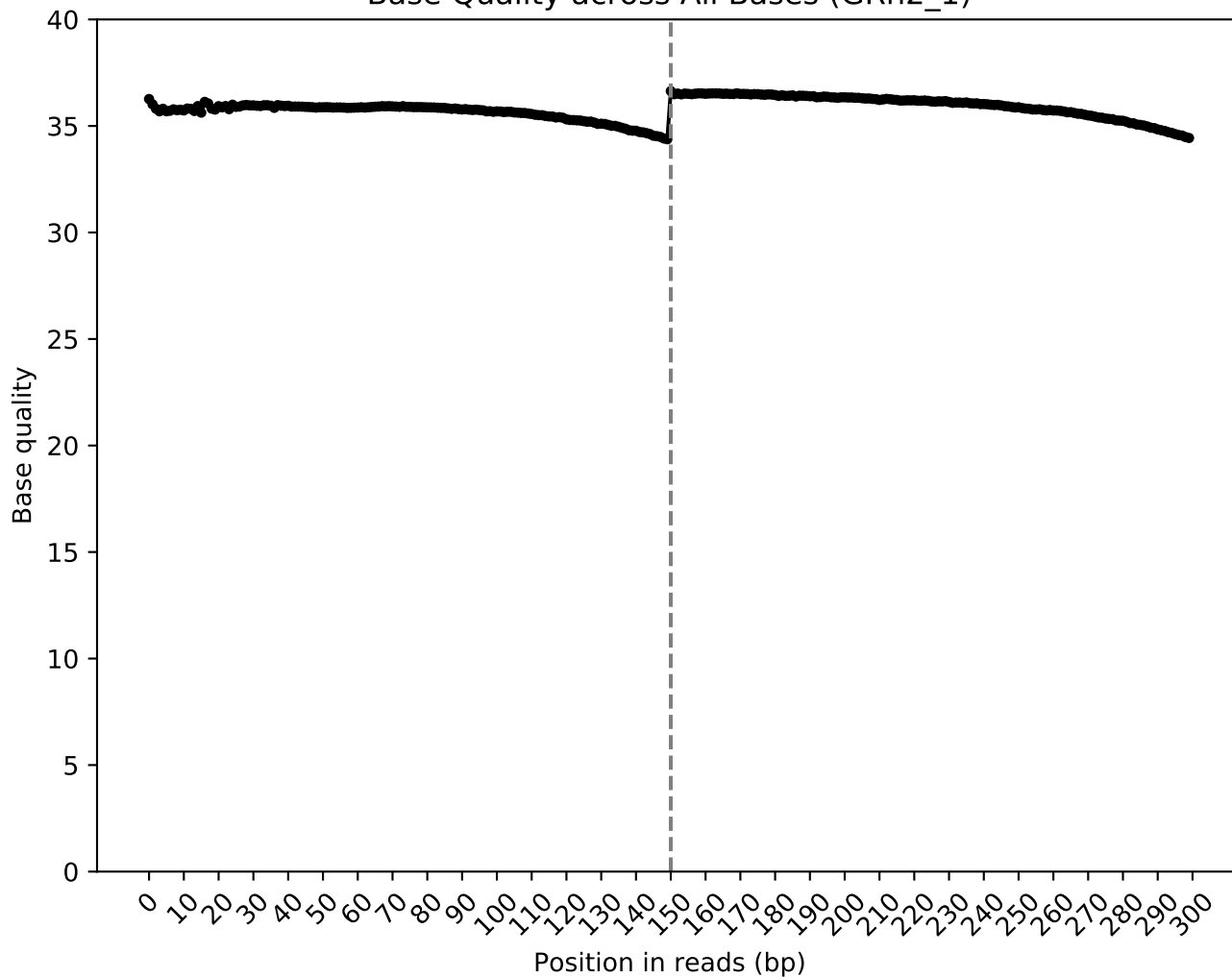

Supplement: Supplementary file 4 [file DataSheet2.zip › supp/QC/clean/GRh2_1/GRh2_1.bases.quality.pdf]

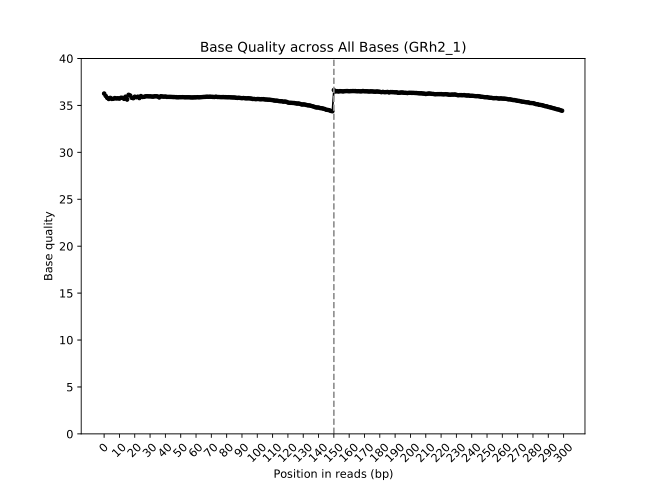

Supplement: Supplementary file 4 [file DataSheet2.zip › supp/QC/clean/GRh2_1/GRh2_1.bases.quality.png]

GC Distribution over all reads (GRh2\_1)

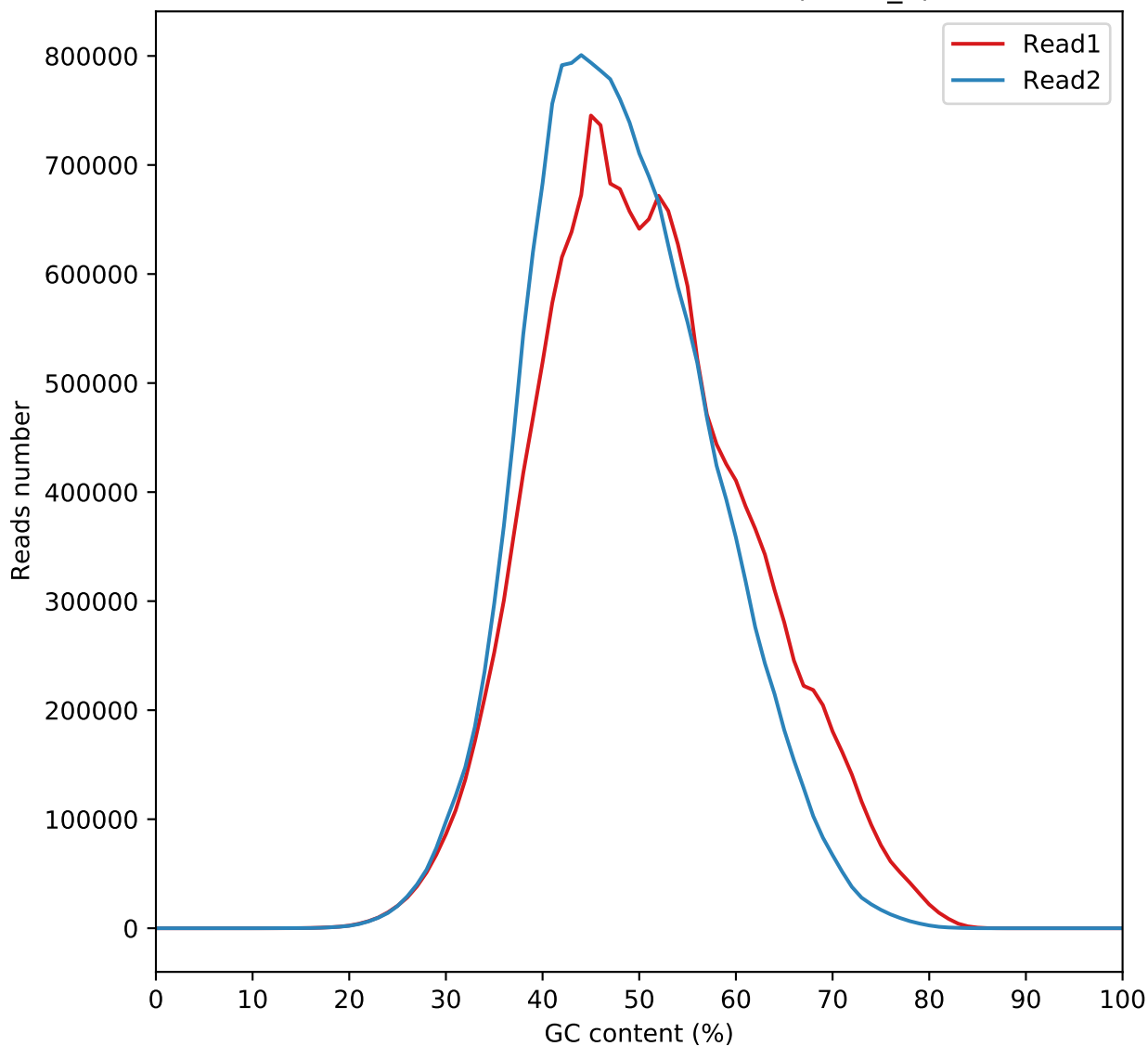

Supplement: Supplementary file 4 [file DataSheet2.zip › supp/QC/clean/GRh2_1/GRh2_1.GC.distribution.pdf]

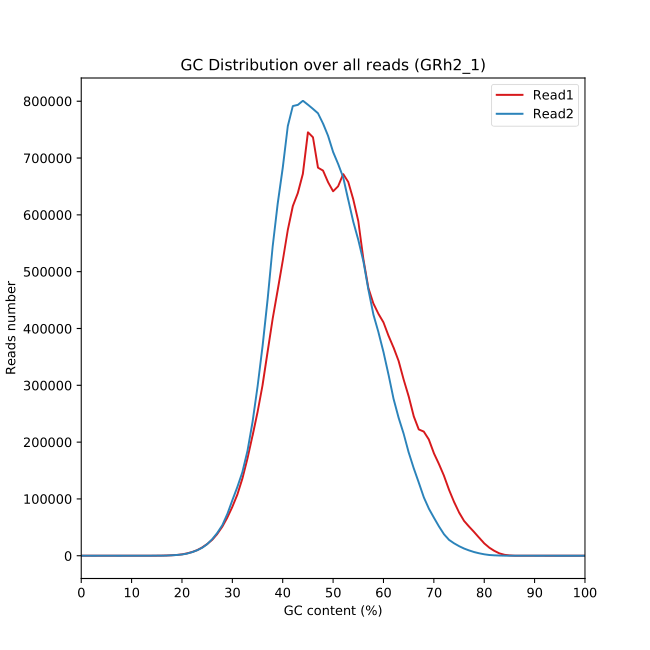

Supplement: Supplementary file 4 [file DataSheet2.zip › supp/QC/clean/GRh2_1/GRh2_1.GC.distribution.png]

## Reads Filtering Result (GRh2\_1)

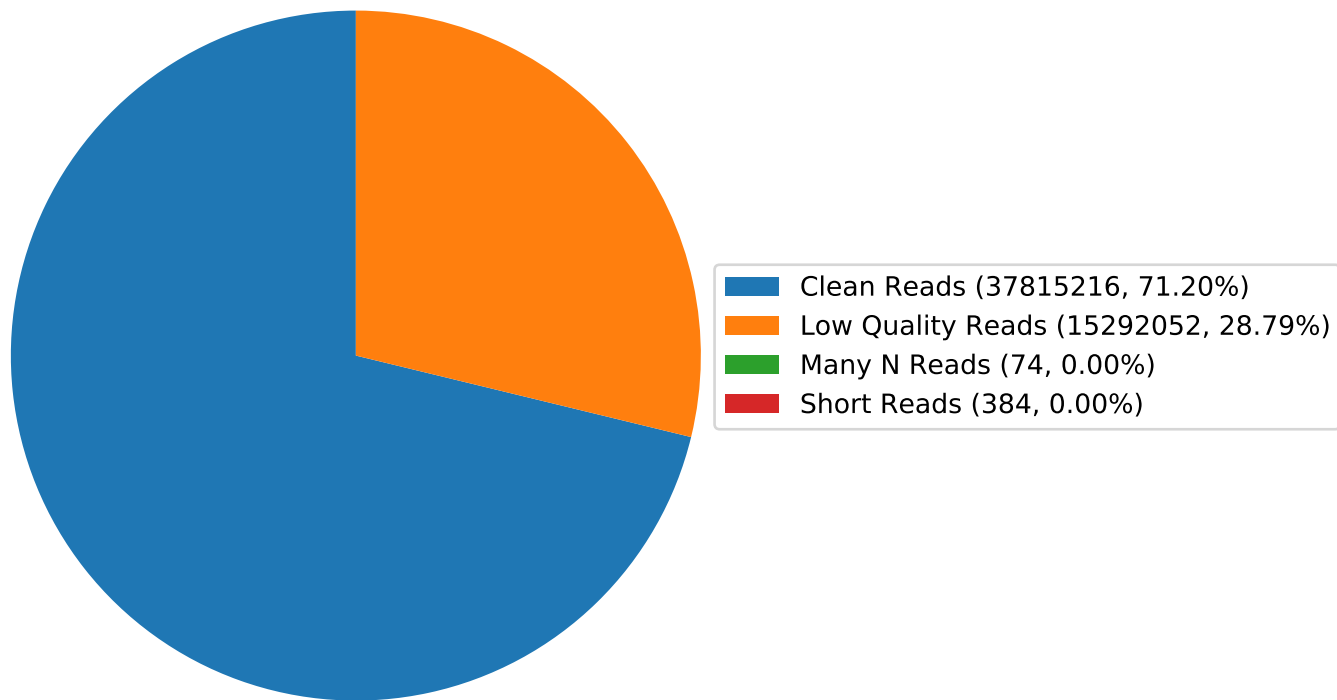

Supplement: Supplementary file 4 [file DataSheet2.zip › supp/QC/clean/GRh2_1/GRh2_1.reads.filter.pdf]

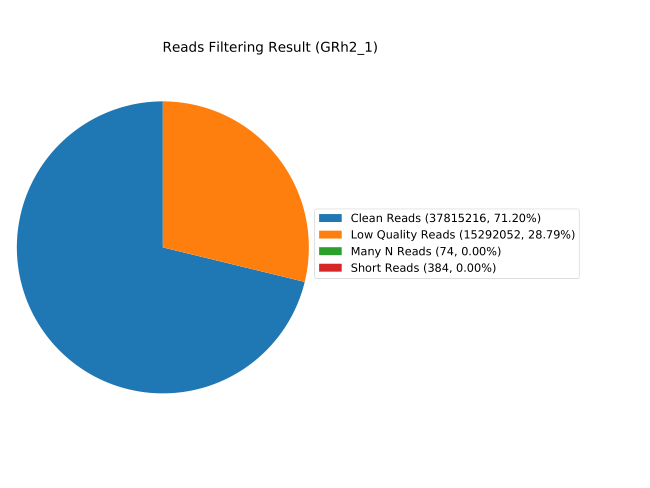

Supplement: Supplementary file 4 [file DataSheet2.zip › supp/QC/clean/GRh2_1/GRh2_1.reads.filter.png]

Percentage of Bases across All Bases (GRh2\_2)

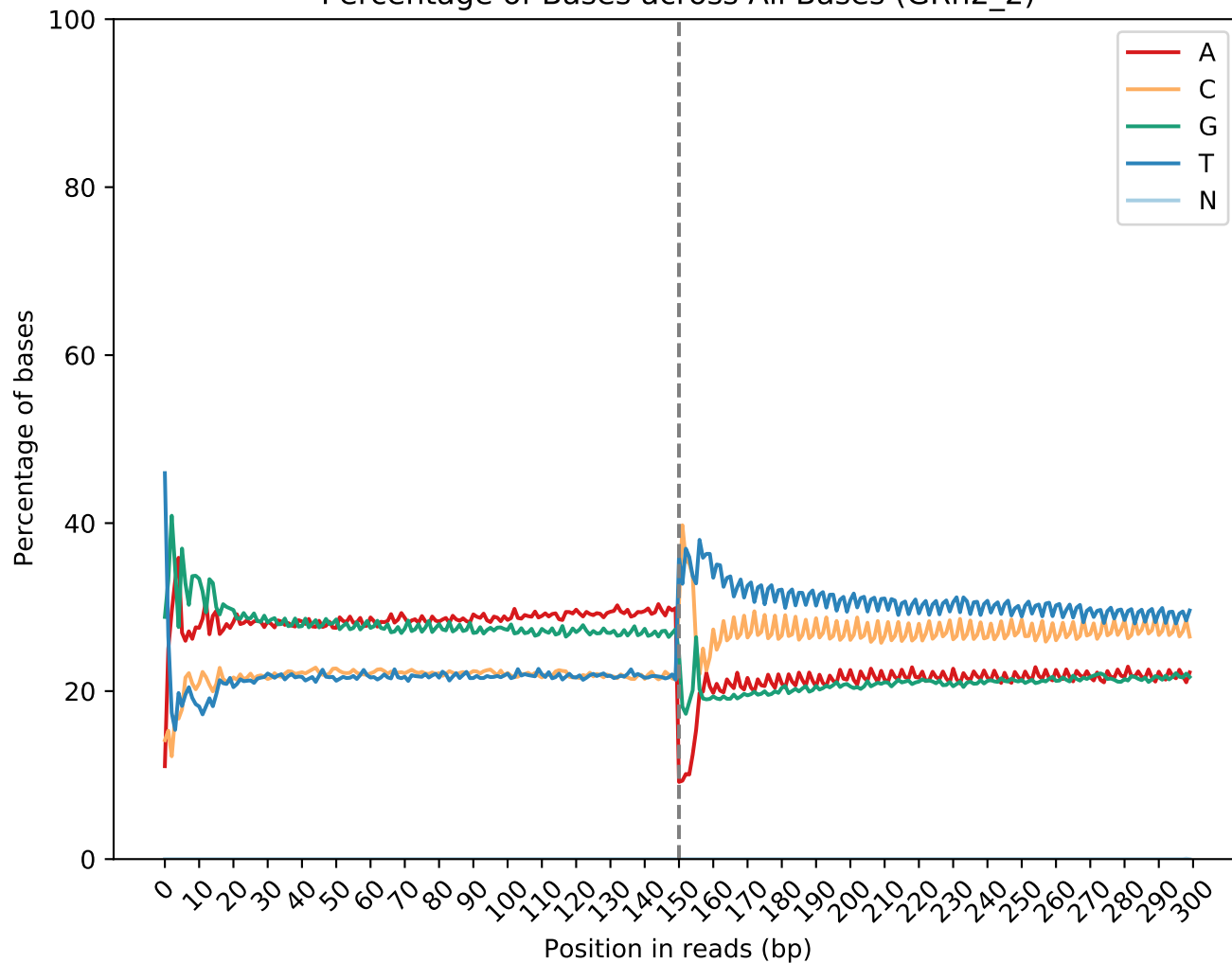

Supplement: Supplementary file 4 [file DataSheet2.zip › supp/QC/clean/GRh2_2/GRh2_2.bases.content.pdf]

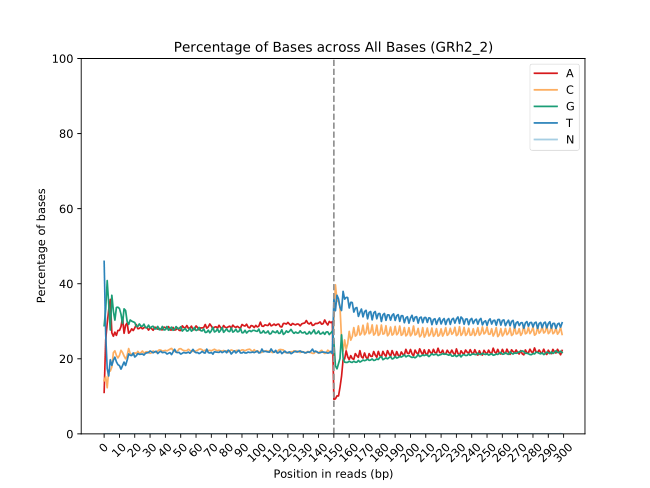

Supplement: Supplementary file 4 [file DataSheet2.zip › supp/QC/clean/GRh2_2/GRh2_2.bases.content.png]

Base Quality across All Bases (GRh2\_2)

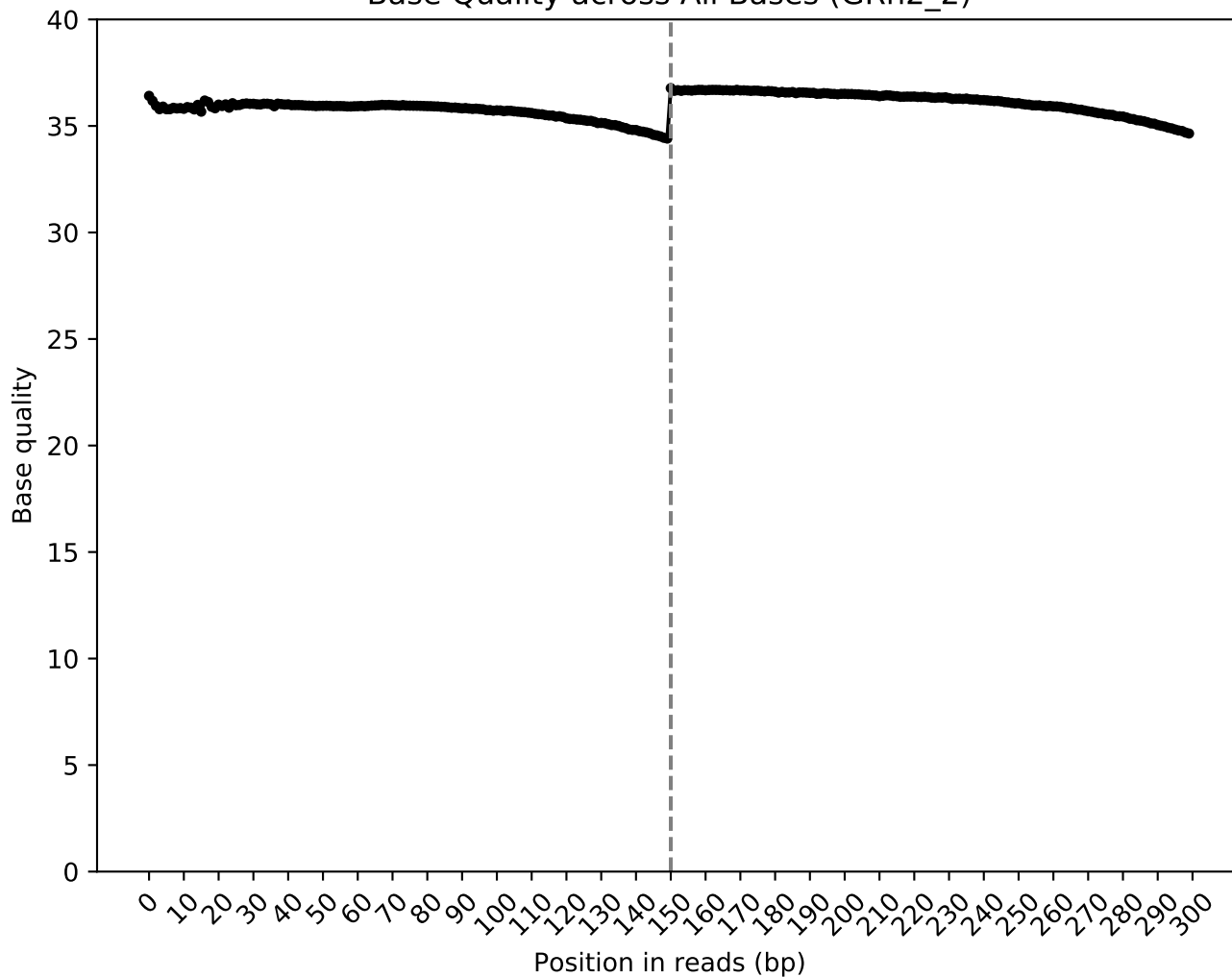

Supplement: Supplementary file 4 [file DataSheet2.zip › supp/QC/clean/GRh2_2/GRh2_2.bases.quality.pdf]

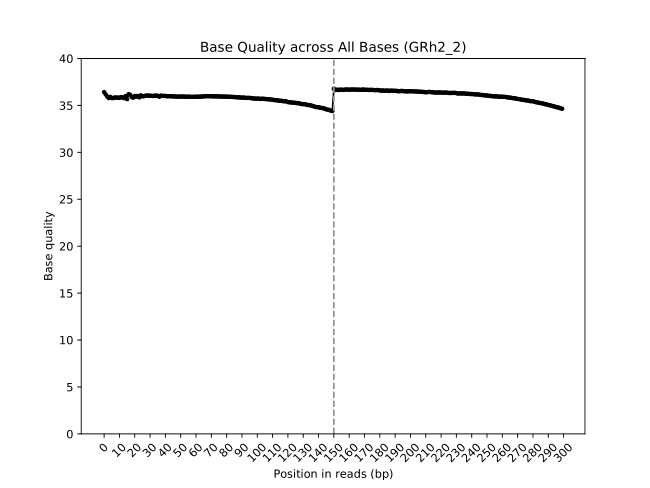

Supplement: Supplementary file 4 [file DataSheet2.zip › supp/QC/clean/GRh2_2/GRh2_2.bases.quality.png]

GC Distribution over all reads (GRh2\_2)

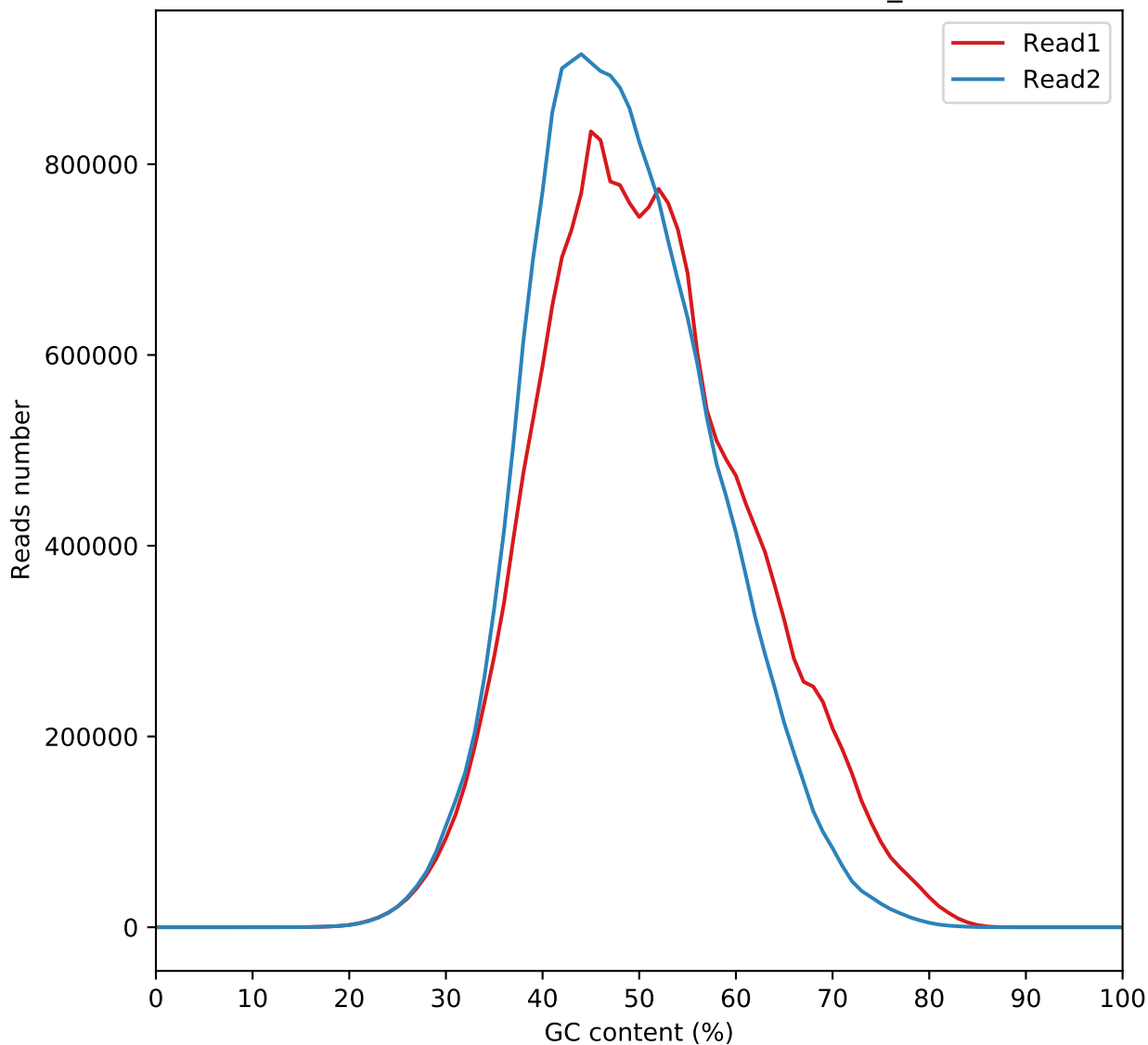

Supplement: Supplementary file 4 [file DataSheet2.zip › supp/QC/clean/GRh2_2/GRh2_2.GC.distribution.pdf]

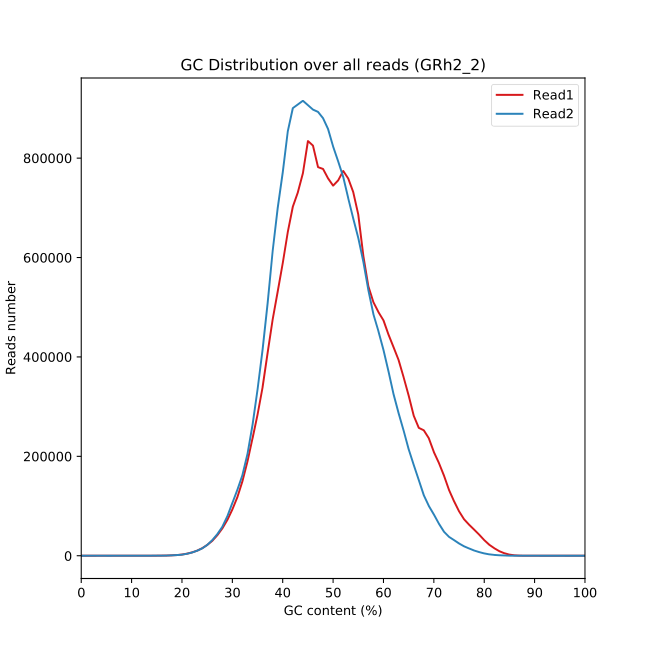

Supplement: Supplementary file 4 [file DataSheet2.zip › supp/QC/clean/GRh2_2/GRh2_2.GC.distribution.png]

## Reads Filtering Result (GRh2\_2)

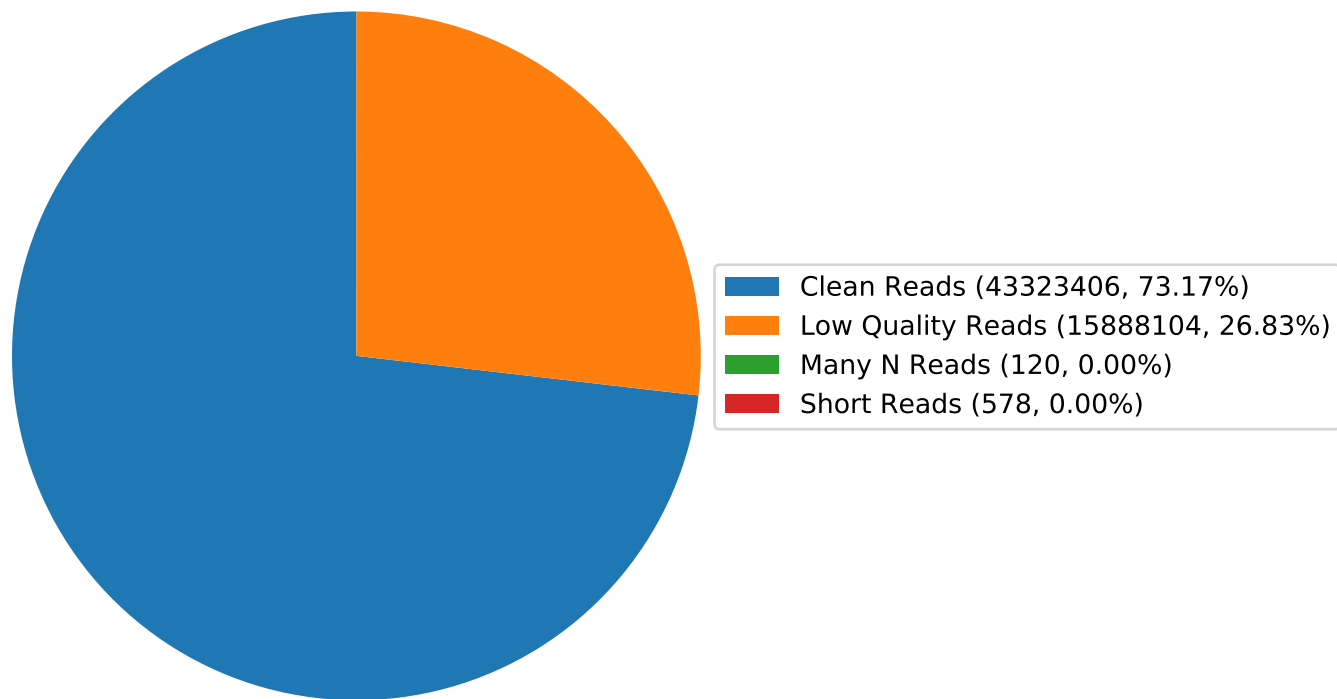

Supplement: Supplementary file 4 [file DataSheet2.zip › supp/QC/clean/GRh2_2/GRh2_2.reads.filter.pdf]

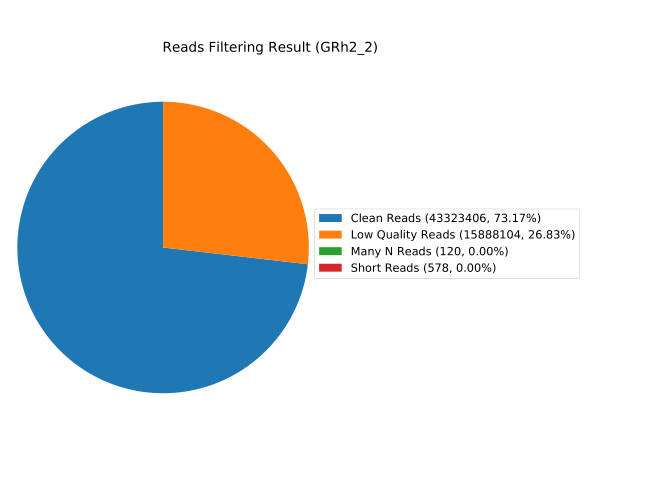

Supplement: Supplementary file 4 [file DataSheet2.zip › supp/QC/clean/GRh2_2/GRh2_2.reads.filter.png]

Percentage of Bases across All Bases (GRh2\_3)

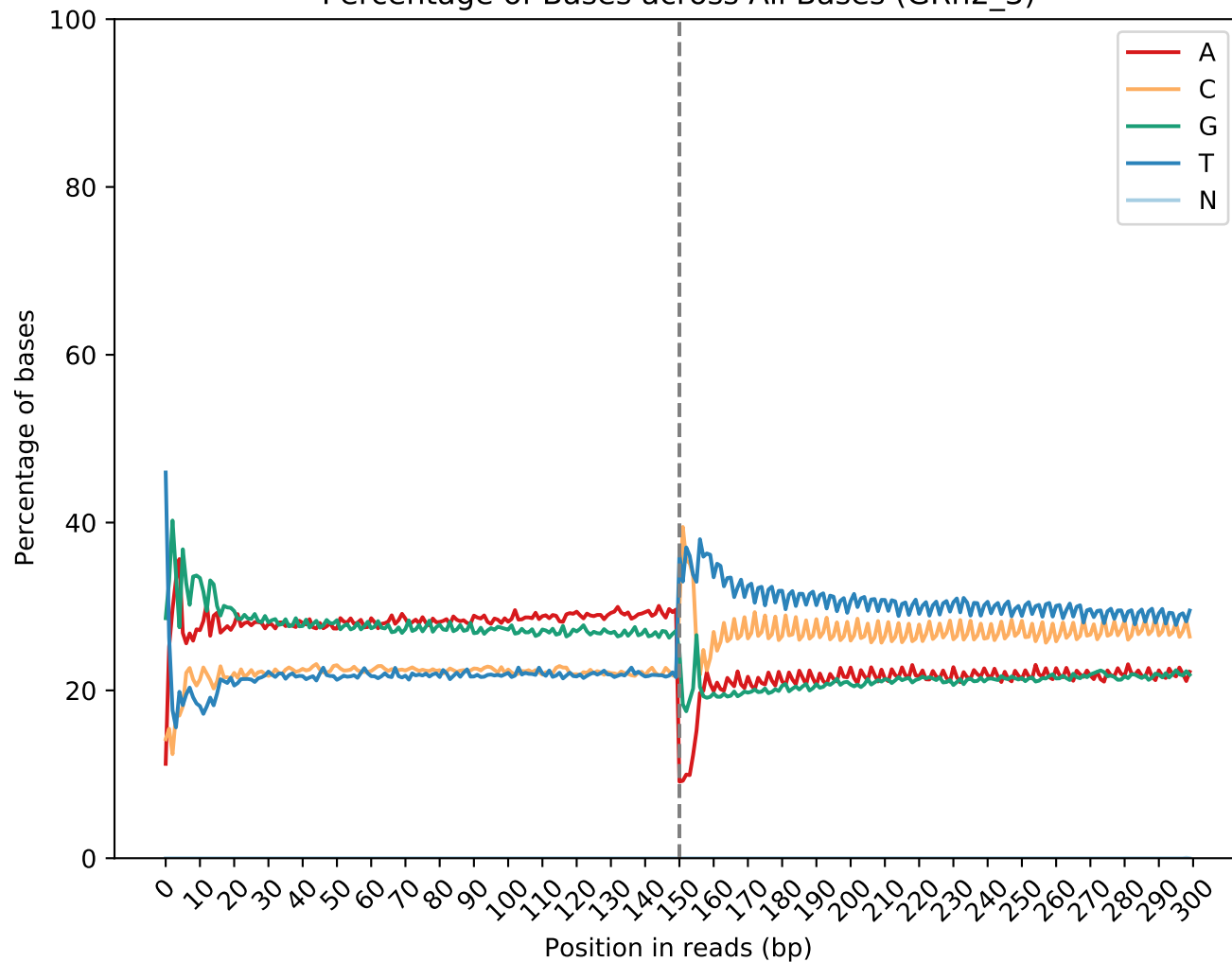

Supplement: Supplementary file 4 [file DataSheet2.zip › supp/QC/clean/GRh2_3/GRh2_3.bases.content.pdf]

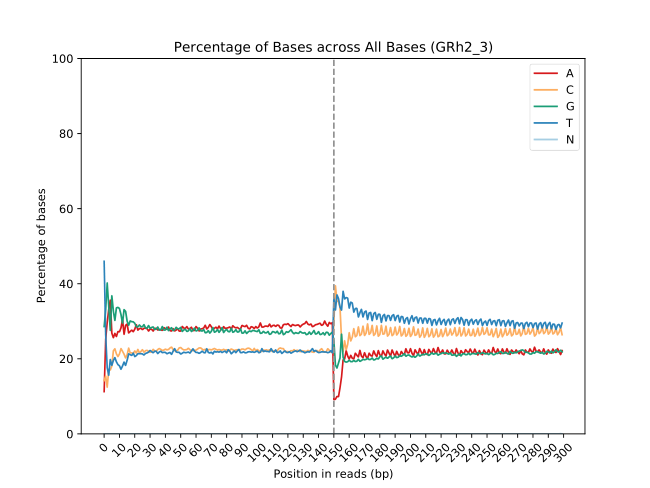

Supplement: Supplementary file 4 [file DataSheet2.zip › supp/QC/clean/GRh2_3/GRh2_3.bases.content.png]

Base Quality across All Bases (GRh2\_3)

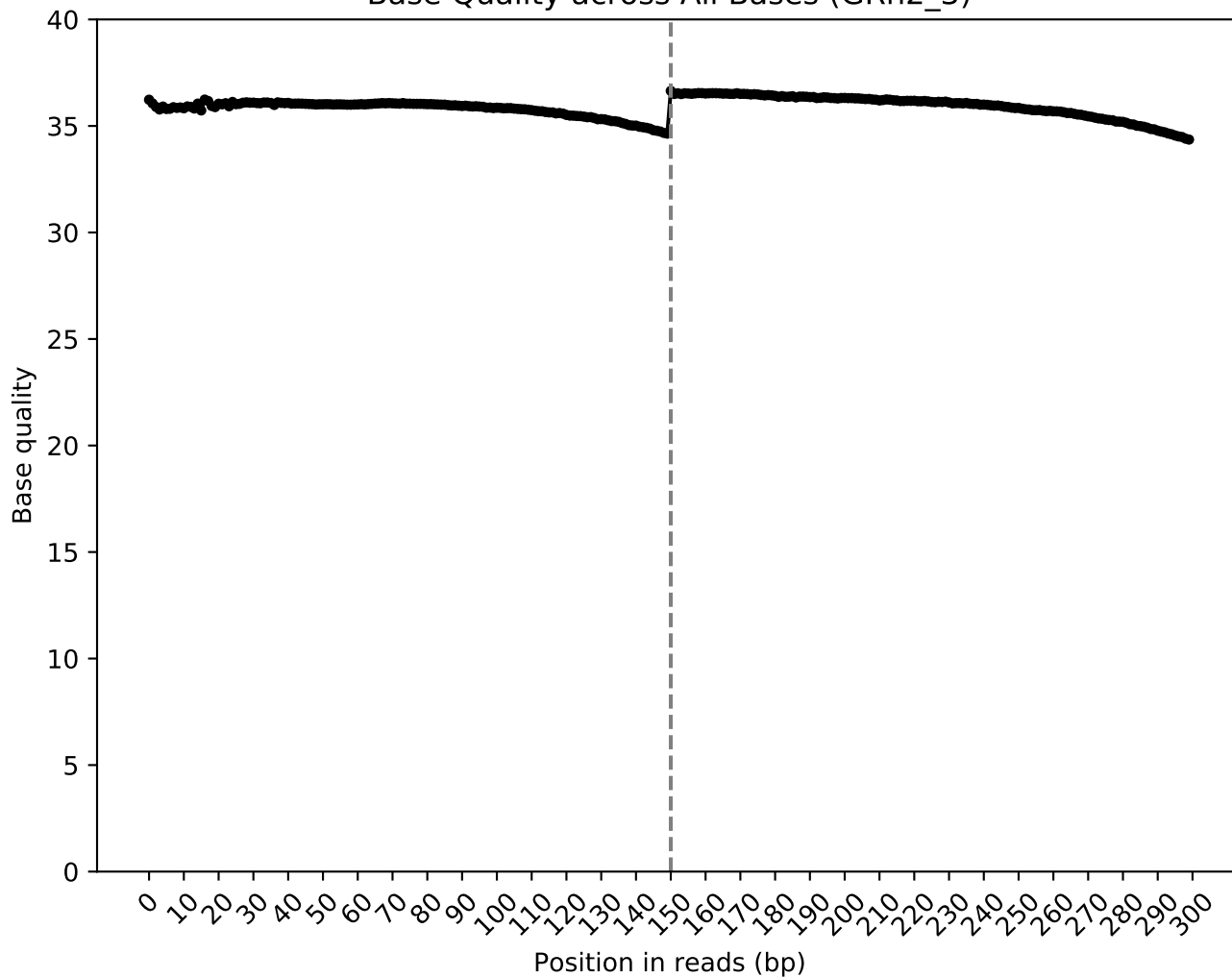

Supplement: Supplementary file 4 [file DataSheet2.zip › supp/QC/clean/GRh2_3/GRh2_3.bases.quality.pdf]
