# Supplementary figures and images for: Ginsenoside Rh2 repressed the progression of prostate cancer through the mitochondrial damage induced by mitophagy and ferroptosis (part 2 of 2)
Source: Front Oncol. 2025 Aug 21;15:1633891. doi: 10.3389/fonc.2025.1633891 (PMC12408308; doi:10.3389/fonc.2025.1633891)

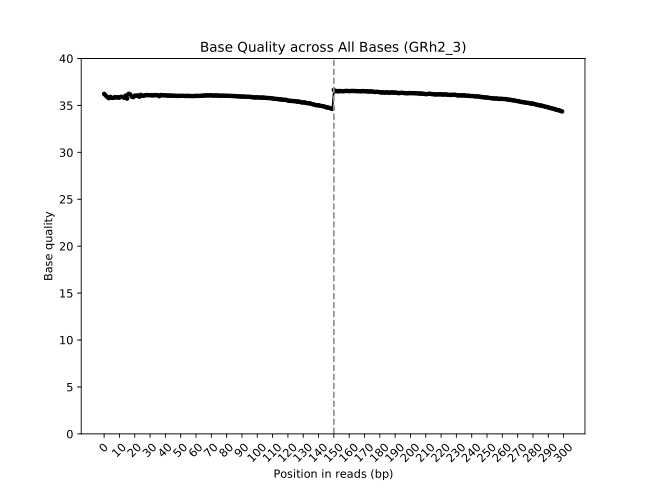

Supplement: Supplementary file 4 [file DataSheet2.zip › supp/QC/clean/GRh2_3/GRh2_3.bases.quality.png]

GC Distribution over all reads (GRh2\_3)

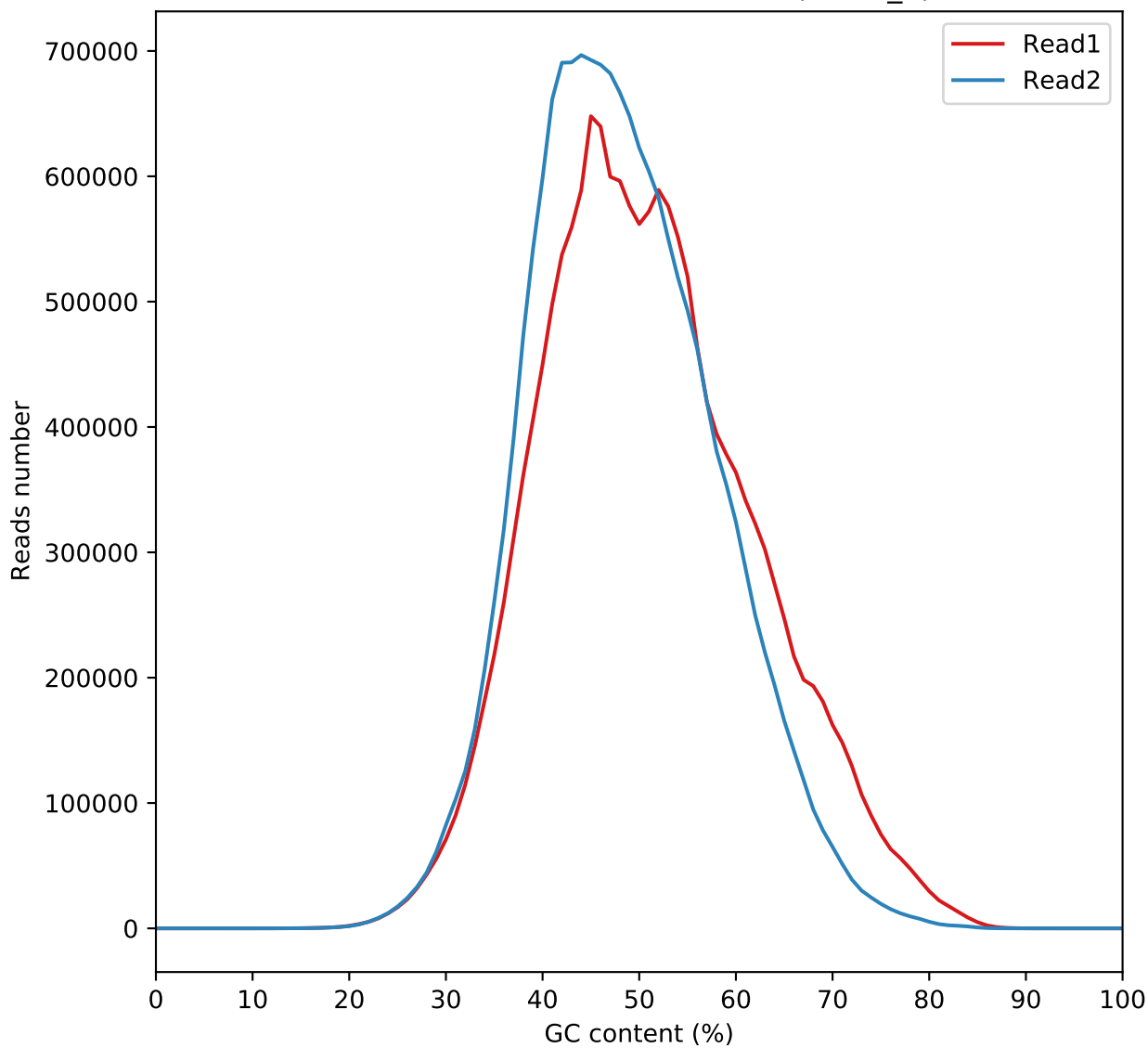

Supplement: Supplementary file 4 [file DataSheet2.zip › supp/QC/clean/GRh2_3/GRh2_3.GC.distribution.pdf]

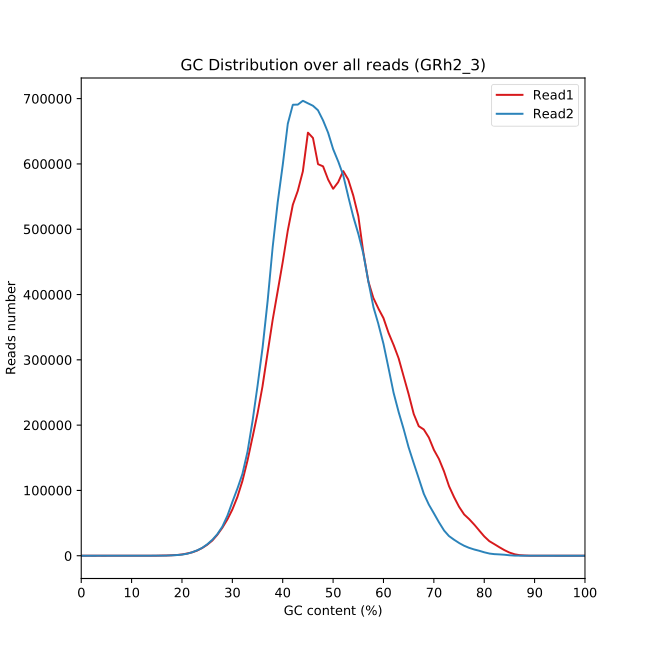

Supplement: Supplementary file 4 [file DataSheet2.zip › supp/QC/clean/GRh2_3/GRh2_3.GC.distribution.png]

## Reads Filtering Result (GRh2\_3)

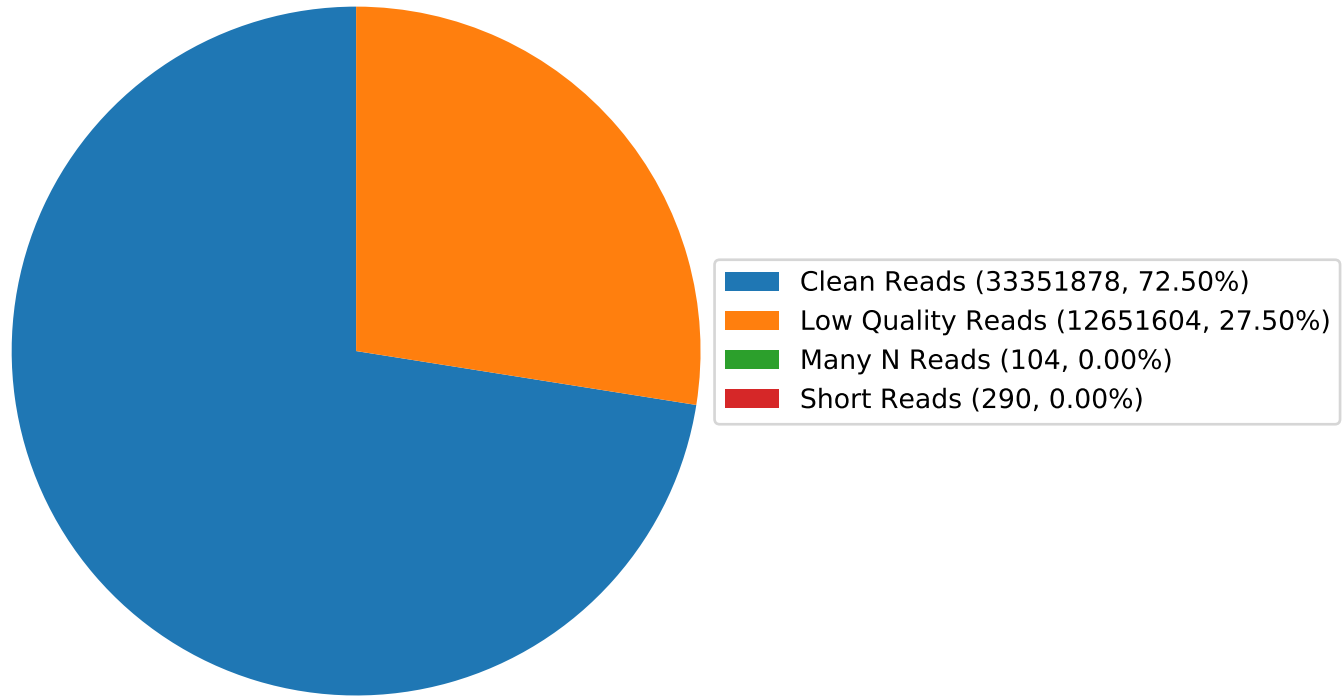

Supplement: Supplementary file 4 [file DataSheet2.zip › supp/QC/clean/GRh2_3/GRh2_3.reads.filter.pdf]

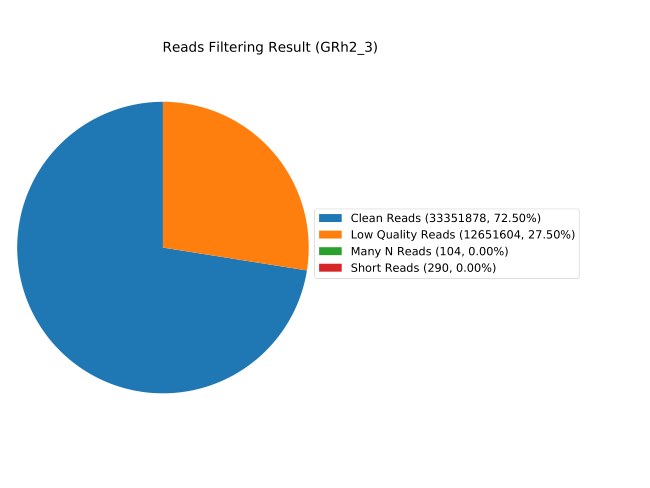

Supplement: Supplementary file 4 [file DataSheet2.zip › supp/QC/clean/GRh2_3/GRh2_3.reads.filter.png]
