# Supplementary material for: Ginsenoside Rh2 repressed the progression of prostate cancer through the mitochondrial damage induced by mitophagy and ferroptosis
Source: Front Oncol. 2025 Aug 21;15:1633891. doi: 10.3389/fonc.2025.1633891 (PMC12408308; doi:10.3389/fonc.2025.1633891)
Supplement: Supplementary file 4 [file heatmap_of_any_diff_genes_2.pdf › supp/DEG/GRh2___Ctrl/heatmap_of_any_diff_genes_2.pdf]

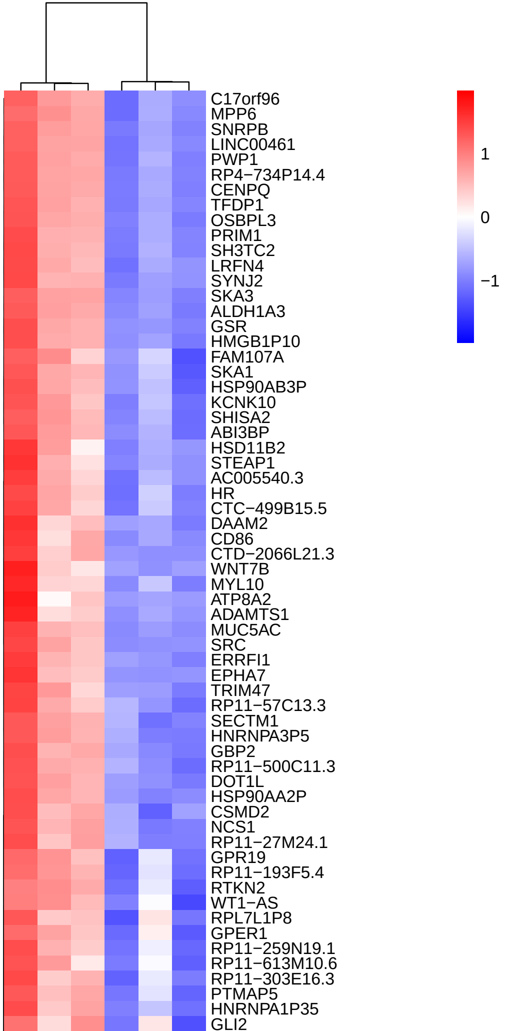
C17orf96
MPP6
SNRPB
LINC00461
PWP1
RP4−734P14.4
CENPQ
TFDP1
OSBPL3
PRIM1
SH3TC2
LRFN4
SYNJ2
SKA3
ALDH1A3
GSR
HMGB1P10
FAM107A
SKA1
HSP90AB3P
KCNK10
SHISA2
ABI3BP
HSD11B2
STEAP1
AC005540.3
HR
CTC−499B15.5
DAAM2
CD86
CTD−2066L21.3
WNT7B
MYL10
ATP8A2
ADAMTS1
MUC5AC
SRC
ERRFI1
EPHA7
TRIM47
RP11−57C13.3
SECTM1
HNRNPA3P5
GBP2
RP11−500C11.3
DOT1L
HSP90AA2P
CSMD2
NCS1
RP11−27M24.1
GPR19
RP11−193F5.4
RTKN2
WT1−AS
RPL7L1P8
GPER1
RP11−259N19.1
RP11−613M10.6
RP11−303E16.3
PTMAP5
HNRNPA1P35
GLI2
1
0
−1

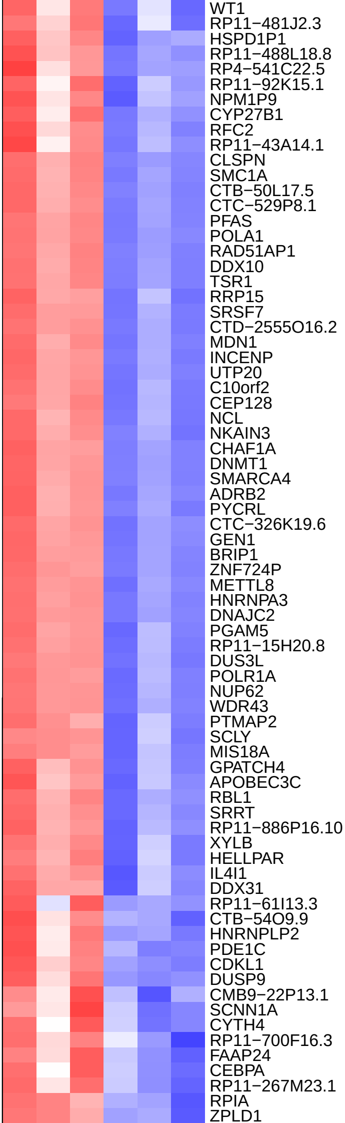
WT1
RP11−481J2.3
HSPD1P1
RP11−488L18.8
RP4−541C22.5
RP11−92K15.1
NPM1P9
CYP27B1
RFC2
RP11−43A14.1
CLSPN
SMC1A
CTB−50L17.5
CTC−529P8.1
PFAS
POLA1
RAD51AP1
DDX10
TSR1
RRP15
SRSF7
CTD−2555O16.2
MDN1
INCENP
UTP20
C10orf2
CEP128
NCL
NKAIN3
CHAF1A
DNMT1
SMARCA4
ADRB2
PYCRL
CTC−326K19.6
GEN1
BRIP1
ZNF724P
METTL8
HNRNPA3
DNAJC2
PGAM5
RP11−15H20.8
DUS3L
POLR1A
NUP62
WDR43
PTMAP2
SCLY
MIS18A
GPATCH4
APOBEC3C
RBL1
SRRT
RP11−886P16.10
XYLB
HELLPAR
IL4I1
DDX31
RP11−61I13.3
CTB−54O9.9
HNRNPLP2
PDE1C
CDKL1
DUSP9
CMB9−22P13.1
SCNN1A
CYTH4
RP11−700F16.3
FAAP24
CEBPA
RP11−267M23.1
RPIA
ZPLD1

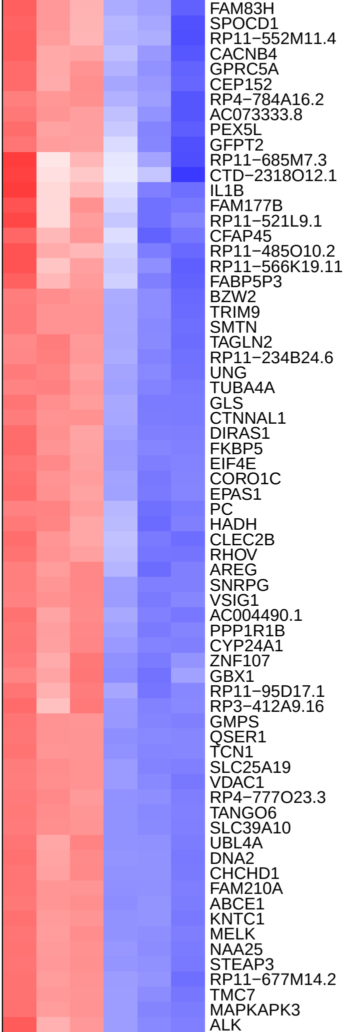
FAM83H
SPOCD1
RP11−552M11.4
CACNB4
GPRC5A
CEP152
RP4−784A16.2
AC073333.8
PEX5L
GFPT2
RP11−685M7.3
CTD−2318O12.1
IL1B
FAM177B
RP11−521L9.1
CFAP45
RP11−485O10.2
RP11−566K19.11
FABP5P3
BZW2
TRIM9
SMTN
TAGLN2
RP11−234B24.6
UNG
TUBA4A
GLS
CTNNAL1
DIRAS1
FKBP5
EIF4E
CORO1C
EPAS1
PC
HADH
CLEC2B
RHOV
AREG
SNRPG
VSIG1
AC004490.1
PPP1R1B
CYP24A1
ZNF107
GBX1
RP11−95D17.1
RP3−412A9.16
GMPS
QSER1
TCN1
SLC25A19
VDAC1
RP4−777O23.3
TANGO6
SLC39A10
UBL4A
DNA2
CHCHD1
FAM210A
ABCE1
KNTC1
MELK
NAA25
STEAP3
RP11−677M14.2
TMC7
MAPKAPK3
ALK

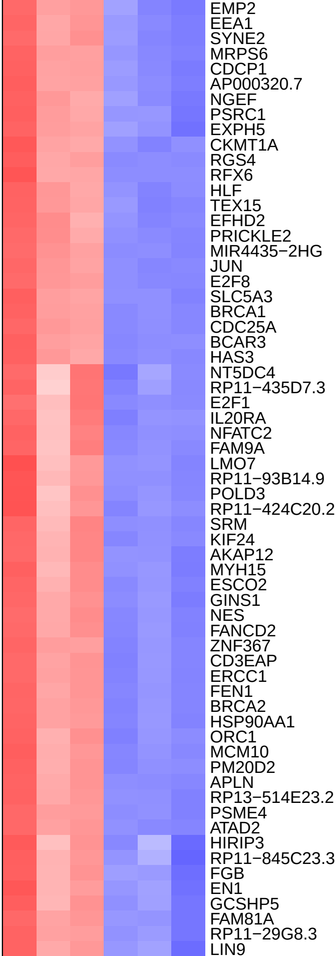
EMP2
EEA1
SYNE2
MRPS6
CDCP1
AP000320.7
NGEF
PSRC1
EXPH5
CKMT1A
RGS4
RFX6
HLF
TEX15
EFHD2
PRICKLE2
MIR4435−2HG
JUN
E2F8
SLC5A3
BRCA1
CDC25A
BCAR3
HAS3
NT5DC4
RP11−435D7.3
E2F1
IL20RA
NFATC2
FAM9A
LMO7
RP11−93B14.9
POLD3
RP11−424C20.2
SRM
KIF24
AKAP12
MYH15
ESCO2
GINS1
NES
FANCD2
ZNF367
CD3EAP
ERCC1
FEN1
BRCA2
HSP90AA1
ORC1
MCM10
PM20D2
APLN
RP13−514E23.2
PSME4
ATAD2
HIRIP3
RP11−845C23.3
FGB
EN1
GCSHP5
FAM81A
RP11−29G8.3
LIN9

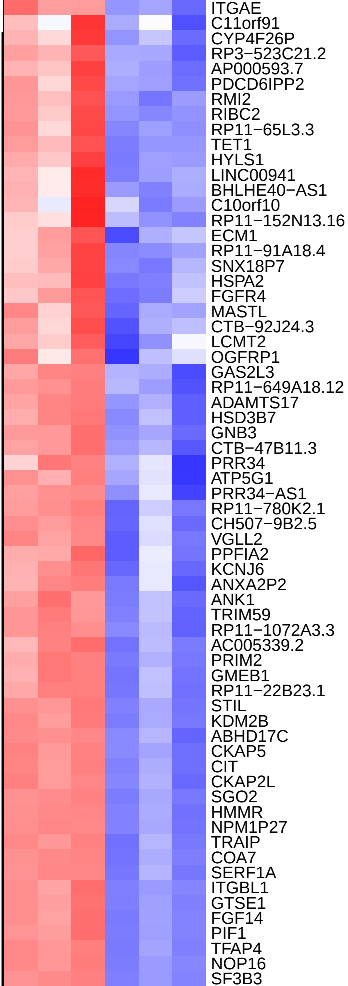
ITGAE
C11orf91
CYP4F26P
RP3−523C21.2
AP000593.7
PDCD6IPP2
RMI2
RIBC2
RP11−65L3.3
TET1
HYLS1
LINC00941
BHLHE40−AS1
C10orf10
RP11−152N13.16
ECM1
RP11−91A18.4
SNX18P7
HSPA2
FGFR4
MASTL
CTB−92J24.3
LCMT2
OGFRP1
GAS2L3
RP11−649A18.12
ADAMTS17
HSD3B7
GNB3
CTB−47B11.3
PRR34
ATP5G1
PRR34−AS1
RP11−780K2.1
CH507−9B2.5
VGLL2
PPFIA2
KCNJ6
ANXA2P2
ANK1
TRIM59
RP11−1072A3.3
AC005339.2
PRIM2
GMEB1
RP11−22B23.1
STIL
KDM2B
ABHD17C
CKAP5
CIT
CKAP2L
SGO2
HMMR
NPM1P27
TRAIP
COA7
SERF1A
ITGBL1
GTSE1
FGF14
PIF1
TFAP4
NOP16
SF3B3

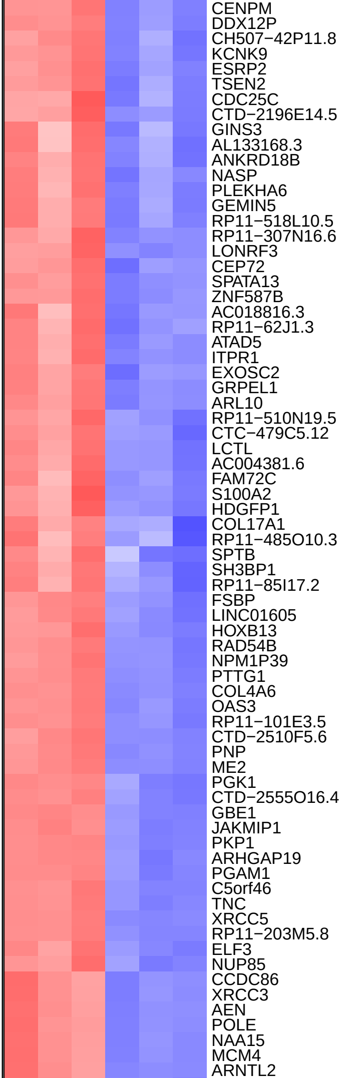

CENPM
DDX12P
CH507−42P11.8
KCNK9
ESRP2
TSEN2
CDC25C
CTD−2196E14.5
GINS3
AL133168.3
ANKRD18B
NASP
PLEKHA6
GEMIN5
RP11−518L10.5
RP11−307N16.6
LONRF3
CEP72
SPATA13
ZNF587B
AC018816.3
RP11−62J1.3
ATAD5
ITPR1
EXOSC2
GRPEL1
ARL10
RP11−510N19.5
CTC−479C5.12
LCTL
AC004381.6
FAM72C
S100A2
HDGFP1
COL17A1
RP11−485O10.3
SPTB
SH3BP1
RP11−85I17.2
FSBP
LINC01605
HOXB13
RAD54B
NPM1P39
PTTG1
COL4A6
OAS3
RP11−101E3.5
CTD−2510F5.6
PNP
ME2
PGK1
CTD−2555O16.4
GBE1
JAKMIP1
PKP1
ARHGAP19
PGAM1
C5orf46
TNC
XRCC5
RP11−203M5.8
ELF3
NUP85
CCDC86
XRCC3
AEN
POLE
NAA15
MCM4
ARNTL2

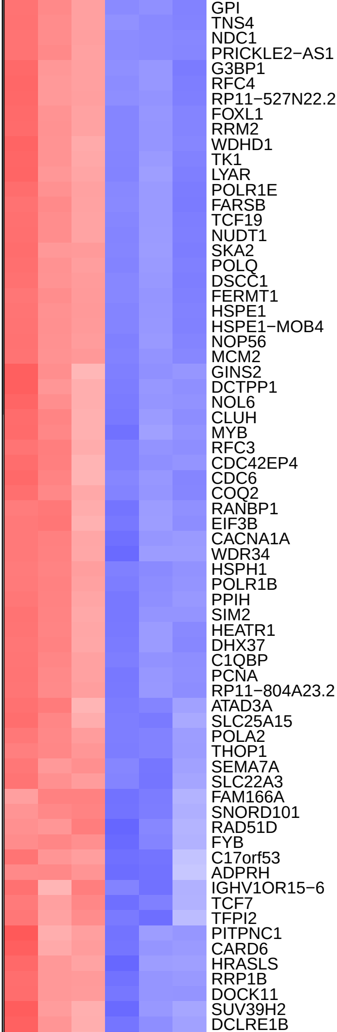
GPI
TNS4
NDC1
PRICKLE2−AS1
G3BP1
RFC4
RP11−527N22.2
FOXL1
RRM2
WDHD1
TK1
LYAR
POLR1E
FARSB
TCF19
NUDT1
SKA2
POLQ
DSCC1
FERMT1
HSPE1
HSPE1−MOB4
NOP56
MCM2
GINS2
DCTPP1
NOL6
CLUH
MYB
RFC3
CDC42EP4
CDC6
COQ2
RANBP1
EIF3B
CACNA1A
WDR34
HSPH1
POLR1B
PPIH
SIM2
HEATR1
DHX37
C1QBP
PCNA
RP11−804A23.2
ATAD3A
SLC25A15
POLA2
THOP1
SEMA7A
SLC22A3
FAM166A
SNORD101
RAD51D
FYB
C17orf53
ADPRH
IGHV1OR15−6
TCF7
TFPI2
PITPNC1
CARD6
HRASLS
RRP1B
DOCK11
SUV39H2
DCLRE1B

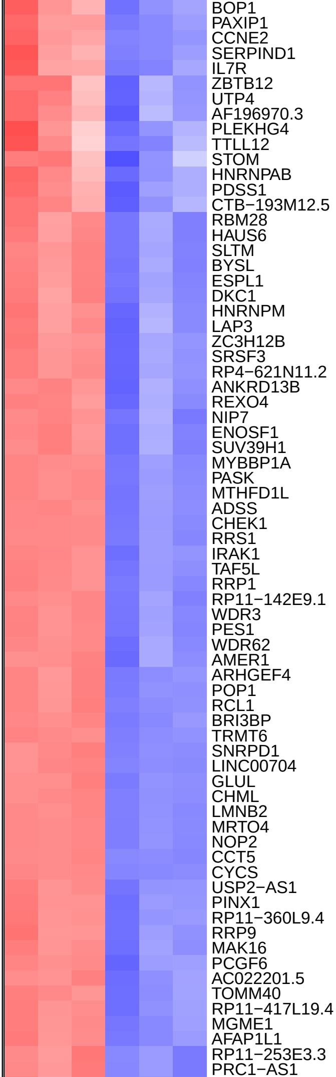
BOP1
PAXIP1
CCNE2
SERPIND1
IL7R
ZBTB12
UTP4
AF196970.3
PLEKHG4
TTLL12
STOM
HNRNPAB
PDSS1
CTB−193M12.5
RBM28
HAUS6
SLTM
BYSL
ESPL1
DKC1
HNRNPM
LAP3
ZC3H12B
SRSF3
RP4−621N11.2
ANKRD13B
REXO4
NIP7
ENOSF1
SUV39H1
MYBBP1A
PASK
MTHFD1L
ADSS
CHEK1
RRS1
IRAK1
TAF5L
RRP1
RP11−142E9.1
WDR3
PES1
WDR62
AMER1
ARHGEF4
POP1
RCL1
BRI3BP
TRMT6
SNRPD1
LINC00704
GLUL
CHML
LMNB2
MRTO4
NOP2
CCT5
CYCS
USP2−AS1
PINX1
RP11−360L9.4
RRP9
MAK16
PCGF6
AC022201.5
TOMM40
RP11−417L19.4
MGME1
AFAP1L1
RP11−253E3.3
PRC1−AS1

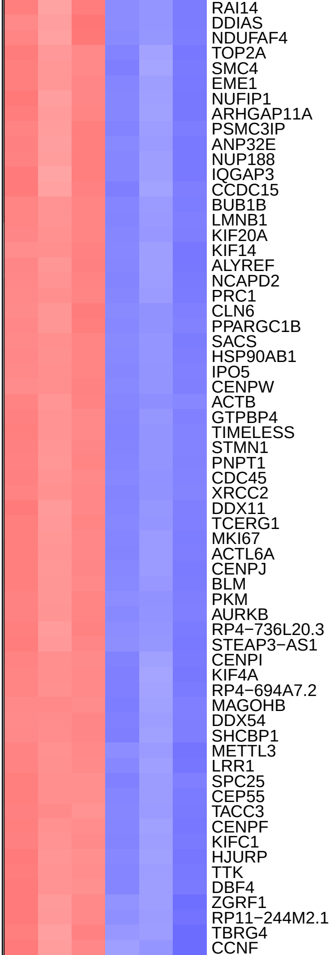
RAI14
DDIAS
NDUFAF4
TOP2A
SMC4
EME1
NUFIP1
ARHGAP11A
PSMC3IP
ANP32E
NUP188
IQGAP3
CCDC15
BUB1B
LMNB1
KIF20A
KIF14
ALYREF
NCAPD2
PRC1
CLN6
PPARGC1B
SACS
HSP90AB1
IPO5
CENPW
ACTB
GTPBP4
TIMELESS
STMN1
PNPT1
CDC45
XRCC2
DDX11
TCERG1
MKI67
ACTL6A
CENPJ
BLM
PKM
AURKB
RP4−736L20.3
STEAP3−AS1
CENPI
KIF4A
RP4−694A7.2
MAGOHB
DDX54
SHCBP1
METTL3
LRR1
SPC25
CEP55
TACC3
CENPF
KIFC1
HJURP
TTK
DBF4
ZGRF1
RP11−244M2.1
TBRG4
CCNF

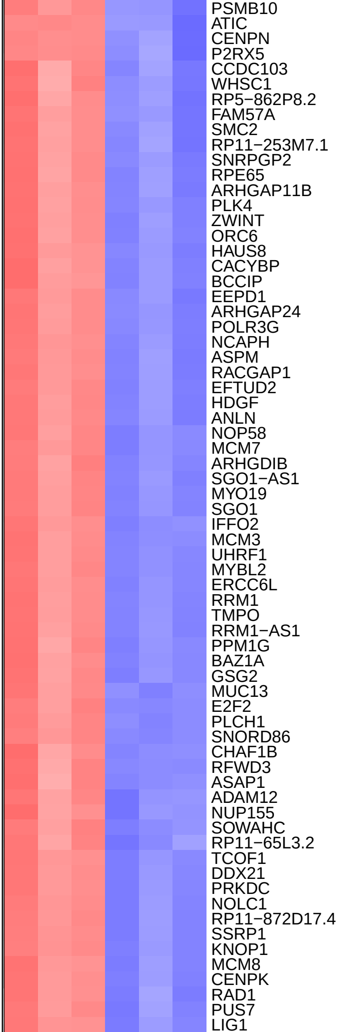
PSMB10
ATIC
CENPN
P2RX5
CCDC103
WHSC1
RP5−862P8.2
FAM57A
SMC2
RP11−253M7.1
SNRPGP2
RPE65
ARHGAP11B
PLK4
ZWINT
ORC6
HAUS8
CACYBP
BCCIP
EEPD1
ARHGAP24
POLR3G
NCAPH
ASPM
RACGAP1
EFTUD2
HDGF
ANLN
NOP58
MCM7
ARHGDIB
SGO1−AS1
MYO19
SGO1
IFFO2
MCM3
UHRF1
MYBL2
ERCC6L
RRM1
TMPO
RRM1−AS1
PPM1G
BAZ1A
GSG2
MUC13
E2F2
PLCH1
SNORD86
CHAF1B
RFWD3
ASAP1
ADAM12
NUP155
SOWAHC
RP11−65L3.2
TCOF1
DDX21
PRKDC
NOLC1
RP11−872D17.4
SSRP1
KNOP1
MCM8
CENPK
RAD1
PUS7
LIG1

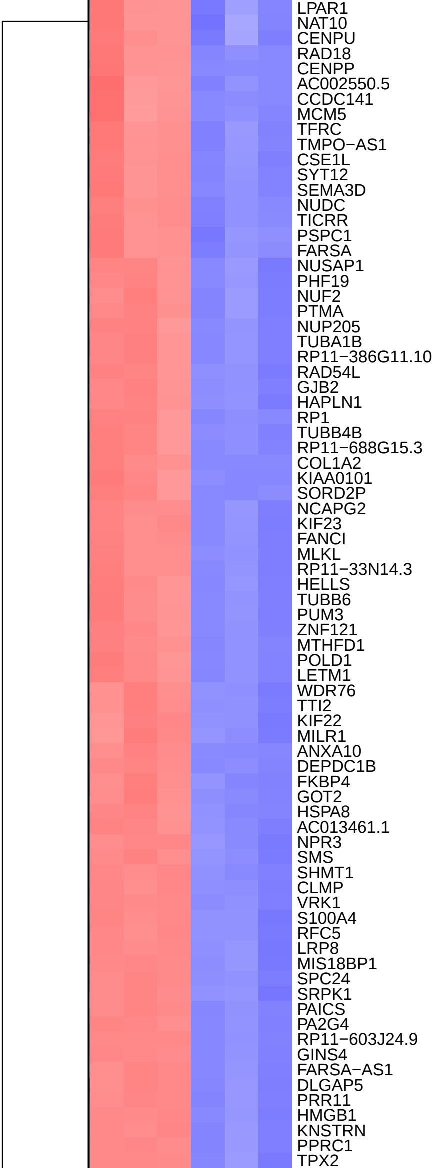
LPAR1
NAT10
CENPU
RAD18
CENPP
AC002550.5
CCDC141
MCM5
TFRC
TMPO−AS1
CSE1L
SYT12
SEMA3D
NUDC
TICRR
PSPC1
FARSA
NUSAP1
PHF19
NUF2
PTMA
NUP205
TUBA1B
RP11−386G11.10
RAD54L
GJB2
HAPLN1
RP1
TUBB4B
RP11−688G15.3
COL1A2
KIAA0101
SORD2P
NCAPG2
KIF23
FANCI
MLKL
RP11−33N14.3
HELLS
TUBB6
PUM3
ZNF121
MTHFD1
POLD1
LETM1
WDR76
TTI2
KIF22
MILR1
ANXA10
DEPDC1B
FKBP4
GOT2
HSPA8
AC013461.1
NPR3
SMS
SHMT1
CLMP
VRK1
S100A4
RFC5
LRP8
MIS18BP1
SPC24
SRPK1
PAICS
PA2G4
RP11−603J24.9
GINS4
FARSA−AS1
DLGAP5
PRR11
HMGB1
KNSTRN
PPRC1
TPX2

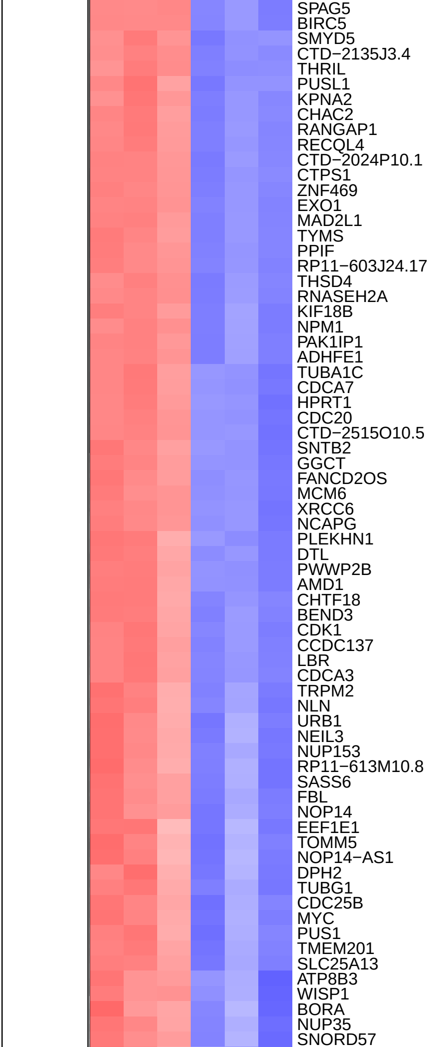
SPAG5
BIRC5
SMYD5
CTD−2135J3.4
THRIL
PUSL1
KPNA2
CHAC2
RANGAP1
RECQL4
CTD−2024P10.1
CTPS1
ZNF469
EXO1
MAD2L1
TYMS
PPIF
RP11−603J24.17
THSD4
RNASEH2A
KIF18B
NPM1
PAK1IP1
ADHFE1
TUBA1C
CDCA7
HPRT1
CDC20
CTD−2515O10.5
SNTB2
GGCT
FANCD2OS
MCM6
XRCC6
NCAPG
PLEKHN1
DTL
PWWP2B
AMD1
CHTF18
BEND3
CDK1
CCDC137
LBR
CDCA3
TRPM2
NLN
URB1
NEIL3
NUP153
RP11−613M10.8
SASS6
FBL
NOP14
EEF1E1
TOMM5
NOP14−AS1
DPH2
TUBG1
CDC25B
MYC
PUS1
TMEM201
SLC25A13
ATP8B3
WISP1
BORA
NUP35
SNORD57

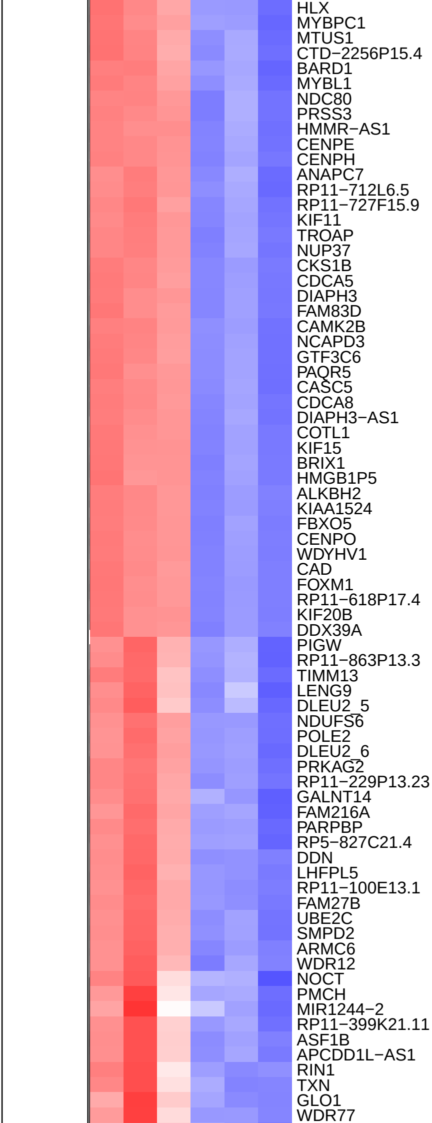
HLX
MYBPC1
MTUS1
CTD−2256P15.4
BARD1
MYBL1
NDC80
PRSS3
HMMR−AS1
CENPE
CENPH
ANAPC7
RP11−712L6.5
RP11−727F15.9
KIF11
TROAP
NUP37
CKS1B
CDCA5
DIAPH3
FAM83D
CAMK2B
NCAPD3
GTF3C6
PAQR5
CASC5
CDCA8
DIAPH3−AS1
COTL1
KIF15
BRIX1
HMGB1P5
ALKBH2
KIAA1524
FBXO5
CENPO
WDYHV1
CAD
FOXM1
RP11−618P17.4
KIF20B
DDX39A
PIGW
RP11−863P13.3
TIMM13
LENG9
DLEU2_5
NDUFS6
POLE2
DLEU2_6
PRKAG2
RP11−229P13.23
GALNT14
FAM216A
PARPBP
RP5−827C21.4
DDN
LHFPL5
RP11−100E13.1
FAM27B
UBE2C
SMPD2
ARMC6
WDR12
NOCT
PMCH
MIR1244−2
RP11−399K21.11
ASF1B
APCDD1L−AS1
RIN1
TXN
GLO1
WDR77

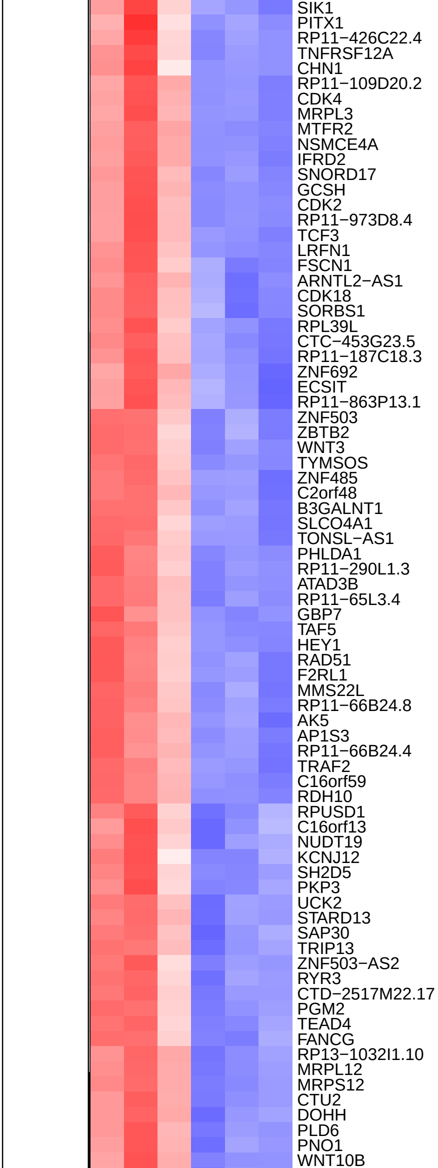
SIK1
PITX1
RP11−426C22.4
TNFRSF12A
CHN1
RP11−109D20.2
CDK4
MRPL3
MTFR2
NSMCE4A
IFRD2
SNORD17
GCSH
CDK2
RP11−973D8.4
TCF3
LRFN1
FSCN1
ARNTL2−AS1
CDK18
SORBS1
RPL39L
CTC−453G23.5
RP11−187C18.3
ZNF692
ECSIT
RP11−863P13.1
ZNF503
ZBTB2
WNT3
TYMSOS
ZNF485
C2orf48
B3GALNT1
SLCO4A1
TONSL−AS1
PHLDA1
RP11−290L1.3
ATAD3B
RP11−65L3.4
GBP7
TAF5
HEY1
RAD51
F2RL1
MMS22L
RP11−66B24.8
AK5
AP1S3
RP11−66B24.4
TRAF2
C16orf59
RDH10
RPUSD1
C16orf13
NUDT19
KCNJ12
SH2D5
PKP3
UCK2
STARD13
SAP30
TRIP13
ZNF503−AS2
RYR3
CTD−2517M22.17
PGM2
TEAD4
FANCG
RP13−1032I1.10
MRPL12
MRPS12
CTU2
DOHH
PLD6
PNO1
WNT10B

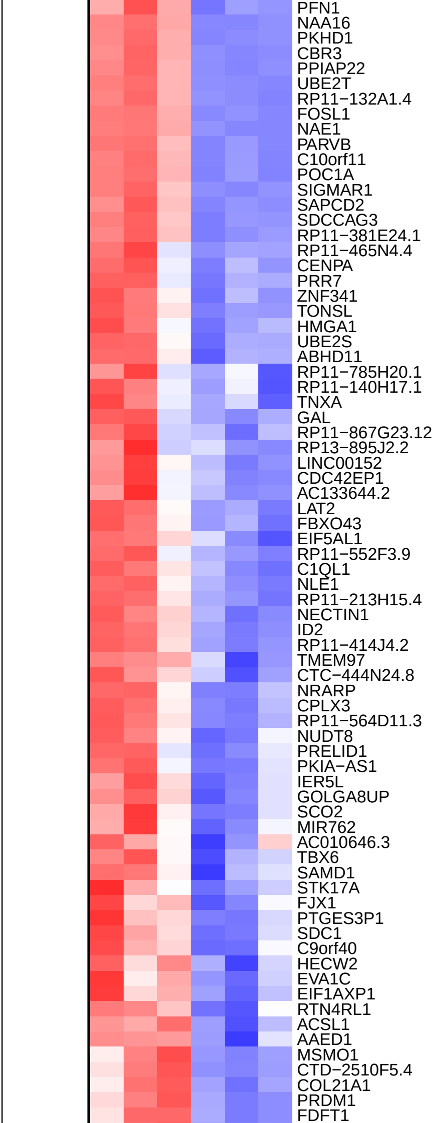
PFN1
NAA16
PKHD1
CBR3
PPIAP22
UBE2T
RP11−132A1.4
FOSL1
NAE1
PARVB
C10orf11
POC1A
SIGMAR1
SAPCD2
SDCCAG3
RP11−381E24.1
RP11−465N4.4
CENPA
PRR7
ZNF341
TONSL
HMGA1
UBE2S
ABHD11
RP11−785H20.1
RP11−140H17.1
TNXA
GAL
RP11−867G23.12
RP13−895J2.2
LINC00152
CDC42EP1
AC133644.2
LAT2
FBXO43
EIF5AL1
RP11−552F3.9
C1QL1
NLE1
RP11−213H15.4
NECTIN1
ID2
RP11−414J4.2
TMEM97
CTC−444N24.8
NRARP
CPLX3
RP11−564D11.3
NUDT8
PRELID1
PKIA−AS1
IER5L
GOLGA8UP
SCO2
MIR762
AC010646.3
TBX6
SAMD1
STK17A
FJX1
PTGES3P1
SDC1
C9orf40
HECW2
EVA1C
EIF1AXP1
RTN4RL1
ACSL1
AAED1
MSMO1
CTD−2510F5.4
COL21A1
PRDM1
FDFT1

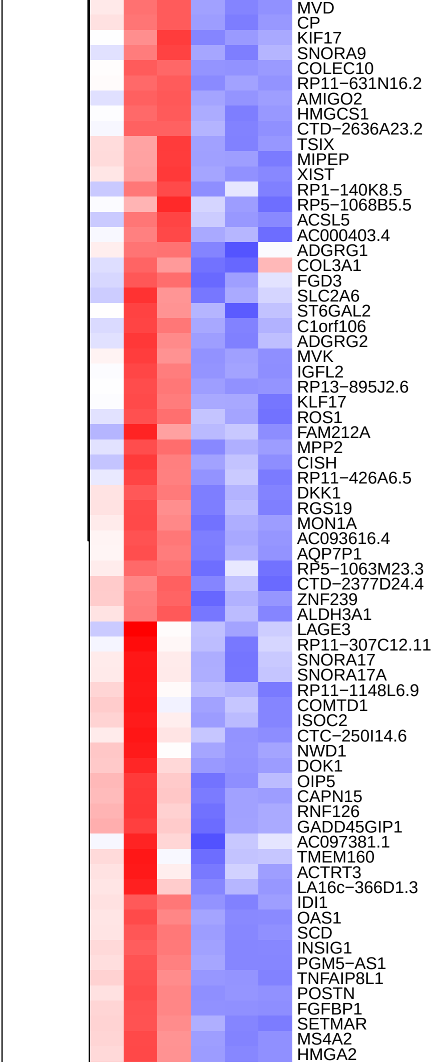
MVD
CP
KIF17
SNORA9
COLEC10
RP11−631N16.2
AMIGO2
HMGCS1
CTD−2636A23.2
TSIX
MIPEP
XIST
RP1−140K8.5
RP5−1068B5.5
ACSL5
AC000403.4
ADGRG1
COL3A1
FGD3
SLC2A6
ST6GAL2
C1orf106
ADGRG2
MVK
IGFL2
RP13−895J2.6
KLF17
ROS1
FAM212A
MPP2
CISH
RP11−426A6.5
DKK1
RGS19
MON1A
AC093616.4
AQP7P1
RP5−1063M23.3
CTD−2377D24.4
ZNF239
ALDH3A1
LAGE3
RP11−307C12.11
SNORA17
SNORA17A
RP11−1148L6.9
COMTD1
ISOC2
CTC−250I14.6
NWD1
DOK1
OIP5
CAPN15
RNF126
GADD45GIP1
AC097381.1
TMEM160
ACTRT3
LA16c−366D1.3
IDI1
OAS1
SCD
INSIG1
PGM5−AS1
TNFAIP8L1
POSTN
FGFBP1
SETMAR
MS4A2
HMGA2

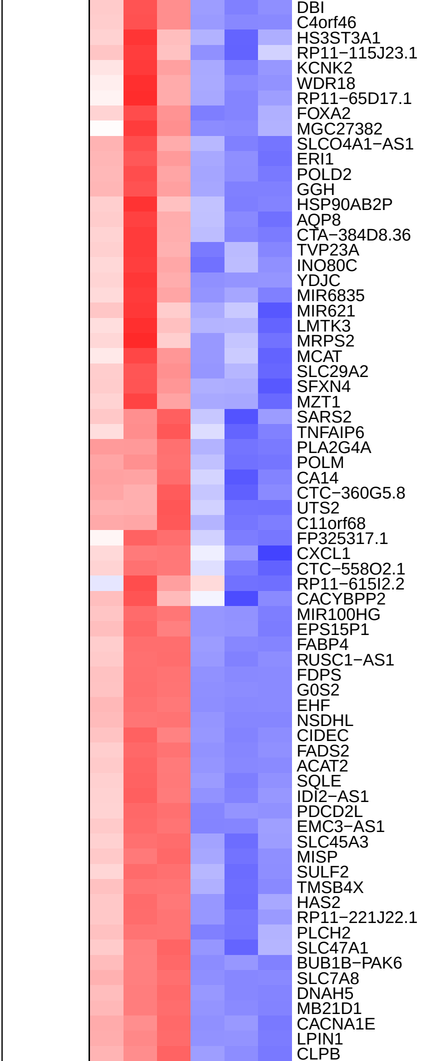
DBI
C4orf46
HS3ST3A1
RP11−115J23.1
KCNK2
WDR18
RP11−65D17.1
FOXA2
MGC27382
SLCO4A1−AS1
ERI1
POLD2
GGH
HSP90AB2P
AQP8
CTA−384D8.36
TVP23A
INO80C
YDJC
MIR6835
MIR621
LMTK3
MRPS2
MCAT
SLC29A2
SFXN4
MZT1
SARS2
TNFAIP6
PLA2G4A
POLM
CA14
CTC−360G5.8
UTS2
C11orf68
FP325317.1
CXCL1
CTC−558O2.1
RP11−615I2.2
CACYBPP2
MIR100HG
EPS15P1
FABP4
RUSC1−AS1
FDPS
G0S2
EHF
NSDHL
CIDEC
FADS2
ACAT2
SQLE
IDI2−AS1
PDCD2L
EMC3−AS1
SLC45A3
MISP
SULF2
TMSB4X
HAS2
RP11−221J22.1
PLCH2
SLC47A1
BUB1B−PAK6
SLC7A8
DNAH5
MB21D1
CACNA1E
LPIN1
CLPB

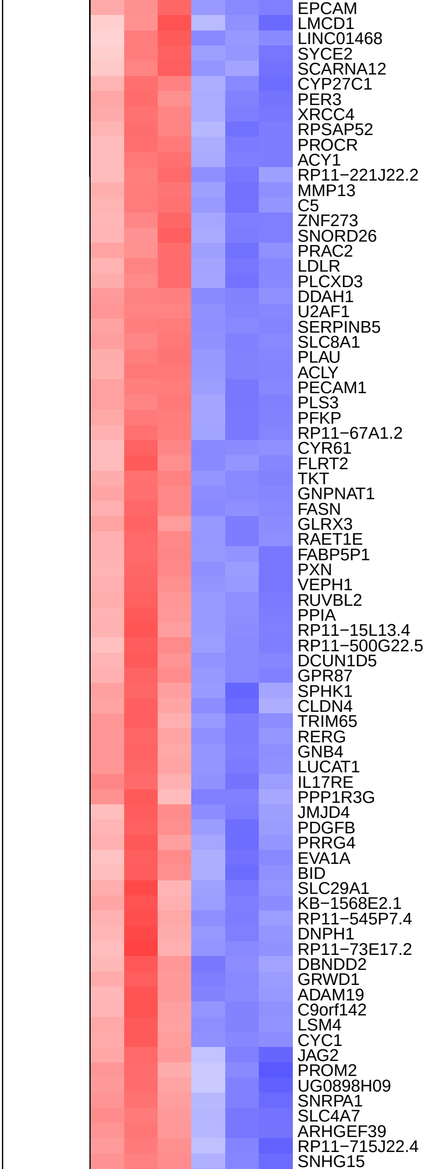
EPCAM
LMCD1
LINC01468
SYCE2
SCARNA12
CYP27C1
PER3
XRCC4
RPSAP52
PROCR
ACY1
RP11−221J22.2
MMP13
C5
ZNF273
SNORD26
PRAC2
LDLR
PLCXD3
DDAH1
U2AF1
SERPINB5
SLC8A1
PLAU
ACLY
PECAM1
PLS3
PFKP
RP11−67A1.2
CYR61
FLRT2
TKT
GNPNAT1
FASN
GLRX3
RAET1E
FABP5P1
PXN
VEPH1
RUVBL2
PPIA
RP11−15L13.4
RP11−500G22.5
DCUN1D5
GPR87
SPHK1
CLDN4
TRIM65
RERG
GNB4
LUCAT1
IL17RE
PPP1R3G
JMJD4
PDGFB
PRRG4
EVA1A
BID
SLC29A1
KB−1568E2.1
RP11−545P7.4
DNPH1
RP11−73E17.2
DBNDD2
GRWD1
ADAM19
C9orf142
LSM4
CYC1
JAG2
PROM2
UG0898H09
SNRPA1
SLC4A7
ARHGEF39
RP11−715J22.4
SNHG15

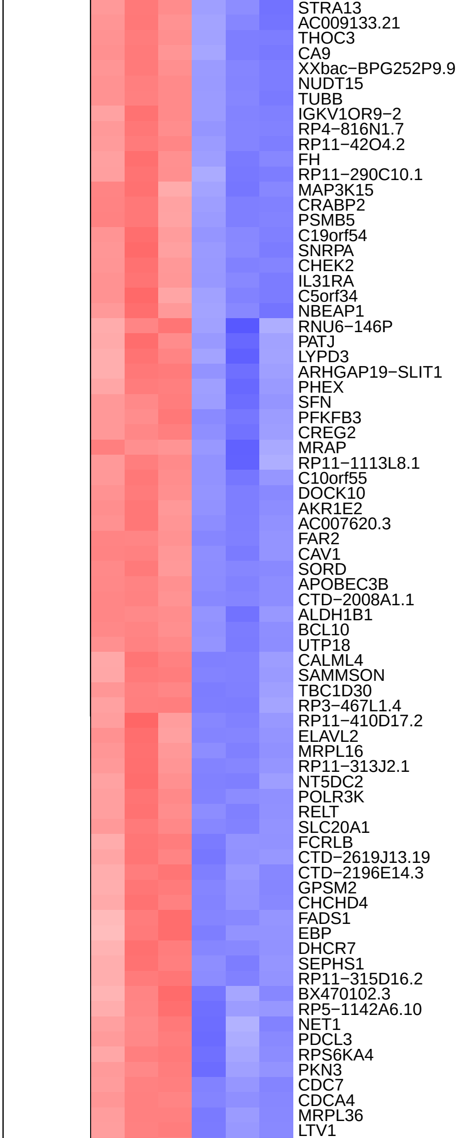
STRA13
AC009133.21
THOC3
CA9
XXbac−BPG252P9.9
NUDT15
TUBB
IGKV1OR9−2
RP4−816N1.7
RP11−42O4.2
FH
RP11−290C10.1
MAP3K15
CRABP2
PSMB5
C19orf54
SNRPA
CHEK2
IL31RA
C5orf34
NBEAP1
RNU6−146P
PATJ
LYPD3
ARHGAP19−SLIT1
PHEX
SFN
PFKFB3
CREG2
MRAP
RP11−1113L8.1
C10orf55
DOCK10
AKR1E2
AC007620.3
FAR2
CAV1
SORD
APOBEC3B
CTD−2008A1.1
ALDH1B1
BCL10
UTP18
CALML4
SAMMSON
TBC1D30
RP3−467L1.4
RP11−410D17.2
ELAVL2
MRPL16
RP11−313J2.1
NT5DC2
POLR3K
RELT
SLC20A1
FCRLB
CTD−2619J13.19
CTD−2196E14.3
GPSM2
CHCHD4
FADS1
EBP
DHCR7
SEPHS1
RP11−315D16.2
BX470102.3
RP5−1142A6.10
NET1
PDCL3
RPS6KA4
PKN3
CDC7
CDCA4
MRPL36
LTV1

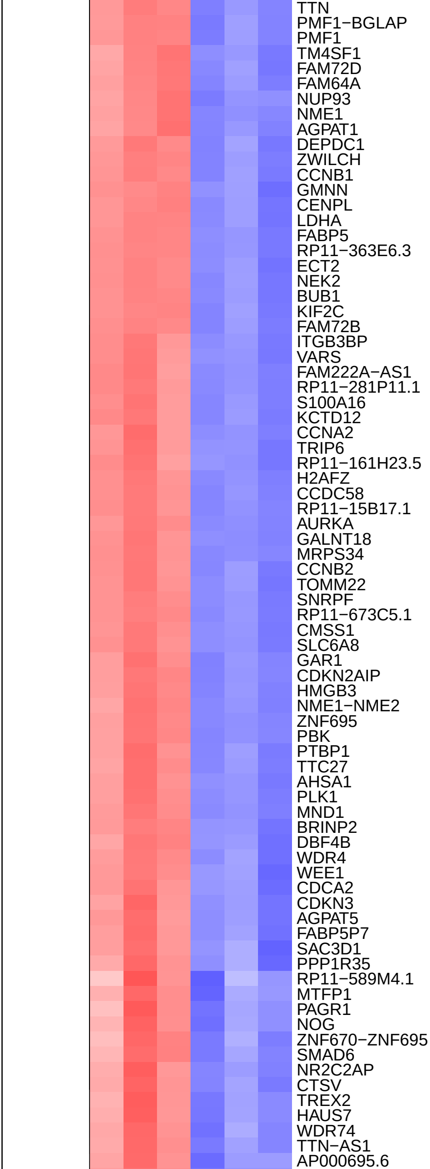
TTN
PMF1−BGLAP
PMF1
TM4SF1
FAM72D
FAM64A
NUP93
NME1
AGPAT1
DEPDC1
ZWILCH
CCNB1
GMNN
CENPL
LDHA
FABP5
RP11−363E6.3
ECT2
NEK2
BUB1
KIF2C
FAM72B
ITGB3BP
VARS
FAM222A−AS1
RP11−281P11.1
S100A16
KCTD12
CCNA2
TRIP6
RP11−161H23.5
H2AFZ
CCDC58
RP11−15B17.1
AURKA
GALNT18
MRPS34
CCNB2
TOMM22
SNRPF
RP11−673C5.1
CMSS1
SLC6A8
GAR1
CDKN2AIP
HMGB3
NME1−NME2
ZNF695
PBK
PTBP1
TTC27
AHSA1
PLK1
MND1
BRINP2
DBF4B
WDR4
WEE1
CDCA2
CDKN3
AGPAT5
FABP5P7
SAC3D1
PPP1R35
RP11−589M4.1
MTFP1
PAGR1
NOG
ZNF670−ZNF695
SMAD6
NR2C2AP
CTSV
TREX2
HAUS7
WDR74
TTN−AS1
AP000695.6

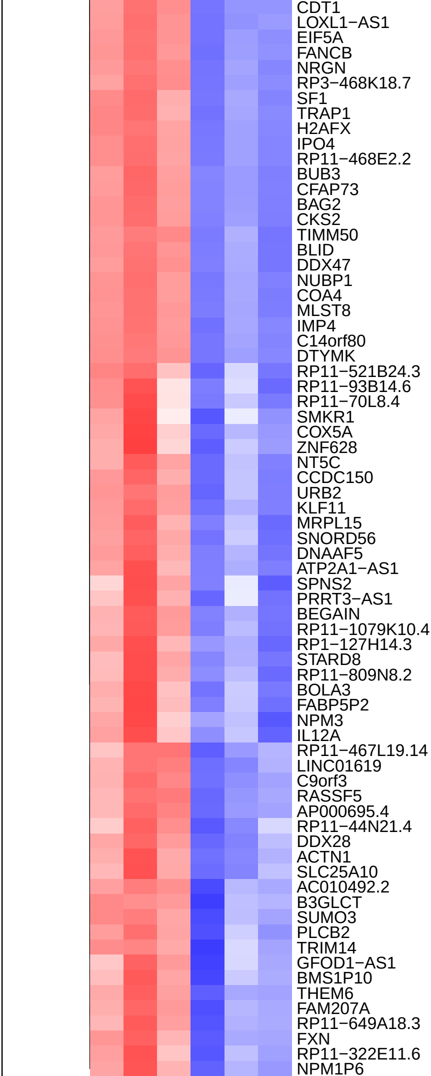
CDT1
LOXL1−AS1
EIF5A
FANCB
NRGN
RP3−468K18.7
SF1
TRAP1
H2AFX
IPO4
RP11−468E2.2
BUB3
CFAP73
BAG2
CKS2
TIMM50
BLID
DDX47
NUBP1
COA4
MLST8
IMP4
C14orf80
DTYMK
RP11−521B24.3
RP11−93B14.6
RP11−70L8.4
SMKR1
COX5A
ZNF628
NT5C
CCDC150
URB2
KLF11
MRPL15
SNORD56
DNAAF5
ATP2A1−AS1
SPNS2
PRRT3−AS1
BEGAIN
RP11−1079K10.4
RP1−127H14.3
STARD8
RP11−809N8.2
BOLA3
FABP5P2
NPM3
IL12A
RP11−467L19.14
LINC01619
C9orf3
RASSF5
AP000695.4
RP11−44N21.4
DDX28
ACTN1
SLC25A10
AC010492.2
B3GLCT
SUMO3
PLCB2
TRIM14
GFOD1−AS1
BMS1P10
THEM6
FAM207A
RP11−649A18.3
FXN
RP11−322E11.6
NPM1P6

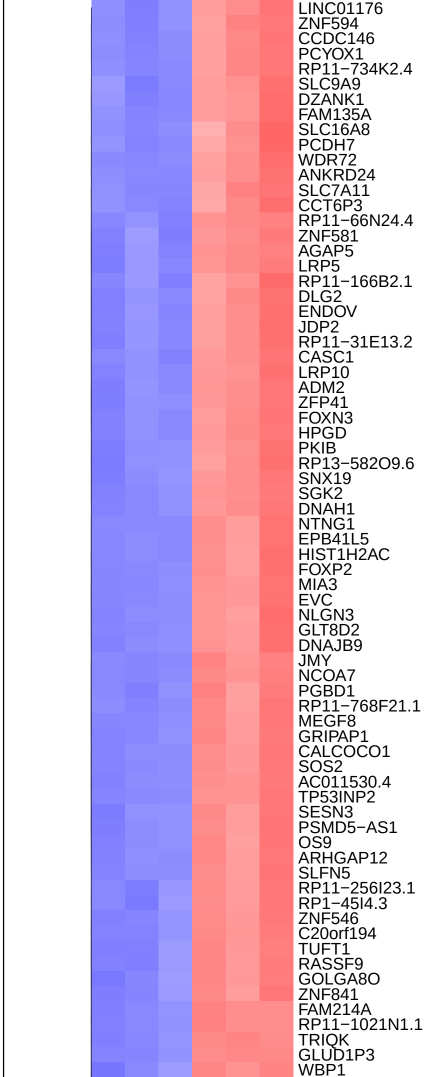
LINC01176
ZNF594
CCDC146
PCYOX1
RP11−734K2.4
SLC9A9
DZANK1
FAM135A
SLC16A8
PCDH7
WDR72
ANKRD24
SLC7A11
CCT6P3
RP11−66N24.4
ZNF581
AGAP5
LRP5
RP11−166B2.1
DLG2
ENDOV
JDP2
RP11−31E13.2
CASC1
LRP10
ADM2
ZFP41
FOXN3
HPGD
PKIB
RP13−582O9.6
SNX19
SGK2
DNAH1
NTNG1
EPB41L5
HIST1H2AC
FOXP2
MIA3
EVC
NLGN3
GLT8D2
DNAJB9
JMY
NCOA7
PGBD1
RP11−768F21.1
MEGF8
GRIPAP1
CALCOCO1
SOS2
AC011530.4
TP53INP2
SESN3
PSMD5−AS1
OS9
ARHGAP12
SLFN5
RP11−256I23.1
RP1−45I4.3
ZNF546
C20orf194
TUFT1
RASSF9
GOLGA8O
ZNF841
FAM214A
RP11−1021N1.1
TRIQK
GLUD1P3
WBP1

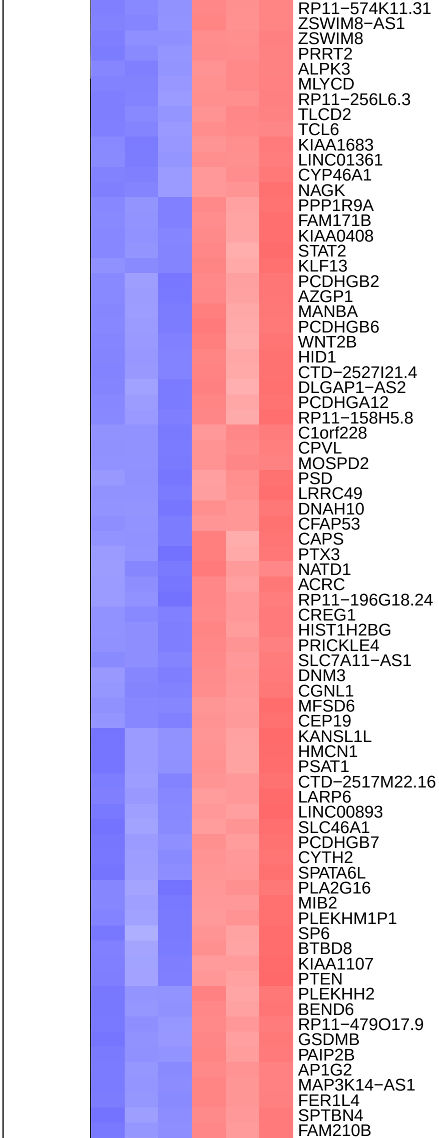
RP11−574K11.31
ZSWIM8−AS1
ZSWIM8
PRRT2
ALPK3
MLYCD
RP11−256L6.3
TLCD2
TCL6
KIAA1683
LINC01361
CYP46A1
NAGK
PPP1R9A
FAM171B
KIAA0408
STAT2
KLF13
PCDHGB2
AZGP1
MANBA
PCDHGB6
WNT2B
HID1
CTD−2527I21.4
DLGAP1−AS2
PCDHGA12
RP11−158H5.8
C1orf228
CPVL
MOSPD2
PSD
LRRC49
DNAH10
CFAP53
CAPS
PTX3
NATD1
ACRC
RP11−196G18.24
CREG1
HIST1H2BG
PRICKLE4
SLC7A11−AS1
DNM3
CGNL1
MFSD6
CEP19
KANSL1L
HMCN1
PSAT1
CTD−2517M22.16
LARP6
LINC00893
SLC46A1
PCDHGB7
CYTH2
SPATA6L
PLA2G16
MIB2
PLEKHM1P1
SP6
BTBD8
KIAA1107
PTEN
PLEKHH2
BEND6
RP11−479O17.9
GSDMB
PAIP2B
AP1G2
MAP3K14−AS1
FER1L4
SPTBN4
FAM210B

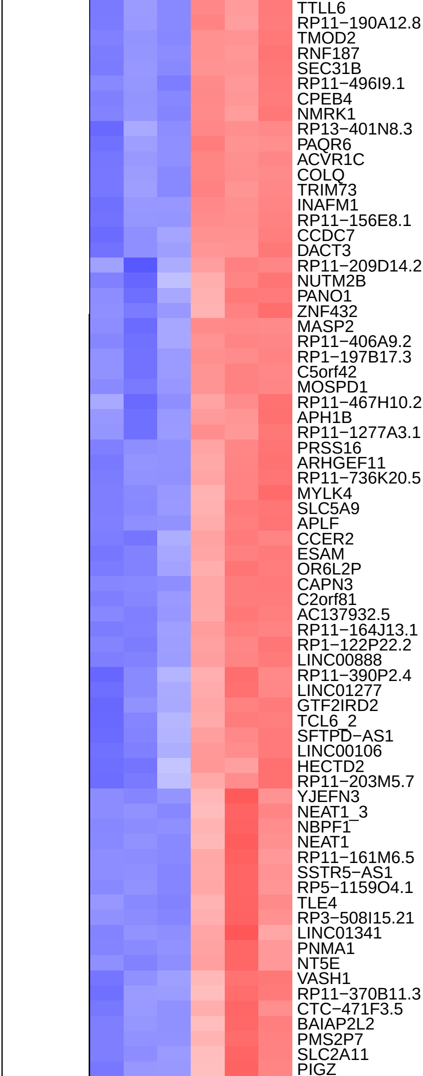
TTLL6
RP11−190A12.8
TMOD2
RNF187
SEC31B
RP11−496I9.1
CPEB4
NMRK1
RP13−401N8.3
PAQR6
ACVR1C
COLQ
TRIM73
INAFM1
RP11−156E8.1
CCDC7
DACT3
RP11−209D14.2
NUTM2B
PANO1
ZNF432
MASP2
RP11−406A9.2
RP1−197B17.3
C5orf42
MOSPD1
RP11−467H10.2
APH1B
RP11−1277A3.1
PRSS16
ARHGEF11
RP11−736K20.5
MYLK4
SLC5A9
APLF
CCER2
ESAM
OR6L2P
CAPN3
C2orf81
AC137932.5
RP11−164J13.1
RP1−122P22.2
LINC00888
RP11−390P2.4
LINC01277
GTF2IRD2
TCL6_2
SFTPD−AS1
LINC00106
HECTD2
RP11−203M5.7
YJEFN3
NEAT1_3
NBPF1
NEAT1
RP11−161M6.5
SSTR5−AS1
RP5−1159O4.1
TLE4
RP3−508I15.21
LINC01341
PNMA1
NT5E
VASH1
RP11−370B11.3
CTC−471F3.5
BAIAP2L2
PMS2P7
SLC2A11
PIGZ

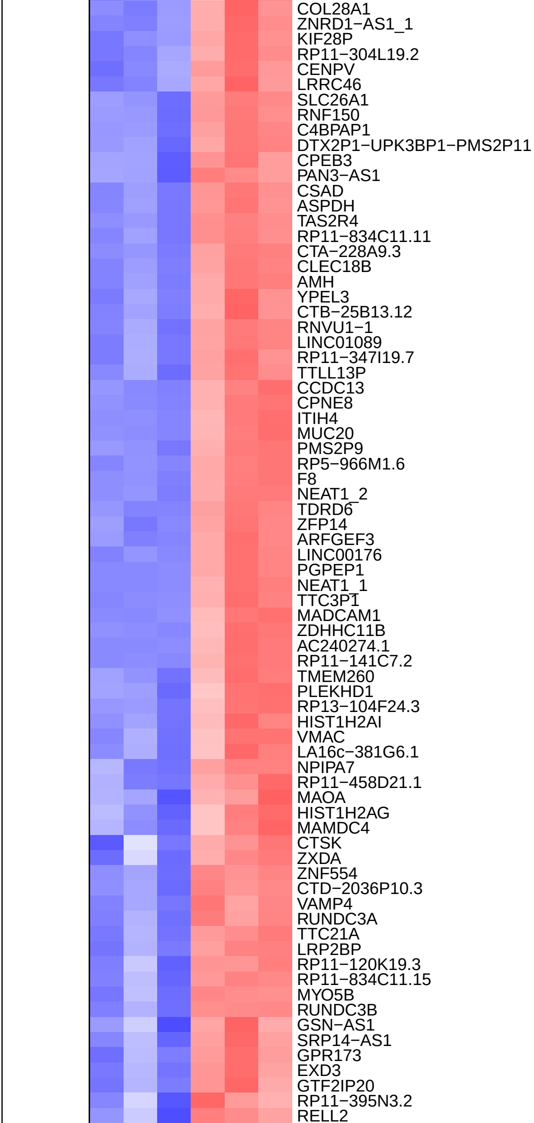

COL28A1
ZNRD1−AS1_1
KIF28P
RP11−304L19.2
CENPV
LRRC46
SLC26A1
RNF150
C4BPAP1
DTX2P1−UPK3BP1−PMS2P11
CPEB3
PAN3−AS1
CSAD
ASPDH
TAS2R4
RP11−834C11.11
CTA−228A9.3
CLEC18B
AMH
YPEL3
CTB−25B13.12
RNVU1−1
LINC01089
RP11−347I19.7
TTLL13P
CCDC13
CPNE8
ITIH4
MUC20
PMS2P9
RP5−966M1.6
F8
NEAT1_2
TDRD6
ZFP14
ARFGEF3
LINC00176
PGPEP1
NEAT1_1
TTC3P1
MADCAM1
ZDHHC11B
AC240274.1
RP11−141C7.2
TMEM260
PLEKHD1
RP13−104F24.3
HIST1H2AI
VMAC
LA16c−381G6.1
NPIPA7
RP11−458D21.1
MAOA
HIST1H2AG
MAMDC4
CTSK
ZXDA
ZNF554
CTD−2036P10.3
VAMP4
RUNDC3A
TTC21A
LRP2BP
RP11−120K19.3
RP11−834C11.15
MYO5B
RUNDC3B
GSN−AS1
SRP14−AS1
GPR173
EXD3
GTF2IP20
RP11−395N3.2
RELL2

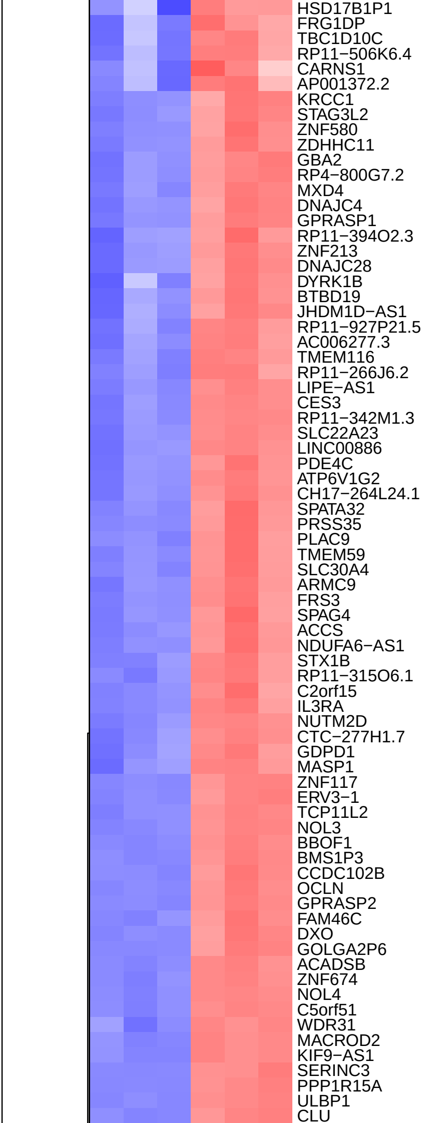
HSD17B1P1
FRG1DP
TBC1D10C
RP11−506K6.4
CARNS1
AP001372.2
KRCC1
STAG3L2
ZNF580
ZDHHC11
GBA2
RP4−800G7.2
MXD4
DNAJC4
GPRASP1
RP11−394O2.3
ZNF213
DNAJC28
DYRK1B
BTBD19
JHDM1D−AS1
RP11−927P21.5
AC006277.3
TMEM116
RP11−266J6.2
LIPE−AS1
CES3
RP11−342M1.3
SLC22A23
LINC00886
PDE4C
ATP6V1G2
CH17−264L24.1
SPATA32
PRSS35
PLAC9
TMEM59
SLC30A4
ARMC9
FRS3
SPAG4
ACCS
NDUFA6−AS1
STX1B
RP11−315O6.1
C2orf15
IL3RA
NUTM2D
CTC−277H1.7
GDPD1
MASP1
ZNF117
ERV3−1
TCP11L2
NOL3
BBOF1
BMS1P3
CCDC102B
OCLN
GPRASP2
FAM46C
DXO
GOLGA2P6
ACADSB
ZNF674
NOL4
C5orf51
WDR31
MACROD2
KIF9−AS1
SERINC3
PPP1R15A
ULBP1
CLU

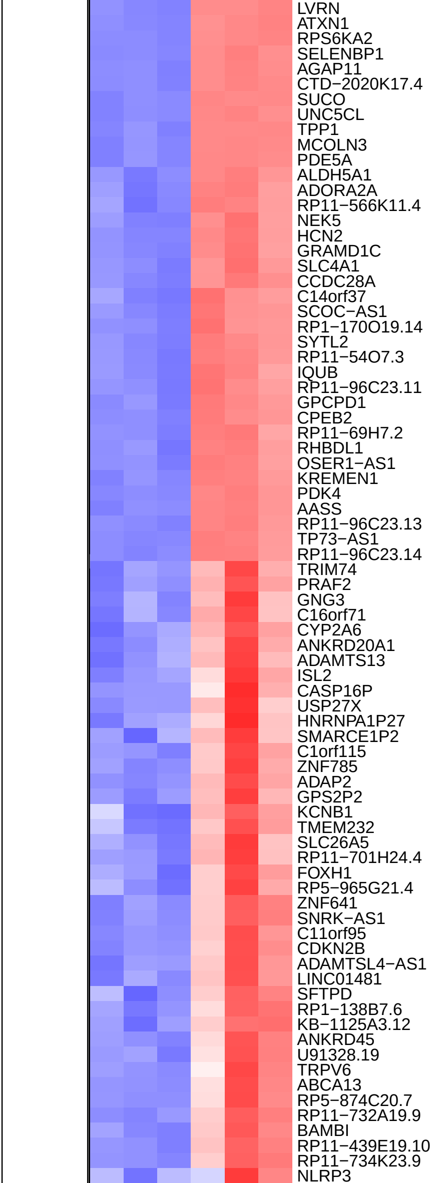
LVRN
ATXN1
RPS6KA2
SELENBP1
AGAP11
CTD−2020K17.4
SUCO
UNC5CL
TPP1
MCOLN3
PDE5A
ALDH5A1
ADORA2A
RP11−566K11.4
NEK5
HCN2
GRAMD1C
SLC4A1
CCDC28A
C14orf37
SCOC−AS1
RP1−170O19.14
SYTL2
RP11−54O7.3
IQUB
RP11−96C23.11
GPCPD1
CPEB2
RP11−69H7.2
RHBDL1
OSER1−AS1
KREMEN1
PDK4
AASS
RP11−96C23.13
TP73−AS1
RP11−96C23.14
TRIM74
PRAF2
GNG3
C16orf71
CYP2A6
ANKRD20A1
ADAMTS13
ISL2
CASP16P
USP27X
HNRNPA1P27
SMARCE1P2
C1orf115
ZNF785
ADAP2
GPS2P2
KCNB1
TMEM232
SLC26A5
RP11−701H24.4
FOXH1
RP5−965G21.4
ZNF641
SNRK−AS1
C11orf95
CDKN2B
ADAMTSL4−AS1
LINC01481
SFTPD
RP1−138B7.6
KB−1125A3.12
ANKRD45
U91328.19
TRPV6
ABCA13
RP5−874C20.7
RP11−732A19.9
BAMBI
RP11−439E19.10
RP11−734K23.9
NLRP3

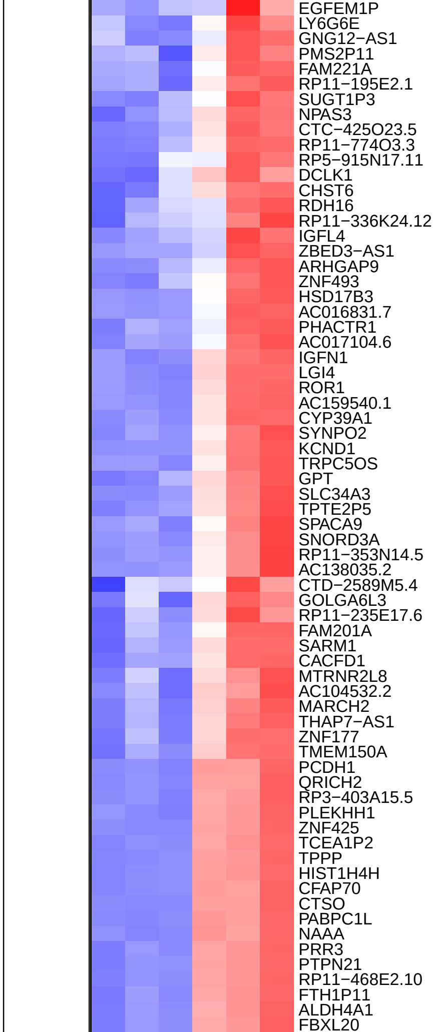
EGFEM1P
LY6G6E
GNG12−AS1
PMS2P11
FAM221A
RP11−195E2.1
SUGT1P3
NPAS3
CTC−425O23.5
RP11−774O3.3
RP5−915N17.11
DCLK1
CHST6
RDH16
RP11−336K24.12
IGFL4
ZBED3−AS1
ARHGAP9
ZNF493
HSD17B3
AC016831.7
PHACTR1
AC017104.6
IGFN1
LGI4
ROR1
AC159540.1
CYP39A1
SYNPO2
KCND1
TRPC5OS
GPT
SLC34A3
TPTE2P5
SPACA9
SNORD3A
RP11−353N14.5
AC138035.2
CTD−2589M5.4
GOLGA6L3
RP11−235E17.6
FAM201A
SARM1
CACFD1
MTRNR2L8
AC104532.2
MARCH2
THAP7−AS1
ZNF177
TMEM150A
PCDH1
QRICH2
RP3−403A15.5
PLEKHH1
ZNF425
TCEA1P2
TPPP
HIST1H4H
CFAP70
CTSO
PABPC1L
NAAA
PRR3
PTPN21
RP11−468E2.10
FTH1P11
ALDH4A1
FBXL20

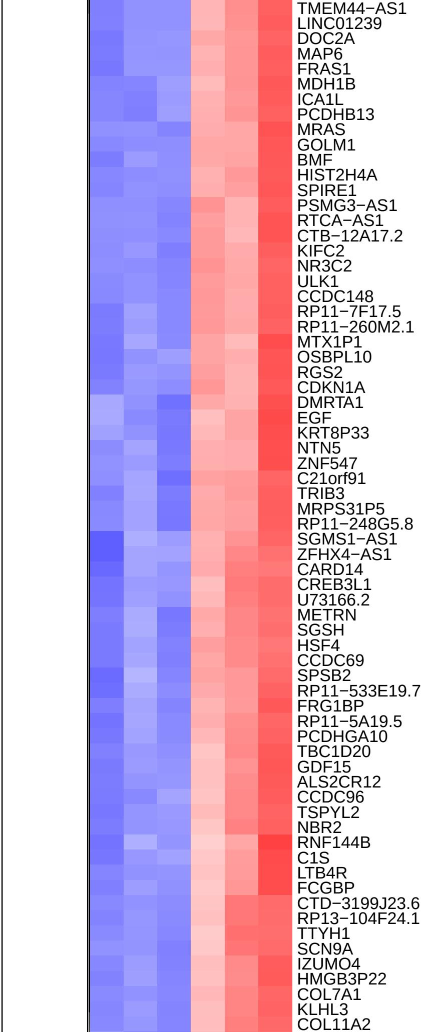
TMEM44−AS1
LINC01239
DOC2A
MAP6
FRAS1
MDH1B
ICA1L
PCDHB13
MRAS
GOLM1
BMF
HIST2H4A
SPIRE1
PSMG3−AS1
RTCA−AS1
CTB−12A17.2
KIFC2
NR3C2
ULK1
CCDC148
RP11−7F17.5
RP11−260M2.1
MTX1P1
OSBPL10
RGS2
CDKN1A
DMRTA1
EGF
KRT8P33
NTN5
ZNF547
C21orf91
TRIB3
MRPS31P5
RP11−248G5.8
SGMS1−AS1
ZFHX4−AS1
CARD14
CREB3L1
U73166.2
METRN
SGSH
HSF4
CCDC69
SPSB2
RP11−533E19.7
FRG1BP
RP11−5A19.5
PCDHGA10
TBC1D20
GDF15
ALS2CR12
CCDC96
TSPYL2
NBR2
RNF144B
C1S
LTB4R
FCGBP
CTD−3199J23.6
RP13−104F24.1
TTYH1
SCN9A
IZUMO4
HMGB3P22
COL7A1
KLHL3
COL11A2

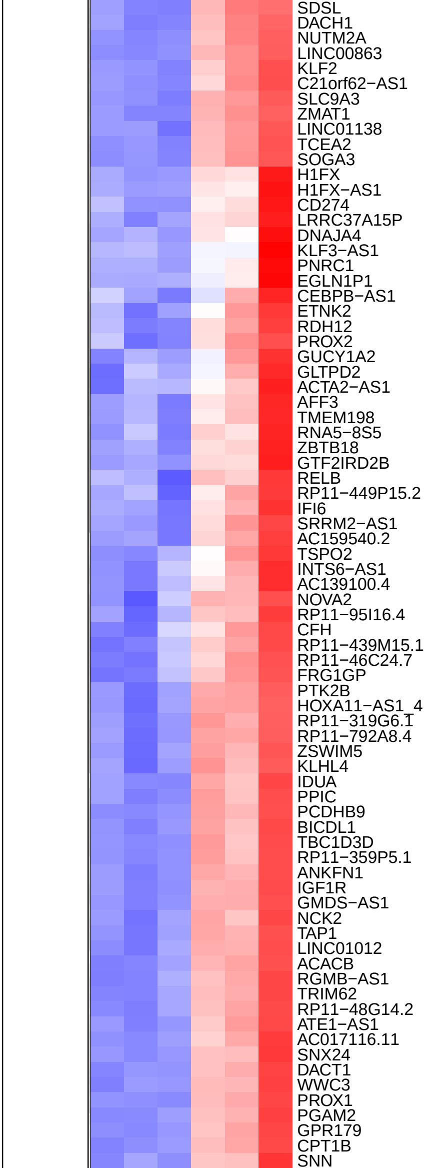
SDSL
DACH1
NUTM2A
LINC00863
KLF2
C21orf62−AS1
SLC9A3
ZMAT1
LINC01138
TCEA2
SOGA3
H1FX
H1FX−AS1
CD274
LRRC37A15P
DNAJA4
KLF3−AS1
PNRC1
EGLN1P1
CEBPB−AS1
ETNK2
RDH12
PROX2
GUCY1A2
GLTPD2
ACTA2−AS1
AFF3
TMEM198
RNA5−8S5
ZBTB18
GTF2IRD2B
RELB
RP11−449P15.2
IFI6
SRRM2−AS1
AC159540.2
TSPO2
INTS6−AS1
AC139100.4
NOVA2
RP11−95I16.4
CFH
RP11−439M15.1
RP11−46C24.7
FRG1GP
PTK2B
HOXA11−AS1_4
RP11−319G6.1
RP11−792A8.4
ZSWIM5
KLHL4
IDUA
PPIC
PCDHB9
BICDL1
TBC1D3D
RP11−359P5.1
ANKFN1
IGF1R
GMDS−AS1
NCK2
TAP1
LINC01012
ACACB
RGMB−AS1
TRIM62
RP11−48G14.2
ATE1−AS1
AC017116.11
SNX24
DACT1
WWC3
PROX1
PGAM2
GPR179
CPT1B
SNN

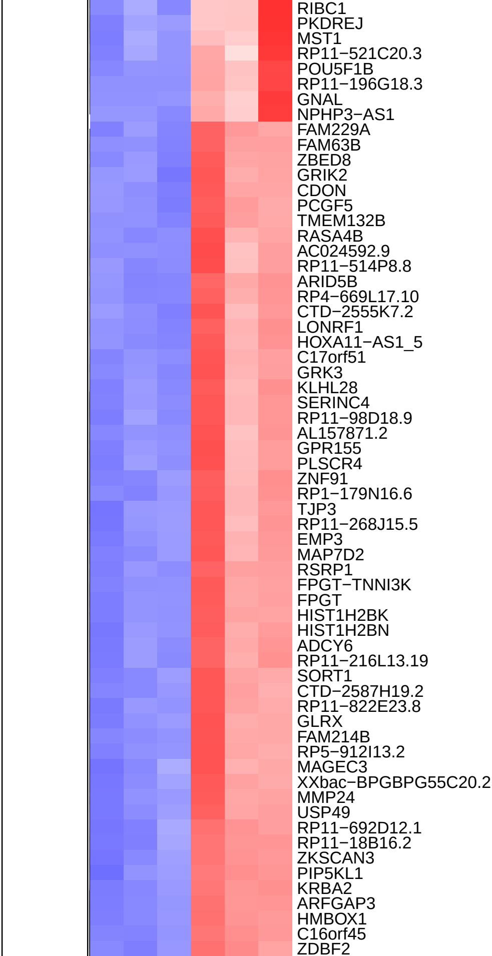
RIBC1
PKDREJ
MST1
RP11−521C20.3
POU5F1B
RP11−196G18.3
GNAL
NPHP3−AS1
FAM229A
FAM63B
ZBED8
GRIK2
CDON
PCGF5
TMEM132B
RASA4B
AC024592.9
RP11−514P8.8
ARID5B
RP4−669L17.10
CTD−2555K7.2
LONRF1
HOXA11−AS1_5
C17orf51
GRK3
KLHL28
SERINC4
RP11−98D18.9
AL157871.2
GPR155
PLSCR4
ZNF91
RP1−179N16.6
TJP3
RP11−268J15.5
EMP3
MAP7D2
RSRP1
FPGT−TNNI3K
FPGT
HIST1H2BK
HIST1H2BN
ADCY6
RP11−216L13.19
SORT1
CTD−2587H19.2
RP11−822E23.8
GLRX
FAM214B
RP5−912I13.2
MAGEC3
XXbac−BPGBPG55C20.2
MMP24
USP49
RP11−692D12.1
RP11−18B16.2
ZKSCAN3
PIP5KL1
KRBA2
ARFGAP3
HMBOX1
C16orf45
ZDBF2

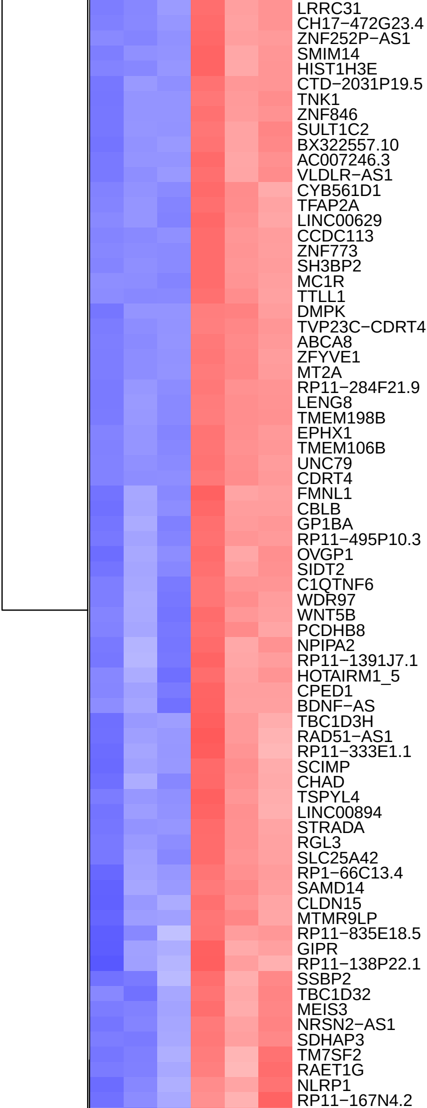
LRRC31
CH17−472G23.4
ZNF252P−AS1
SMIM14
HIST1H3E
CTD−2031P19.5
TNK1
ZNF846
SULT1C2
BX322557.10
AC007246.3
VLDLR−AS1
CYB561D1
TFAP2A
LINC00629
CCDC113
ZNF773
SH3BP2
MC1R
TTLL1
DMPK
TVP23C−CDRT4
ABCA8
ZFYVE1
MT2A
RP11−284F21.9
LENG8
TMEM198B
EPHX1
TMEM106B
UNC79
CDRT4
FMNL1
CBLB
GP1BA
RP11−495P10.3
OVGP1
SIDT2
C1QTNF6
WDR97
WNT5B
PCDHB8
NPIPA2
RP11−1391J7.1
HOTAIRM1_5
CPED1
BDNF−AS
TBC1D3H
RAD51−AS1
RP11−333E1.1
SCIMP
CHAD
TSPYL4
LINC00894
STRADA
RGL3
SLC25A42
RP1−66C13.4
SAMD14
CLDN15
MTMR9LP
RP11−835E18.5
GIPR
RP11−138P22.1
SSBP2
TBC1D32
MEIS3
NRSN2−AS1
SDHAP3
TM7SF2
RAET1G
NLRP1
RP11−167N4.2

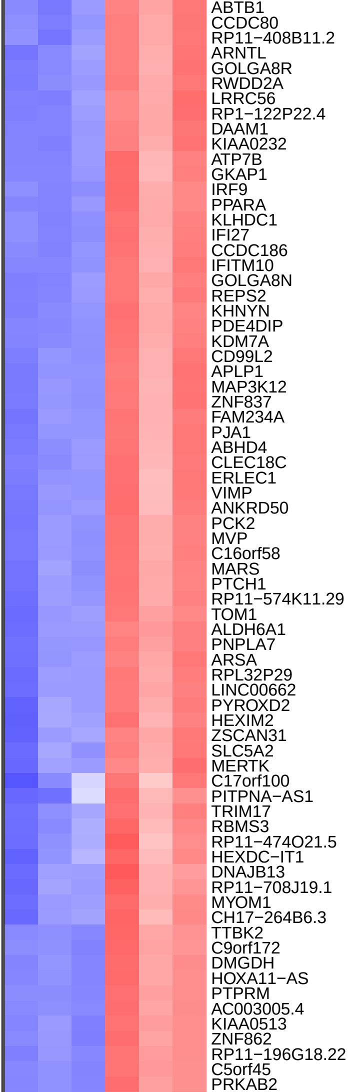
ABTB1
CCDC80
RP11−408B11.2
ARNTL
GOLGA8R
RWDD2A
LRRC56
RP1−122P22.4
DAAM1
KIAA0232
ATP7B
GKAP1
IRF9
PPARA
KLHDC1
IFI27
CCDC186
IFITM10
GOLGA8N
REPS2
KHNYN
PDE4DIP
KDM7A
CD99L2
APLP1
MAP3K12
ZNF837
FAM234A
PJA1
ABHD4
CLEC18C
ERLEC1
VIMP
ANKRD50
PCK2
MVP
C16orf58
MARS
PTCH1
RP11−574K11.29
TOM1
ALDH6A1
PNPLA7
ARSA
RPL32P29
LINC00662
PYROXD2
HEXIM2
ZSCAN31
SLC5A2
MERTK
C17orf100
PITPNA−AS1
TRIM17
RBMS3
RP11−474O21.5
HEXDC−IT1
DNAJB13
RP11−708J19.1
MYOM1
CH17−264B6.3
TTBK2
C9orf172
DMGDH
HOXA11−AS
PTPRM
AC003005.4
KIAA0513
ZNF862
RP11−196G18.22
C5orf45
PRKAB2

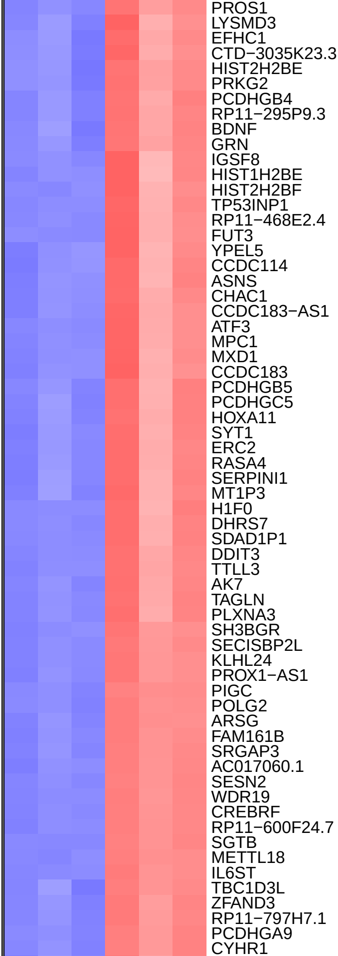
PROS1
LYSMD3
EFHC1
CTD−3035K23.3
HIST2H2BE
PRKG2
PCDHGB4
RP11−295P9.3
BDNF
GRN
IGSF8
HIST1H2BE
HIST2H2BF
TP53INP1
RP11−468E2.4
FUT3
YPEL5
CCDC114
ASNS
CHAC1
CCDC183−AS1
ATF3
MPC1
MXD1
CCDC183
PCDHGB5
PCDHGC5
HOXA11
SYT1
ERC2
RASA4
SERPINI1
MT1P3
H1F0
DHRS7
SDAD1P1
DDIT3
TTLL3
AK7
TAGLN
PLXNA3
SH3BGR
SECISBP2L
KLHL24
PROX1−AS1
PIGC
POLG2
ARSG
FAM161B
SRGAP3
AC017060.1
SESN2
WDR19
CREBRF
RP11−600F24.7
SGTB
METTL18
IL6ST
TBC1D3L
ZFAND3
RP11−797H7.1
PCDHGA9
CYHR1

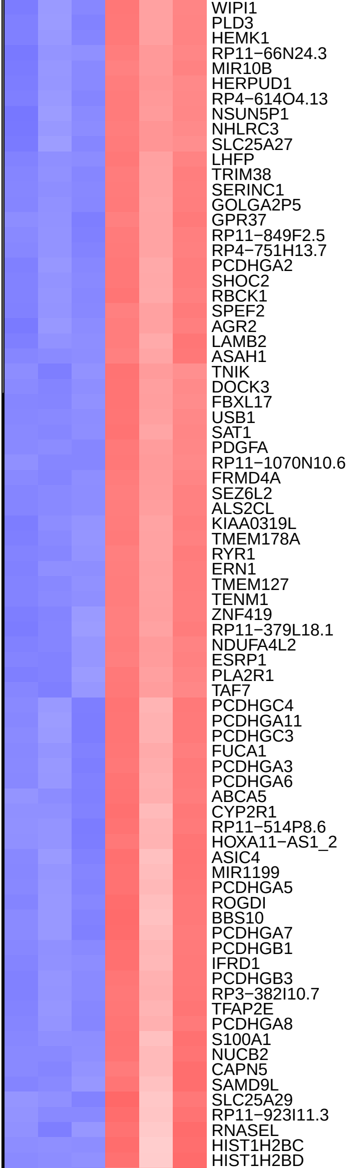
WIPI1
PLD3
HEMK1
RP11−66N24.3
MIR10B
HERPUD1
RP4−614O4.13
NSUN5P1
NHLRC3
SLC25A27
LHFP
TRIM38
SERINC1
GOLGA2P5
GPR37
RP11−849F2.5
RP4−751H13.7
PCDHGA2
SHOC2
RBCK1
SPEF2
AGR2
LAMB2
ASAH1
TNIK
DOCK3
FBXL17
USB1
SAT1
PDGFA
RP11−1070N10.6
FRMD4A
SEZ6L2
ALS2CL
KIAA0319L
TMEM178A
RYR1
ERN1
TMEM127
TENM1
ZNF419
RP11−379L18.1
NDUFA4L2
ESRP1
PLA2R1
TAF7
PCDHGC4
PCDHGA11
PCDHGC3
FUCA1
PCDHGA3
PCDHGA6
ABCA5
CYP2R1
RP11−514P8.6
HOXA11−AS1_2
ASIC4
MIR1199
PCDHGA5
ROGDI
BBS10
PCDHGA7
PCDHGB1
IFRD1
PCDHGB3
RP3−382I10.7
TFAP2E
PCDHGA8
S100A1
NUCB2
CAPN5
SAMD9L
SLC25A29
RP11−923I11.3
RNASEL
HIST1H2BC
HIST1H2BD

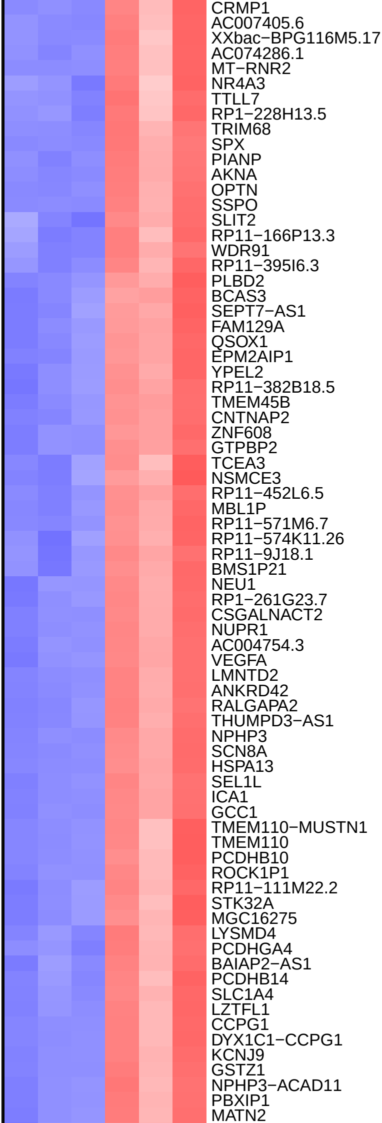
CRMP1
AC007405.6
XXbac−BPG116M5.17
AC074286.1
MT−RNR2
NR4A3
TTLL7
RP1−228H13.5
TRIM68
SPX
PIANP
AKNA
OPTN
SSPO
SLIT2
RP11−166P13.3
WDR91
RP11−395I6.3
PLBD2
BCAS3
SEPT7−AS1
FAM129A
QSOX1
EPM2AIP1
YPEL2
RP11−382B18.5
TMEM45B
CNTNAP2
ZNF608
GTPBP2
TCEA3
NSMCE3
RP11−452L6.5
MBL1P
RP11−571M6.7
RP11−574K11.26
RP11−9J18.1
BMS1P21
NEU1
RP1−261G23.7
CSGALNACT2
NUPR1
AC004754.3
VEGFA
LMNTD2
ANKRD42
RALGAPA2
THUMPD3−AS1
NPHP3
SCN8A
HSPA13
SEL1L
ICA1
GCC1
TMEM110−MUSTN1
TMEM110
PCDHB10
ROCK1P1
RP11−111M22.2
STK32A
MGC16275
LYSMD4
PCDHGA4
BAIAP2−AS1
PCDHB14
SLC1A4
LZTFL1
CCPG1
DYX1C1−CCPG1
KCNJ9
GSTZ1
NPHP3−ACAD11
PBXIP1
MATN2

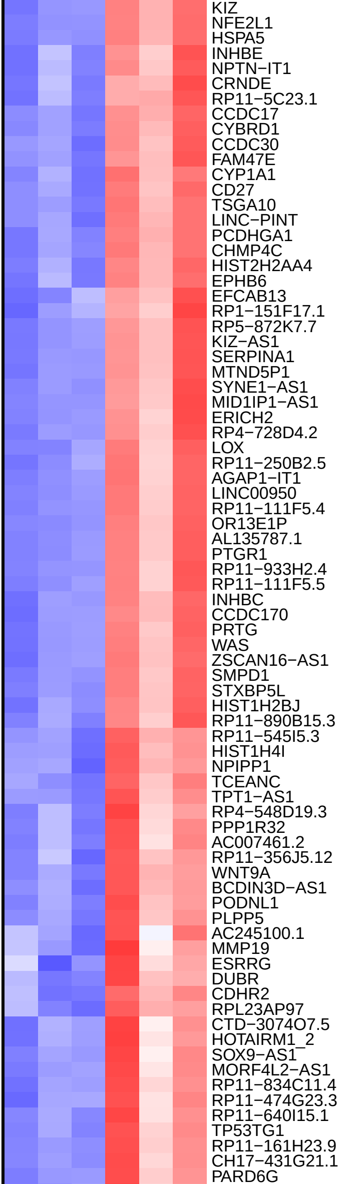
KIZ
NFE2L1
HSPA5
INHBE
NPTN−IT1
CRNDE
RP11−5C23.1
CCDC17
CYBRD1
CCDC30
FAM47E
CYP1A1
CD27
TSGA10
LINC−PINT
PCDHGA1
CHMP4C
HIST2H2AA4
EPHB6
EFCAB13
RP1−151F17.1
RP5−872K7.7
KIZ−AS1
SERPINA1
MTND5P1
SYNE1−AS1
MID1IP1−AS1
ERICH2
RP4−728D4.2
LOX
RP11−250B2.5
AGAP1−IT1
LINC00950
RP11−111F5.4
OR13E1P
AL135787.1
PTGR1
RP11−933H2.4
RP11−111F5.5
INHBC
CCDC170
PRTG
WAS
ZSCAN16−AS1
SMPD1
STXBP5L
HIST1H2BJ
RP11−890B15.3
RP11−545I5.3
HIST1H4I
NPIPP1
TCEANC
TPT1−AS1
RP4−548D19.3
PPP1R32
AC007461.2
RP11−356J5.12
WNT9A
BCDIN3D−AS1
PODNL1
PLPP5
AC245100.1
MMP19
ESRRG
DUBR
CDHR2
RPL23AP97
CTD−3074O7.5
HOTAIRM1_2
SOX9−AS1
MORF4L2−AS1
RP11−834C11.4
RP11−474G23.3
RP11−640I15.1
TP53TG1
RP11−161H23.9
CH17−431G21.1
PARD6G

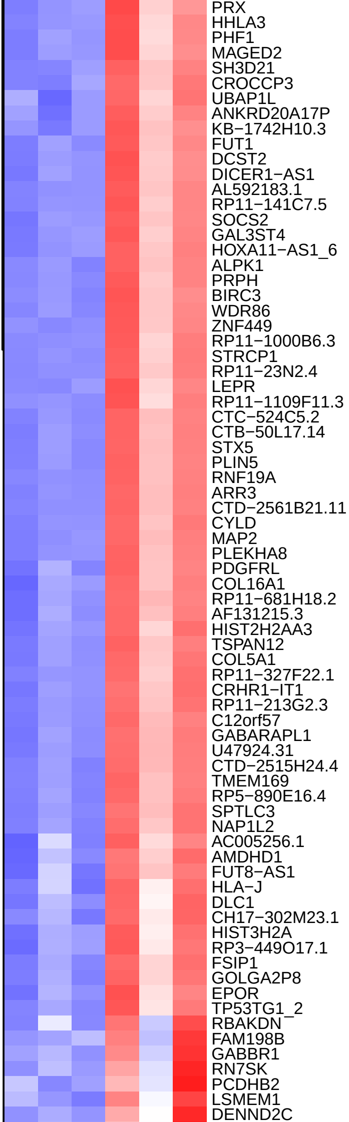
PRX
HHLA3
PHF1
MAGED2
SH3D21
CROCCP3
UBAP1L
ANKRD20A17P
KB−1742H10.3
FUT1
DCST2
DICER1−AS1
AL592183.1
RP11−141C7.5
SOCS2
GAL3ST4
HOXA11−AS1_6
ALPK1
PRPH
BIRC3
WDR86
ZNF449
RP11−1000B6.3
STRCP1
RP11−23N2.4
LEPR
RP11−1109F11.3
CTC−524C5.2
CTB−50L17.14
STX5
PLIN5
RNF19A
ARR3
CTD−2561B21.11
CYLD
MAP2
PLEKHA8
PDGFRL
COL16A1
RP11−681H18.2
AF131215.3
HIST2H2AA3
TSPAN12
COL5A1
RP11−327F22.1
CRHR1−IT1
RP11−213G2.3
C12orf57
GABARAPL1
U47924.31
CTD−2515H24.4
TMEM169
RP5−890E16.4
SPTLC3
NAP1L2
AC005256.1
AMDHD1
FUT8−AS1
HLA−J
DLC1
CH17−302M23.1
HIST3H2A
RP3−449O17.1
FSIP1
GOLGA2P8
EPOR
TP53TG1_2
RBAKDN
FAM198B
GABBR1
RN7SK
PCDHB2
LSMEM1
DENND2C

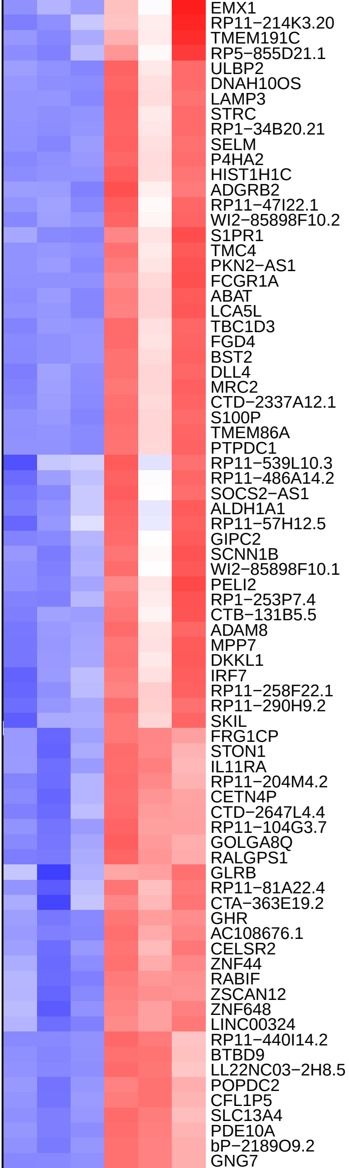
EMX1
RP11−214K3.20
TMEM191C
RP5−855D21.1
ULBP2
DNAH10OS
LAMP3
STRC
RP1−34B20.21
SELM
P4HA2
HIST1H1C
ADGRB2
RP11−47I22.1
WI2−85898F10.2
S1PR1
TMC4
PKN2−AS1
FCGR1A
ABAT
LCA5L
TBC1D3
FGD4
BST2
DLL4
MRC2
CTD−2337A12.1
S100P
TMEM86A
PTPDC1
RP11−539L10.3
RP11−486A14.2
SOCS2−AS1
ALDH1A1
RP11−57H12.5
GIPC2
SCNN1B
WI2−85898F10.1
PELI2
RP1−253P7.4
CTB−131B5.5
ADAM8
MPP7
DKKL1
IRF7
RP11−258F22.1
RP11−290H9.2
SKIL
FRG1CP
STON1
IL11RA
RP11−204M4.2
CETN4P
CTD−2647L4.4
RP11−104G3.7
GOLGA8Q
RALGPS1
GLRB
RP11−81A22.4
CTA−363E19.2
GHR
AC108676.1
CELSR2
ZNF44
RABIF
ZSCAN12
ZNF648
LINC00324
RP11−440I14.2
BTBD9
LL22NC03−2H8.5
POPDC2
CFL1P5
SLC13A4
PDE10A
bP−2189O9.2
GNG7

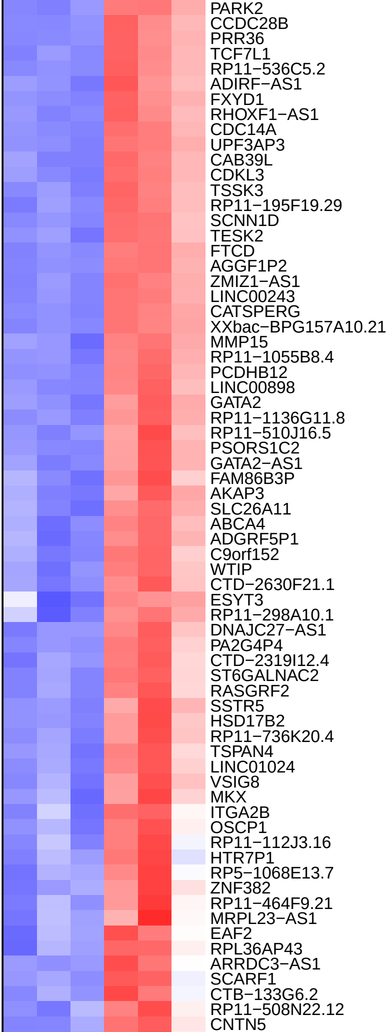
PARK2
CCDC28B
PRR36
TCF7L1
RP11−536C5.2
ADIRF−AS1
FXYD1
RHOXF1−AS1
CDC14A
UPF3AP3
CAB39L
CDKL3
TSSK3
RP11−195F19.29
SCNN1D
TESK2
FTCD
AGGF1P2
ZMIZ1−AS1
LINC00243
CATSPERG
XXbac−BPG157A10.21
MMP15
RP11−1055B8.4
PCDHB12
LINC00898
GATA2
RP11−1136G11.8
RP11−510J16.5
PSORS1C2
GATA2−AS1
FAM86B3P
AKAP3
SLC26A11
ABCA4
ADGRF5P1
C9orf152
WTIP
CTD−2630F21.1
ESYT3
RP11−298A10.1
DNAJC27−AS1
PA2G4P4
CTD−2319I12.4
ST6GALNAC2
RASGRF2
SSTR5
HSD17B2
RP11−736K20.4
TSPAN4
LINC01024
VSIG8
MKX
ITGA2B
OSCP1
RP11−112J3.16
HTR7P1
RP5−1068E13.7
ZNF382
RP11−464F9.21
MRPL23−AS1
EAF2
RPL36AP43
ARRDC3−AS1
SCARF1
CTB−133G6.2
RP11−508N22.12
CNTN5

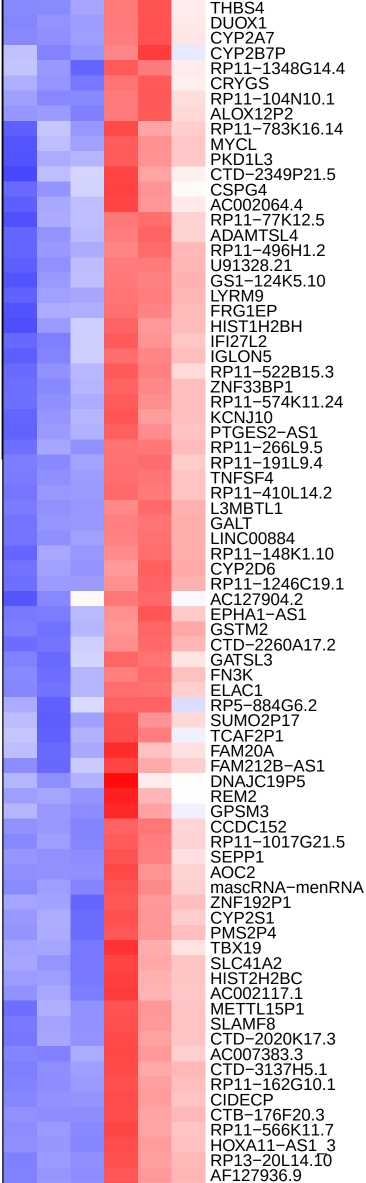
THBS4
DUOX1
CYP2A7
CYP2B7P
RP11−1348G14.4
CRYGS
RP11−104N10.1
ALOX12P2
RP11−783K16.14
MYCL
PKD1L3
CTD−2349P21.5
CSPG4
AC002064.4
RP11−77K12.5
ADAMTSL4
RP11−496H1.2
U91328.21
GS1−124K5.10
LYRM9
FRG1EP
HIST1H2BH
IFI27L2
IGLON5
RP11−522B15.3
ZNF33BP1
RP11−574K11.24
KCNJ10
PTGES2−AS1
RP11−266L9.5
RP11−191L9.4
TNFSF4
RP11−410L14.2
L3MBTL1
GALT
LINC00884
RP11−148K1.10
CYP2D6
RP11−1246C19.1
AC127904.2
EPHA1−AS1
GSTM2
CTD−2260A17.2
GATSL3
FN3K
ELAC1
RP5−884G6.2
SUMO2P17
TCAF2P1
FAM20A
FAM212B−AS1
DNAJC19P5
REM2
GPSM3
CCDC152
RP11−1017G21.5
SEPP1
AOC2
mascRNA−menRNA
ZNF192P1
CYP2S1
PMS2P4
TBX19
SLC41A2
HIST2H2BC
AC002117.1
METTL15P1
SLAMF8
CTD−2020K17.3
AC007383.3
CTD−3137H5.1
RP11−162G10.1
CIDECP
CTB−176F20.3
RP11−566K11.7
HOXA11−AS1_3
RP13−20L14.10
AF127936.9

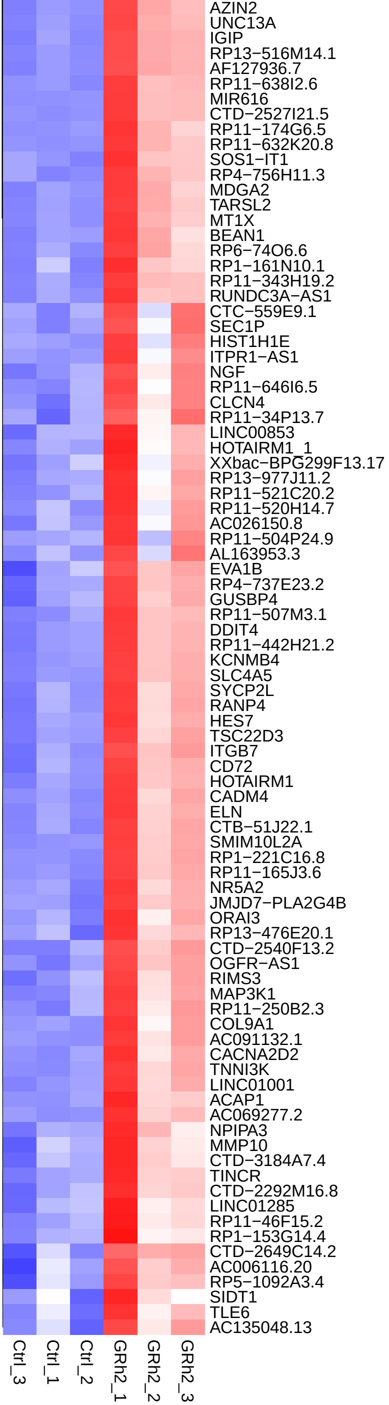
AZIN2
UNC13A
IGIP
RP13−516M14.1
AF127936.7
RP11−638I2.6
MIR616
CTD−2527I21.5
RP11−174G6.5
RP11−632K20.8
SOS1−IT1
RP4−756H11.3
MDGA2
TARSL2
MT1X
BEAN1
RP6−74O6.6
RP1−161N10.1
RP11−343H19.2
RUNDC3A−AS1
CTC−559E9.1
SEC1P
HIST1H1E
ITPR1−AS1
NGF
RP11−646I6.5
CLCN4
RP11−34P13.7
LINC00853
HOTAIRM1_1
XXbac−BPG299F13.17
RP13−977J11.2
RP11−521C20.2
RP11−520H14.7
AC026150.8
RP11−504P24.9
AL163953.3
EVA1B
RP4−737E23.2
GUSBP4
RP11−507M3.1
DDIT4
RP11−442H21.2
KCNMB4
SLC4A5
SYCP2L
RANP4
HES7
TSC22D3
ITGB7
CD72
HOTAIRM1
CADM4
ELN
CTB−51J22.1
SMIM10L2A
RP1−221C16.8
RP11−165J3.6
NR5A2
JMJD7−PLA2G4B
ORAI3
RP13−476E20.1
CTD−2540F13.2
OGFR−AS1
RIMS3
MAP3K1
RP11−250B2.3
COL9A1
AC091132.1
CACNA2D2
TNNI3K
LINC01001
ACAP1
AC069277.2
NPIPA3
MMP10
CTD−3184A7.4
TINCR
CTD−2292M16.8
LINC01285
RP11−46F15.2
RP1−153G14.4
CTD−2649C14.2
AC006116.20
RP5−1092A3.4
SIDT1
TLE6
AC135048.13
Ctrl_3
Ctrl_1
Ctrl_2
GRh2_1
GRh2_2
GRh2_3
